# Improved discovery of genetic interactions using CRISPRiSeq across multiple environments

**DOI:** 10.1101/gr.246603.118

**Published:** 2019-04

**Authors:** Mia Jaffe, Adam Dziulko, Justin D. Smith, Robert P. St.Onge, Sasha F. Levy, Gavin Sherlock

**Affiliations:** 1 Department of Genetics, Stanford University School of Medicine, Stanford, California 94305, USA;; 2 Joint Initiative for Metrology in Biology, Stanford, California 94305, USA;; 3 SLAC National Accelerator Laboratory, Menlo Park, California 94025, USA;; 4 Laufer Center for Physical and Quantitative Biology, Stony Brook University, Stony Brook, New York 11794, USA;; 5 Stanford Genome Technology Center, Stanford University, Palo Alto, California 94305, USA;; 6 Department of Biochemistry, Stanford University School of Medicine, Stanford, California 94305, USA;; 7 National Institute of Standards and Technology, Gaithersburg, Maryland 20899, USA

## Abstract

Large-scale genetic interaction (GI) screens in yeast have been invaluable for our understanding of molecular systems biology and for characterizing novel gene function. Owing in part to the high costs and long experiment times required, a preponderance of GI data has been generated in a single environmental condition. However, an unknown fraction of GIs may be specific to other conditions. Here, we developed a pooled-growth CRISPRi-based sequencing assay for GIs, CRISPRiSeq, which increases throughput such that GIs can be easily assayed across multiple growth conditions. We assayed the fitness of approximately 17,000 strains encompassing approximately 7700 pairwise interactions in five conditions and found that the additional conditions increased the number of GIs detected nearly threefold over the number detected in rich media alone. In addition, we found that condition-specific GIs are prevalent and improved the power to functionally classify genes. Finally, we found new links during respiratory growth between members of the Ras nutrient–sensing pathway and both the COG complex and a gene of unknown function. Our results highlight the potential of conditional GI screens to improve our understanding of cellular genetic networks.

Large-scale interrogation of biological systems across environmental perturbations has been invaluable to our understanding of molecular and systems biology. Examples of studies across perturbations in *Saccharomyces cerevisiae* include genome-scale measurements of RNA (Gasch et al. 2000) and protein levels (Newman et al. 2006), as well as the effects of individual gene-loss on growth (Hillenmeyer et al. 2008). Large-scale perturbation studies of genetic interactions (GIs) provide potentially fertile ground to uncover new biology, in which a GI is defined as an unexpected double-mutant fitness, based on the fitness of the corresponding single mutants (Phillips et al. 2000). A GI can be either positive or negative, depending on whether the observed double-mutant fitness is, respectively, higher or lower than expectation. GI screens have been a crucial tool for identifying new gene functions, because genes with similar functions have been shown to have correlated GI profiles (Schuldiner et al. 2005; Pan et al. 2006; St Onge et al. 2007; Costanzo et al. 2010). Recently, screens for higher-order GIs have yielded additional novel insights (Kuzmin et al. 2018). However, the majority of GI screening has been performed in a single growth condition, meaning that GIs that are specific to other conditions remain largely undiscovered. Indeed, a handful of GI perturbation studies performed in yeast and other organisms have uncovered many new GIs (St Onge et al. 2007; Musso et al. 2008; Bandyopadhyay et al. 2010; Guénolé et al. 2013; Barker et al. 2015; Fischer et al. 2015; Martin et al. 2015; Kumar et al. 2016), suggesting that further conditional GI screens may reveal the function of uncharacterized genes (∼10% of the yeast genome) or discover new functions of those already characterized. In addition to functional discovery, further conditional GI screens will reveal the extent to which the GI network is dynamic, and provide information as to which parts of the network change in response to a particular perturbation.

The most widely used technique to perform GI screens in yeast, synthetic genetic array (SGA), uses robotic systems to generate experimental strains through several rounds of mating and selection (Tong et al. 2001). SGA requires that individual strains be kept spatially segregated, and as a result, large collections are impractical to store and difficult to reassay under new conditions. Pooled assays provide a potentially powerful alternative, because they require significantly less space and resources to generate, store, and subsequently assay across conditions (Pan et al. 2004; Decourty et al. 2008; Jaffe et al. 2017; Díaz-Mejía et al. 2018). Recently, we introduced a DNA barcode-fusion–based method, iSeq, to perform GI screens in a pooled format (Jaffe et al. 2017). Although this approach made it possible to easily retest the fitness across multiple conditions, it was limited both by the two rounds of arrayed mating and selection and by the influence of segregating variation and de novo mutations that occur during strain generation, which impacted reproducibility. A related method has since been introduced, also relying on barcode fusion, that increases throughput of strain generation by performing a pooled mating to generate double mutants (Díaz-Mejía et al. 2018). However, whole-genome sequencing, in these and other studies, has shown that spontaneous mutations occur often during strain generation, leading to confounding genetic variation between ostensibly replicate strains (van Leeuwen et al. 2016; Jaffe et al. 2017; Díaz-Mejía et al. 2018). These types of mutations may be partly responsible for the relatively low reproducibility previously observed across replicate SGA screens (Schuldiner et al. 2005; Jasnos and Korona 2007; Dodgson et al. 2016).

To minimize the occurrence and selection of spontaneous mutations, we turned to an inducible system of genetic knockdown, CRISPR interference (CRISPRi). CRISPRi relies on the expression of a single guide RNA (gRNA), which localizes a catalytically inactive *Streptococcus pyrogenes* dCas9 protein to the promoter of a target gene to knock down its expression by blocking transcription (Qi et al. 2013). CRISPRi has been adopted in many model systems, and either the gRNA or dCas9 expression can be placed under an inducible promoter (Smith et al. 2016; Liu et al. 2017). In yeast, dCas9 fused with the transcriptional repressor Mxi1 has yielded target knockdown levels of up to 53-fold for endogenous targets (Smith et al. 2016; Gilbert et al. 2013). Because gene expression is repressed only after induction of the CRISPRi system, essential genes can be targeted. Furthermore, one can study a gradient of effects by including, for each target gene, multiple gRNA sequences that vary in their knockdown efficiencies (Peters et al. 2016; Deaner and Alper 2017; Liu et al. 2017; Smith et al. 2017). Although CRISPRi screens in yeast have thus far focused mainly on single-gene knockdowns (Deaner and Alper 2017; Smith et al. 2017; Vanegas et al. 2017), two studies report successfully using CRISPRi to test for GIs between 16 genes in bacteria (Peters et al. 2016) and 107 genes in mammalian cells (Du et al. 2017). Additionally, there have been several GI studies in mammalian systems that use the catalytically active Cas9 enzyme, further illustrating the biological utility of such combinatorial screens (e.g., Dixit et al. 2016, Wong et al. 2016, Han et al. 2017). Here we introduce a novel pooled-growth CRISPRi-based method in yeast, CRISPRiSeq, which we used to assay for GIs between approximately 7700 gene pairs across five experimental conditions using approximately 17,000 strains.

## Results

### Gene targets chosen for CRISPRiSeq

We have previously developed a method to quickly generate single-gene knockdown strains using CRISPRi and to assay for growth defects in a pooled format (Smith et al. 2016, 2017). Here, we aimed to extend this framework to conditional screening of GI networks. First, we selected and pooled 760 single CRISPRi strains with a range of known growth defects (see Supplemental Methods; Smith et al. 2017). Together, the 760 single CRISPRi strains carry gRNAs targeting 403 essential genes and 56 genes involved in respiration (Supplemental Table S1), spanning 97 GO-Slim Biological Process annotations (Hong et al. 2008), with up to five unique gRNAs per gene. In contrast to studies that use newly designed guide sequences (typically including up to 10 targeting sequences per gene), our guides had been previously validated (Smith et al. 2017), which allowed us to decrease the number of guides targeting each gene and thereby increase the number of unique genes included in this study. We also generated 22 additional control starting pool strains carrying nontargeting guides (see Supplemental Methods). Each starting pool strain carries a constitutively expressed dCas9-Mxi1 fusion, which has defective nuclease activity, a constitutively expressed Tet repressor protein (TetR), as well as a gRNA under an ATc-inducible promoter integrated at the neutral *YBR209W* locus (Breslow et al. 2008; Kao and Sherlock 2008; Pelechano et al. 2013). Because gRNA expression is inducible, a growth defect conferred by CRISPRi-induced gene-expression knockdown is unlikely to occur in the absence of ATc, which minimizes the selection of suppressor mutations during strain generation and before fitness measurement. Furthermore, in contrast to systems that express gRNAs directly off of plasmids or systems that integrate gRNAs randomly in the genome, our system of genomic integration of a single copy of each gRNA at a known genomic location eliminates experimental noise due to gRNA copy number variation and variability in integration site.

Next, a total of 17 genes was chosen as “query” targets, and plasmids carrying ATc-inducible gRNAs and a 25-nucleotide (nt) DNA barcode identifier (BC) were generated as described in the Methods, with up to two unique gRNAs chosen for each gene target. Query plasmids for three nontargeting control guides were also generated. The query gene targets included three proteasome, five secretory pathway, three small ribosome biogenesis, and three large ribosome biogenesis genes. Previous work has shown that the chosen query gene targets sharing the same annotated biological process show GIs with similar subsets of the genes targeted in our starting pool strains (Costanzo et al. 2016). We also chose to include two uncharacterized genes (*YCR016W* and *YLR050W*) and *SAP30*, a member of the Rpd3L histone deacetylation complex (Supplemental Table S1).

### Characterizing gRNA efficacy using a GFP-based assay

**Figure 1.**
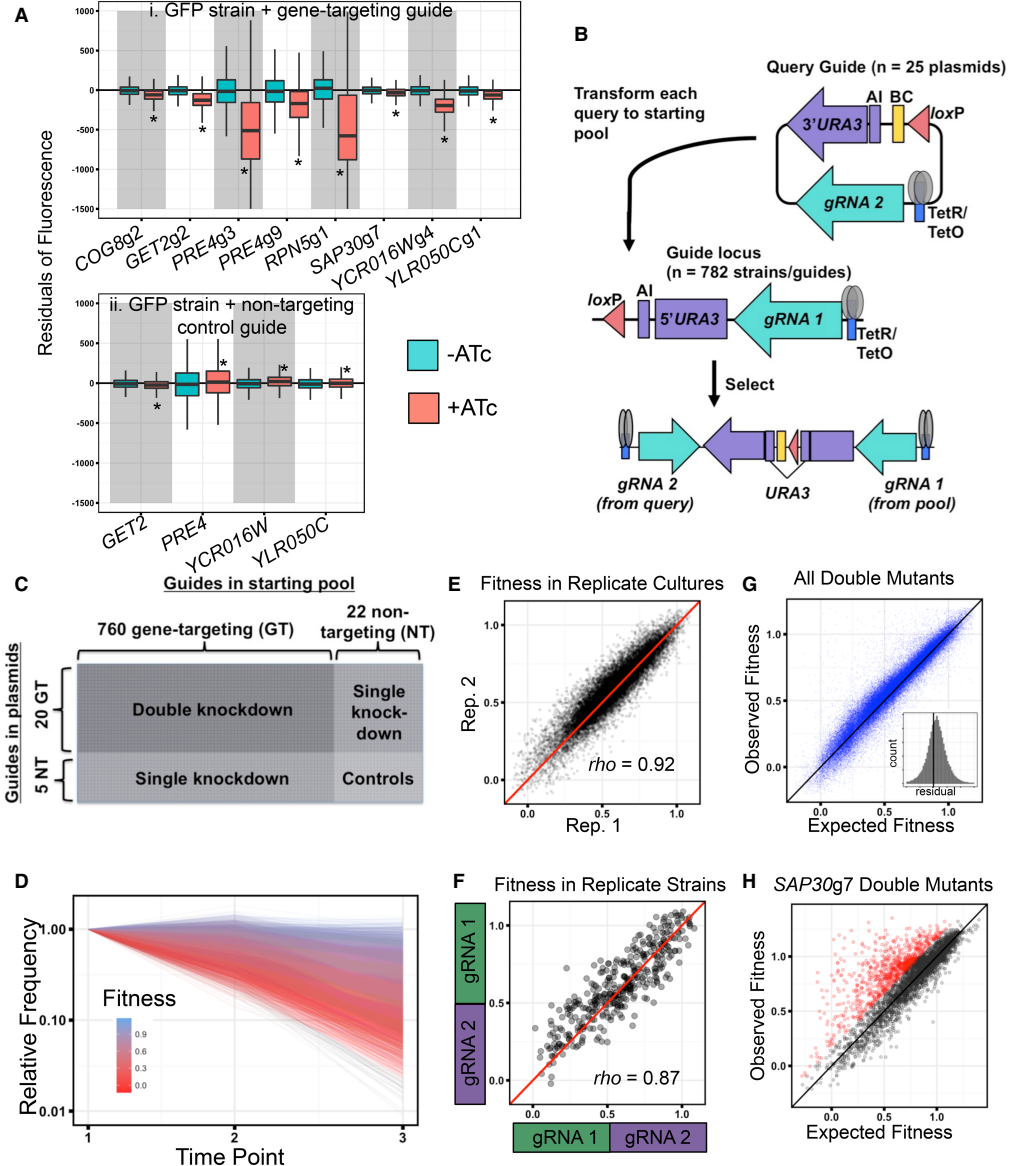
Rapid double CRISPRi strain generation and parallel fitness measurement of approximately 17,000 mutants. (*A*) Boxplots depicting the distributions of residuals for GFP-tagged strains carrying either a gene-targeting (*i*), or nontargeting control (*ii*) CRISPRi plasmid and grown for 4 h in inducing (+ATc; red) or noninducing (−ATc; turquoise) conditions. Residuals were calculated from a loess regression fit to the uninduced sample (for details, see Methods). Asterisks indicate significant changes in distributions upon induction with ATc (*P* < 10^−8^ by Student's *t*-test). (*B*) Constructs used during the generation of double CRISPRi strains via transformation of a query plasmid into a pool of starting strains. (AI) Artificial intron; (BC) DNA barcode. (*C*) Guide combinations resulting in the single-, double-, and no-gene knockdown control strains generated for this study (for details, see Methods). (*D*) Representative normalized relative frequency trajectories from pooled fitness assay of approximately 17,000 strains in one replicate culture of YPD; time points are collected every 24 h. Trajectories are used to estimate fitness. Raw count data are derived from amplicon sequencing. (*E*,*F*) Reproducibility of fitness from pooled assay across two replicate YPD cultures with 24-h transfer time (*E*; approximately 17,000 strains) and replica strains carrying the same two gRNA sequences in reverse orientations (*F*; 78 pairs of strains in each of five conditions). Spearman's *rho* for each comparison is displayed. (*G*) Observed double-mutant fitness compared with expectation calculated using the multiplicative model. *Inset* is a histogram of residuals from expectation for each observed double mutant; vertical line is at *x* = 0. (*H*) Same plot as in *G*, but only for *SAP30*g7 containing double knockdowns. Red points have a *z*-score >2. For *E*–*H*, diagonal lines are *y* = *x*. For *F*–*H*, data are shown from all five growth conditions.

Before generating double CRISPRi strains, we developed an assay to functionally validate CRISPRi-mediated target protein knockdown, a phenotype more proximal to eventual gene function than mRNA level. gRNAs present in our starting pool had previously been validated for activity by either a fitness assay or qPCR (Smith et al. 2017); however, a subset of the gRNA sequences chosen for this study was newly designed and thus had not been previously validated. To validate these remaining gRNAs, we obtained seven strains from the yeast GFP fusion collection (Huh et al. 2003) and then derived two or three strains from each by introducing one or two gene-targeting and one nontargeting control CRISPRi plasmids to each. Flow cytometry analysis on 50:50 mixtures of each GFP collection strain with a nonfluorescent control (BY4741) revealed that four of the strains had high enough GFP-tagged protein expression to form a distinct population from the non-GFP control when plotting fluorescence against cell size (Supplemental Fig. S1A). The remaining three GFP strain populations partially overlapped with the nonfluorescent control strain, but expression levels were still high enough to detect potential knockdown upon CRISPRi induction, as these strains had reportedly detectable GFP levels (Newman et al. 2006). Visual inspection of plots of the fluorescence versus cell size of CRISPRi-induced versus -uninduced samples revealed a marked reduction in fluorescence upon CRISPRi induction (Supplemental Fig. S1B). However, in all cases, the reduction in the induced CRISPRi sample was not a complete ablation of GFP (i.e., fluorescence levels were always distinctly higher than the non-GFP control); this could be due in part to the GFP fusion proteins having longer half-lives than the time of induction (4 h), with the decrease in fluorescence likely because of the dilution of starting protein levels during cell division. We quantified this reduction by calculating residuals for each cell in a sample from a loess regression fit to the uninduced sample, and showed that all seven strains carrying a gene-targeting plasmid had a significant decrease in GFP compared with that of an uninduced control (Fig. 1A). No significant decrease in GFP was detected in three of the four nontargeting control samples tested (Fig. 1A).

### Rapid generation and conditional screening of more than 17,000 double CRISPRi strains

We next developed a protocol to rapidly generate double CRISPRi strains compatible with a sequencing-based conditional pooled fitness assay. Previously, we have built collections of approximately 500,000 barcoded yeast using Cre-mediated recombination to integrate a barcoded plasmid library (Levy et al. 2015). Here, we applied the same principle to add second gRNAs to our CRISPRi strain library. The guide locus (*YBR209W*) of each strain of the CRISPRi library carried a gRNA, a 5′ half of the *URA3* selectable marker, an artificial intron, a *lox*P variant (*lox*71) site (Fig. 1B), and a galactose inducible Cre recombinase gene. Each query plasmid contained another *lox*P variant (*lox*66) site, a unique DNA barcode, a gRNA, the complementary 3′ half of the *URA3* marker, and an artificial intron. After transformation of a query plasmid into the starting pool, and induction of Cre-recombinase, successful genomic integration of the plasmid can be selected by growth on media lacking uracil (Levy et al. 2015; Jaffe et al. 2017; Smith et al. 2017). We transformed each of our 25 query plasmids into one or more pools consisting of subsets of our 782 starting strains. This strain generation strategy was implemented to span all possible gene–gene combinations while leaving out a subset of guide–guide combinations. This strategy was necessary to remove sequencing artifacts during a downstream analysis step, which requires that not all possible double guide combinations are present in the pool (for details, see Methods). We collected an average of 52 plasmid insertion events per resulting double CRISPRi strain. In total, we aimed to generate 17,069 double CRISPRi strains, which included 15,200 double-gene knockdowns, as well as 1769 single-gene knockdown and 100 no-gene knockdown controls (for details, see Methods) (Fig. 1C). During pooled growth, each strain can be uniquely identified by sequencing amplicons of the *YBR209W* guide locus carrying the gRNA sequence derived from the starting pool strain and DNA barcode derived from the query plasmid.

Having generated a unique genotype identifier for each double CRISPRi strain allowed us to easily and quantitatively measure the fitness of each strain in parallel and across multiple growth conditions. We pooled all double CRISPRi strains, propagated the pool by serial batch culture (see below), sequenced gRNA/barcode amplicons at the end of each of the three growth cycles, and used changes in amplicon frequencies as a proxy for fitness (described in Methods and Supplemental Methods) (Fig. 1D; see diagram in Supplemental Fig. S2; Jaffe et al. 2017). We tested five growth conditions in triplicate. Four conditions were performed by diluting 1:4 every 24 h (about two generations per cycle): YPD24hr (fermentative growth in rich media), YPD37C (high temperature, 37°C), YPEG (respiratory growth), and SC-URA (media lacking uracil). One condition was performed by diluting 1:8 every 48 h (about three generations per cycle): YPD48hr (fermentative growth in rich media). Only 14 of the 17,069 strains (0.08%) were undetected at time point zero (before the start of the fitness assay), and we observed relatively even coverage across strains, with just a 2.4-fold difference between first and third quartile coverage values (Supplemental Fig. S3). Overall, we estimated fitness in all five conditions for 85% of our double-gene knockdown strains, whereas in any single condition, fitness was unmeasurable for between 0.5% and 11.7% of strains because of low relative frequency in at least one sample (for details on thresholds used, see Supplemental Methods).

### Assay reproducibility and characteristics

To characterize the reproducibility in our screen, we first examined fitness correlations of all strains across replicate cultures and generally observed high correlations (mean Spearman's *rho* for all comparisons = 0.90, median SD across replicates = 0.053) (Fig. 1E; Supplemental Fig. S4). Independently constructed strains with the same gRNA pair also showed high fitness correlations, indicating that strain construction artifacts were rare (Spearman's *rho* = 0.87 and 0.92, median SD of 0.034 across single-gene knockdown replicate strains) (Fig. 1F; Supplemental Fig. S5A). Furthermore, fitness of single-mutant strains carrying a nontargeting control guide from the query plasmid correlated well (*rho* = 0.53 and 0.68, for YPD and YPEG, respectively) with our previously published data using strains without a nontargeting control (Smith et al. 2017; Supplemental Fig. S5B). Notably, 13 strains carried two copies of an identical guide sequence, and in 96.9% of comparisons (126 of 130), the fitness defect of the strain carrying two guide copies was greater than the defect present in the single-mutant control (Supplemental Fig. S5C), which further supports our finding in Supplemental Figure S1B that CRISPRi gene knockdown is only partial.

### Identification of GIs

Next, we used these data to identify significant GIs in each of our five growth conditions. Standard practice is to use a multiplicative model to define the expected double-mutant fitness, which is the product of the two single-mutant fitnesses (Phillips et al. 2000). However, in most cases we observed a positive bias from the multiplicative expectation, suggesting this model was not appropriate (Fig. 1G): The distribution of interaction scores calculated was centered above zero (Fig. 1G, inset). The magnitude of this positive bias varied both by the query guide and the environmental condition (Supplemental Fig. S6). Taking these observations into account and operating under the assumption that most gene pairs do not genetically interact, we recalculated the expectation empirically using a linear model for each group of double mutants derived from the same query guide in each condition as has been performed previously for pooled GI screens in mammalian systems (see Methods; Supplemental Fig. S6) (Bassik et al. 2013). For double mutants derived from the query guide targeting Rpd3L histone deacetylase complex member *SAP30*, a linear model did not fit the data (Fig. 1H). We observed that in YPD and YPEG, a significant proportion of the growth defects induced by the guides derived from the starting pool strains was alleviated upon the additional knockdown of *SAP30* (25%–42% of guides). Thus, for *SAP30*g7-derived double mutants, we calculated the interaction score as the deviation of the double-mutant fitness from expectation based on the multiplicative model.

To assess whether our GI scores were reasonable, we first used data for the 78 pairs of strains carrying the same two gRNAs in reverse orientation. Although the majority of these gene pairs did not show a significant GI in any of the five growth conditions, we nevertheless observed modest agreement between the GI scores for replicate strains in each condition (Spearman's *rho* = 0.39) (Supplemental Fig. S6F, gray box indicates significance threshold). Furthermore, two of the strongest negative GIs we observed in rich media were between *SEC22* and either *BET1* or *SED5*, gene pairs known to be synthetically lethal (Newman et al. 1990; Sacher et al. 1997), suggesting that our screening technology is capable of detecting previously identified GIs. To investigate the mechanism underlying the high frequency of positive interactions with *SAP30*, we used qPCR to test whether *SAP30* knockdown results in an increase in mRNA expression for three gene targets observed to positively interact with *SAP30* (see Supplemental Methods). Two of these genes increased in expression upon *SAP30* knockdown, suggesting *SAP30* knockdown can suppress the effects of a second guide by increasing mRNA expression (Supplemental Fig. S7).

### Increased GI discovery from screening in multiple conditions

**Figure 2.**
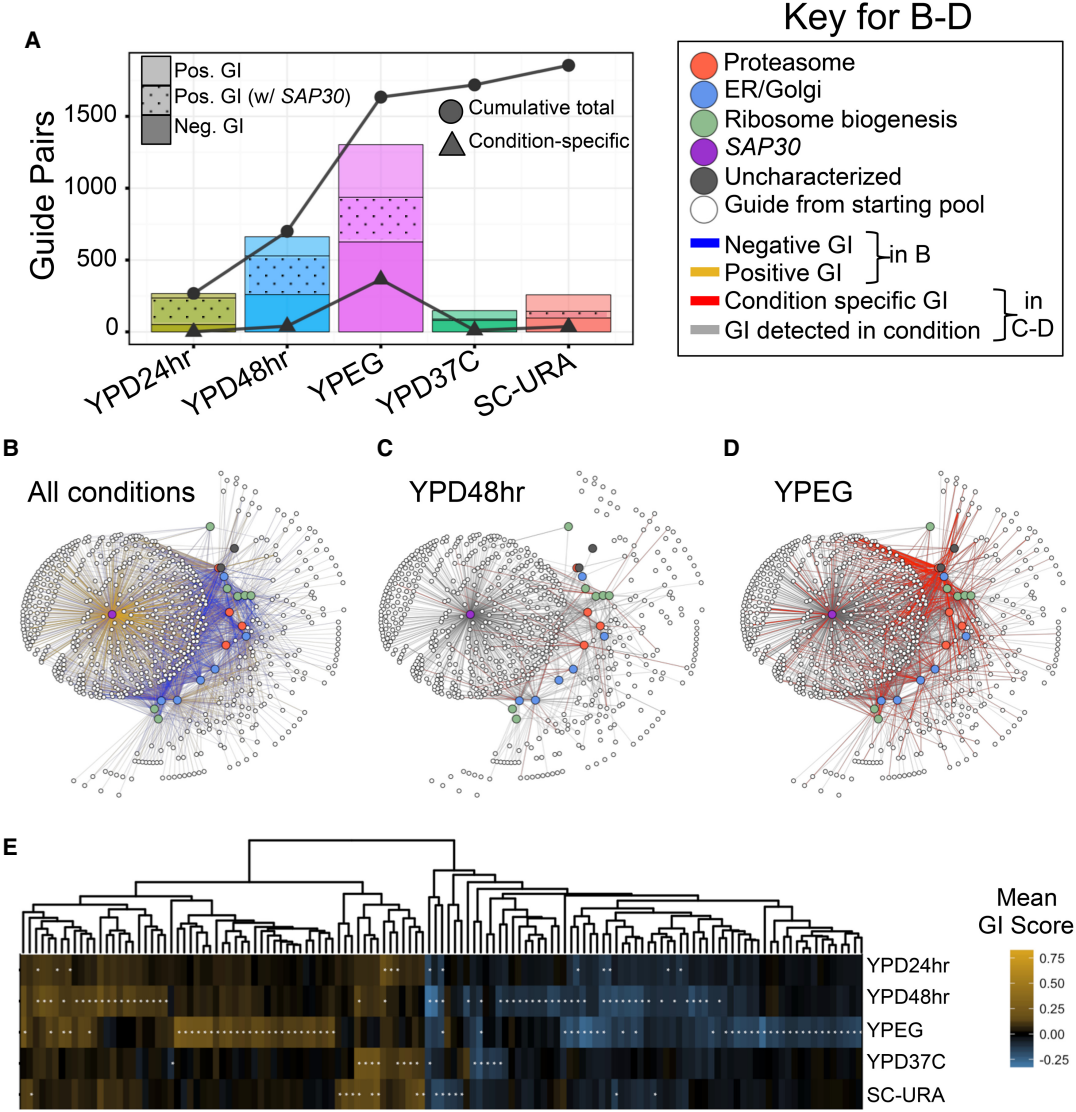
Condition-specific versus condition-independent GIs detected via CRISPRiSeq in five conditions. (*A*) Bar plot depicting number of significant GIs detected between guide pairs in each of five growth conditions tested. Negative and positive GIs are distinguished by shading, and positive interactions with *SAP30* have a dotted pattern. Lines indicate the cumulative total of unique interactions (circles) and condition-specific interactions (triangles). (*B*–*D*) Network diagrams of significant interactions measured in at least one condition (*B*), YPD media with a 48-h transfer time (*C*) or YP + ethanol + glycerol media (*D*). Nodes represent guides; query guides are colored by their target's biological process. In *B*, blue edges are negative interactions; gold are positive. In *C*,*D*, gray edges are interactions detected in the condition; red are condition-specific. (*E*) Heatmap depicting mean GI score across three to 10 replicate strains for gene pairs passing significance in at least one condition (95% CI nonoverlapping with absolute *z*-score of one; denoted with asterisk). For gene pair labels, see Supplemental Figure S8.

We calculated the number of significant GIs in each condition using a significance threshold of an absolute *z*-score greater than two (Fig. 2A, barplot). Depending on the condition, between 1.2% and 10.2% of guide pairs interacted significantly, and the cumulative proportion of unique pairs increased with each additional condition tested (Fig. 2A, line). In total, 14.7% of the 12,715 guide pairs for which we had a GI estimate for all conditions showed an interaction in at least one condition (Fig. 2B), representing 20.7% of the 6841 unique gene pairs in our screen. The *SAP30* targeting query guide, which we previously noted rescued many single-mutant fitness defects, accounted for the majority of significant positive interactions in both of the YPD conditions. After removing these pairs, a total of 16.9% of the remaining 6391 gene pairs interacted. To further validate our choice in using a z-score threshold to identify GIs, we reanalyzed the data using a method to call significance that instead was based on false-discovery rate (Supplemental Note 1; Supplemental Table S3). Similar to our *z*-score–based threshold, we observed an increase in the number of GIs with the number of conditions screened, suggesting this result was not an artifact of using a *z*-score–based threshold to call significance.

To understand the frequency of condition-specific versus condition-independent GIs, we first defined condition-specific GIs as guide pairs with an absolute *z*-score greater than two in one condition and less than one in all other conditions. In total, 3.6% of guide pairs had a condition-specific GI, and most of these were observed in YPEG (Fig. 2A,C,D). Pairwise comparisons of GI scores between conditions revealed a general positive correlation across conditions, with only 25 GI scores switching sign with condition (Supplemental Fig. S8A). Most GIs observed in YPD24hr were also observed in YPD48hr (229 of 267, 85.8%), although the magnitudes of the scores generally increased with a longer time between transfers (mean fold increase in score of 1.4 in 48 h for significant GIs in both conditions) (Supplemental Fig. S8A). When comparing the two YPD transfer conditions (24 h and 48 h) to YPEG, we saw a slightly stronger correlation between GI scores with the longer transfer time to YPEG (*R*^2^ = 0.52 vs. 0.58) (Supplemental Fig. S8A), possibly reflecting an increased role of respiratory growth during 48-h growth cycles.

To more confidently identify significantly interacting gene pairs in each condition, we aggregated estimates from replicate strains carrying guide pairs that targeted the same gene pair. In total, 198 gene pairs had a significant GI in at least one condition (of the 1711 total gene pairs measured with at least three replicate strains; 95% CI nonoverlapping with an absolute *z*-score score greater than one) (Fig. 2E; Supplemental Fig. S8B).

### Additional environments improve biological classification

**Figure 3.**
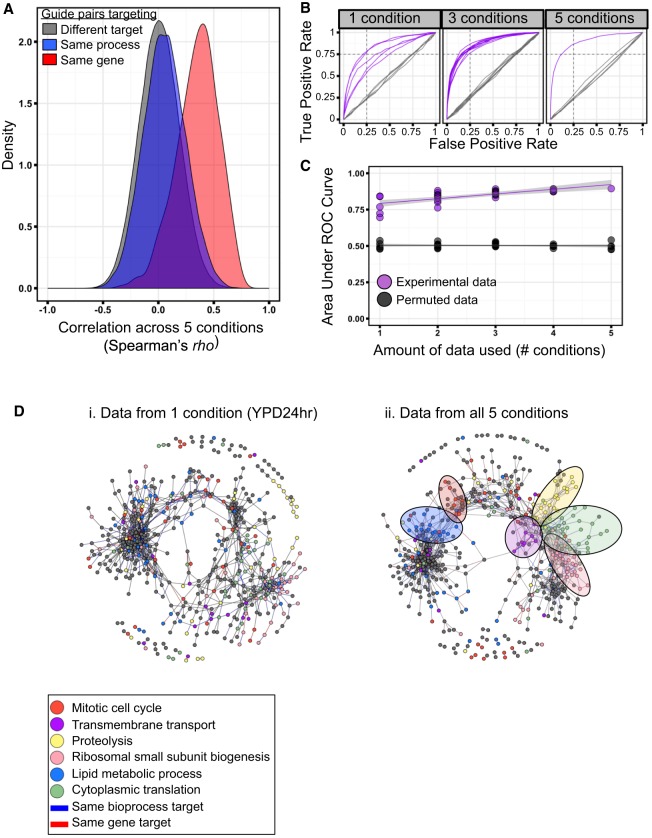
Screening in multiple conditions improves functional classification. (*A*) Density distributions depicting GI profile similarity of pairs of guides targeting the same gene (*n* = 422), targeting the same bioprocess (*n* = 36,029), or having different gene and bioprocess targets (*n* = 239,202). (*B*) Receiver operating characteristic (ROC) curves depicting true-positive and false-positive rates when predicting whether a starting pool guide pair targets the same gene based on GI profile similarity using different thresholds of Spearman's *rho*. Analysis was performed on all possible subsets of the data set for one and three conditions. Experimental data are purple; permuted data, gray. (*C*) The area under the ROC curves from *B*. (*D*) Network diagrams built from data from one condition (YPD24hr; *i*) or from the entire data set (*ii*). Nodes represent starting pool guides and are colored by bioprocess of target gene (for bioprocesses where a region of enrichment in the network was detected). Edges link nodes with similar GI profiles and are colored by whether the guide pair targets the same gene (red) or same bioprocess (blue).

We used our entire conditional GI data set to characterize biological function. As a first test, we compared different query gRNA sequences that target the same gene (either *SEC22* or *PRE4*) and observed that GI scores were correlated across the 760 paired gRNAs in all five growth conditions (*R*^2^ = 0.71 for *SEC22* and 0.26 for *PRE4*, both at or above the 75th percentile of pairwise comparisons of guides targeting different genes). For the remaining pairwise comparisons, query guides targeting the same bioprocess tended to have higher GI profile similarity than those with different targets (*P* = 3.4 × 10^−9^ Wilcoxon rank-sum test) (Supplemental Fig. S9). The GI profiles of *COG8* and the uncharacterized ORF *YCR016W* were among the most correlated in our set (*R*^2^ of 0.74) (Supplemental Fig. S9), suggesting a potentially similar role in the cell. This high degree of similarity was driven by approximately 30 shared positive interactions in YPEG, which were enriched (*P* = 4.7 × 10^−5^, hypergeometric test) for guides targeting genes involved in signaling, including Ras-, RAM-, and Tor-pathway signaling, and two guanine–nucleotide exchange factors involved in translation. We next asked if the 760 guides in the starting pool could be classified by function using GIs with only the 20 query guides. By using our entire data set, which includes all five conditions, we observed a clear increase in the distributions of GI profile correlations for guides targeting the same gene or biological process, as defined by GO-Slim annotations, over those with different targets (Fig. 3A). However, it was unclear if assaying multiple conditions had improved the separation between the distributions. To investigate this possibility, we partitioned GI scores by which condition they were measured in and calculated a receiver operating characteristic (ROC) curve for each possible subset of one, two, three, four, or five conditions. For this analysis, ROC curves diagnose the classification of whether two guides target the same gene using the correlation in GI scores by plotting the “true-positive rate” versus the “false-positive rate” at various thresholds of Spearman's *rho* (Fig. 3B). We observed a clear increase in area under the curve (AUC), or classification performance, with the number of conditions used (Fig. 3C, purple points). We also performed the same analysis on all possible subsets of conditions from the entire data set after randomly permuting the data for each query guide in each condition and observed all power to classify function was lost (Fig. 3C, gray points). To determine whether the improvement in classification with additional conditions is a general property of having more data rather than more conditions, we randomly selected 20, 40, 60, or 80 query genes (ignoring which condition they were measured in and taking eight replicate samplings each) and performed the same analysis. Again, we observed clear increases in AUC with the amount of data used, indicating that functional classification generally improves with more GI data (Supplemental Fig. S10, green points). Precision-recall curves, quantifying the true-positive rate versus the positive predict power, showed a similar trend of improved classification with additional data (Supplemental Fig. S10). To visualize the impact of assaying more conditions and thus generating more GI data, we constructed GI profile correlation networks using data from one condition or using the entire data set. We observed qualitatively that using more conditions resulted in better clustering of gRNAs targeting genes in the same biological process, as defined by GO-Slim annotations (correlation threshold controlled for a 0.5% false-positive rate; see Methods) (Fig. 3D; Supplemental Fig. S11).

### Comparison of CRISPRiSeq to SGA

We compared our CRISPRi-based GI scores to the most comprehensive published GI study that uses SGA technology (Costanzo et al. 2016). There are many major experimental differences between the two screens that may limit the expected overlap: (1) We used CRISPRi to knock down transcription of the genes of interest, whereas SGA has used temperature-sensitive, dAMP (decreased abundance by mRNA perturbation) and gene deletion alleles; (2) we assayed in liquid culture instead of on agar plates; (3) we quantified fitness by competitive growth instead of by colony size; (4) we assayed several different growth conditions instead of one; (5) there are differences in strain background; and (6) the definition of GI used is slightly different (see Methods). After acknowledging these differences, to compare the data of Costanzo et al. (2016) to our data, we excluded gene pairs that included the highly suppressive *SAP30* guide (discussed more below), and characterized a gene pair as interacting in our study if we observed a significant GI (absolute *z*-score greater than two) in any of the five conditions assayed. A total of 5072 unique gene pairs were measured in both studies, and the majority showed no significant GI in either study (67.1%) (Supplemental Table S4). Of the remaining 1671 gene pairs, most observed GIs were unique to one study or the other: 45% were only observed in our data, whereas 41% were only observed by Costanzo et al. (2016). Only 149 gene pairs had a significant GI common to both studies, with the remaining 74 gene pairs having switched signs of GI between the studies. Although there was no significant overlap between the lists of gene pairs with positive interactions, the overlap between those with negative interactions was significant (*P* = 2.5 × 10^−7^, hypergeometric test).

### Validation of condition-specific GIs

**Figure 4.**
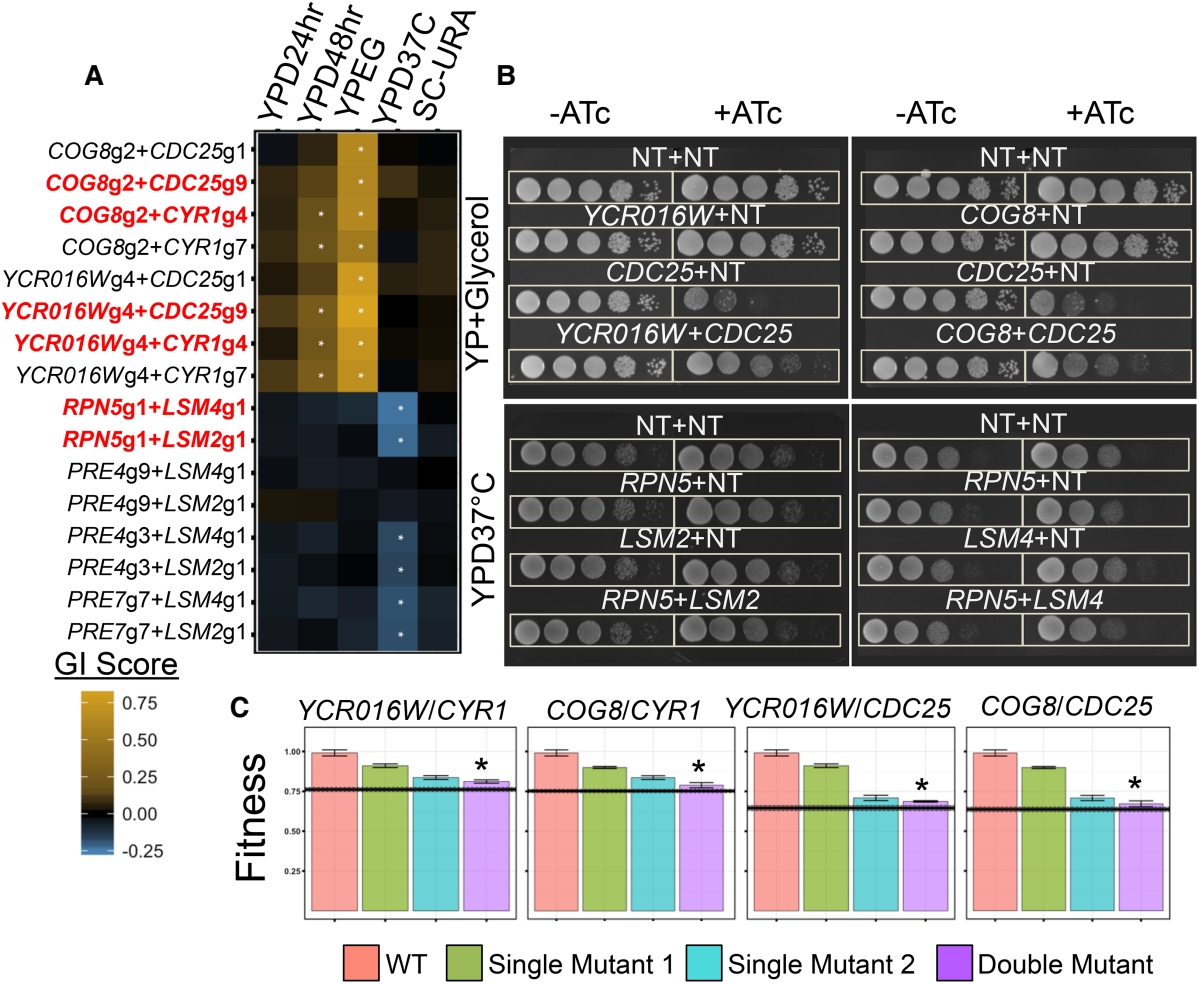
Validating condition-specific GIs detected in pooled screen. (*A*) Heatmap depicting GI scores derived from pooled fitness assay in each of five growth conditions for 17 guide pairs. Red text indicates strains reconstructed for validation, and asterisks signify absolute *z*-score greater than two. (*B*) Positive GIs for *COG8* and *YCR016W* with *CDC25* and negative GIs for *RPN5* with *LSM2* and *LSM4* are validated on spot assay plates in the presence of CRISPRi-inducing agent ATc. (ATc) Anhydrotetracycline, (NT) nontargeting control gRNA. (*C*) Positive GIs validated in monoculture for *COG8* and *YCR016W* with *CDC25* and *CYR1*. Error bars, 95% confidence intervals around mean. Horizontal line is 95% confidence interval around expected double-mutant fitness (using multiplicative model). Asterisks represent values passing significance based on nonoverlapping 95% confidence intervals of observed and expected double-mutant fitness.

To increase confidence in the new, CRISPRi-based, condition-specific GIs we identified, we validated a subset of these GIs using two low-throughput fitness assays: a spot assay and an assay based on optical density growth curves (see Supplemental Methods). We first validated interactions involving genes in the Ras-pathway, a highly conserved nutrient-sensing pathway involved in regulating cell growth (Broach 2012). In our pooled assay, we had observed that the severe fitness defects conferred by the knockdown of the Ras-pathway positive regulators, *CDC25* or *CYR1*, in the respiratory growth condition (YPEG), were almost completely rescued by the additional knockdown of either *COG8* or *YCR016W*, resulting in condition-specific positive GIs (Fig. 4A). In our spot assay, interactions were observed on YP + glycerol agar, but not on YPD agar, for two of the four gene pairs: *COG8*/*CDC25* and *YCR016W*/*CDC25* (Fig. 4B; Supplemental Fig. S12A). For the two gene pairs in which the assay did not validate an interaction (*COG8*/*CYR1* and *YCR016W*/*CYR1*), the *CYR1* single mutant did not show a strong growth defect on YPG or YPD agar, and therefore, there was no defect to rescue by additional knockdown of a second gene target (Supplemental Fig. S12B). In our OD-based assay, we observed positive interactions between all four gene pairs (*COG8*/*CDC25*, *YCR016W*/*CDC25*, *COG8*/*CYR1*, *YCR016W*/*CYR1*; significant by nonoverlapping 95% confidence intervals) (Figure 4C). The conserved oligomeric Golgi (COG) complex is involved in autophagy (Yen et al. 2010), and Ras-pathway activation inhibits autophagy (Budovskaya et al. 2004; Schmelzle et al. 2004), but no direct link between the Ras-pathway and COG complex has previously been reported.

We next validated newly discovered interactions between *LSM2* and *LSM4*, members of two highly conserved protein complexes involved in RNA processing and degradation (Bouveret et al. 2000), and the proteasome target genes *RPN5*, *PRE4*, and *PRE7.* A physical interaction has previously been reported between Lsm4 and Rpn5 (Yu et al. 2011), and our data suggest additional crosstalk between other members of the proteasome and RNA processing complexes. In agreement with our large-scale screen (Fig. 4A), we observed on YPD plates that the fitness benefit conferred by *LSM2* or *LSM4* knockdown at 37°C was ablated by the additional knockdown of *RPN5* (Fig. 4B; interaction somewhat less observable at 30°C in Supplemental Fig. S12A). We were unable to verify these interactions using our OD-based method, as neither *LSM2* nor *LSM4* knockdown showed a strong growth advantage in this assay, so depletion of such an advantage by knockdown of a second gene would not be observable (Supplemental Fig. S12C). In total, GIs were observed in at least one validation assay for all six of the gene pairs tested, and for instances in which the interaction was unconfirmed, it was likely because of a difference between the pooled fitness assay condition and the validation condition.

## Discussion

In this study, we rapidly generated and screened for GIs between approximately 7700 gene pairs in five conditions using CRISPRi technology. We showed that our estimates are highly reproducible and that condition-specific as well as condition-independent GIs are common. Building networks of GI profile similarity of the 760 starting pool guides against just 20 gene-targeting query guides effectively clustered many genes by biological function, confirming previous studies that GI profile networks are useful for gene annotation (Collins et al. 2007). Further, we found that functional annotation can be significantly improved by screening multiple conditions. Finally, we validated a subset of novel and condition-specific interactions, linking a previously uncharacterized gene and a member of the secretory pathway with multiple conserved signaling pathways during respiratory growth.

### Advantages of screening for GIs with double CRISPRi

Using CRISPRi technology to quantify GIs has several key advantages over traditional methods. First, for each condition we tested, fitness estimates were highly reproducible across replicate strains carrying the same guide sequence. Previously, we, and others, have reported that when generating strains for GI screening via mating and plate-based selection, suppressors or other mutations that can affect fitness commonly occur (van Leeuwen et al. 2016; Jaffe et al. 2017; Díaz-Mejía et al. 2018). In contrast, here, for the strains generated through pooled transformation that carry inducible gRNAs, we observed that the error on fitness across replicate strains is similar to the error across replicate cultures (Fig. 1E,F), although the error in GI scores across replicate strains was higher, which is unsurprising given that the error on GI score estimate is affected by each of the errors on the estimates of the respective single and double mutants (Supplemental Fig. S6F). Although suppressor mutations have been reported to occur at low frequencies in bacterial CRISPRi strains via frameshift mutations in the *dcas9* gene (Zhao et al. 2016; Liu et al. 2017), we minimized the potential effect of these mutations by collecting many representative transformants for each genotype during strain generation and by sequencing amplicons often and across few generations during pooled growth. However, we do note that satellite colonies were observed in low fitness strains on our spot assay, suggesting that suppressor mutations can and do occur at a low rate in our strains (e.g., see *CDC25* single mutant in Fig. 4B). To address this issue, future CRISPRi GI studies might consider incorporating an additional copy of *dcas9* in the genome of the ancestor strain, as has been performed in bacteria (Zhao et al. 2016), and/or by performing these assays under the selection for the *URA3* marker.

A second advantage of GI screening with CRISPRi is that knockdowns observe biological connections that may be hidden when using traditional gene knockout GI screens. For example, we found that a *SAP30* knockdown, a member of the Rpd3L histone deacetylation complex, partially rescued the fitness defects caused by many other gene knockdowns (∼42% in YPEG). One potential explanation for this finding is that *SAP30* knockdown globally increases mRNA levels by increasing acetylation of chromatin (Verdin and Ott 2015) and thereby reduces the impact of other gene knockdowns. A positive GI with *SAP30* through this mechanism would be unobservable with previous methods using deletion strains, because mRNA for a deleted gene is completely absent, so its expression levels cannot be modulated by changes in histone marks. This parallels positive GIs between hypomorphic alleles and knockdown or deletion of genes involved in mRNA stability and protein turnover (van Leeuwen et al. 2016). Indeed, coupling epigenetic profiling, such as nucleosome positioning and chromatin modifications, with CRISPRiSeq for chromatin modifiers across different conditions may highlight the dynamics of functional epigenetic marks. However, the high rate of *SAP30* positive interactions could also be explained by an inhibition of CRISPRi knockdown, as the transcriptional repressor Mxi1 fused to dCas9 has been shown to interact with another member of the Rpd3L complex, Sin3 (Gilbert et al. 2013). Our qPCR results (Supplemental Fig. S7) do not distinguish between potential mechanisms of suppression; however, we did observe the magnitude of positive GI increased with the gRNA target distance from the transcriptional start site (Supplemental Fig. S13), suggesting that *SAP30* inhibition reduces CRISPRi efficacy at distal gRNA targeting sites, whereas steric effects still mediate knockdown at more proximal sites.

Other advantages of CRISPRi GI screening are its scalability, portability, and ease of reproducibility, particularly for conditional screening. In *S. cerevisiae*, new starting pool strains can be taken from our existing collection or rapidly generated using the same technique used to generate this collection (Smith et al. 2017). New query plasmids can be generated by the straightforward protocols developed here or, for larger screens, by using more high-throughput methods of cloning. We estimate the cost to generate and verify 25,000 new guides sequences to be just $12,500. This number of guides would allow for screening GIs between all pairwise combinations of 700 genes, accounting for preliminary functional testing and replication. We have previously shown that at least 500,000 plasmids can be integrated into the yeast genome and tracked by Bar-seq, suggesting that CRISPRi GI screens of at least that size are possible. Once the upfront work of generating a pool of double CRISPRi strains is performed, the pooled fitness assay can be easily performed across conditions, and we estimate the cost of sequencing to be less than $0.02 per GI, per condition, or $9800 per replicate for a screen of 700 × 700 genes. In addition, because CRISPRi GI screens can be performed without first generating knockout libraries, as is required for traditional GI screens, it can be more easily ported to nonmodel organisms. One possibility would be to perform GI screens across strains or closely related species as a new means of examining the evolution of genetic networks.

### GIs using knockdown versus knockout screening

In contrast to knockout alleles, knockdown by CRISPRi is more similar to naturally occurring genetic variation that decreases mRNA or protein levels (such as eQTLs) or diminishes protein activity. One possibility is that GI screens using knockdown alleles will reflect GIs that could occur because of segregating genetic variation, which would be missed using knockout alleles. However, a difference between knockout and knockdown alleles is that for knockout alleles, all cells in the population will have identical levels of protein for that gene (i.e., it will be absent), whereas with knockdown alleles, there is likely heterogeneity in protein levels within a population of cells. Although work in bacteria has shown that binding of dCas9 to its target is irreversible (Jones et al. 2017), the actual level of protein knockdown in a given cell will depend on both stochastic and deterministic factors. These could include the time it takes for dCas9 to find the target locus, the efficiency of dCas9-induced transcriptional blockage, cell cycle state, division time, and degradation rate of the target mRNA and protein. Furthermore, the level of knockdown for a given guide when expressed alone may differ from that experienced when two guides are coexpressed. For example, Du et al. (2017) showed in mammalian cell culture that the population level knockdown of an individual gene target was often lower in strains carrying two gene-targeting CRISPRi guides than in those carrying just one, although this difference varied by guide (see Supplemental Fig. S2 in Du et al. 2017). The positive bias we observed in our double-mutant fitness from the multiplicative model (Fig. 1), as well as the subtle bias observed in a bacterial screen using 289 double CRISPRi strains (Supplemental Fig. S7 in Peters et al. 2016), could potentially be explained by a similar mechanism, wherein dCas9 is limiting when multiple gRNAs are expressed, so knockdown of each becomes less efficacious. With regard to the effect of CRISPRi on protein levels, here, we report that after a 4-h induction, ablation of target protein in single mutants, at the single-cell level, was incomplete. Future work will be required to further understand the intricacies of double CRISPRi target knockdown and how growth condition can affect efficacy.

Generally, we observed little overlap between the GIs we measured here with those that were measured using deletion, temperature-sensitive, and dAMP alleles (Costanzo et al. 2016). In addition to the differences between knockout and knockdown screening, possible explanations for the poor overlap include measurement noise, differences in growth conditions (Musso et al. 2008), and differences in the fitness measurement scheme. Indeed, in our validation assays, one-third of the GIs detected in our pooled screen could not be validated on agar plates or in small cultures. Furthermore, we were unable to validate the GI between *COG8* and *CDC25* with a spot assay and using a knockout and dAMP allele, respectively, as the dAMP *CDC25* strain showed no growth defect on YP-glycerol plates (Supplemental Fig. S14). Previous work has illustrated that different mutant alleles, either temperature sensitive or dAMP, of the same gene typically have poor overlap in GI profiles (<20% of GIs overlap for 90% of mutant allele pairs studied in Xu et al. 2012). This, coupled with the observation that there is a weak correlation between mutational effect and the strength of observed GI score (Velenich and Gore 2013), likely further contributed to the poor overlap. Despite differences in specific interactions detected, it is clear that both data sets allow the construction of biologically meaningful networks. Technologies for GI screening using cutting Cas9 in *Candida albicans* are now available (Shapiro et al. 2018), so future work in *S. cerevisiae* could determine whether screens using Cas9-induced loss-of-function mutations better overlap with prior work.

### Future applications and considerations for CRISPRiSeq

Although feasible, it is currently costly to apply CRISPRiSeq to screen for all pairwise interactions in the *S. cerevisiae* genome. However, our data, and the work of others (Guénolé et al. 2013; Martin et al. 2015), suggest that conditional screens between even subsets of the genome will reveal novel functional information. Indeed, in the case of our 760 starting pool guides, we detected network clusters enriched in function using measurements for GIs across just 20 query guides in five growth conditions. Furthermore, we showed that annotation accuracy is increased by screening in multiple conditions. Future work is needed to determine the most effective method by which GI data from multiple conditions should be combined: Our simple approach of concatenating the data across conditions was effective for five conditions but is likely not optimal because most gene pairs do not genetically interact in a given condition and thus introduce measurement noise. A new method has been recently developed to aid in the selection of an informative subset of the genome to include in GI screening (Deshpande et al. 2017), although more work is needed to determine the most informative set of experimental conditions.

With regards to designing new guide target sequences for future studies, we note that our understanding of guide design for CRISPRi is still nascent. For our experiments, 14 of the 18 query guide sequences we initially tested did not induce a fitness defect. However, our GFP-based validation assay showed that four of these actually did knock down the target protein (Supplemental Fig. S15). Although the functional validation method we present may be too low throughput for large gRNA libraries, it may be possible to generate and examine the knockdown efficacy of large libraries of barcoded CRISPRi/GFP strains in parallel using a combination of FACS and Bar-seq, for example, FlowSeq (Kosuri et al. 2013).

Although this study focused on filling the gap in our understanding of GI network dynamics across conditions, open questions remain regarding how the GI network changes with genetic background and with gene knockdown level. Although several GI screens have been performed in a different yeast species, *Schizosaccharomyces pombe* (Dixon et al. 2008; Tosti et al. 2014), most screens in *S. cerevisiae* have been performed in the S288C laboratory strain. Because the CRISPRi platform is portable, GI studies across multiple strain backgrounds using this technology are now more feasible. Furthermore, by modulating the concentration of the inducing agent or by testing multiple guides per gene (Smith et al. 2016; Deaner and Alper 2017; Liu et al. 2017), it will be possible to tease apart how GI scores vary with gene knockdown level, a question with broad implications to evolutionary theory (Trindade et al. 2009; Xu et al. 2012). Finally, the strongest impact of this approach is the power we illustrated for this technology to rapidly screen for GIs across conditions; furthermore, our data suggest that conditional screens are likely to identify GIs that would be missed in a single condition.

## Methods

### Generation of query plasmids carrying DNA barcode and gRNA

Twenty-two 60-mers, each containing a different 20-nt PAM-adjacent gRNA targeting sequence, required for CRISPRi dCas9 targeting, either were derived from the published single CRISPRi strains selected above (Smith et al. 2017) or were made by oligonucleotide synthesis (IDT). Nineteen of these sequences targeted genes, and three were nontargeting controls; eight of the gene-targeting guides were functionally validated using the GFP assay described below. For newly synthesized oligonucleotides, sequences were designed using the Yeast CRISPRi webtool (Smith et al. 2016). Each 60-mer also contained 20 nt of homology on each side to the pRS416-dCas9-Mxi1 + TetR + pRPR1(TetO)-NotI-gRNA plasmid vector (Addgene 73796) (Smith et al. 2016). PCR using primers P50 and P51 (Supplemental Table S1) and the Kapa HiFi HotStart PCR kit (KAPA Biosystems) was used to add an additional 20 nt of homology with each side of the insert, and the product was then cloned into the vector as described (Smith et al. 2016). After plasmid isolation, the entire gRNA sequence, including the inducible promoter and 20-nt PAM-adjacent targeting sequence, was amplified using P95 and P96 (Supplemental Table S1; Supplemental Fig. S16) and PrimeSTAR HS DNA polymerase (Takara Clontech) to add homology with the L001 BC library vector (Jaffe et al. 2017). The L001 BC plasmid library vector was digested with NotI HF, SpeI HF, and XhoI (NEB) for 16 h; gel purified; diluted to 10 ng/µL; and stored at −20°C until cloning. NEBuilder HiFi DNA assembly master mix (NEB) was used to clone the gRNA insert sequence into the BC library vector. All cloned plasmids were purified by Qiagen miniprep, and Sanger sequencing was used to verify assembly and to identify the unique DNA barcode sequence corresponding to each query plasmid's guide sequence. All query plasmids are listed in Supplemental Table S1, with their respective 20-nt PAM-adjacent targeting sequence and DNA barcode sequence.

### GFP fusion knockdown assays

GFP collection strains (Huh et al. 2003) were PCR verified using strain-specific primer pairs flanking the expected GFP insertion site (Primer3) (Supplemental Table S1; Howson et al. 2005). CRISPRi plasmids, generated from the first cloning step of the query plasmid generation process described above, were introduced to each GFP strain by lithium acetate transformation (Gietz and Schiestl 2007), and successful transformation was selected for using the *URA3* marker. To quantify GFP levels upon CRISPRi induction, strains were grown for 4 h in 1.5 mL each of either YPD or YPD + ATc (final concentration 250 ng/µL anhydrotetracycline, Clontech). These outgrowths were inoculated at a starting cellular concentration of 2.5 × 10^7^ cells/mL from SC-URA overnight cultures. After growth, samples were diluted to approximately 1 × 10^7^ cells/mL in SC-URA and stored at 4°C until analysis. GFP levels for 10,000 cells from each sample were measured at the Stanford Shared FACs Facility using the BluFL1 channel (560 short-pass splitter and 525/50 band-pass filter) on a custom Stanford and Cytek upgraded FACScan machine. Small and large FSC value thresholds were used to filter out dead cells and particulate matter as well as aggregates of cells, respectively. For each GFP strain carrying a CRISPRi plasmid, a loess regression, modeling the dependence of fluorescence on cell size, was fit to the ATc− sample data using the gam() function from the mgcv package in R (R Core Team 2014). Residuals from this model were then calculated for the ATc− and ATc+ samples using the predict() function.

### Generation of double CRISPRi strains via pooled transformation

One pool of 760 single-gene-targeting CRISPRi strains (Sublib1) and two pools of five and 19 nontargeting control CRISPRi strains (CCP2 and CCP3) were generated (see Supplemental Methods). To generate double CRISPRi strains, 57 transformations of individual query plasmids into one or more starting pools were performed across three batches using a standard lithium acetate protocol (Gietz and Schiestl 2007). Double-gene knockdown strains were produced by transformation of gene-targeting query plasmids into Sublib1. Single-gene knockdown control strains were generated by transforming each gene-targeting query plasmid into either or both of CCP2 and CCP3 and by transforming a nontargeting query plasmid into Sublib1. Finally, transformations of the nontargeting query plasmids into either or both of CCP2 and CCP3 generated 100 control strains carrying two nontargeting guides. Note that not all possible pairwise combinations of guides were generated in these pools, allowing for subsequent quantification of PCR chimeras (see Supplemental Methods and below) (Du et al. 2017, Schlecht et al. 2017). Genomic integration of the query plasmid was achieved by inducing Gal-Cre-mediated recombination by resuspending transformed cells near saturation density in YP + galactose (2%) and rotating for 20 h at 30°C. Cells were then plated on SC-URA, and after 3 d of growth, transformants were sterilely pooled and aliquots stored in 17% glycerol at −80°C at known cell densities.

### Data analysis

Raw sequencing reads were parsed with custom Python scripts, and subsequent count data were corrected for the presence of chimeric reads (see Supplemental Methods; Schlecht et al. 2017).

#### Estimating fitness and GI scores

Estimates of fitness for each strain were made as described (see Supplemental Methods; Jaffe et al. 2017). The mean fitness across the three replicate cultures was taken, and we required all three replicate measurements to be present for downstream analysis. For single-mutant fitness estimates, averages across two, three, five, or 17 replicate strains were taken (for strains generated, see Supplemental Table S1). Except in the case of strains carrying the *SAP30*g7 query guide, GI scores were quantified for each double mutant by first fitting a linear model, using the lm() function in R, to data from all double mutants generated from the same query guide for a given condition, whereby the dependent variable was the observed double-mutant fitness and the independent variable was the respective single-mutant fitness for the gene-targeting guide derived from the starting pool. Next, residuals were calculated for each strain from the fitted line and subsequently used as the GI score. For strains carrying the *SAP30*g7 query guide, we used the multiplicative model to estimate expected double-mutant fitness (see Results). To determine significance, we calculated the standard deviation of GI scores between all guide pairs in all conditions and used a significance cut-off of an absolute *z*-score greater than two. For each gene pair with at least three replicate strains, we computed a 95% confidence interval and determined whether it overlapped with a threshold absolute *z*-score of one. We acknowledge that other aggregation methods such as a voting scheme are potentially valid, but these methods do not account for a potential false negative derived from less efficacious guides. See Supplemental Note 1 for discussion of the results from a secondary method to call significant GIs based on false-discovery rate. Supplemental Table S3 contains GI scores and significance values for all guide pairs using both analyses.

#### Network analysis

The igraph package in R was used to visualize graphs generated from GI data. The layout.kamada.kawai() function was used to draw the network layout that visualized condition-specific GIs between query guides and guides in the starting pool. For this network, a GI was called condition-specific if its *z*-score was greater than two in one condition and was less than one in all other conditions. The layout.frucherman.reingold() function was used to draw the network layout that visualized the correlation of the GI profiles of the guides in the starting pool, whereby nodes/guides were only connected by edges if the Spearman's *rho* correlation of their GI profiles passed a threshold value chosen for each network such that <0.5% of guide pairs with different (gene and biological process) targets passed. Areas enriched for guides targeting genes in the same bioprocess, defined using GO-Slim annotations, were identified visually and outlined in Figure 3D, ii. Seventeen guides were removed from the analysis because >25% of their measurements were missing because of strain dropout during the fitness assays.

#### Using GI profile similarity to classify guide targets

The cor() function in R (with parameters method = “spearman” and use = “pairwise.complete.obs”) was used to calculate correlation coefficients for GI profiles for all pairwise combinations of starting pool guides for all subsets outlined in the Results of the entire data set. To construct a ROC curve that characterized our ability to predict, based on their GI profile similarity, whether two guides targeted the same gene, for each sample the proportions of true positives and false positives were calculated for threshold values of Spearman's *rho* between −1 and 1 using a step size of 0.05. The trapz() function from the pracma package in R was subsequently used for each sample to calculate the AUC. As a supplemental analysis, we also computed precision-recall curves, and their AUC values, for each of our subsets of data, wherein the *x*-axis is the recall (or sensitivity value computed for the ROC curve) and the *y*-axis is the precision, which is the positive predictive power calculated as the fraction of total positives assigned that are true positives.

## Data access

Sequencing counts and normalized relative frequencies, fitness, and interaction score estimates for each strain in each growth condition can be found in Supplemental Table S2. The custom Python and R scripts used to parse raw sequencing data, analyze results, and render manuscript figures are available as Supplemental Code and at GitHub (https://github.com/Sherlock-Lab/CRISPRiSeq). The raw FASTQ files from three lanes of HiSeq 2500 sequencing from this study have been submitted to the NCBI BioProject database (https://www.ncbi.nlm.nih.gov/bioproject) under accession number PRJNA421372.

## Supplementary Material

Supplemental Material
