# Supplementary material for: Improved discovery of genetic interactions using CRISPRiSeq across multiple environments
Source: Genome Res. 2019 Apr;29(4):668–81. doi: 10.1101/gr.246603.118 (PMC6442382; doi:10.1101/gr.246603.118)
Supplement: Supplemental Material [file supp_29_4_668__index.html]

Improved discovery of genetic interactions using CRISPRiSeq across multiple environments — Supplemental Material 

# Improved discovery of genetic interactions using CRISPRiSeq across multiple environments

## Supplemental Material

- Supplemental\_Information\_Final.pdf
- Supplemental\_Table\_S1.xlsx
- Supplemental\_Table\_S2.xlsx
- Supplemental\_Table\_S3.xlsx
- Supplemental\_CRISPRiSeq-master.zip
