# Supplementary material for: Improved discovery of genetic interactions using CRISPRiSeq across multiple environments
Source: Genome Res. 2019 Apr;29(4):668–81. doi: 10.1101/gr.246603.118 (PMC6442382; doi:10.1101/gr.246603.118)

Supplemental Information File

Jaffe *et al.* CRISPRiSeq Manuscript

Contents:

- Supplemental Methods and References
- Supplemental Figures S1-S16
- Supplemental Table S4
- Supplemental Note 1

Supplemental Methods

Isolation and pooling of single CRISPRi strains

A total of 760 strains, with previously established anhydrotetracycline (ATc)-dependent growth defects, were cherry-picked from a published collection of ~9,000 single CRISPRi strains (Smith *et al.* 2017) using the Stinger extension of the Rotor HDA robot (Singer Instruments). All 760 strains were cultured individually for 2 days at 30°C in 1 mL of YPD using eight 96-deep-well plates, and then combined to generate a starting pool (hereafter referred to as Sublib1); aliquots of this pool were stored in 17% glycerol at -80°C. To generate additional starting pools carrying non-targeting control guides, oligonucleotides carrying homology to the integration site and 20 random nucleotides (nt), instead of 20 nt of a PAM-adjacent targeting sequence, were transformed into the same ancestral strain that was used to generate the single CRISPRi collection (Smith *et al.* 2017). DNA was isolated from 40 clones by colony lysis, and the gRNA locus was then amplified using primers P82 and P22 (Supplemental Table S1) and OneTaq 2x Master Mix with Standard Buffer (NEB). ExoSAP-IT (Thermo Fisher) was used to digest excess primers before Sanger sequencing with primer P82. The e-crisp web tool (www.e-crisp.org) was used to verify that the random 20 base sequences had no genomic target (Heigwer *et al.* 2014). These strains carrying non-targeting guides, were grown up individually and combined as described above to create two control pools (CCP2 and CCP3, consisting of 5 and 19 strains, respectively). Supplemental Table S1 lists all strains and their respective 20 base gRNA targeting sequences within each of the three generated pools (Sublib1, CCP2 and CCP3). Two of the 19 control strains in CCP3 (CC3 and CC33) were removed from subsequent analysis, as they exhibited a growth defect in a preliminary fitness assay (Supplemental Fig. S15).

Pooled growth for fitness assay

Aliquots from all double CRISPRi pooled transformations were thawed and mixed such that each double CRISPRi strain would be represented at an approximately equal starting frequency in the pool. After pooling, to recover the cells from the freezer, cells were grown at a starting density of 2 x 10^7 cells/mL for 5 hr in YPD at 30°C. This outgrowth was then used to inoculate 3 replicate cultures of both

YPD+ATc (250ng/μL) and YPEG+ATc (250ng/μL) at a starting density of 5 x 10^7 cells/mL in 5 mL each. The remaining outgrowth was split in half and either stored at -80°C in 17% glycerol for inoculating the second batch of growth conditions, or spun and then stored at -20°C with the supernatant removed for DNA extraction and barcode locus sequencing of time zero (T0).

Batch serial growth and transfer were performed as follows: the 5 mL growth cultures were rotated at 30°C for 24 hours and then 1.25 mL of the culture were transferred to the next growth cycle by first spinning at 5K rpm, then re-suspending in 5 mL fresh growth media and returning to 30°C. This 1:4 dilution factor was chosen such that the population goes through approximately 2 generations per growth cycle. The remaining culture was split in two and saved at -20°C with the supernatant removed for DNA extraction. At each transfer, cellular density was also measured using the Coulter Counter (Beckman), and the culture was observed under the microscope for contamination. A total of 7 time points were collected, and at the final time point, to detect any significant loss of the construct carried at the YBR209W guide locus, -URA colonies were quantified by plating for single colonies on YPD and then replica plating to SC-URA. See Supplemental Fig. S2 for diagram of cell growth, measurements of cell density at each transfer and measurements of percent URA- colonies at the end of the pooled fitness assay.

For the second batch of conditions, the cells saved from the outgrowth for the first batch were recovered from the freezer by growing for 5 hrs at 30°C in YPD at a starting density of 2 x 10^7 cells/mL, after which an aliquot was saved for T0 sequencing. Next, 3 replicate cultures of YPD+ATc (250ng/μL, to be grown at 37°C) and 3 replicate cultures of SC-URA+ATc (250ng/μL, to be grown at 30°C) were each inoculated at a starting density of 5 x 10^7 cells/mL in 5mL and transferred, verified and saved as described above, with a total of 4 time points collected. At the start of the second batch of conditions, an additional 3 replicates of YPD+ATc (250ng/μL) were inoculated at 2.5 x 10^7 cells/mL, and 625 μL were transferred every 48 hrs, so that the population went through approximately 3 generations per 48 hr cycle. Batch serial culture was performed in order to have sufficient cellular material at each transfer to perform amplicon sequencing and such that phases of the growth cycle other than exponential growth were captured (i.e. saturation and recovery from saturation). Sampling of multiple time points should also

decrease the noise in fitness estimates compared to only using the first and last time points for this estimate.

DNA extraction and barcode locus sequencing for fitness assay

For the first three time points for all replicates of each growth condition, as well as both T0 outgrowth samples, genomic DNA was isolated from frozen cell pellets using the YeaStar Genomic DNA Kit (Zymo Research) and yields were quantified with Qubit dsDNA HS Assay Kit (Invitrogen). To generate sequencing libraries, for each sample a total of 150 ng of DNA (~11 million genomes, ~600 genomes per strain) was split between four identical PCRs and amplified using Q5 polymerase with 22 cycles. The forward and reverse primers (Supplemental Table S1) amplified a 942 nt amplicon of the YBR209W guide locus carrying the barcode identifying the query guide and the 20 nt PAM-adjacent targeting sequence identifying the guide derived from the starting pool. Note that future screens could decrease this relatively large amplicon size by designing each starting pool strain such that it carries its own unique DNA barcode adjacent to the loxP site. Subsequent PCR of the resulting ~300 nt double barcode locus could be used to identify corresponding genetic elements (Jaffe *et al*. 2017). The PCR used here also added Illumina adaptors and a 0 to 6 nt multiplexing tag on each side to be used to identify each sample library within the sequencing data. All 4 reactions were purified on one Qiagen PCR purification column, and then purified again on E-Gel SizeSelect 2% Agarose Gels (Invitrogen). Yields were quantified by Qubit dsDNA HS Assay Kit (Invitrogen), and the 50 sample libraries were each pooled at equimolar ratios into one of three sequencing libraries, consisting of 14, 18 and 18 samples each. The Bioanalyzer High-Sensitivity DNA Assay (Agilent) was used to verify the quality before running samples on an Illumina HiSeq at a core facility which runs samples with paired-end 2x101 nt reads, aiming to generate >100x coverage per strain per time point.

Data analysis

Parsing raw sequencing data and removing chimeric reads

Raw fastq files were parsed via custom python scripts that assigned each read pair to the correct sequencing library and strain based on the multiplexing tags, barcode and 20 nt PAM-adjacent site-

directed sequence (or SDS) it carried. Up to 1 mismatch was allowed for all identifiers except the primer sequence that was used as a reference point in the read to locate the other identifiers. For the primer sequence, 1 mismatch was allowed anywhere in its sequence in addition to allowing a mismatch at the first position. An estimate of the percentage of chimeric reads was calculated for each library by plotting the observed read counts for query barcode and starting pool SDS combinations which were not present in the experimental pool, versus the expected frequency of observation based on the observed read count for each individual component, i.e. the SDS and barcode sequences (Schlecht *et al*. 2017). A linear fit was made to these data using the `lm()` function in `R`, and this model was used to subtract out an estimated proportion of chimeric reads from the observed read counts for each BC/SDS combination that did exist in the experimental pool.

Fitness Estimation

For each strain, we normalized each count by the total counts at that respective time point. We then required a threshold frequency of 5 x 10^{-6} at the first time point (~30-40 reads) and 1 x 10^{-6} at the third time point (~5-8 reads), for a fitness estimate to be made. For each time point, (*tn*), frequencies were normalized to the change in frequency for that time point of the 100 “WT” control strains by multiplying by the following factor:

$$f_{wt,t1} / f_{wt,tn}$$

and then by dividing by the frequency at the first time point. We then fit a linear model to the change in `log(normalized frequency)` over the number of generations the pool was grown, and used the `slope + 1` of its fitted line as each strain’s fitness. Because our pooled fitness assay was performed over relatively few generations (up to 6 generations in the condition with the most growth), we expect the mean fitness of the population to be relatively constant, such that a linear model is a reasonable approach to estimate fitness. An experimental design utilizing more time points might model more complex components of fitness, for example to account for any delay between gRNA induction and decrease in

protein level, with the trade-off of a higher sequencing cost. However, since we expect any such delay to be consistent for a given guide, estimates of genetic interactions should not be affected.

qPCR

Four single CRISPRi collection strains (carrying either *COG1*g1, *REB1*g6, *RET2*g4, or a non-target guide (NTG) control) were collected and transformed with the *SAP30*g7 CRISPRi plasmid or a CRISPRi control (CC) resulting in the following double CRISPRi strains: *COG1*g1-*SAP30*g7, *COG1*g1-CC, *REB1*g6-*SAP30*g7, *REB1*g6-CC, *RET2*g4-*SAP30*g7, *RET2*g4-CC, NTG-*SAP30*g7, NTG-CC. The three gene targeting guides were chosen based on strong observed positive GIs with *SAP30*g7 in rich media. One μL of an overnight culture of each double CRISPRi strain was inoculated into 99μL of YPD plus or minus ATc (250ng/μL), in triplicate. Plates were read for OD595 every 15 minutes for a total of 70 cycles, until strains reached mid- to late-log phase. RNA from each strain was immediately extracted using the EPICENTRE MaterPure RNA Purification Kit. Using the Luna Universal One-Step RT-qPCR kit (E3005), 1 μL of RNA per reaction was amplified using primers specific to *COG1*, *RET2*, or *REB1*, and with primers targeting housekeeping gene *ALG9* (see Supplemental Table S1 for primer sequences). For each sample, the cycle time (Ct) of *ALG9* was subtracted from the Ct of the gRNA target, and these values were used to make comparisons between different conditions.

Validation of genetic interactions identified in pooled screen

Gene pair selection

We chose six gene pairs, based on one or more of the following: 1) the GI was reproducible across replicate guides, 2) multiple members of the same bioprocess or pathway exhibited the GI, 3) the GI included an uncharacterized ORF, or 4) the magnitude of the GI was among the largest detected in our screen.

Strain generation for GI validation

A total of 17 double CRISPRi strains were re-generated in a non-pooled format by transforming individual query plasmids into individually grown single CRISPRi strains using the protocol above. A total of 5 plasmids and 6 strains were used in different combinations to yield all single, double and no

gene knockdown strains required to detect genetic interactions between a total of 7 gene pairs (*BET1*/*SEC22*, *RPN5*/*LSM2*, *RPN5*/*LSM4*, *COG8*/*CDC25*, *COG8*/*CYR1*, YCR016W/*CDC25*, YCR016W/*CYR1*). Transformants were streaked for single colonies before saving, and then colony lysis, PCR and Sanger sequencing were used to verify that the expected gRNA and BC sequences were present in each saved strain.

Spot assay

Individual strains were streaked onto YPD plates from -80°C, and grown at 30°C for 2-3 days, then single colonies were used to inoculate 2mL YPD for overnight growth, rotating at 30°C. A Beckman Coulter Z2 particle counter was used to quantify saturation density, and cultures were diluted to ~ 1 x 10^8 cells/mL in 100 µL of media. Next, a series of 4 additional 1:10 serial dilutions were made for each strain. A total of 5 µL of each dilution were pipetted to two plates for each media condition to be tested: one plate with 2.5 µg ATc spread over the surface of the plate with glass beads and dried for 2-4 hrs, and one without the drug. Plate media were chosen based on the batch culture conditions in which the GI had been identified: YPD, grown at 30°C or 37°C, and YP+Glycerol (3%) grown at 30°C. Plates were inspected and imaged 1-2 times per day for up to 3 days, and no drug control plates served to verify the serial dilutions represented equal concentrations across strains.

Optical density based fitness measurement

Overnight cultures of each strain were prepared as for the spot assay. For each strain, 3 replicates each of media minus- or media plus- ATc (250ng/µL) were prepared using 2µL overnight culture and 98µL media. Media were chosen based on batch culture conditions: YPD grown at 30°C or 37°C, and YP + Ethanol (2%) + Glycerol (2%). Plates were read for OD595 every 15 minutes for a total of 90, 120 or 180 cycles, depending on the condition. Fitness was estimated for each strain by comparing the maximum doubling time for each ATc treated sample, to its untreated control. Here, expected double mutant fitness was computed as the product of the two single mutant fitness (Phillips *et al.* 2000), as not enough data were generated to compute expectation empirically.

Supplemental References:

Heigwer F, Kerr G, Boutros M. 2014. E-CRISP: fast CRISPR target site identification. *Nat Methods* **11**: 122–123.

Jaffe M, Sherlock G, Levy SF. 2017. iSeq: A New Double-Barcode Method for Detecting Dynamic Genetic Interactions in Yeast. *G3 (Bethesda)* **7**: 143–153.

Phillips P. C., Otto S. P. and Whitlock M. C. Beyond the average. The evolutionary importance of gene interactions and variability of epistatic effects. In: Wolf, J., Brodie, E. and Wade, M. (eds) *Epistasis Evol. Process*. 2000;. New York: Oxford Univ. Press, 20–38.

Schlecht U, Liu Z, Blundell JR, St Onge RP, Levy SF. 2017. A scalable double-barcode sequencing platform for characterization of dynamic protein-protein interactions. *Nat Commun* **8**: 15586.

Smith JD, Schlecht U, Xu W, Suresh S, Horecka J, Proctor MJ, Aiyar RS, Bennett RAO, Chu A, Li YF, et al. 2017. A method for high-throughput production of sequence-verified DNA libraries and strain collections. *Mol Syst Biol* **13**: 913.

Supplemental Figure S1.

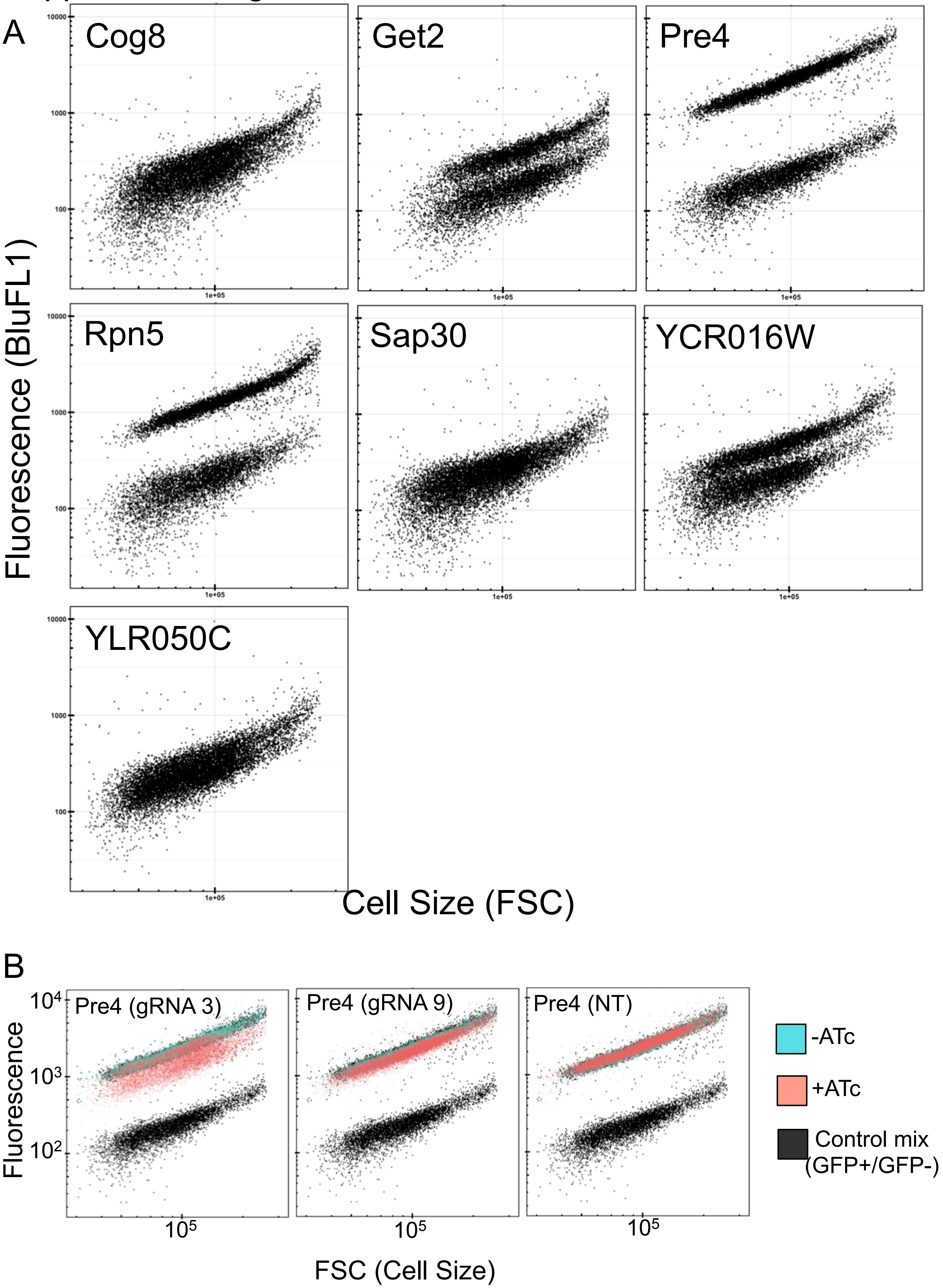

Supplemental Figure S1. Expanded results from GFP-based assay. **A)** Control mixtures (50:50) of GFP tagged strains with BY4741 illustrate distinct separation between GFP+ and GFP- cellular populations for 5 of 9 strains tested when fluorescence (BluFL1) is plotted against cell size (FSC). Name of protein tagged with GFP in the GFP+ strain is depicted on each panel.

B) Flow cytometry analysis of Pre4-GFP tagged strain carrying CRISPRi plasmids with either of two PRE4 targeting (gRNA 3 or gRNA 9) or a non-targeting control gRNA (NT). Colors indicate fluorescence in the presence (red) or absence (turquoise) of ATc (anhydrotetracycline), or of control mixture of GFP+/GFP- control strains (black). FSC is forward scatter.

Supplemental Figure S2.

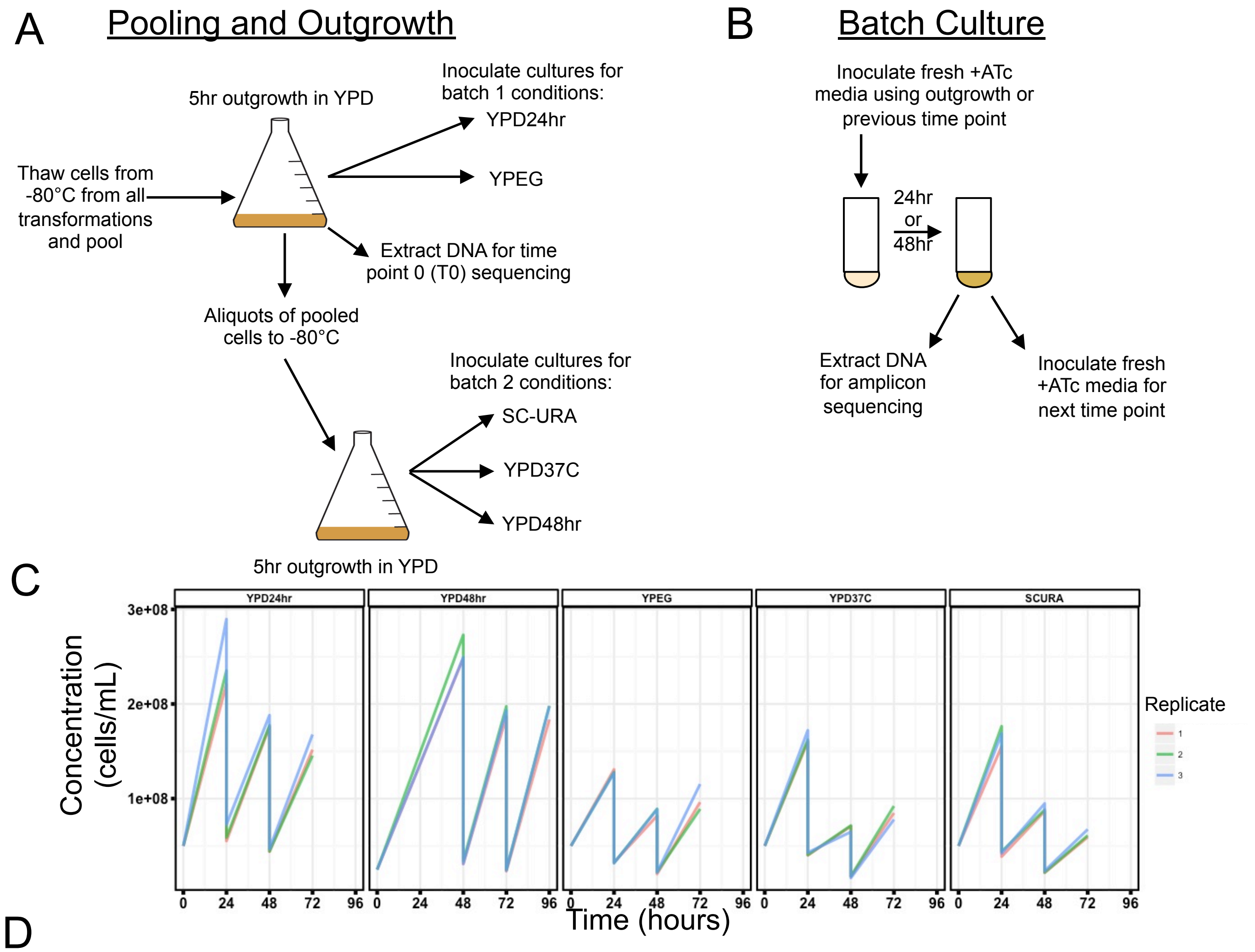

D

Condition	Replicate	Time Point Sampled	Number URA+ Colonies	Number URA- Colonies	Percent URA- Colonies
Batch1 Outgrowth	NA	0	572	9	1.55
YPD24hr	1	7	246	4	1.60
YPD24hr	2	7	207	17	7.59
YPD24hr	3	7	235	12	4.86
YPEG	1	7	170	4	2.30
YPEG	2	7	139	1	0.71
YPEG	3	7	156	1	0.64
Batch2 Outgrowth	NA	0	319	2	0.62
SCURA	1	4	52	0	0.00
SCURA	2	4	59	0	0.00
SCURA	3	4	42	1	2.33
YPD37C	1	4	126	9	6.67
YPD37C	2	4	95	5	5.00
YPD37C	3	4	121	2	1.63
YPD48hr	1	4	213	7	3.18
YPD48hr	2	4	221	9	3.91
YPD48hr	3	4	229	6	2.55

Supplemental Figure S2.

A) Diagram depicting steps prior to pooled fitness assay, including pooling and outgrowth in rich media to recover cells from freezer. Five growth conditions were tested in two batches (see Methods for details). **B)** Diagram depicting steps taking during batch culture for pooled fitness assay. **C)** Cell concentration measurements taken during pooled fitness assay at each transfer for five growth conditions. **D)** Table documenting number of URA+ and URA- colonies counted after plating single colonies from pooled fitness assay cultures to YPD agar plates then replica plating to SC-URA. Number of transfers that the pooled fitness assay had been through before plating is recorded in the column labeled 'Time point sampled'. Percent URA- colonies were also measure before the start of the pooled fitness assay (rows labeled 'Batch1 outgrowth' and 'Batch2 outgrowth').

Supplemental Figure S3.

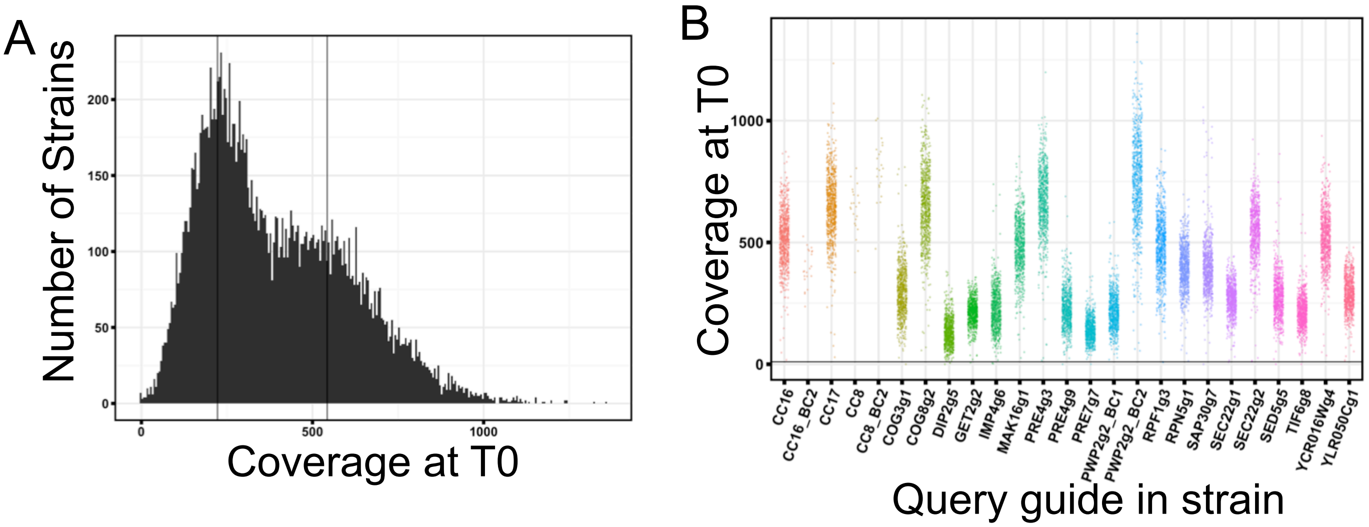

Supplemental Figure S3. Starting coverage of double CRISPRi strains. Histogram of coverage for each of 17,069 double CRISPRi strains generated for this study showed relatively even starting frequencies for each strain in the pool (**A**). T0 was collected after a 4 hr recovery in YPD from the -80°C, prior to CRISPRi induction with ATc and prior to the beginning of the pooled fitness assay. Vertical lines represent the 1st and 3rd quartiles of starting pool coverage. Variability in coverage is likely due to pipetting error during pooling. **B)** Scatter plot of the data from (A) binned by the query plasmid carried in each strain.

Supplemental Figure S4.

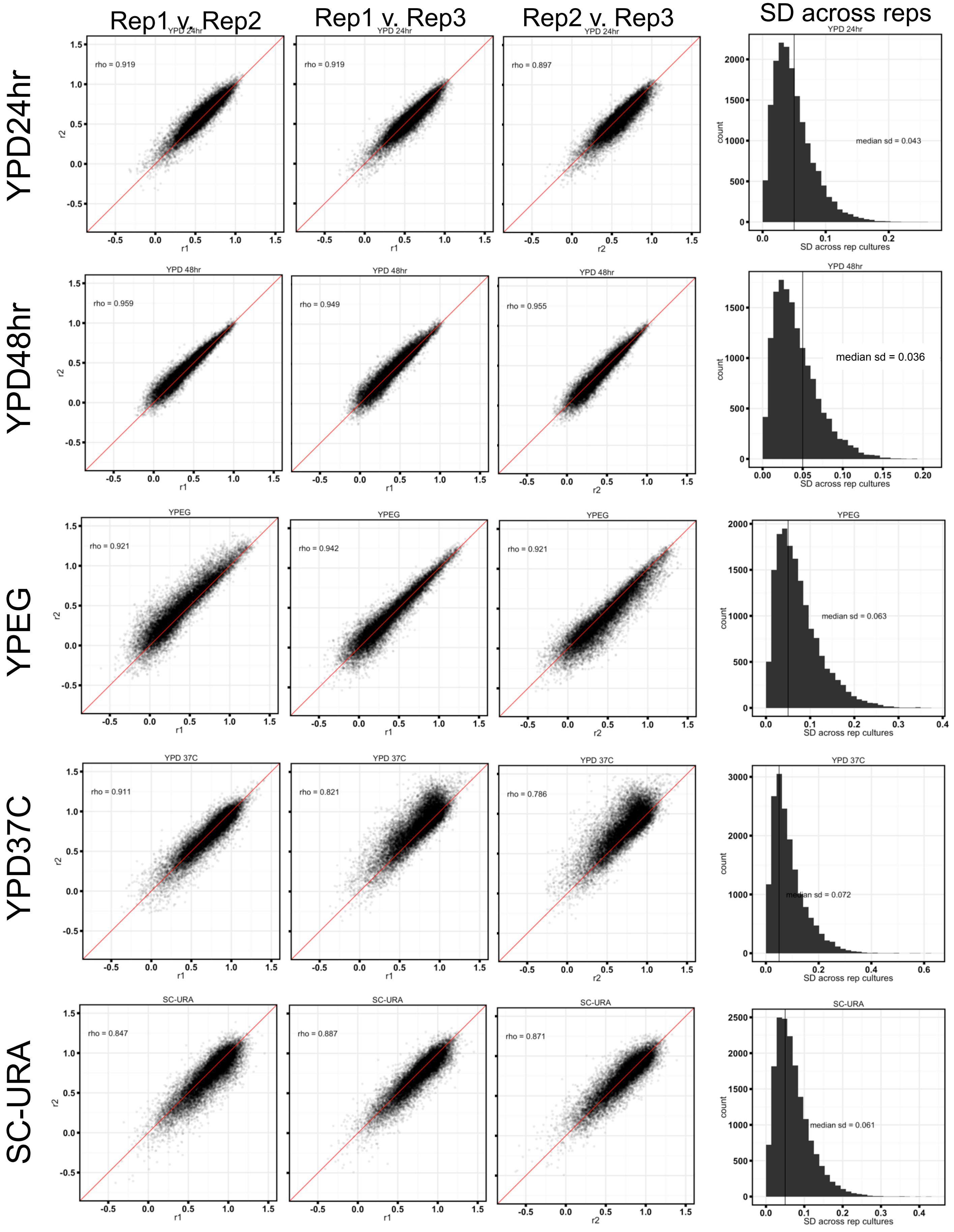

Supplemental Figure S4. Pairwise comparisons of replicate fitness measurements. For each of five conditions (rows), the first three columns are pairwise comparisons of fitness for each of ~17,000 strains in 3 replicate cultures with Spearman's *rho* depicted on plot. The fourth column is a histogram of standard deviations across measurements in replicate cultures for all strains with three replicate measurements, with the median standard deviation depicted as a vertical line. Subset of strains in YPD37C with higher fitness in Replicate 3 were present at a low starting frequency.

Supplemental Figure S5.

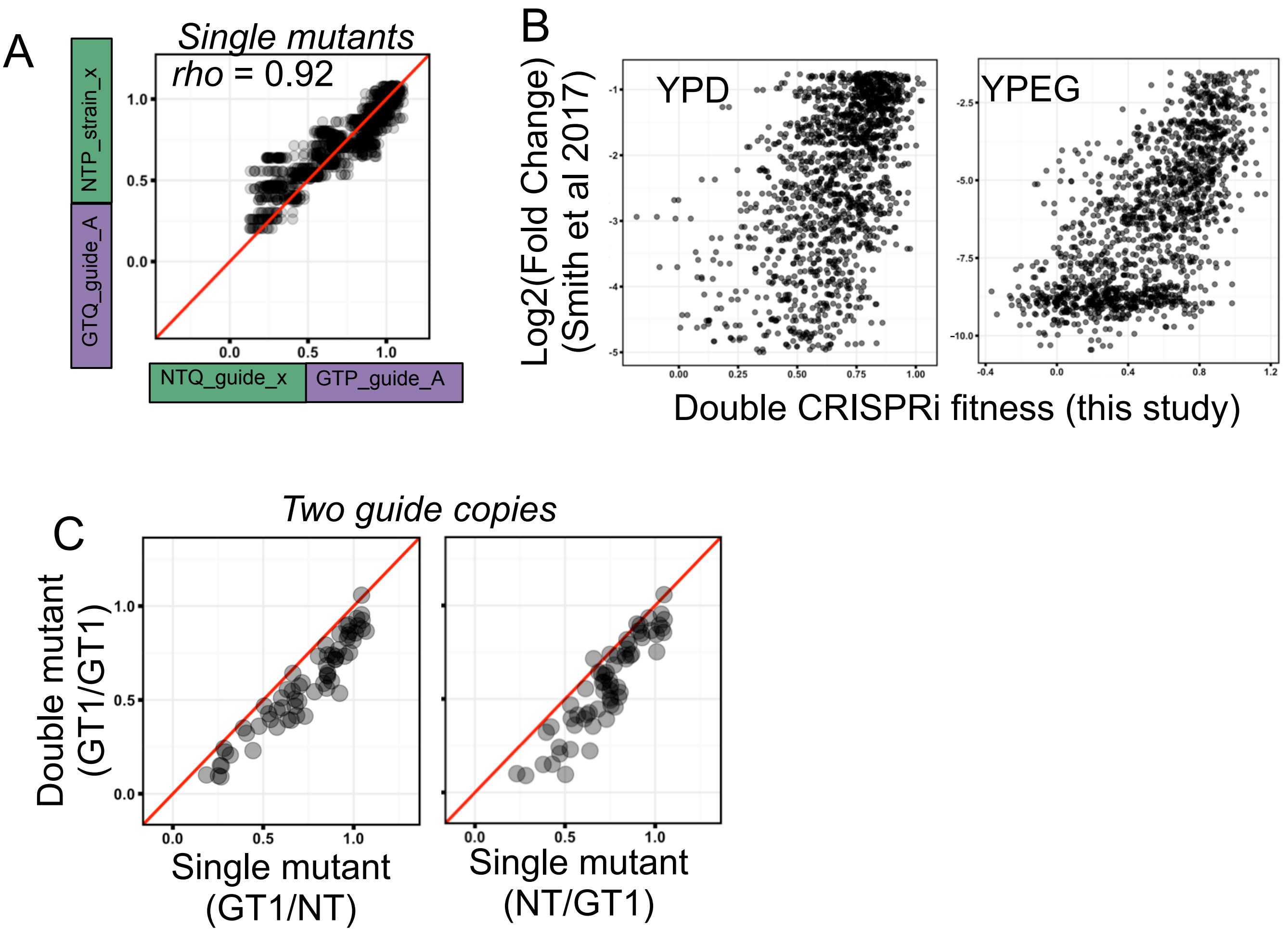

Supplemental Figure S5. Expanded reproducibility data for the pooled screen. **A)** Reproducibility of fitness across replicate single mutant strains carrying the same gene-targeting gRNA (labeled GTQ or GTP, for whether it derived from the query plasmid or starting pool), and up to 22 different non-targeting control gRNAs (labeled NTQ or NTP, for whether it derived from the query plasmid or starting pool), in reverse orientation. Pairwise comparisons of fitness in each of five conditions is depicted on same plot, and Spearman's *rho* indicates correlation value. **B)** For 760 starting pool guides, comparison of mean fitness across replicate single mutant strains (each carrying the same gene-targeting gRNA and 1 non-targeting control gRNA) is compared to log2(fold change) data (Smith *et al.* 2017) from strains carrying the same gene targeting guide and no non-targeting control guide. Data from YPD and YPEG are shown, as these were the only conditions previously tested. **C)** Comparison of mean fitness across replicate single mutant strains (each carrying the same gene-targeting gRNA and 1 non-targeting control gRNA) to fitness of double mutant strain carrying 2 of the same gene targeting gRNA. Two panels represent single mutant strains where gene-targeting guide was derived from the query plasmid (left, GT1/NT), or from the starting pool strain (right, NT/GT1). Fitness data is shown for 13 gene targeting gRNAs in 5 growth conditions. NT stands for non-targeting and GT stands for gene-targeting.

Supplemental Figure S6.

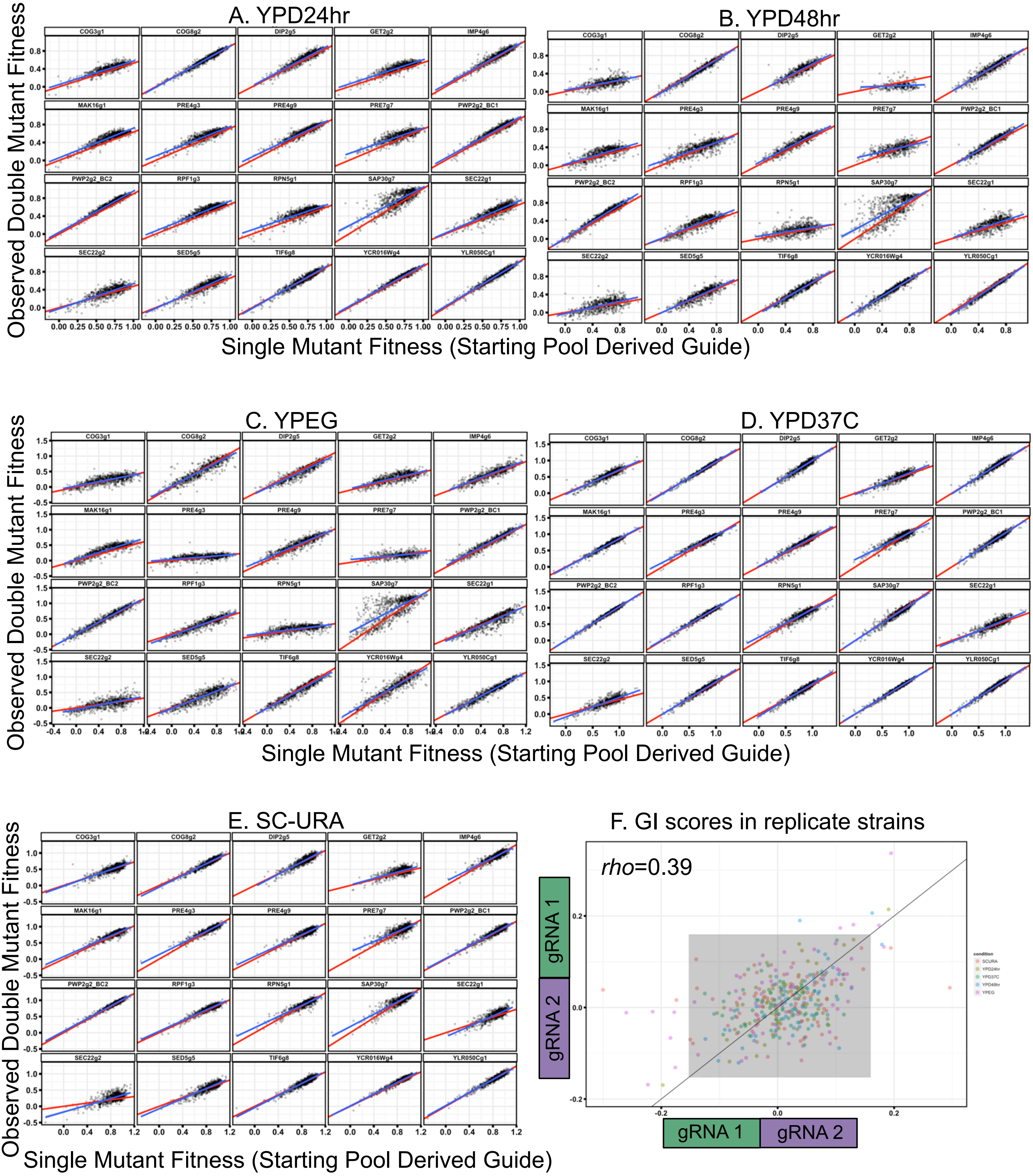

Supplemental Figure S6. Estimating GI score from fitness data. **A-E)** For each growth condition, each panel depicts data from up to 760 double mutant strains carrying a common query guide labeled at the top of the subpanel. Observed double mutant fitness is on the y-axis, and the x-axis is the mean single mutant fitness (across replicate strains) for the guide carried in the double mutant strain and derived from the starting pool strain. The red line is the expected double mutant fitness based on the multiplicative model (slope is equivalent to the single mutant fitness of the labeled query guide). The blue line is a linear regression fit, used as an empirical estimate of expected double mutant fitness, as we expect that genetic interactions between guides are rare. The empirical fit was used for expected fitness values for all cases except double mutant strains derived from *SAP30*g7, wherein the multiplicative model was used.

F) Genetic interaction scores measured in replicate strains wherein the gRNAs are in reverse orientation. Spearman's *rho* is depicted on plot. Points are colored by condition the GI score was measured in. Grey box depicts significance threshold of GI scores using absolute z-score greater than 2.

Supplemental Figure S7.

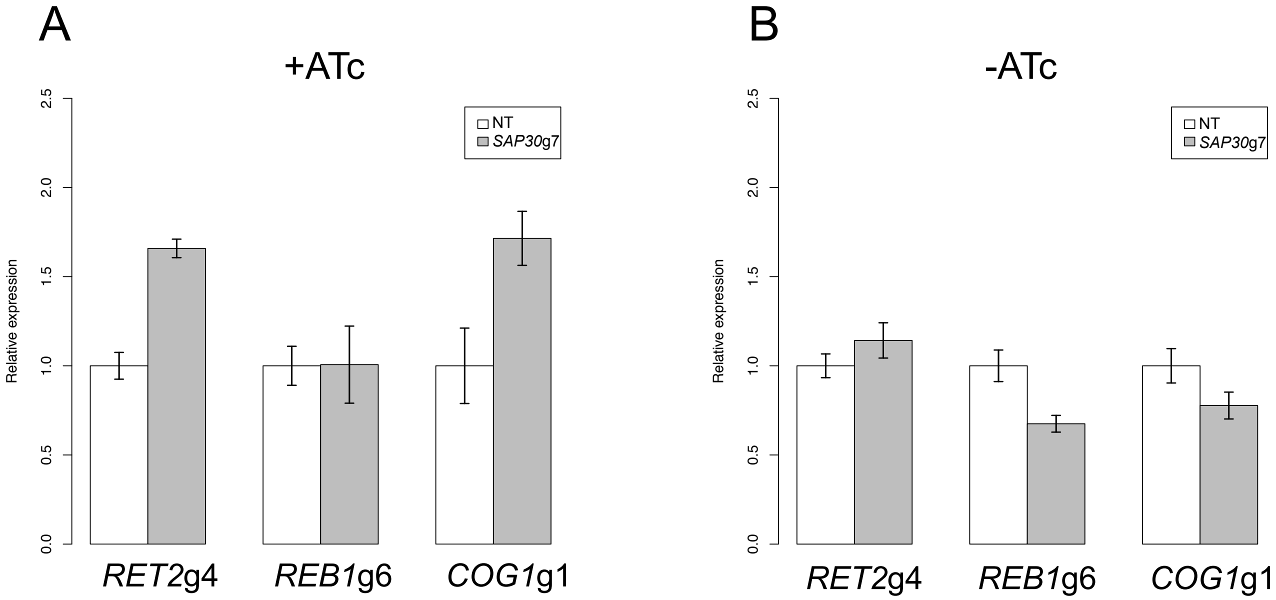

Supplemental Figure S7. Quantifying target gene expression upon *SAP30* knockdown. *RET2*, *REB1*, and *COG1* targeting gRNAs were paired with either a non-targeting control gRNA (white) or *SAP30* targeting gRNA (grey) and grown in CRISPRi inducing (**A**, +ATc) and non-inducing (**B**, -ATc) conditions. Error bars are standard error across three replicate measurements. All expression is normalized relative to the housekeeping gene *ALG9* (see Methods for details). ATc is anhydrotetracycline and NT is a non-targeting control gRNA.

Supplemental Figure S8.

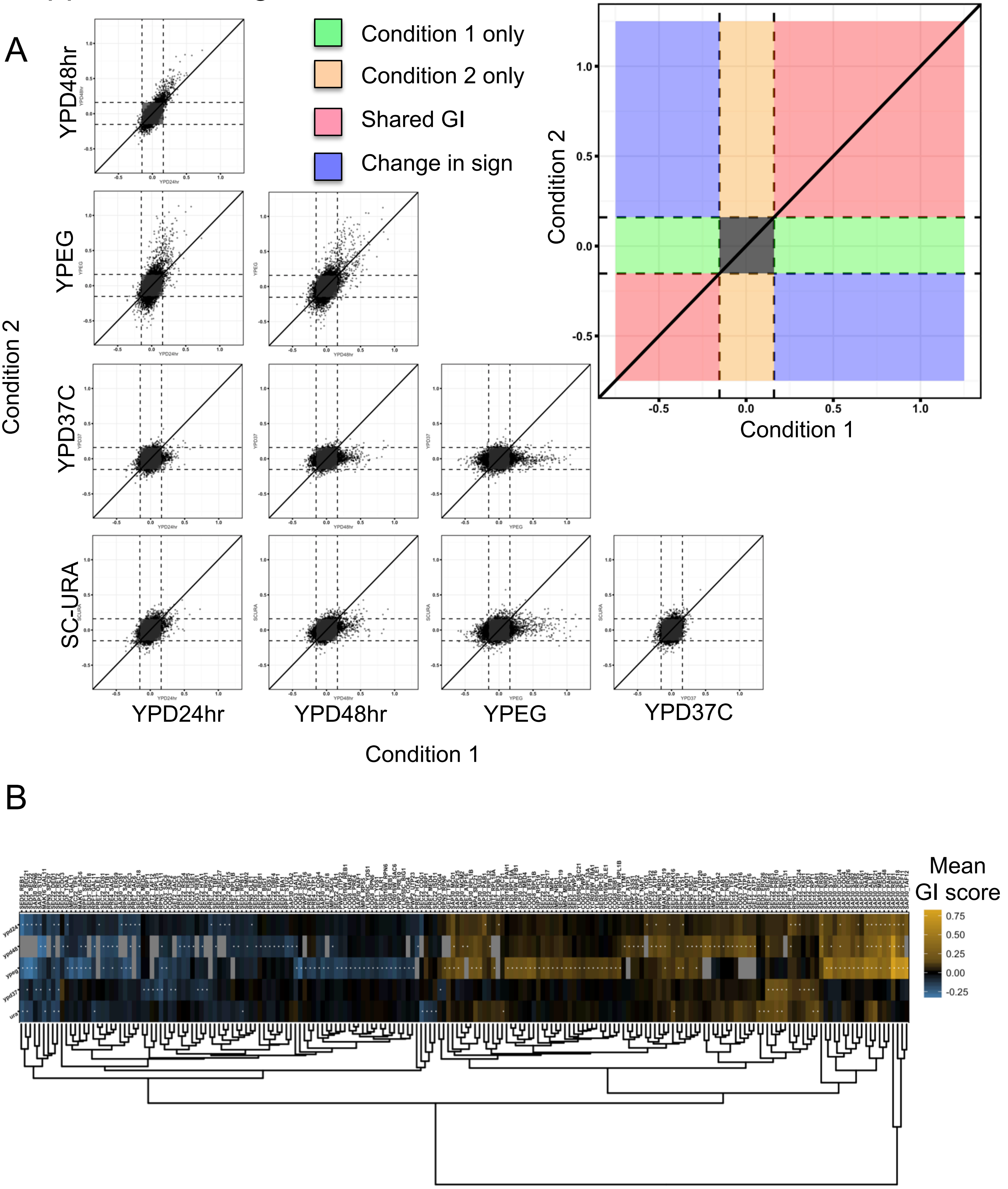

Supplemental Figure S8. GI score comparisons across conditions. **A)** Pairwise comparisons of genetic interaction scores between up to 15,200 CRISPRi guide pairs (~7,800 unique gene pairs). Horizontal and vertical dashed lines represent significant thresholds of absolute value of z-score greater than 2. Diagram in top right corner illustrates which quadrants common GIs, condition-specific GIs, and cases where sign of GI score switches. **B)** Heatmap representing mean GI score across 3 to 10 replicate strains for all gene pairs passing threshold of significance (95% CI non-overlapping with z-score of 1, denoted with *) in at least one condition. Hierarchical clustering was performed using Euclidean distance and the average clustering algorithm. These figures include all data in Fig 2E, as well as gene pairs including positive interactions with *SAP30* and gene pairs with data missing from at least one condition, which were removed from Fig 2E.

Supplemental Figure S9.

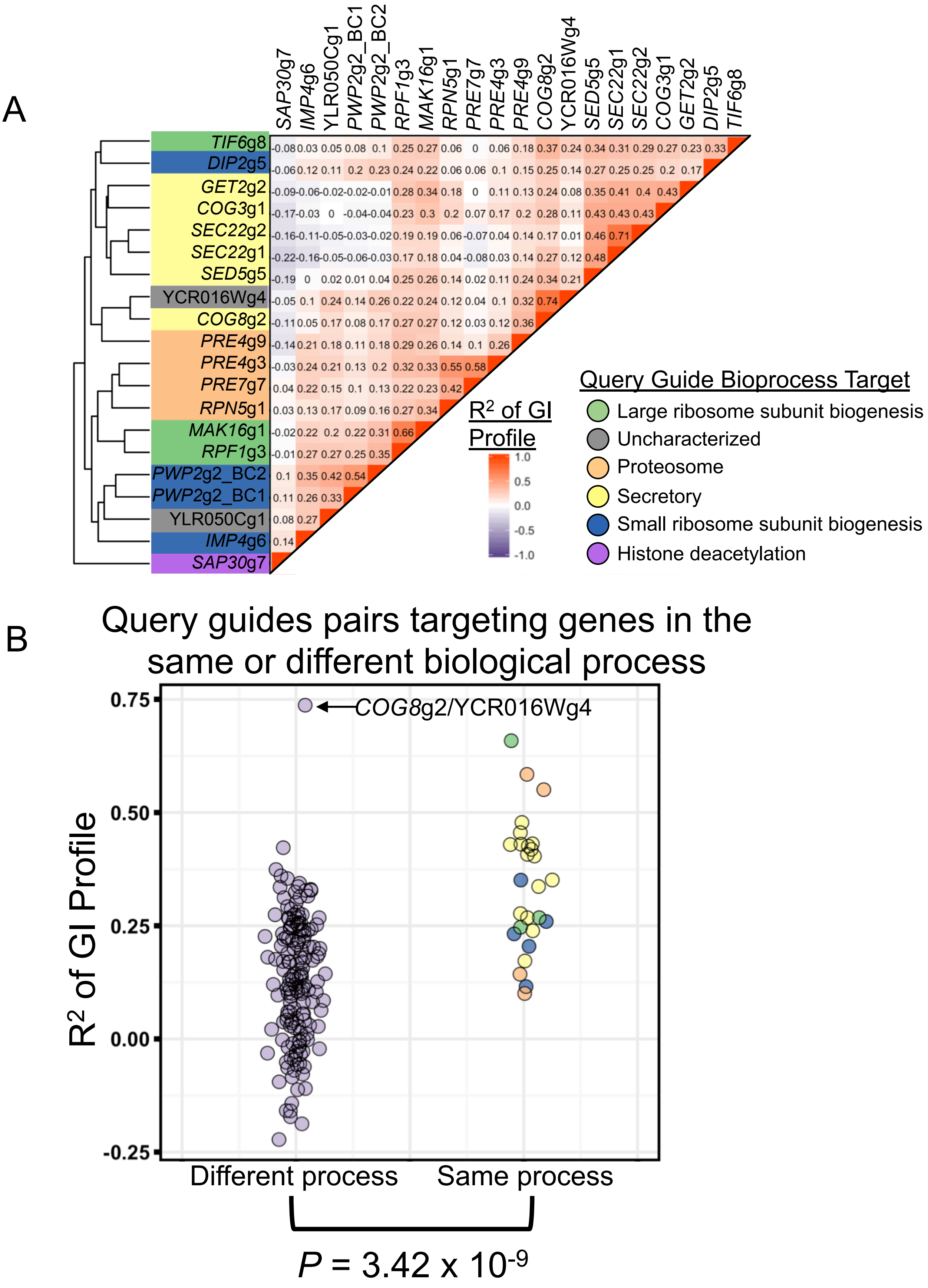

Supplemental Figure S9. GI profile similarity of query guides. **A)** Heatmap depicting similarity of GI profiles for all pairs of 20 query guides. The similarity metric is a Pearson's R^2 value, using pairwise complete observations of GI scores across 760 starting pool guides and 5 growth conditions. The dendrogram on left of heatmap depicts results of hierarchical clustering (using correlation coefficient as distance and average clustering algorithm, in R's hclust() function). The color bar on the dendrogram depicts bioprocess label of the query guide's target gene. **B)** GI profile similarity (using same metric and calculation as in (**A**) separated by whether the pair of guides target genes in the same bioprocess (right) or different processes (left, guide pairs targeting the same gene are not shown). *P*-value computed using Wilcoxon Rank-Sum test.

Supplemental Figure S10.

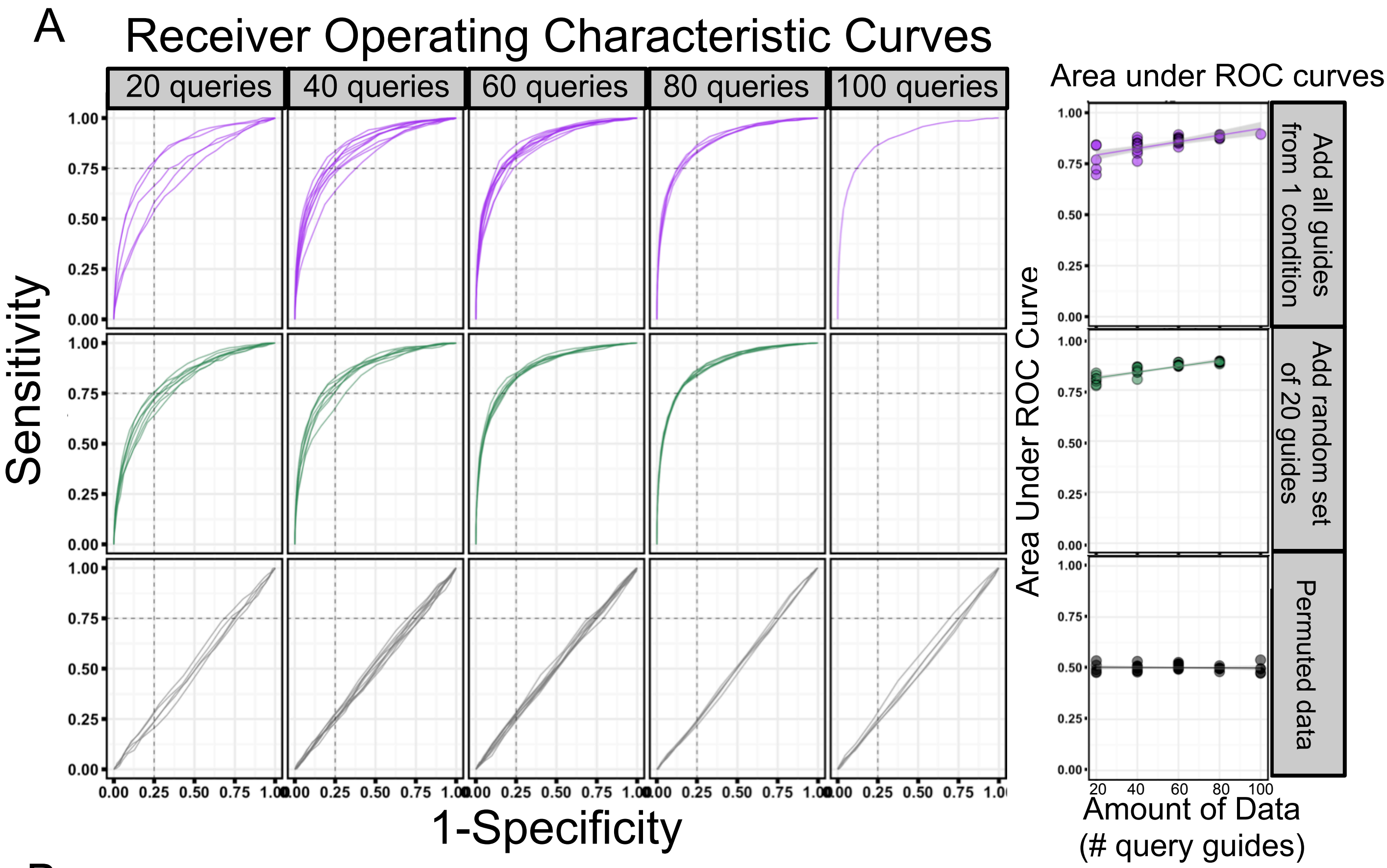

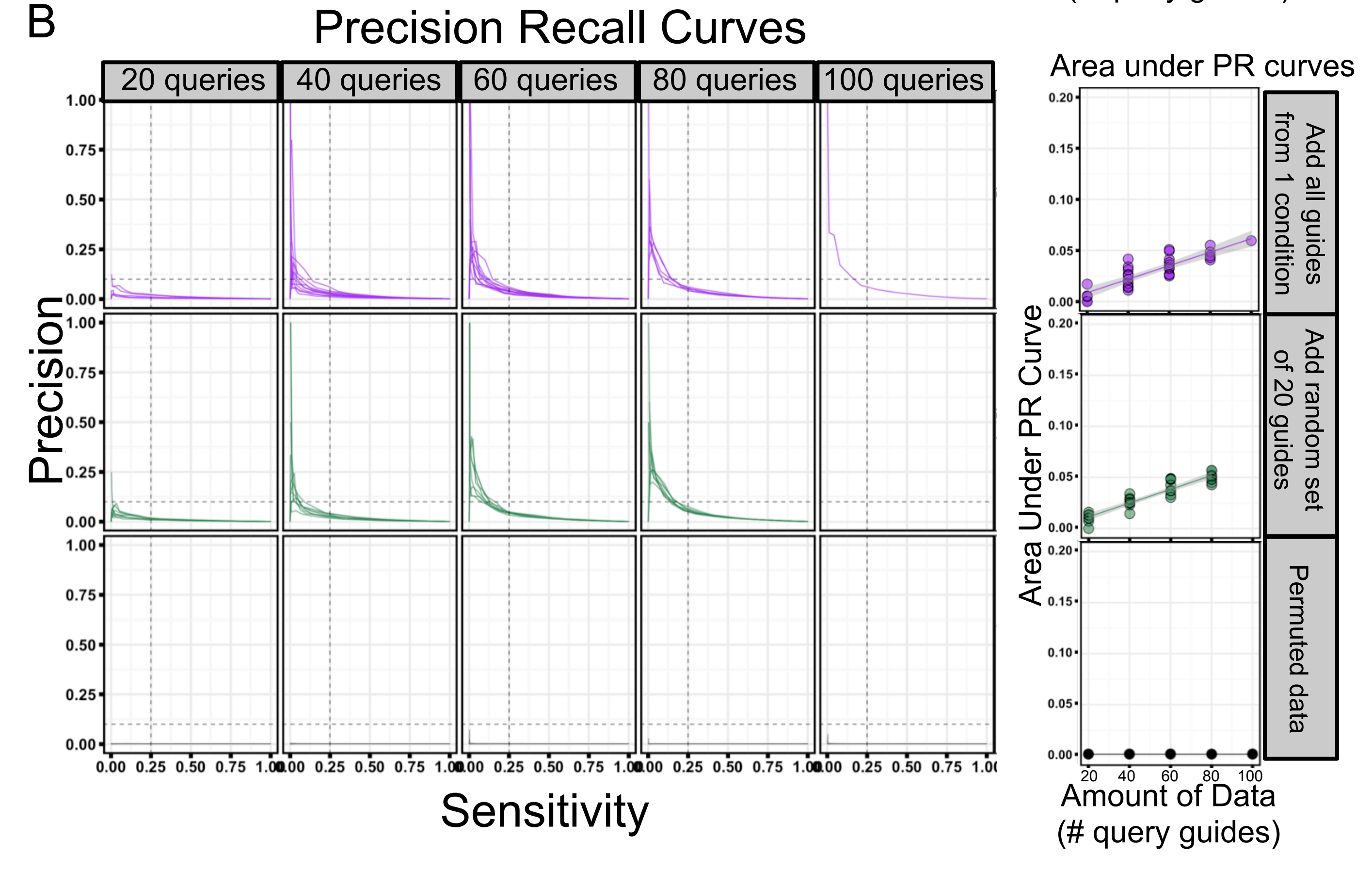

Supplemental Figure S10. Expanded results from classification analysis. **(A)** Right operator characteristic curves depicting *true positive* (Sensitivity) and *false positive* (1-Specificity) rates when classifying whether two starting pool guides target the same gene using GI profile similarity and different thresholds of Spearman's *rho*. **(B)** Precision-Recall Curves depicting *positive predictive power* (Precision) and *true positive* rate (Sensitivity or Recall) when classifying guides using the same threshold values. For (A) and (B) the analysis was performed on distributions of GI profile correlation values calculated from each possible subset of the entire dataset from 1 condition up to all 5 conditions (permuted data in black, experimental data in purple), as well as on data from 20, 40, 60, or 80 randomly selected query guides (ignoring which condition they were measured in, and performing analysis on 8 replicate samples each; data in green). The area under the curves (right) increases as a function of number of conditions used (purple) or the amount of data used (green), and this trend was absent upon permuting the data (grey).

Supplemental Figure S11.

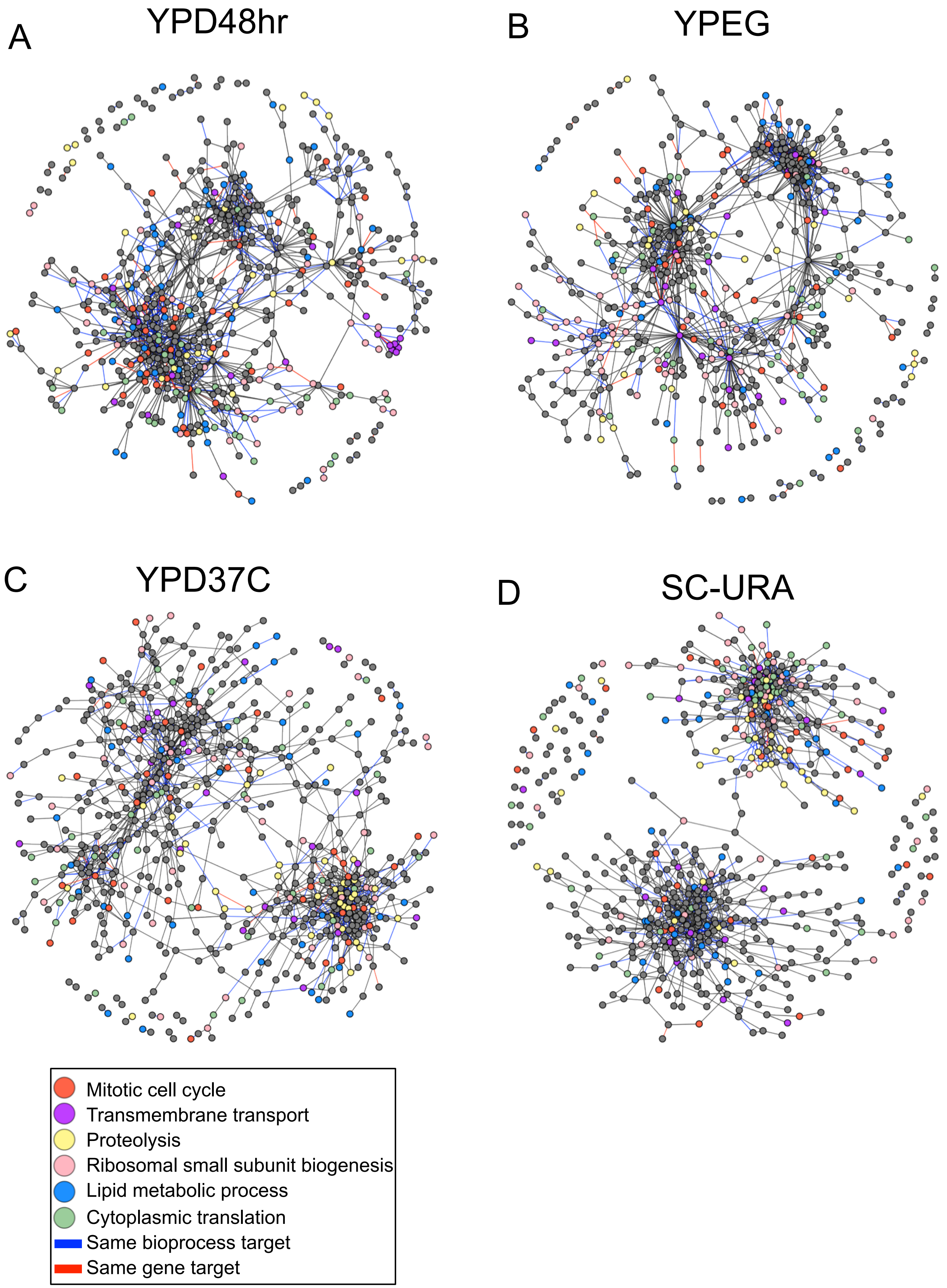

Supplemental Figure S11. Network diagrams built from data from a single condition. Nodes represent starting pool guides and are colored by bioprocess of target gene. Edges link nodes with similar GI profiles, and are colored by whether the guide pair targets the same gene (red) or same bioprocess (blue). YPD24hr is shown in Figure 3D of the main text.

Supplemental Figure S12.

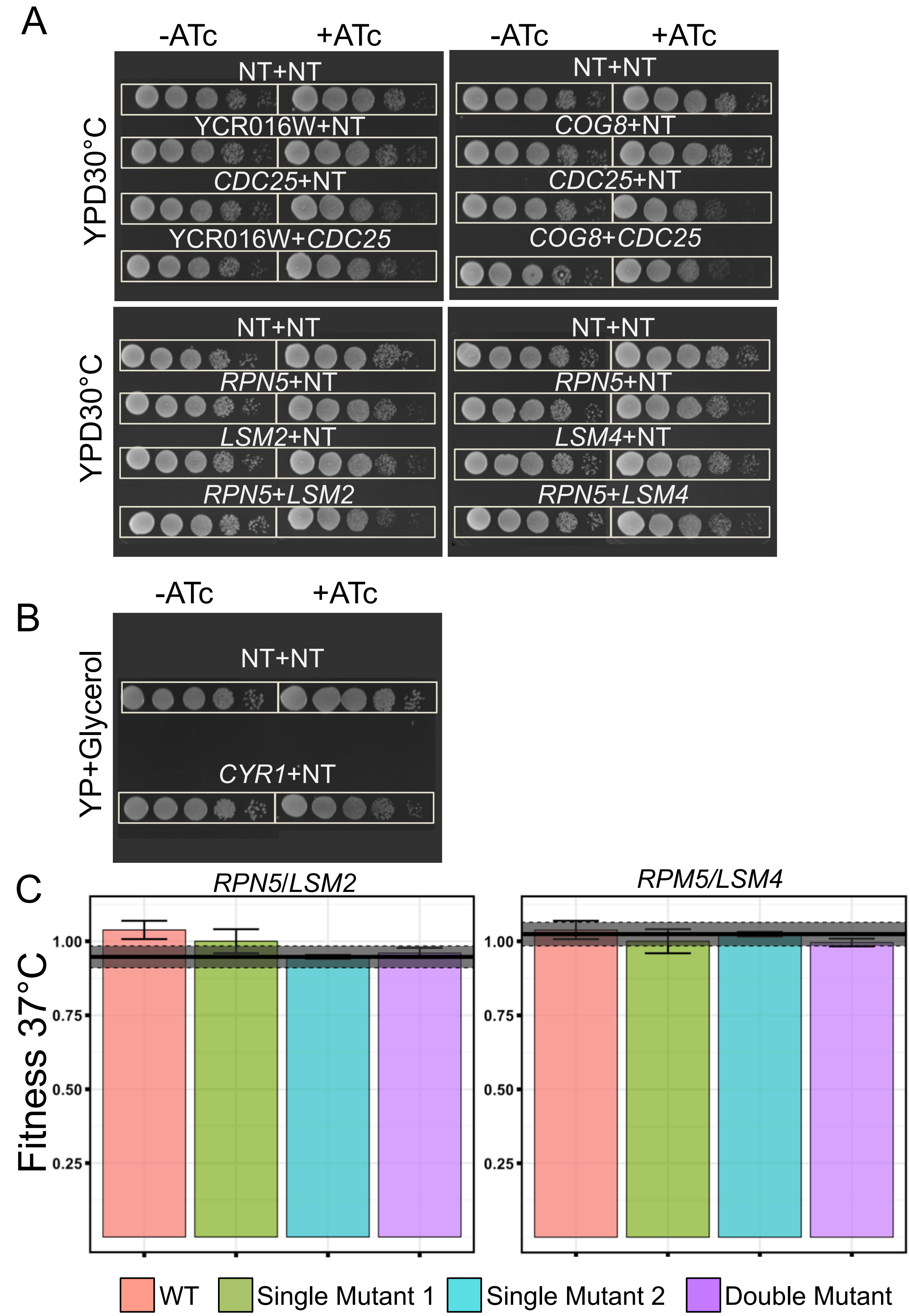

Supplemental Figure S12. Expanded results from GI validation assays. **A)** At 30°C, and in the presence of CRISPRi inducing agent ATc, no GIs are observed between *COG8* and YCR016W with *CDC25* (top), and a subtle GI is observable between *RPN5* with *LSM2* and *LSM4* (bottom). **B)** Single mutant of *CYR1* exhibits no growth defect on YP+Glycerol (3%) plates grown at 30°C. **C)** In monoculture, GIs were unobservable for *RPN5* with *LSM2* and *LSM4*. Error bars are 95% confidence intervals around mean. Horizontal line is 95% confidence interval around expected double mutant fitness (using multiplicative model). ATc is anhydrotetracycline and NT is a non-targeting control gRNA.

Supplemental Figure S13.

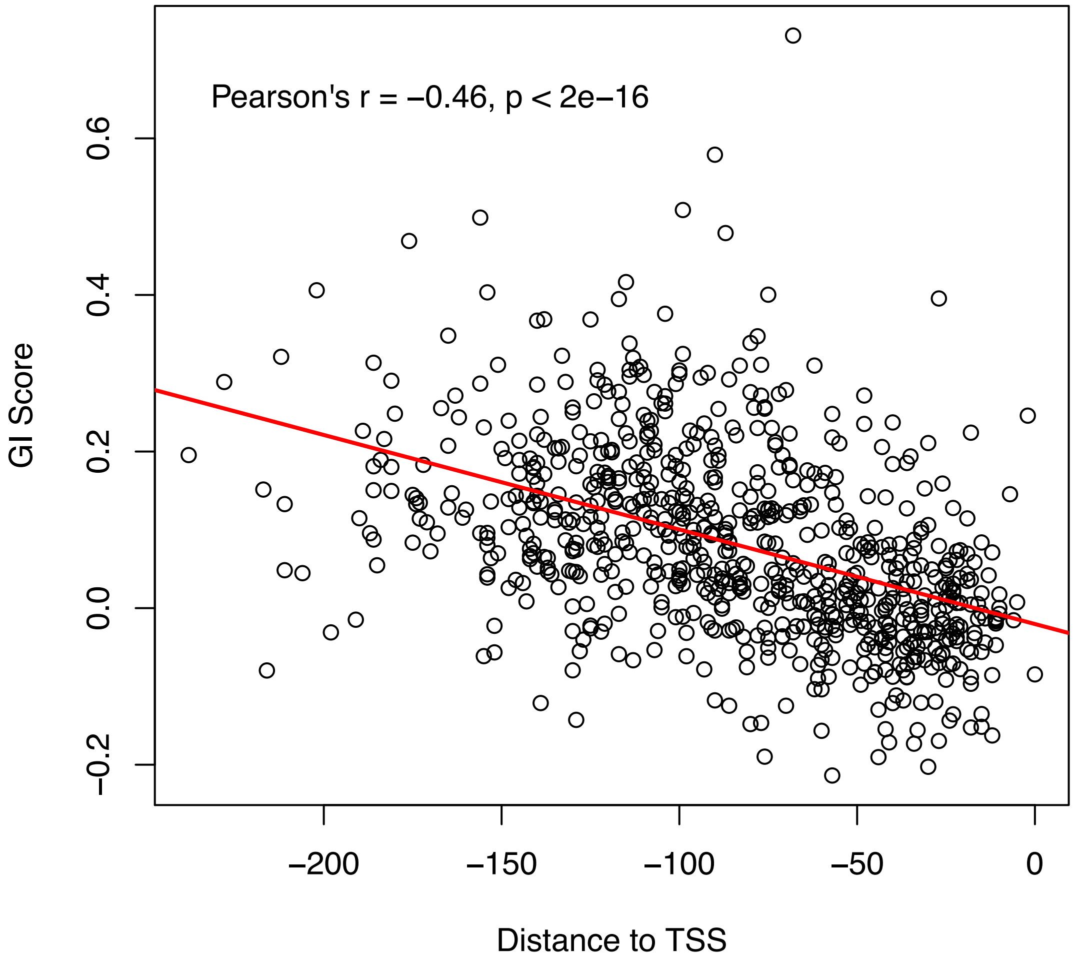

Supplemental Figure S13. GI score in rich media for each gRNA with *SAP30g7* plotted against the distance, in base pairs, for each gRNA target sequence to the transcriptional start site (TSS).

Supplemental Figure S14.

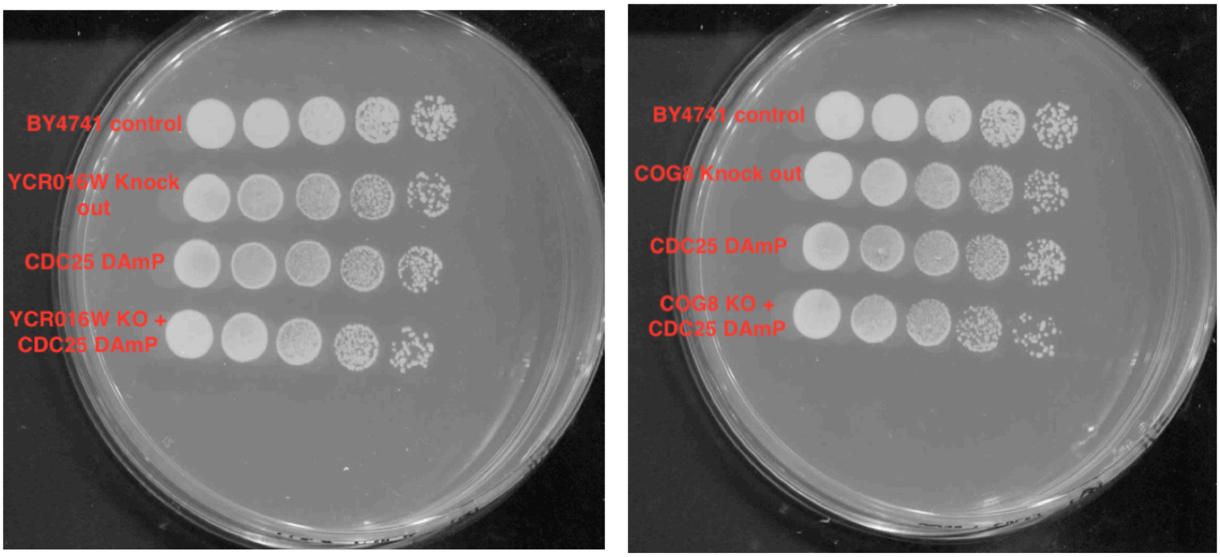

Supplemental Figure S14. Spot assay on YP+Glycerol plates. DAmP allele of *CDC25* exhibits no fitness defect.

Supplemental Figure S15.

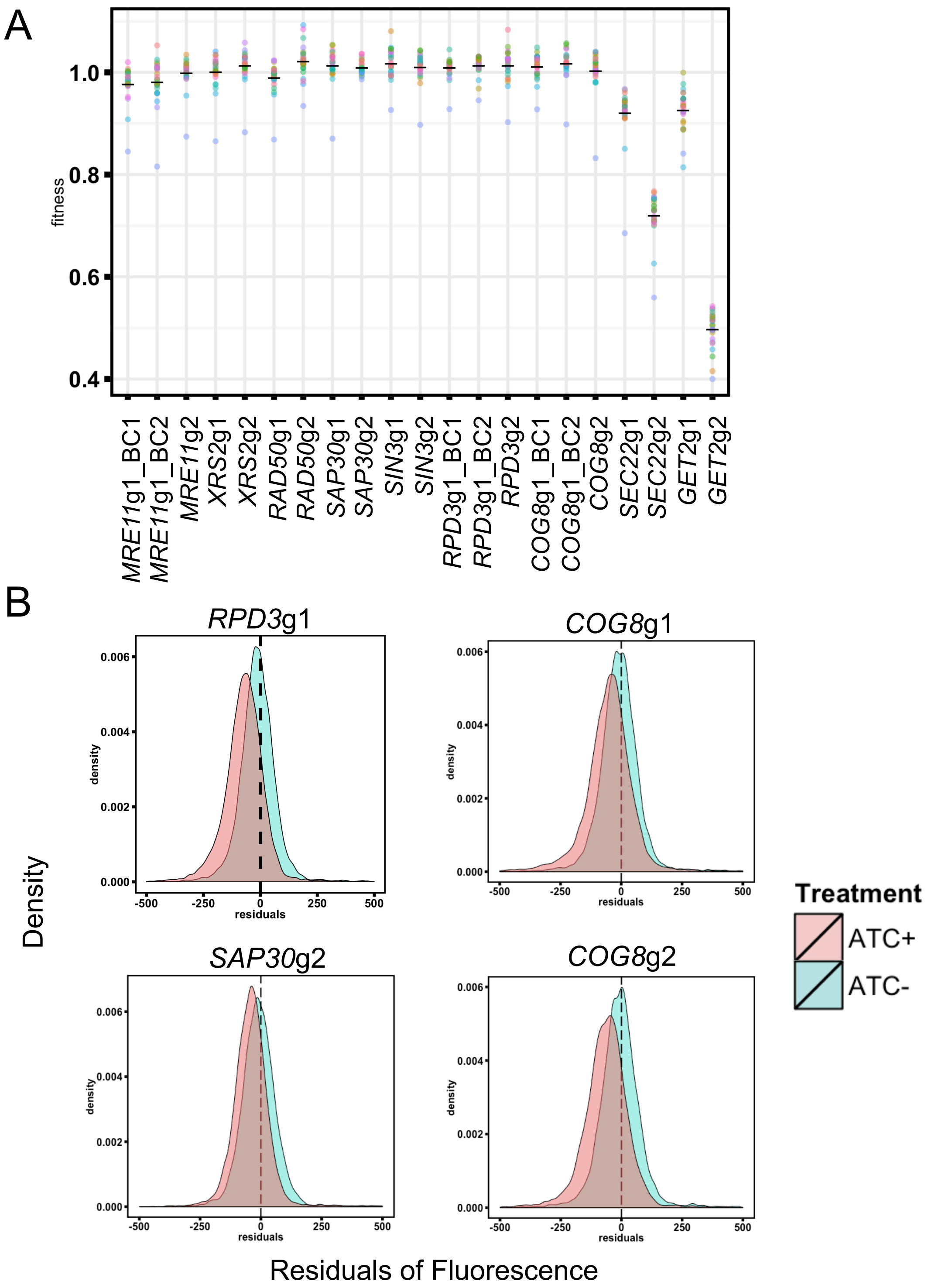

Supplemental Figure S15. A) Preliminary single mutant fitness estimates for 21 query guides paired with up to 25 non-targeting control guides in a preliminary screen. Strains carrying two of the non-targeting control guides consistently exhibit lower fitness (blue and purple dots), and so were excluded from subsequent analyses. B) GFP assay results for 4 of the query guide sequences from (A) that exhibited no fitness defect show a decrease in protein abundance upon induction of CRISPRi with anhydrotetracycline (ATC). Query guide target sequences can be found in Supplemental Table S1.

Supplemental Figure S16.

A

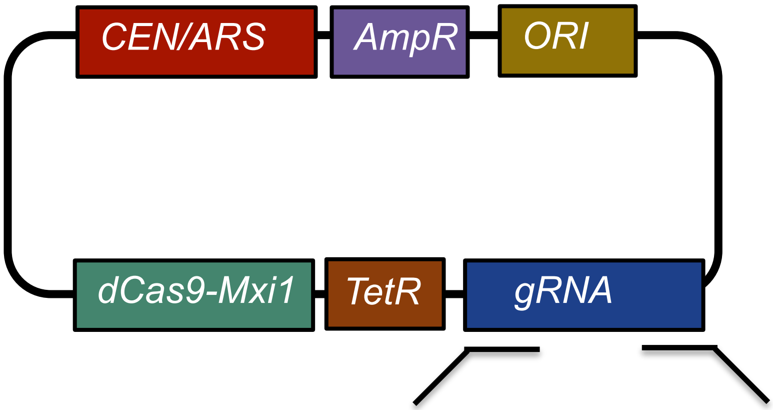

Insert:
PCR gRNA sequence from CRISPRi plasmid generated after first cloning step using P95 and P96 to add homology to vector.

B

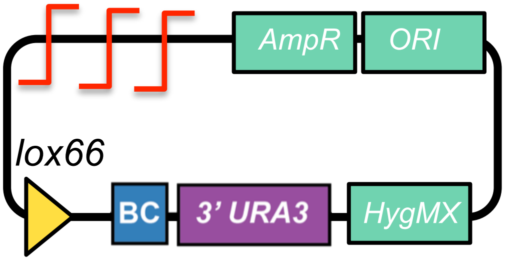

Vector:
Digest L001 with NotI, SpeI, and XhoI and then gel purify.

C

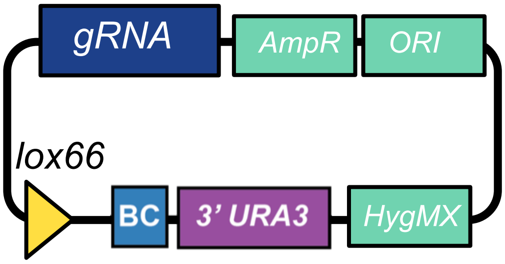

Clone insert into vector using NEBuilder (NEB), purify by Qiagen Miniprep and verify by Sanger sequencing.

Supplemental Figure S16. Cloning scheme for gRNA insertion into a barcode library plasmid.

Supplemental Table S4.

Costanzo *et al.* 2016	CRISPRiSeq (This Study): Positive	Negative	No GI	Total
Positive	18	19	144	181
Negative	55	131	547	733
No GI	276	481	3401	4158
Total	349	631	4092	5072

Supplemental Table S4. Comparison with data published in Costanzo *et al.* 2016. Prior to comparison, we excluded the following: 1) Guide pairs (in this study) exhibiting a significant positive interaction in one condition and a significant negative interaction in a second condition (n = 25 guide pairs), 2) Gene pairs including *SAP30* as one of the target genes (n = 459 gene pairs), 3) Gene pairs measured in one study but not the other, 4) Gene pairs in which a significant positive interaction that was observed in one replicate strain, and a significant negative interaction that was observed in a second replicate strain (n = 32 gene pairs). This table depicts the 5,072 gene pairs remaining (Red). Green is the number of gene pairs where findings agree between studies, Purple is number of gene pairs where the sign of GI disagrees between studies, and Grey is number of gene pairs where a significant genetic interaction was detected in one study but not the other.

Supplemental Note: Secondary analysis of GI score significance

To determine whether the increase in number of GIs observed with the number of conditions assayed was an artifact of using a z-score based significance threshold, we performed a secondary analysis that incorporated a false discovery rate. In our z-score based approach, and for each condition, we had first taken the mean fitness for each strain across replicate cultures before calculating a GI score for each double mutant strain. Here, we calculated a GI score for each double mutant strain in each replicate culture, thereby having three estimates per strain. Comparison of the GI scores computed using each of these two approaches were highly correlated (Fig. 1). For each strain, we next tested whether the three estimates from a given condition were significantly different than all estimates from that condition using a Student's t-test. We then performed the Benjamini-Hochberg false discovery rate (FDR) correction on p-values from all five conditions, and compared these adjusted p-values (also called q-values), to their values prior to adjustment (Fig. 2). We next visualized, for each condition, the number of guide pairs passing each threshold with or without an additional threshold requiring that the absolute value of the GI score be greater than 0.1 (Fig. 3). We proceeded with a q-value threshold of 0.2 (FDR of 20%), and absolute GI score greater than 0.1 (Fig. 4).

As in the main text Figure 2A, we counted the number of significant GIs in each condition, as well as the cumulative total of unique GIs across conditions (Fig. 5). In total, 8.4% of the 12,729 guide pairs tested showed a significant interaction in at least one condition. This is 43% fewer guide pairs than were detected using the z-score threshold, with the majority of the difference due to the decrease in number of significant GIs called in the YPEG condition using the FDR method (likely due to higher error on estimates in this condition). This discrepancy could also be explained in part by the thresholds we chose. Still, we observed, using this new method to call significant GIs, that the cumulative total of unique guide pairs interacting increases with number of conditions tested. Overall, there was a 1.8-fold increase in observed GIs when comparing those detected in any of the five conditions to those detected in rich media alone. To call condition-specific GIs, we required that the GI score must be less than 0.1 in all but one condition for positive interactions, and greater than -0.1 in all but one conditions for negative interactions. Similar to the z-score threshold method, we again observed conditions specific GIs in each of the 5 conditions tested. Together this analysis supports our conclusion that novel GIs are observed by assaying across multiple growth conditions.

Supplemental Note Figures:

Supplemental Note Fig. 1: Comparison of GI scores values using each approach. Pairwise comparisons of GI score estimates using two approaches in each condition (panels). X-axis is mean of 3 GI score

estimates computed separately for each replicate culture. Y-axis is GI score computed after taking the mean fitness across replicate cultures. Each point is a double mutant strain. Red line is y = x.

Supplemental Note Fig. 2: Pairwise comparison of p-values from Student's t-test (x-axis) and adjusted p-values (or q-values) from Benjamini-Hochberg's false discovery rate correction (y-axis).

Supplemental Note Fig. 3: Cumulative distributions of guide pairs passing different q-value thresholds. Left panel is number of guide pairs called as having a significant GI (y-axis) for each q-value cut-off (x-axis). Right panel is the same as the left, except an addition threshold of having an absolute GI score estimate of greater than 0.1 was required to pass significance. Each condition is represented by a different color.

Supplemental Note Fig. 4: Visualization of thresholds chosen for downstream analysis. Each panel is data from one condition, and each point is a double mutant strain. The y-axis is the absolute value of the mean GI score across replicate cultures, and the x-axis is the q-value for the estimate. Points in the blue boxes represent strains with significant GI scores using the chosen thresholds (FDR of 20% and absolute GI score of greater than 0.1). The number of significant GIs called is written on each plot.

Supplemental Note Fig. 5: Bar plot of significant GIs across conditions (to compare to Figure 2A in main text). Negative and positive GIs, as well as positive GIs with *SAP30*, are distinguished by shading. Lines indicate the cumulative total of unique interactions (circles), and condition-specific interactions (triangles).

Supplemental Note Figure 1

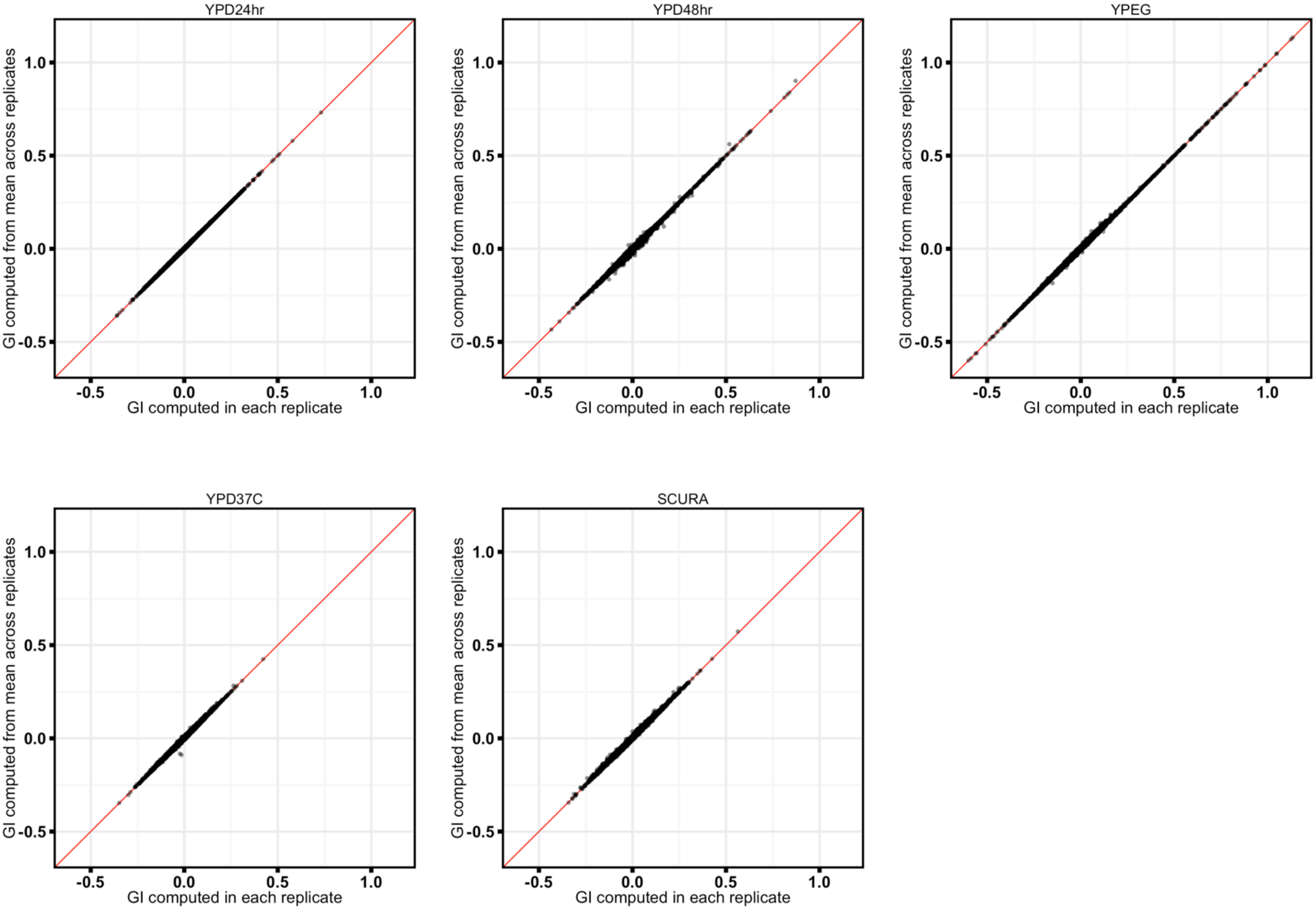

Supplemental Note Figure 2

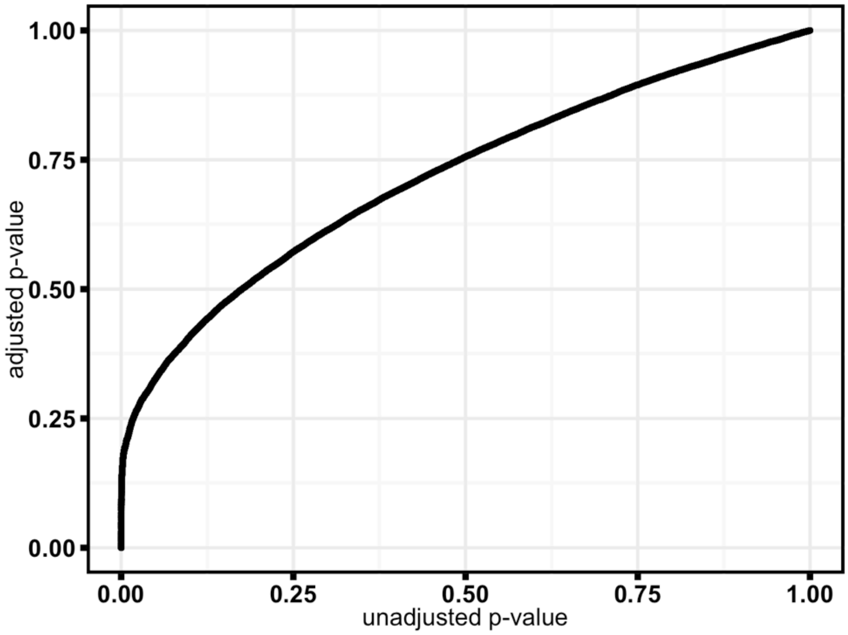

Supplemental Note Figure 3

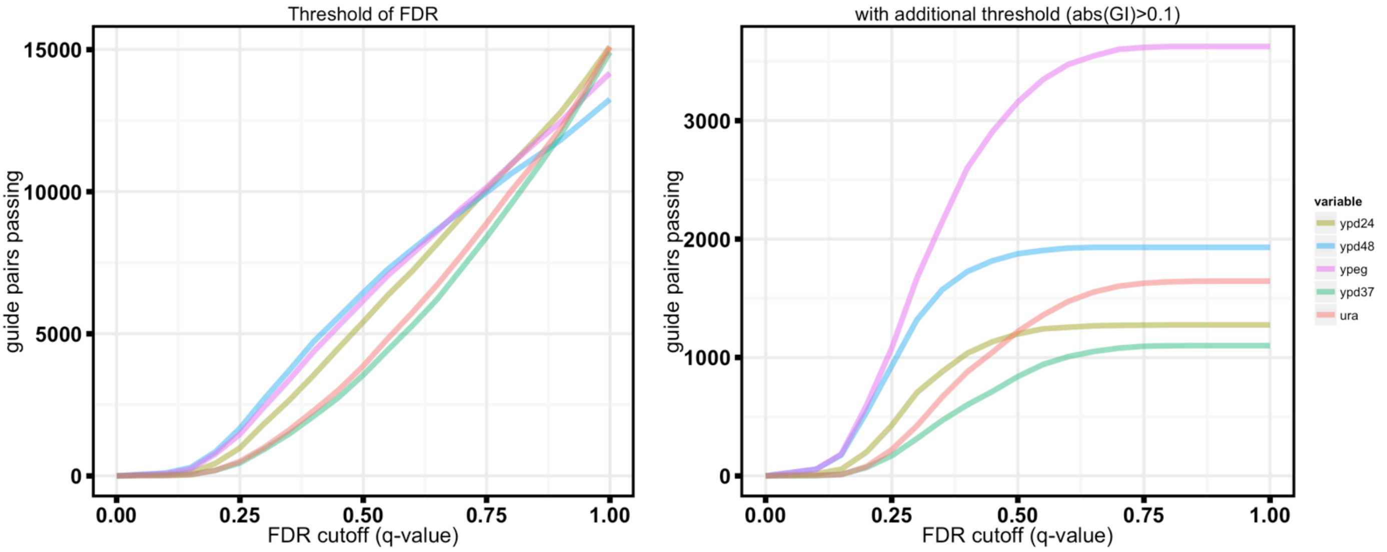

Supplemental Note Figure 4

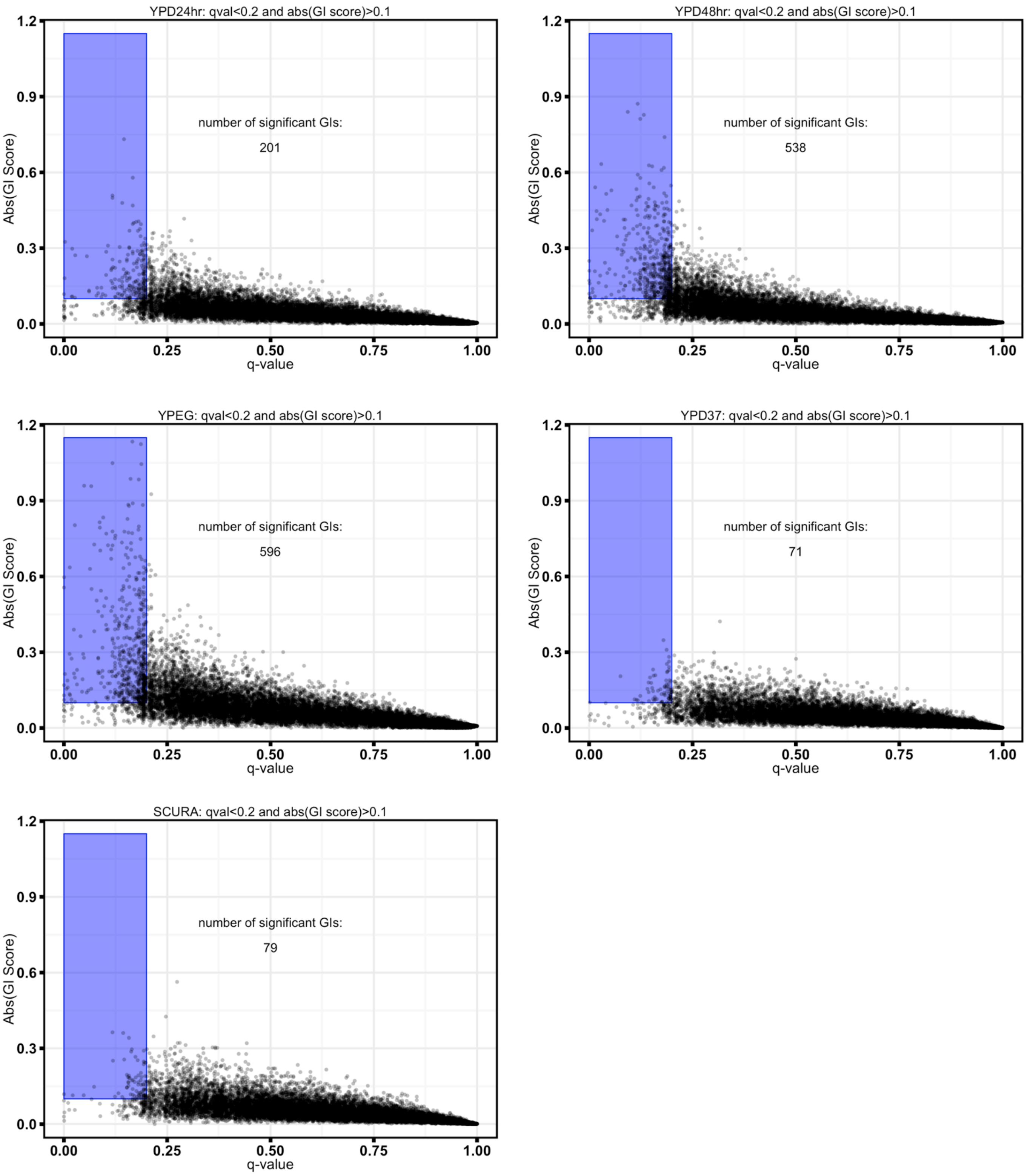

Supplemental Note Figure 5

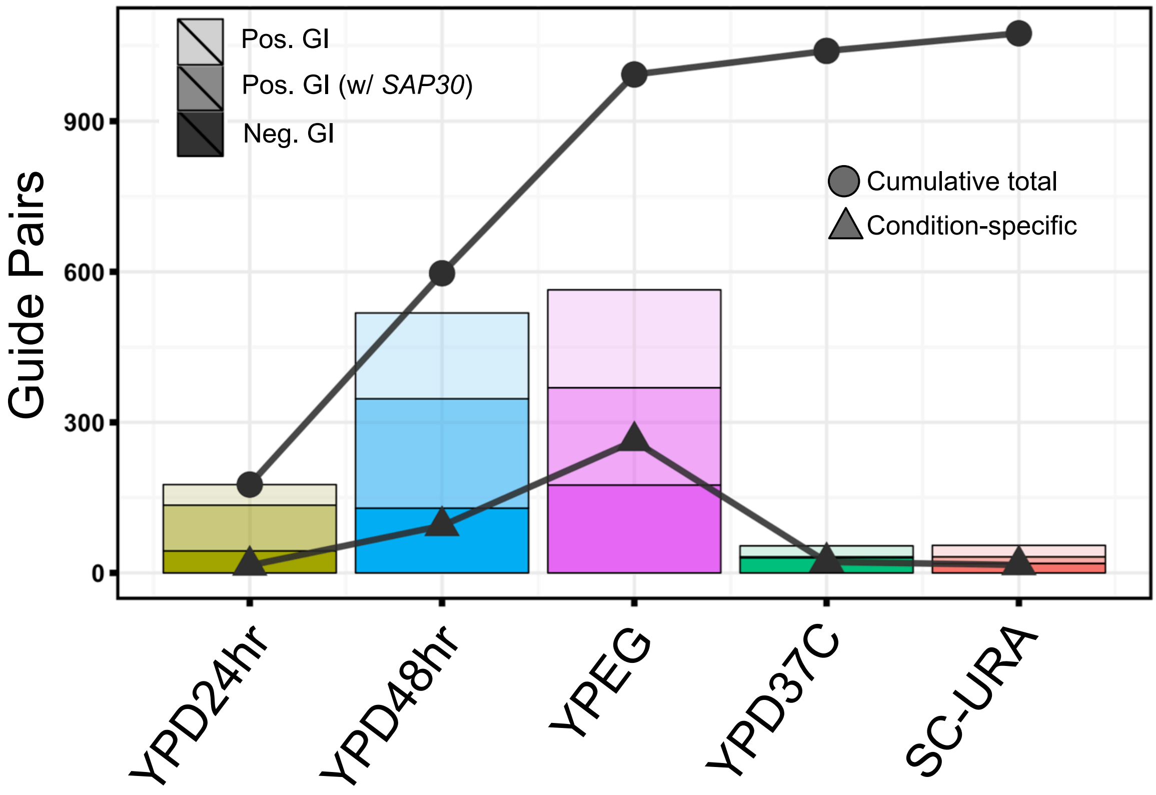

Supplement: Supplemental Material [file supp_gr.246603.118_Supplemental_Information_Final.pdf]
