# Supplementary material for: Improved discovery of genetic interactions using CRISPRiSeq across multiple environments
Source: Genome Res. 2019 Apr;29(4):668–81. doi: 10.1101/gr.246603.118 (PMC6442382; doi:10.1101/gr.246603.118)
Supplement: Supplemental Material [file supp_gr.246603.118_Supplemental_CRISPRiSeq-master.zip › CRISPRiSeq-master/visual_workflow.pdf]

Workflow for Double CRISPRi sequencing data analysis

fastq files

Seqlib11_R1.fastq
Seqlib11_R2.fastq
Seqlib12_R1.fastq
Seqlib12_R2.fastq
Seqlib13_R1.fastq
Seqlib13_R2.fastq

Process raw sequencing data

Code (for each sequencing lane run following scripts sequentially):
pull_tags_double_crispri_v2_seqlibXX.py
parse_discarded_seqlibXX.py

Count data

Two files generated per lane from python scripts:
all_counts.txt
recovered_counts.txt

Normalize count data to remove PCR chimeras

Code (separate R markdown with QC on each lanes data):
seqlibXX.Rmd

Count data (chimeras removed)

One file per lane:
seqlibXX_chimera_normalized_counts.Rdata

Also saved raw counts to RData objects:
seqlibXX_raw_counts.RData

Compute fitness and GI score estimates

Code (data from all lanes combined and analyzed in one R markdown file):
fit_gi_v4.Rmd

Fitness and GI Data:

Two files per condition, one with all information and one abbreviated:
ypd24_data.txt
ypd24_short.txt
ypd48_data.txt
ypd48_short.txt
ypeg_data.txt
ypeg_short.txt
ypd37_data.txt
ypd37_short.txt
ura_data.txt
ura_short.txt

Visualize data

Code:
One R markdown file per figure and an additional file for all supplemental figures

Manuscript Figures
