# Supplementary material for: Improved discovery of genetic interactions using CRISPRiSeq across multiple environments
Source: Genome Res. 2019 Apr;29(4):668–81. doi: 10.1101/gr.246603.118 (PMC6442382; doi:10.1101/gr.246603.118)
Supplement: Supplemental Material [file supp_gr.246603.118_Supplemental_CRISPRiSeq-master.zip › CRISPRiSeq-master/figures/figure1.html]

fig.1


# fig.1

```
require(ggplot2)
```

```
## Loading required package: ggplot2
```

```
require(reshape2)
```

```
## Loading required package: reshape2
```

```
require(RColorBrewer)
```

```
## Loading required package: RColorBrewer
```

```
theme_dbc <- theme_set(theme_gray())
theme_dbc <- theme_update(
  panel.background = element_rect(fill = "white"),
  panel.border = element_rect( colour = "black",fill=NA,size=2),
  panel.grid.major = element_line(colour = "gray93",size=1),
  panel.grid.minor = element_line(colour = "gray98",size=1),
  strip.text.x = element_text(size=12,face='bold'),
  axis.title = element_text(size=16),
  strip.background = element_rect(colour="black", fill="white",size = 1),
  axis.text = element_text(colour = "black",face="bold",size=16),
  axis.ticks=element_line(color="black",size=2))

#import fitness and calculation data
ypd24_all_dat = read.table("~/Desktop/SherlockLab2/manuscript_aug2017/code/final_fitness_GI_estimates/ypd24_data.txt",sep="\t",header=TRUE,stringsAsFactors = FALSE)
ypd48_all_dat = read.table("~/Desktop/SherlockLab2/manuscript_aug2017/code/final_fitness_GI_estimates/ypd48_data.txt",sep="\t",header=TRUE,stringsAsFactors = FALSE)
ypd37_all_dat = read.table("~/Desktop/SherlockLab2/manuscript_aug2017/code/final_fitness_GI_estimates/ypd37_data.txt",sep="\t",header=TRUE,stringsAsFactors = FALSE)
ypeg_all_dat = read.table("~/Desktop/SherlockLab2/manuscript_aug2017/code/final_fitness_GI_estimates/ypeg_data.txt",sep="\t",header=TRUE,stringsAsFactors = FALSE)
ura_all_dat = read.table("~/Desktop/SherlockLab2/manuscript_aug2017/code/final_fitness_GI_estimates/ura_data.txt",sep="\t",header=TRUE,stringsAsFactors = FALSE)

rep_array_names=c("PRE7-TRg-7","PRE4-NRg-9","PRE4-TRg-3","RPN5-NRg-1","COG3-TRg-1","SED5-TRg-5","SEC22-NRg-8","IMP4-TRg-6","DIP2-TRg-5","PWP2-NRg-2","TIF6-NRg-8","RPF1-TRg-3","MAK16-TRg-1")
rep_query_names=c("PRE7g7","PRE4g9","PRE4g3","RPN5g1","COG3g1","SED5g5","SEC22g2","IMP4g6","DIP2g5","PWP2g2_BC1","TIF6g8","RPF1g3","MAK16g1","PWP2g2_BC2")
```

Figure 1D Frequency Trajectories

```
#for ypd 24hr culture, generations at T1-T3 = 0,1.5476,3.3255
temp = ypd24_all_dat[,c(19:21,28,31:33)]
temp = melt(temp,id.vars=c("r1","strain","query","array"))
temp$time = NA
temp$time[which(temp$variable=="r1t1_nf")]=0
temp$time[which(temp$variable=="r1t2_nf")]=1.5476
temp$time[which(temp$variable=="r1t3_nf")]=3.3255

ggplot(temp,aes(x=time,y=value,color=r1))+geom_line(aes(group=strain),alpha=0.05)+
  scale_color_gradient(limits=c(-0.1,1.2),low="firebrick1",high="steelblue2")+
  scale_y_log10()+xlab("")+ylab("")+scale_x_continuous(breaks=c(0,1.5476,3.3255),labels = c(1,2,3))
```

Figure 1E Make replicate vs replicate scatter plot

```
ggplot(ypd24_all_dat,aes(x=r1,y=r2))+geom_point(alpha=0.15)+geom_abline(color="red",size=1.5)+
  xlab("")+ylab("")+scale_x_continuous(breaks=c(0,0.5,1),limits=c(-0.1,1.1))+
  scale_y_continuous(breaks=c(0,0.5,1),limits=c(-0.1,1.1))
```

```
## Warning: Removed 141 rows containing missing values (geom_point).
```

```
cor.test(ypd24_all_dat$r1,ypd24_all_dat$r2,method="spearman")
```

```
## 
##  Spearman's rank correlation rho
## 
## data:  ypd24_all_dat$r1 and ypd24_all_dat$r2
## S = 64852611024, p-value < 2.2e-16
## alternative hypothesis: true rho is not equal to 0
## sample estimates:
##       rho 
## 0.9192412
```

```
#look at mean correlation coefficient for all comparisons
mean(c(cor.test(ypd24_all_dat$r1,ypd24_all_dat$r2,method="spearman")$estimate,
       cor.test(ypd24_all_dat$r1,ypd24_all_dat$r3,method="spearman")$estimate,
       cor.test(ypd24_all_dat$r3,ypd24_all_dat$r2,method="spearman")$estimate,
       cor.test(ypd48_all_dat$r1,ypd48_all_dat$r2,method="spearman")$estimate,
       cor.test(ypd48_all_dat$r1,ypd48_all_dat$r3,method="spearman")$estimate,
       cor.test(ypd48_all_dat$r3,ypd48_all_dat$r2,method="spearman")$estimate,
       cor.test(ypeg_all_dat$r1,ypeg_all_dat$r2,method="spearman")$estimate,
       cor.test(ypeg_all_dat$r1,ypeg_all_dat$r3,method="spearman")$estimate,
       cor.test(ypeg_all_dat$r3,ypeg_all_dat$r2,method="spearman")$estimate,
       cor.test(ypd37_all_dat$r1,ypd37_all_dat$r2,method="spearman")$estimate,
       cor.test(ypd37_all_dat$r1,ypd37_all_dat$r3,method="spearman")$estimate,
       cor.test(ypd37_all_dat$r3,ypd37_all_dat$r2,method="spearman")$estimate,
       cor.test(ura_all_dat$r1,ura_all_dat$r2,method="spearman")$estimate,
       cor.test(ura_all_dat$r1,ura_all_dat$r3,method="spearman")$estimate,
       cor.test(ura_all_dat$r3,ura_all_dat$r2,method="spearman")$estimate))
```

```
## [1] 0.9003932
```

```
#print median standard deviation across replicate cultures
median(c(ypd24_all_dat$sd,
         ypd48_all_dat$sd,
         ypeg_all_dat$sd,
         ypd37_all_dat$sd,
         ura_all_dat$sd),na.rm=TRUE)
```

```
## [1] 0.05292324
```

Figure 1F Replicate strains Look at fitness of double mutants where orientation of guides is reversed

```
#make list representing all pairwise combos of guides
plot_rev_double = function(data,condition){
  all_dat = data.frame(matrix(nrow=78,ncol=6))
  names(all_dat) = c("guide1","guide2","fit1","sd1","fit2","sd2")
  
  count = 0
  for(i in 1:12){
    for(j in (i+1):13){
      count = count + 1
      all_dat$guide1[count] = rep_query_names[i]
      all_dat$guide2[count] = rep_query_names[j]
      
      rep1 = subset(data,query==rep_query_names[i]&array==rep_array_names[j])
      rep2 = subset(data,query==rep_query_names[j]&array==rep_array_names[i])
      
      all_dat[count,c(3:4)]=rep1[,35:36]
      all_dat[count,c(5:6)]=rep2[,35:36]
      }
    }
  
  all_dat$guide_label = paste(all_dat$guide1,all_dat$guide2,sep="_")
  
  return(all_dat)
}
ypd24_rev=plot_rev_double(ypd24_all_dat,"YPD24hr")
ypd48_rev=plot_rev_double(ypd48_all_dat,"YPD48hr")
ypd37_rev=plot_rev_double(ypd37_all_dat,"YPD37C")
ypeg_rev=plot_rev_double(ypeg_all_dat,"YPEG")
ura_rev=plot_rev_double(ura_all_dat,"SC-URA")

all_rev = rbind(ypd24_rev,ypd48_rev,ypd37_rev,ypeg_rev,ura_rev)
all_rev$condition = rep(c("YPD24hr","YPD48hr","YPD37C","YPEG","SC-URA"),each=78)

#update 6-21 remove color and error bars
p=ggplot(all_rev,aes(x=fit1,y=fit2))+
  geom_point(size=4,alpha=0.4,color="black")+
  geom_point(shape=1,size=4,color="black",alpha=0.6)+geom_abline(color="red",size=1.5)+
  xlab("")+ylab("")+scale_x_continuous(breaks=c(0,0.5,1),limits=c(-0.1,1.1))+
  scale_y_continuous(breaks=c(0,0.5,1),limits=c(-0.1,1.1))
p
```

```
## Warning: Removed 26 rows containing missing values (geom_point).
```

```
## Warning: Removed 26 rows containing missing values (geom_point).
```

```
cor.test(all_rev$fit1,all_rev$fit2,method="spearman")
```

```
## 
##  Spearman's rank correlation rho
## 
## data:  all_rev$fit1 and all_rev$fit2
## S = 1025774, p-value < 2.2e-16
## alternative hypothesis: true rho is not equal to 0
## sample estimates:
##       rho 
## 0.8734309
```

Figure 1G Plot mean obverved vs mean expected and distributions of GIs, all conditions on one plot

```
ggplot()+
  geom_point(aes(x=ypd24_all_dat$mean_expected,y=ypd24_all_dat$mean),
             size=0.5,color="blue",alpha=0.3)+
  geom_point(aes(x=ypd48_all_dat$mean_expected,y=ypd48_all_dat$mean),
             size=0.5,color="blue",alpha=0.3)+
  geom_point(aes(x=ypd37_all_dat$mean_expected,y=ypd37_all_dat$mean),
             size=0.5,color="blue",alpha=0.3)+
  geom_point(aes(x=ypeg_all_dat$mean_expected,y=ypeg_all_dat$mean),
             size=0.5,color="blue",alpha=0.3)+
  geom_point(aes(x=ura_all_dat$mean_expected,y=ura_all_dat$mean),
             size=0.5,color="blue",alpha=0.3)+
  geom_abline(color="black",size=1)+
  xlab("")+ylab("")+scale_x_continuous(breaks=c(0,0.5,1),limits=c(-0.2,1.2))+
  scale_y_continuous(breaks=c(0,0.5,1),limits=c(-0.2,1.2))
```

```
## Warning: Removed 1861 rows containing missing values (geom_point).
```

```
## Warning: Removed 3729 rows containing missing values (geom_point).
```

```
## Warning: Removed 2145 rows containing missing values (geom_point).
```

```
## Warning: Removed 2981 rows containing missing values (geom_point).
```

```
## Warning: Removed 1938 rows containing missing values (geom_point).
```

```
ggplot()+geom_histogram(aes(x=c(ypd24_all_dat$i_score,
                                ypd48_all_dat$i_score,
                                ypeg_all_dat$i_score,
                                ypd37_all_dat$i_score,
                                ura_all_dat$i_score)),binwidth=0.01,fill="grey19",alpha=0.6,color="black")+
  geom_vline(xintercept=0,size=2.5)+xlim(-0.2,0.3)+xlab("")+ylab("")+
  theme(axis.text.x=element_blank(),axis.text.y=element_blank())
```

Calculate mean and sd of GI scores across conditions for z-score significance calling

```
#Load in GI scores (short file)
ypd24_doubles=read.table(file="~/Desktop/SherlockLab2/manuscript_aug2017/code/final_fitness_GI_estimates/ypd24_short.txt",sep="\t",header=TRUE,stringsAsFactors = FALSE)
ypd48_doubles=read.table(file="~/Desktop/SherlockLab2/manuscript_aug2017/code/final_fitness_GI_estimates/ypd48_short.txt",sep="\t",header=TRUE,stringsAsFactors = FALSE)
ypd37_doubles=read.table(file="~/Desktop/SherlockLab2/manuscript_aug2017/code/final_fitness_GI_estimates/ypd37_short.txt",sep="\t",header=TRUE,stringsAsFactors = FALSE)
ypeg_doubles=read.table(file="~/Desktop/SherlockLab2/manuscript_aug2017/code/final_fitness_GI_estimates/ypeg_short.txt",sep="\t",header=TRUE,stringsAsFactors = FALSE)
ura_doubles=read.table(file="~/Desktop/SherlockLab2/manuscript_aug2017/code/final_fitness_GI_estimates/ura_short.txt",sep="\t",header=TRUE,stringsAsFactors = FALSE)

#record sd and mean of all gi_scores
gi_sd = sd(c(ypd24_doubles$gi_score,ypd48_doubles$gi_score,ypeg_doubles$gi_score,ypd37_doubles$gi_score,ura_doubles$gi_score),na.rm=TRUE)
#record mean of all gi_scores
gi_mean = mean(c(ypd24_doubles$gi_score,ypd48_doubles$gi_score,ypeg_doubles$gi_score,ypd37_doubles$gi_score,ura_doubles$gi_score),na.rm=TRUE)

#set thresholds for z score
zval = 2
zthresh_upper = (zval*gi_sd)+gi_mean
zthresh_lower = (-zval*gi_sd)+gi_mean
gi_sd
```

```
## [1] 0.0781284
```

```
gi_mean
```

```
## [1] 0.003516402
```

```
zthresh_upper
```

```
## [1] 0.1597732
```

```
zthresh_lower
```

```
## [1] -0.1527404
```

Figure 1H SAP30 observed versus expected double mutant fitness, all conditions on one plot

```
temp=rbind(subset(ypd24_all_dat,query=="SAP30g7"&category=="double")[,10:50],
           subset(ypd48_all_dat,query=="SAP30g7"&category=="double")[,10:50],
           subset(ypeg_all_dat,query=="SAP30g7"&category=="double")[,10:50],
           subset(ypd37_all_dat,query=="SAP30g7"&category=="double")[,10:50],
           subset(ura_all_dat,query=="SAP30g7"&category=="double")[,10:50])
temp$sig = temp$i_score > (zthresh_upper)
ggplot(temp,aes(x=mean_expected,y=mean))+
  geom_point(aes(color=sig),size=2,alpha=0.2)+
  geom_point(aes(color=sig),shape=1,size=2,alpha=0.4)+xlab("")+ylab("")+
  geom_abline(color="black",size=1)+scale_color_manual(values=c("grey19","red"))+
  theme(legend.position="none")+scale_x_continuous(breaks=c(0,0.5,1),limits=c(-0.3,1.4))+
  scale_y_continuous(breaks=c(0,0.5,1),limits=c(-0.3,1.4))
```

```
## Warning: Removed 45 rows containing missing values (geom_point).
```

```
## Warning: Removed 45 rows containing missing values (geom_point).
```

```
#print number of guide pairs passing significance for each condition
length(which(ypd24_all_dat$i_score>(zthresh_upper)&ypd24_all_dat$query=="SAP30g7"&ypd24_all_dat$category=="double"))/760*100
```

```
## [1] 24.86842
```

```
length(which(ypd48_all_dat$i_score>(zthresh_upper)&ypd48_all_dat$query=="SAP30g7"&ypd48_all_dat$category=="double"))/760*100
```

```
## [1] 35.65789
```

```
length(which(ypeg_all_dat$i_score>(zthresh_upper)&ypeg_all_dat$query=="SAP30g7"&ypeg_all_dat$category=="double"))/760*100
```

```
## [1] 41.84211
```

```
length(which(ypd37_all_dat$i_score>(zthresh_upper)&ypd37_all_dat$query=="SAP30g7"&ypd37_all_dat$category=="double"))/760*100
```

```
## [1] 1.315789
```

```
length(which(ura_all_dat$i_score>(zthresh_upper)&ura_all_dat$query=="SAP30g7"&ura_all_dat$category=="double"))/760*100
```

```
## [1] 6.315789
```
