# Supplementary material for: Improved discovery of genetic interactions using CRISPRiSeq across multiple environments
Source: Genome Res. 2019 Apr;29(4):668–81. doi: 10.1101/gr.246603.118 (PMC6442382; doi:10.1101/gr.246603.118)
Supplement: Supplemental Material [file supp_gr.246603.118_Supplemental_CRISPRiSeq-master.zip › CRISPRiSeq-master/figures/figure2.html]

fig.2


# fig.2

Load data and packages

```
require(ggplot2)
```

```
## Loading required package: ggplot2
```

```
require(reshape2)
```

```
## Loading required package: reshape2
```

```
require(RColorBrewer)
```

```
## Loading required package: RColorBrewer
```

```
require(igraph)
```

```
## Loading required package: igraph
```

```
require(scales)
```

```
## Loading required package: scales
```

```
theme_dbc <- theme_set(theme_gray())
theme_dbc <- theme_update(
  panel.background = element_rect(fill = "white"),
  panel.border = element_rect( colour = "black",fill=NA,size=2),
  panel.grid.major = element_line(colour = "gray93",size=1),
  panel.grid.minor = element_line(colour = "gray98",size=1),
  strip.text.x = element_text(size=12,face='bold'),
  axis.title = element_text(size=16),
  strip.background = element_rect(colour="black", fill="white",size = 1),
  axis.text = element_text(colour = "black",face="bold",size=16),
  axis.ticks=element_line(color="black",size=2))

#Load in corrected GI scores
ypd24_doubles=read.table(file="~/Desktop/SherlockLab2/manuscript_aug2017/code/final_fitness_GI_estimates/ypd24_short.txt",sep="\t",header=TRUE,stringsAsFactors = FALSE)
ypd48_doubles=read.table(file="~/Desktop/SherlockLab2/manuscript_aug2017/code/final_fitness_GI_estimates/ypd48_short.txt",sep="\t",header=TRUE,stringsAsFactors = FALSE)
ypd37_doubles=read.table(file="~/Desktop/SherlockLab2/manuscript_aug2017/code/final_fitness_GI_estimates/ypd37_short.txt",sep="\t",header=TRUE,stringsAsFactors = FALSE)
ypeg_doubles=read.table(file="~/Desktop/SherlockLab2/manuscript_aug2017/code/final_fitness_GI_estimates/ypeg_short.txt",sep="\t",header=TRUE,stringsAsFactors = FALSE)
ura_doubles=read.table(file="~/Desktop/SherlockLab2/manuscript_aug2017/code/final_fitness_GI_estimates/ura_short.txt",sep="\t",header=TRUE,stringsAsFactors = FALSE)

#load in array go annotations
array_go = read.table("~/Desktop/SherlockLab2/manuscript_aug2017/code/figures/GO_lists.txt",sep="\t",stringsAsFactors = FALSE,header=TRUE)

#get sd of gi_scores
temp=data.frame(ypd24_doubles$gi_score,
                ypd48_doubles$gi_score,
                ypeg_doubles$gi_score,
                ypd37_doubles$gi_score,
                ura_doubles$gi_score)
                
names(temp)=c("YPD24hr","YPD48hr","YPEG","YPD37","SCURA")

#calculate sd of interaction scores across all conditions
gi_sd = sd(melt(temp)$value,na.rm=TRUE)
```

```
## No id variables; using all as measure variables
```

```
gi_mean = mean(melt(temp)$value,na.rm=TRUE)
```

```
## No id variables; using all as measure variables
```

```
rm(temp)

#set thresholds for z score
zval = 2
zthresh_upper = (zval*gi_sd)+gi_mean
zthresh_lower = (-zval*gi_sd)+gi_mean
gi_sd
```

```
## [1] 0.0781284
```

```
gi_mean
```

```
## [1] 0.003516402
```

```
zthresh_upper
```

```
## [1] 0.1597732
```

```
zthresh_lower
```

```
## [1] -0.1527404
```

```
#record bioprocesses for query guides
proteosome = c("PRE7g7","PRE4g9","PRE4g3","RPN5g1")
secretory = c("COG3g1","SED5g5","SEC22g1","SEC22g2","COG8g2","GET2g2")
ribo = c("IMP4g6","DIP2g5","PWP2g2_BC1","PWP2g2_BC2","TIF6g8","RPF1g3","MAK16g1")
```

Make scaffold network use z score >2 in any condition use max gi score for edge weights

```
#zthresh=2

#put GI score data from each conditions into a single data frame
all_data = ypd24_doubles[,c(2,3,9)]
names(all_data)[3]="YPD24hr"
all_data$YPD48hr = ypd48_doubles[,9]
all_data$YPEG = ypeg_doubles[,9]
all_data$YPD37C = ypd37_doubles[,9]
all_data$SCURA = ura_doubles[,9]
all_data$num_pos = apply(all_data[,3:7],1,function(x) length(which(x > (zthresh_upper))))
all_data$num_neg = apply(all_data[,3:7],1,function(x) length(which(x < (zthresh_lower))))

#check how many were in each category
xtabs(~num_pos+num_neg,data=all_data)
```

```
##        num_neg
## num_pos     0     1     2     3     4     5
##       0 12892  1010   163    45    10     2
##       1   692    18     0     0     0     0
##       2   191     5     0     0     0     0
##       3   125     2     0     0     0     0
##       4    39     0     0     0     0     0
##       5     6     0     0     0     0     0
```

```
#save gene pairs where sign change occurs
dyn_sub=subset(all_data,num_pos!=0&num_neg!=0)
dim(dyn_sub)[1]#number of gene pairs with switch in sign
```

```
## [1] 25
```

```
#remove rows where change in sign occurs
sub_data = all_data[-which(all_data$num_pos>0&all_data$num_neg>0),]

#remove rows where no interaction was detected
sub_data = sub_data[-which(sub_data$num_pos==0&sub_data$num_neg==0),]

#record maximum interaction score for network drawing
sub_data$weight = apply(sub_data[,3:7],1,function(x)max(abs(x),na.rm=TRUE))


#record whether the interaction is condition specific
cond_data = subset(sub_data,num_pos==1|num_neg==1)#first subset to rows with just 1 GI
#remove rows with missing measurements and use stringent def of cond. spec (all other z scores < 1)
cond_data$num_na=apply(cond_data[,3:7],1,function(x)length(which(is.na(x))))
cond_data = cond_data[-which(cond_data$num_na!=0),1:9]
cond_data$stringent = apply(cond_data[,3:7],1,function(x)length(which(x > (gi_sd+gi_mean) | x < (-gi_sd+gi_mean))))
#cond_data$stringent_pos = apply(cond_data[,3:7],1,function(x)length(which(x > (gi_sd+gi_mean))))
#cond_data$stringent_neg = apply(cond_data[,3:7],1,function(x)length(which(x < (-gi_sd+gi_mean))))
cond_data = subset(cond_data,stringent==1)[,1:9]
#cond_data = subset(cond_data,(num_pos==1&stringent_pos==1)|(num_neg==1&stringent_neg==1))[,1:9]
sub_data$stringent = rownames(sub_data)%in%rownames(cond_data)


#format data for igraph plotting
#first data frame stores edge information
temp = sub_data[,c(1:2,8:11)]
table(temp$stringent)
```

```
## 
## FALSE  TRUE 
##  1829   454
```

```
names(temp)[1:2]=c("from","to")
temp$sign.label = "pos"
temp$sign.type = 1
temp$sign.label[which(temp$num_neg>0)]="neg"
temp$sign.type[which(temp$num_neg>0)]=2

table(temp$sign.label)
```

```
## 
##  neg  pos 
## 1230 1053
```

```
#second data frame stores node information
temp2 = data.frame(c(unique(temp$from),unique(temp$to)),
                   c(rep("query",length(unique(temp$from))),rep("array",length(unique(temp$to)))),
                   c(rep(1,length(unique(temp$from))),rep(2,length(unique(temp$to)))),
                   c(rep(5,length(unique(temp$from))),rep(2.5,length(unique(temp$to)))))
names(temp2)=c("id","guide.label","guide.type","guide.size")
temp2$names = gsub("-.+","",temp2$id)
temp2$names[which(temp2$guide.label=="array")]=NA
temp2$query_process = 6
temp2$query_process[which(temp2$id%in%proteosome)]=1
temp2$query_process[which(temp2$id%in%secretory)]=2
temp2$query_process[which(temp2$id%in%ribo)]=3
temp2$query_process[which(temp2$id=="SAP30g7")]=4
temp2$query_process[which(temp2$id%in%c("YCR016Wg4","YLR050Cg1"))]=5

#build igraph network object
net = graph.data.frame(d=temp,vertices=temp2,directed=F)

#fill in cosmetics of plot
colrs3 = c("tomato","cornflowerblue","darkseagreen","darkorchid3","gray35","white")
V(net)$color <- colrs3[V(net)$query_process]
E(net)$width=E(net)$weight*1
colrs2 <- c("goldenrod","blue","red","grey40")
E(net)$color = colrs2[E(net)$sign.type]

#calculate and save layout of network
set.seed(9099)
l <- layout.kamada.kawai(net,weights=E(net)$weight)

plot(net,vertex.label=NA, main="any",layout=l,vertex.size=V(net)$guide.size)
```

```
#plot subnetworks for YPD48h and YPEG conditions
#red edges are conditions specific
#all other detected GIS in the condition are grea
for(i in 4:5){
  #make data frame for containing edge information for igraph
  temp = sub_data[which(sub_data[,i] > zthresh_upper | sub_data[,i] < zthresh_lower),c(1,2,8,9,i,11)]
  table(temp$stringent)
  names(temp)[c(1:2,5)]=c("from","to","weight")
  temp$sign.type=4
  temp$sign.type[which(temp$stringent==TRUE)]=3
  temp$weight = abs(temp$weight)
  temp$weight[which(temp$stringent==TRUE)]=temp$weight[which(temp$stringent==TRUE)]*3
  
  #build network object and plot
  net = graph.data.frame(d=temp,vertices=temp2,directed=F)
  V(net)$color <- colrs3[V(net)$query_process]
  E(net)$width=E(net)$weight
  E(net)$color = colrs2[E(net)$sign.type]
  plot(net,main=names(sub_data)[i],layout=l,vertex.size=V(net)$guide.size,vertex.label=NA)
  
}
```

Key for network diagrams

```
ggplot()+geom_point(aes(x=1,y=1),color="tomato",size=12)+
  geom_point(aes(x=1.5,y=1),color="cornflowerblue",size=12)+
  geom_point(aes(x=2,y=1),color="darkseagreen",size=12)+
  geom_point(aes(x=2.5,y=1),color="darkorchid3",size=12)+
  geom_point(aes(x=3,y=1),color="gray35",size=12)+
  geom_point(aes(x=3.5,y=1),color="white",size=12)+xlim(0.5,4)+
  geom_point(aes(x=c(1,1.5,2,2.5,3,3.5),y=rep(1,6)),size=12,shape=1)+
  theme(panel.grid.major = element_blank(),
        panel.grid.minor = element_blank())
```

Barplot with line graphs number of guide pairs with significant gis in each condition

```
#subset all data to only guide pairs with measurements in all 5 conditions
temp2 = all_data
temp2$missing = apply(temp2[,3:7],1,function(x)length(which(is.na(x))))
temp2 = temp2[-which(temp2$missing>0),1:9]
temp2$stringent = rownames(temp2)%in%rownames(cond_data)
print(dim(temp2))
```

```
## [1] 12715    10
```

```
#record number of GIs in each condition, also note condition specific and cumulative total
int_cond = data.frame(matrix(nrow=5,ncol=8))
names(int_cond)=c("cond","neg_gi","pos_gi","pos_sap","pos_notsap","cond_spec","cumulative","both")
int_cond$cond = 1:5

count = 0
for(i in 3:7){
  count = count + 1
  int_cond$pos_gi[count]=length(which(temp2[,i] > zthresh_upper))#/dim(temp2)[1]*100
  int_cond$neg_gi[count]=length(which(temp2[,i] < zthresh_lower))#/dim(temp2)[1]*100
  sub = temp2[which(temp2[,i] > zthresh_upper),]
  int_cond$pos_sap[count] = length(which(sub$query=="SAP30g7"))#/dim(temp2)[1]*100
  int_cond$pos_notsap[count] = (dim(sub)[1]-length(which(sub$query=="SAP30g7")))#/dim(temp2)[1]*100
  
  sub2 = temp2[which(temp2[,i] > zthresh_upper | temp2[,i] < zthresh_lower),]
  if(count ==1){runtot = rownames(sub2)}
  if(count > 1){runtot = unique(c(runtot,rownames(sub2)))}
  int_cond$cumulative[count]=length(runtot)#/dim(temp2)[1]*100
  
  int_cond$cond_spec[count]=length(which(sub2$stringent==TRUE))#/dim(temp2)[1]*100
  int_cond$both[count]=dim(sub2)[1]
  
}

#make bar graph
ggplot(melt(int_cond[,-3],id.vars=c("cond","cumulative","cond_spec","both")))+
  geom_bar(aes(x=cond,y=value,fill=as.character(cond),alpha=variable),
           stat="identity",position="stack",color="black")+
  scale_fill_manual(values=c("#A3A500","#00B0F6","#E76BF3","#00BF7D","#F8766D"))+
  scale_alpha_manual(values=c(0.9,0.6,0.6))+
  geom_line(size=2,alpha=0.9,aes(x=cond,y=cumulative),color="grey19")+
  geom_point(size=8,alpha=0.9,aes(x=cond,y=cumulative),color="grey19")+
  geom_line(size=2,alpha=0.9,aes(x=cond,y=cond_spec),color="grey19")+
  geom_point(size=8,alpha=0.9,aes(x=cond,y=cond_spec),color="grey19",shape=17)+
  xlab("")+ylab("")+theme(axis.text.x=element_blank())
```

Make heatmaps for GI scores aggregated across strains carrying guides targeting the same gene pair

```
#record guide names for guide sequences contained in both query plasmids and starting pool
rep_array_names=c("PRE7-TRg-7","PRE4-NRg-9","PRE4-TRg-3","RPN5-NRg-1","COG3-TRg-1","SED5-TRg-5","SEC22-NRg-8","IMP4-TRg-6","DIP2-TRg-5","PWP2-NRg-2","TIF6-NRg-8","RPF1-TRg-3","MAK16-TRg-1")
rep_query_names=c("PRE7g7","PRE4g9","PRE4g3","RPN5g1","COG3g1","SED5g5","SEC22g2","IMP4g6","DIP2g5","PWP2g2_BC1","TIF6g8","RPF1g3","MAK16g1","PWP2g2_BC2")

#combine all GI scores into one table
temp=data.frame(ypd24_doubles$gi_score,
                ypd48_doubles$gi_score,
                ypeg_doubles$gi_score,
                ypd37_doubles$gi_score,
                ura_doubles$gi_score)
names(temp)=c("YPD24hr","YPD48hr","YPEG","YPD37","SCURA")
temp$strain = ypd24_doubles$strain
temp$query = ypd24_doubles$query
temp$array = ypd24_doubles$array
temp$query_target = gsub("g.+","",temp$query)
temp$array_target = gsub("-.+","",temp$array)
temp$gene_pair = paste(temp$query_target,temp$array_target,sep="_")

#remove rows where the gene targets are the same
temp = temp[-which(temp$query_target==temp$array_target),]

#re-label gene_pair for reverse orientation replicates
temp$gene_pair2 = temp$gene_pair
rep_gene_targets = unique(gsub("-.+","",rep_array_names))
for(i in 1:(length(rep_gene_targets)-1)){
  for(j in (i+1):length(rep_gene_targets)){
    old_label = paste(rep_gene_targets[j],rep_gene_targets[i],sep="_")#get gene pair labels to replace
    new_label = paste(rep_gene_targets[i],rep_gene_targets[j],sep="_")#get label to replace old label
    temp$gene_pair2[which(temp$gene_pair==old_label)]=new_label
  }
}
print(length(unique(temp$gene_pair)))
```

```
## [1] 7791
```

```
print(length(unique(temp$gene_pair2)))
```

```
## [1] 7725
```

```
#make new data frame to record mean and sd of GI scores across replicate strains
#require at least 3 replicate strains for this analysis
rep_dat = subset(data.frame(table(temp$gene_pair2)),Freq>2)
rep_dat = cbind(rep_dat,data.frame(matrix(nrow=dim(rep_dat)[1],ncol=15)))

names(rep_dat)=c("gene_pair","nstrains",
                 "nest.ypd24","nest.ypd48","nest.ypeg","nest.ypd37","nest.ura",
                 "mean.ypd24","mean.ypd48","mean.ypeg","mean.ypd37","mean.ura",
                 "sd.ypd24","sd.ypd48","sd.ypeg","sd.ypd37","sd.ura")

#loop through each gene pair and record gi score data from across strains and conditions
for(i in 1:dim(rep_dat)[1]){
  sub = subset(temp,gene_pair2 == rep_dat$gene_pair[i])[,1:5]
  rep_dat[i,3:7]=apply(sub,2,function(x)length(which(!is.na(x))))
  rep_dat[i,8:12]=apply(sub,2,function(x)mean(x,na.rm=TRUE))
  rep_dat[i,13:17]=apply(sub,2,function(x)sd(x,na.rm=TRUE))
  
  }
rownames(rep_dat)=rep_dat$gene_pair

#re-format for heatmap plotting
rep_dat_l = rep_dat[,c(1:3,8,13)]
names(rep_dat_l)[3:5]=c("num_est","mean","sd")
rep_dat_l$condition = "ypd24"

temp4 = rep_dat[,c(1:2,4,9,14)]
names(temp4)[3:5]=c("num_est","mean","sd")
temp4$condition = "ypd48"
rep_dat_l = rbind(rep_dat_l,temp4)

temp4 = rep_dat[,c(1:2,5,10,15)]
names(temp4)[3:5]=c("num_est","mean","sd")
temp4$condition = "ypeg"
rep_dat_l = rbind(rep_dat_l,temp4)

temp4 = rep_dat[,c(1:2,6,11,16)]
names(temp4)[3:5]=c("num_est","mean","sd")
temp4$condition = "ypd37"
rep_dat_l = rbind(rep_dat_l,temp4)

temp4 = rep_dat[,c(1:2,7,12,17)]
names(temp4)[3:5]=c("num_est","mean","sd")
temp4$condition = "ura"
rep_dat_l = rbind(rep_dat_l,temp4)

#set factor levels for conditions
rep_dat_l$condition = factor(rep_dat_l$condition,levels=c("ypd24","ypd48","ypeg","ypd37","ura"))

#set mean and sd to NA if there are fewer than 3 replicate measurments
rep_dat_l$mean[which(rep_dat_l$num_est<3)]=NA
rep_dat_l$sd[which(rep_dat_l$num_est<3)]=NA

#record whether estimates pass threshold of 95% CI across replicate strains 
#non overlapping with zscore of 1
for(i in 1:dim(rep_dat_l)[1]){
  rep_dat_l$lower.95[i]=rep_dat_l$mean[i]-(1.96*(rep_dat_l$sd[i]/sqrt(rep_dat_l$num_est[i])))
  rep_dat_l$upper.95[i]=rep_dat_l$mean[i]+(1.96*(rep_dat_l$sd[i]/sqrt(rep_dat_l$num_est[i])))
}
rep_dat_l$sig = (rep_dat_l$upper < (-gi_sd+gi_mean) | rep_dat_l$lower > (gi_sd+gi_mean))
rep_dat_l$sig2 = ""
rep_dat_l$sig2[which(rep_dat_l$sig==TRUE)]="*"

#generate list of unique gene pairs with a significant interaction in at least one condition
sig_pairs = unique(subset(rep_dat_l,sig==TRUE)$gene_pair)
print(length(sig_pairs))
```

```
## [1] 198
```

```
print(dim(rep_dat))#total gene pairs tested
```

```
## [1] 1711   17
```

```
#Make first heatmap for supplement including all gene pairs passing threshold
#first get order of guide pairs (clustered based on euclidean distant), also plot dendrogram
coldist=dist(subset(rep_dat,gene_pair%in%sig_pairs)[,8:12])
colclust = hclust(coldist,method="average")
colclust.d = as.dendrogram(colclust)
colorder=colclust$labels[colclust$order]
```

```
par(mar=c(10,4,4,2))
plot(colclust.d,edgePar=list(lwd=3))
```

```
par(mar=c(5,4,4,2))
```

```
#make heatmap
rep_dat_plot = subset(rep_dat_l,gene_pair%in%sig_pairs)
rep_dat_plot$gene_pair = factor(rep_dat_plot$gene_pair, levels = colorder)
print(qplot(x=condition, y=gene_pair, data=rep_dat_plot,
            fill=mean,geom="tile")+
        scale_fill_gradientn(colours=c("steelblue","black","goldenrod"),
                             values=rescale(c(-0.33,zthresh_lower,0,zthresh_upper,0.82)),
                             guide="colorbar",limits=c(-0.33,0.82))+
        geom_text(aes(label=sig2),color="white")+
        theme(axis.text.x=element_text(angle=120,hjust=1),axis.text.y=element_text(angle=180,hjust=0))+xlab("")+ylab(""))
```

```
#Make second heatmap for figure 2 excluding gene pairs with positive interaction including SAP30
#and gene pairs that did not have estimate in all 5 conditions
rem_pairs1 = rep_dat_l$gene_pair[which(is.na(rep_dat_l$mean))] #names of those with missing values
rem_pairs2 = temp$gene_pair2[which(temp$query_target=="SAP30"&temp$YPEG>(gi_sd+gi_mean))] #names of those with pos SAP30 (z score >1)
sig_pairs2 = sig_pairs[-which(sig_pairs%in%rem_pairs1|sig_pairs%in%rem_pairs2)]
print(length(sig_pairs2))
```

```
## [1] 131
```

```
coldist=dist(subset(rep_dat,gene_pair%in%sig_pairs2)[,8:12])
colclust = hclust(coldist,method="average")
colclust.d = as.dendrogram(colclust)
colorder=colclust$labels[colclust$order]
```

```
par(mar=c(10,4,4,2))
plot(colclust.d,edgePar=list(lwd=3))
```

```
par(mar=c(5,4,4,2))
```

```
#make heatmap
rep_dat_plot = subset(rep_dat_l,gene_pair%in%sig_pairs2)
rep_dat_plot$gene_pair = factor(rep_dat_plot$gene_pair, levels = colorder)
print(qplot(x=condition, y=gene_pair, data=rep_dat_plot,
            fill=mean,geom="tile")+
        scale_fill_gradientn(colours=c("steelblue","black","goldenrod"),
                             values=rescale(c(-0.33,zthresh_lower,0,zthresh_upper,0.82)),
                             guide="colorbar",limits=c(-0.33,0.82))+
        geom_text(aes(label=sig2),color="white")+
        theme(axis.text.x=element_text(angle=120,hjust=1),axis.text.y=element_text(angle=180,hjust=0))+xlab("")+ylab(""))
```

Print summary stats cited in manuscript for this section

```
#print summary stats for manuscript text record number of significant interactions
int_cond #print table of data by condition
```

```
##   cond neg_gi pos_gi pos_sap pos_notsap cond_spec cumulative both
## 1    1     51    216     185         31         1        267  267
## 2    2    260    402     269        133        40        700  662
## 3    3    626    677     311        366       365       1634 1303
## 4    4     80     68       9         59        11       1719  148
## 5    5     97    161      46        115        37       1855  258
```

```
int_cond$cumulative[5]/dim(temp2)[1]*100 #print percent of unique guide pairs with interaction in any condition
```

```
## [1] 14.58907
```

```
int_cond$cumulative[2]/dim(temp2)[1]*100 #print percent of unique guide pairs with interaction in either ypd condition
```

```
## [1] 5.505309
```

```
int_cond$both/dim(temp2)[1]*100 #print percent of guide pairs interacting in each condition
```

```
## [1]  2.099882  5.206449 10.247739  1.163980  2.029099
```

```
sum(int_cond$cond_spec)/dim(temp2)[1]*100 #print percent of condition specific pairs
```

```
## [1] 3.570586
```

```
(int_cond$cumulative[5]/dim(temp2)[1]*100)/(int_cond$cumulative[2]/dim(temp2)[1]*100) #print fold increasing using all 5 conditions versus just rich media
```

```
## [1] 2.65
```

```
#record gene pairs from temp to temp2
temp2$gene_pair = temp$gene_pair[match(rownames(temp2),temp$strain)]
#record percent of unique gene pairs showing a significant interactions in at least one condition
length(unique(temp2$gene_pair[-which(is.na(temp2$gene_pair))]))
```

```
## [1] 6841
```

```
length(unique(temp2$gene_pair[which(temp2$num_pos>0|temp2$num_neg>0)]))/length(unique(temp2$gene_pair[-which(is.na(temp2$gene_pair))]))*100
```

```
## [1] 20.65488
```

```
#remove SAP30 containing pairs and calculate again
temp3 = temp2[-which(temp2$query=="SAP30g7"),]
length(unique(temp3$gene_pair[-which(is.na(temp3$gene_pair))]))
```

```
## [1] 6391
```

```
length(unique(temp3$gene_pair[which(temp3$num_pos>0|temp3$num_neg>0)]))/length(unique(temp3$gene_pair[-which(is.na(temp3$gene_pair))]))*100
```

```
## [1] 16.93006
```

```
#Number of GIs detected in YPD24hr also detected in YPD48hr (and fold increase in magnitude of GI)
#ypd24_gis = which(abs(temp2$YPD24hr)>(2*gi_sd+gi_mean))
ypd24_gis = which(temp2$YPD24hr > zthresh_upper | temp2$YPD24hr < zthresh_lower)
#ypd48_gis = which(abs(temp2$YPD48hr)>(2*gi_sd+gi_mean))
ypd48_gis = which(temp2$YPD48hr > zthresh_upper | temp2$YPD48hr < zthresh_lower)
length(ypd24_gis)
```

```
## [1] 267
```

```
length(which(ypd24_gis%in%ypd48_gis))
```

```
## [1] 229
```

```
length(which(ypd24_gis%in%ypd48_gis))/length(ypd24_gis)*100
```

```
## [1] 85.76779
```

```
sub = temp2[ypd24_gis[which(ypd24_gis%in%ypd48_gis)],]
mean(abs(sub$YPD48hr)/abs(sub$YPD24hr))
```

```
## [1] 1.39651
```
