# Supplementary material for: Improved discovery of genetic interactions using CRISPRiSeq across multiple environments
Source: Genome Res. 2019 Apr;29(4):668–81. doi: 10.1101/gr.246603.118 (PMC6442382; doi:10.1101/gr.246603.118)
Supplement: Supplemental Material [file supp_gr.246603.118_Supplemental_CRISPRiSeq-master.zip › CRISPRiSeq-master/figures/figure3.html]

fig.3


# fig.3

Network analysis: Part 1: Calculate all pairwise correlations for GI profiles of the 760 array guides using different subsets and permutations of the entire data set. First, use all possible condition subsets, i.e. all single conditions, all combinations of 2 conditions, all combinations of 3 conditions etc. Next, choose 8 random sets each of 20, 40, 60 or 80 queries (in any condition). Finally, shuffle/permute the data for each row for all possible combinations of 1-5 conditions before calculating the pairwise correlation coefficients. 17 of the original 760 guides were excluded from this analysis, because they were missing >25% of measurements due to strain dropout. This analysis yields 97 possible samples of the data to compute network statistics on in Part 2.

Part 2: For each of the 97 samples generated above, perform two classification performance tests: Right operator characteristic and precision-recall. Using these characteristics, determine whether there is a relationship between amount of data used and power of the data to predict whether any given guide pair targets the same gene or not (as measured by AUC).

Part 3: Generate network diagram from correlation data from only the YPD24hr condition, and for correlatation data from all 5 conditions. In these networks, only connect starting pool guides with a correlation coefficient greater than a threshold chosen to control for just 0.5% of guide pairs with different targets pass.

```
require(ggplot2)
```

```
## Loading required package: ggplot2
```

```
require(reshape2)
```

```
## Loading required package: reshape2
```

```
require(RColorBrewer)
```

```
## Loading required package: RColorBrewer
```

```
require(pracma)
```

```
## Loading required package: pracma
```

```
require(igraph)
```

```
## Loading required package: igraph
```

```
theme_dbc <- theme_set(theme_gray())
theme_dbc <- theme_update(
  panel.background = element_rect(fill = "white"),
  panel.border = element_rect( colour = "black",fill=NA,size=2),
  panel.grid.major = element_line(colour = "gray93",size=1),
  panel.grid.minor = element_line(colour = "gray98",size=1),
  strip.text.x = element_text(size=12,face='bold'),
  axis.title = element_text(size=16),
  strip.background = element_rect(colour="black", fill="white",size = 1),
  axis.text = element_text(colour = "black",face="bold",size=16),
  axis.ticks=element_line(color="black",size=2))

#load in array go annotations
array_go = read.table("~/Desktop/SherlockLab2/manuscript_aug2017/code/figures/GO_lists.txt",sep="\t",stringsAsFactors = FALSE,header=TRUE)
```

```
#Load in GI scores in each condition
ypd24_doubles=read.table(file="~/Desktop/SherlockLab2/manuscript_aug2017/code/final_fitness_GI_estimates/ypd24_short.txt",sep="\t",header=TRUE,stringsAsFactors = FALSE)
ypd48_doubles=read.table(file="~/Desktop/SherlockLab2/manuscript_aug2017/code/final_fitness_GI_estimates/ypd48_short.txt",sep="\t",header=TRUE,stringsAsFactors = FALSE)
ypd37_doubles=read.table(file="~/Desktop/SherlockLab2/manuscript_aug2017/code/final_fitness_GI_estimates/ypd37_short.txt",sep="\t",header=TRUE,stringsAsFactors = FALSE)
ypeg_doubles=read.table(file="~/Desktop/SherlockLab2/manuscript_aug2017/code/final_fitness_GI_estimates/ypeg_short.txt",sep="\t",header=TRUE,stringsAsFactors = FALSE)
ura_doubles=read.table(file="~/Desktop/SherlockLab2/manuscript_aug2017/code/final_fitness_GI_estimates/ura_short.txt",sep="\t",header=TRUE,stringsAsFactors = FALSE)
```

Function to generate data frame holding all GI scores from a condition’s data table

```
#columns are 760 starting pool guides
#rows 20 query guides
#cells are GI scores
#use corrected GI scores for all queries except SAP30g7
get_cond_sub = function(data){
  array_names = unique(data$array)
  array_data = data.frame(matrix(nrow=length(unique(data$query)), ncol=length(array_names)))
  names(array_data)=array_names
  rownames(array_data)=unique(data$query)
  
  #loop through all queries
  for(i in 1:dim(array_data)[1]){
    qn = rownames(array_data)[i]
    array_data[i,]=subset(data,query==qn)$gi_score
    }
  return(array_data)
}
```

Define function to calculate all pairwise correlations of starting pool guides

```
#function to return matrix with correlation coeffifient of all pairwise combination of array guides
get_paiwise = function(dat_mat){
  
  #get correlation matrix
  return_dat=as.data.frame(cor(dat_mat,method="spearman",use="pairwise.complete.obs"))
  
  print("cor done")
  #convert top right half of matrix to value of -100, to remove later and avoid duplicate measurements
  for(i in 1:dim(return_dat)[2]){
    for(j in i:dim(return_dat)[2]){
      return_dat[i,j]=-100 #will use later to subset melted data frame
    }
  }
  return(return_dat)  
}


#####Get data for all 5 conditions##########
data = rbind(get_cond_sub(ypd24_doubles),
             get_cond_sub(ypd48_doubles),
             get_cond_sub(ypeg_doubles),
             get_cond_sub(ypd37_doubles),
             get_cond_sub(ura_doubles))

#record and remove array guides which have more than 25% missing values
rem_guides = which(apply(data,2,function(x)length(which(is.na(x))))>25)
data = data[,-rem_guides]

#generate matrix to save correlation data
data4 = cbind(get_paiwise(data),names(data))
temp4 = melt(data4)

#set up data frame to store all the data
array_similarity = temp4[,1:2]
names(array_similarity)=c("guide1","guide2")
array_similarity$gene1 = gsub("-.+","",array_similarity$guide1)
array_similarity$gene2 = gsub("-.+","",array_similarity$guide2)
array_similarity$same_gene = array_similarity$gene1==array_similarity$gene2

#record similarity metrics for all conditions
array_similarity$spearman.all = signif(temp4$value,digits=4)

#write function to add column to data table holding correlation data
add_column = function(in_dat){
  data_c = in_dat[,-c(rem_guides)]
  data_c = cbind(get_paiwise(data_c),names(data_c))
  temp = melt(data_c)
  names(temp)[1:2]=c("guide1","guide2")
  print(dim(temp))
  print(length(which(temp$guide1==array_similarity$guide1)))
  print(length(which(temp$guide2==array_similarity$guide2)))
  return(signif(temp$value,digits=4))
  }

#look at all possible combinations of 4 conditions
array_similarity$spearman.1234 = add_column(rbind(get_cond_sub(ypd24_doubles),
                                                get_cond_sub(ypd48_doubles),
                                                get_cond_sub(ypeg_doubles),
                                                get_cond_sub(ypd37_doubles)))
array_similarity$spearman.1235 = add_column(rbind(get_cond_sub(ypd24_doubles),
                                                get_cond_sub(ypd48_doubles),
                                                get_cond_sub(ypeg_doubles),
                                                get_cond_sub(ura_doubles)))
array_similarity$spearman.1245 = add_column(rbind(get_cond_sub(ypd24_doubles),
                                                get_cond_sub(ypd48_doubles),
                                                get_cond_sub(ypd37_doubles),
                                                get_cond_sub(ura_doubles)))
array_similarity$spearman.1345 = add_column(rbind(get_cond_sub(ypd24_doubles),
                                                get_cond_sub(ypeg_doubles),
                                                get_cond_sub(ypd37_doubles),
                                                get_cond_sub(ura_doubles)))
array_similarity$spearman.2345 = add_column(rbind(get_cond_sub(ypd48_doubles),
                                                get_cond_sub(ypeg_doubles),
                                                get_cond_sub(ypd37_doubles),
                                                get_cond_sub(ura_doubles)))

#check all combinations of 3 conditions
array_similarity$spearman.123 = add_column(rbind(get_cond_sub(ypd24_doubles),
                                                get_cond_sub(ypd48_doubles),
                                                get_cond_sub(ypeg_doubles)))
array_similarity$spearman.124 = add_column(rbind(get_cond_sub(ypd24_doubles),
                                                get_cond_sub(ypd48_doubles),
                                                get_cond_sub(ypd37_doubles)))
array_similarity$spearman.125 = add_column(rbind(get_cond_sub(ypd24_doubles),
                                                get_cond_sub(ypd48_doubles),
                                                get_cond_sub(ura_doubles)))
array_similarity$spearman.134 = add_column(rbind(get_cond_sub(ypd24_doubles),
                                                get_cond_sub(ypeg_doubles),
                                                get_cond_sub(ypd37_doubles)))
array_similarity$spearman.135 = add_column(rbind(get_cond_sub(ypd24_doubles),
                                                get_cond_sub(ypeg_doubles),
                                                get_cond_sub(ura_doubles)))
array_similarity$spearman.145 = add_column(rbind(get_cond_sub(ypd24_doubles),
                                                get_cond_sub(ypd37_doubles),
                                                get_cond_sub(ura_doubles)))
array_similarity$spearman.234 = add_column(rbind(get_cond_sub(ypd48_doubles),
                                                get_cond_sub(ypeg_doubles),
                                                get_cond_sub(ypd37_doubles)))
array_similarity$spearman.235 = add_column(rbind(get_cond_sub(ypd48_doubles),
                                                get_cond_sub(ypeg_doubles),
                                                get_cond_sub(ura_doubles)))
array_similarity$spearman.245 = add_column(rbind(get_cond_sub(ypd48_doubles),
                                                get_cond_sub(ypd37_doubles),
                                                get_cond_sub(ura_doubles)))
array_similarity$spearman.345 = add_column(rbind(get_cond_sub(ypeg_doubles),
                                                get_cond_sub(ypd37_doubles),
                                                get_cond_sub(ura_doubles)))


#check combinations of 2 conditions
array_similarity$spearman.12 = add_column(rbind(get_cond_sub(ypd24_doubles),
                                                get_cond_sub(ypd48_doubles)))
array_similarity$spearman.13 = add_column(rbind(get_cond_sub(ypd24_doubles),
                                                get_cond_sub(ypeg_doubles)))
array_similarity$spearman.14 = add_column(rbind(get_cond_sub(ypd24_doubles),
                                                get_cond_sub(ypd37_doubles)))
array_similarity$spearman.15 = add_column(rbind(get_cond_sub(ypd24_doubles),
                                                get_cond_sub(ura_doubles)))
array_similarity$spearman.23 = add_column(rbind(get_cond_sub(ypd48_doubles),
                                                get_cond_sub(ypeg_doubles)))
array_similarity$spearman.24 = add_column(rbind(get_cond_sub(ypd48_doubles),
                                                get_cond_sub(ypd37_doubles)))
array_similarity$spearman.25 = add_column(rbind(get_cond_sub(ypd48_doubles),
                                                get_cond_sub(ura_doubles)))
array_similarity$spearman.34 = add_column(rbind(get_cond_sub(ypeg_doubles),
                                                get_cond_sub(ypd37_doubles)))
array_similarity$spearman.35 = add_column(rbind(get_cond_sub(ypeg_doubles),
                                                get_cond_sub(ura_doubles)))
array_similarity$spearman.45 = add_column(rbind(get_cond_sub(ypd37_doubles),
                                                get_cond_sub(ura_doubles)))

#record correlations for each of the 5 individual conditions
array_similarity$spearman.ypd24 = add_column(get_cond_sub(ypd24_doubles))
array_similarity$spearman.ypd48 = add_column(get_cond_sub(ypd48_doubles))
array_similarity$spearman.ypeg = add_column(get_cond_sub(ypeg_doubles))
array_similarity$spearman.ypd37 = add_column(get_cond_sub(ypd37_doubles))
array_similarity$spearman.ura = add_column(get_cond_sub(ura_doubles))
```

Calculate and record correlations for 8 random groups of 20, 40, 60, or 80 guides (ignoring which condition they came from)

```
#For 8 iterations, choose 20 random rows and calculate and record spearman correlations
set.seed(7812)
add_column2 = function(in_dat,no_rows){
  print(dim(in_dat))
  in_dat = in_dat[sample(1:dim(in_dat)[1],no_rows,replace=FALSE),]
  print(dim(in_dat))
  data_c = cbind(get_paiwise(in_dat),names(in_dat))
  temp = melt(data_c)
  names(temp)[1:2]=c("guide1","guide2")
  print(dim(temp))
  print(length(which(temp$guide1==array_similarity$guide1)))
  print(length(which(temp$guide2==array_similarity$guide2)))
  return(signif(temp$value,digits=4))
  }

array_similarity$random80.1=add_column2(data,80)
array_similarity$random80.2=add_column2(data,80)
array_similarity$random80.3=add_column2(data,80)
array_similarity$random80.4=add_column2(data,80)
array_similarity$random80.5=add_column2(data,80)
array_similarity$random80.6=add_column2(data,80)
array_similarity$random80.7=add_column2(data,80)
array_similarity$random80.8=add_column2(data,80)

array_similarity$random60.1=add_column2(data,60)
array_similarity$random60.2=add_column2(data,60)
array_similarity$random60.3=add_column2(data,60)
array_similarity$random60.4=add_column2(data,60)
array_similarity$random60.5=add_column2(data,60)
array_similarity$random60.6=add_column2(data,60)
array_similarity$random60.7=add_column2(data,60)
array_similarity$random60.8=add_column2(data,60)

array_similarity$random40.1=add_column2(data,40)
array_similarity$random40.2=add_column2(data,40)
array_similarity$random40.3=add_column2(data,40)
array_similarity$random40.4=add_column2(data,40)
array_similarity$random40.5=add_column2(data,40)
array_similarity$random40.6=add_column2(data,40)
array_similarity$random40.7=add_column2(data,40)
array_similarity$random40.8=add_column2(data,40)

array_similarity$random20.1=add_column2(data,20)
array_similarity$random20.2=add_column2(data,20)
array_similarity$random20.3=add_column2(data,20)
array_similarity$random20.4=add_column2(data,20)
array_similarity$random20.5=add_column2(data,20)
array_similarity$random20.6=add_column2(data,20)
array_similarity$random20.7=add_column2(data,20)
array_similarity$random20.8=add_column2(data,20)
```

Finally, look at all possible combinations of 1-5 conditions, and permute/shuffle each row’s data before calculating correlation

```
set.seed(8787)
permute_data=function(in_dat2){
  ncols=dim(in_dat2)[2]
  for(i in 1:dim(in_dat2)[1]){
    in_dat2[i,]=in_dat2[i,sample(1:ncols,ncols)]
  }
  return(in_dat2)
}

add_column3 = function(in_dat){
  in_dat = in_dat[,-rem_guides]
  in_dat = permute_data(in_dat)
  data_c = cbind(get_paiwise(in_dat),names(in_dat))
  temp = melt(data_c)
  names(temp)[1:2]=c("guide1","guide2")
  print(dim(temp))
  print(length(which(temp$guide1==array_similarity$guide1)))
  print(length(which(temp$guide2==array_similarity$guide2)))
  return(signif(temp$value,digits=4))
  }

#look at all data permuted (try 5 permutations each)
array_similarity$spearman.all.permuted.1 = add_column3(rbind(get_cond_sub(ypd24_doubles),
                                                             get_cond_sub(ypd48_doubles),
                                                             get_cond_sub(ypeg_doubles),
                                                             get_cond_sub(ypd37_doubles),
                                                             get_cond_sub(ura_doubles)))
array_similarity$spearman.all.permuted.2 = add_column3(rbind(get_cond_sub(ypd24_doubles),
                                                             get_cond_sub(ypd48_doubles),
                                                             get_cond_sub(ypeg_doubles),
                                                             get_cond_sub(ypd37_doubles),
                                                             get_cond_sub(ura_doubles)))
array_similarity$spearman.all.permuted.3 = add_column3(rbind(get_cond_sub(ypd24_doubles),
                                                             get_cond_sub(ypd48_doubles),
                                                             get_cond_sub(ypeg_doubles),
                                                             get_cond_sub(ypd37_doubles),
                                                             get_cond_sub(ura_doubles)))
array_similarity$spearman.all.permuted.4 = add_column3(rbind(get_cond_sub(ypd24_doubles),
                                                             get_cond_sub(ypd48_doubles),
                                                             get_cond_sub(ypeg_doubles),
                                                             get_cond_sub(ypd37_doubles),
                                                             get_cond_sub(ura_doubles)))
array_similarity$spearman.all.permuted.5 = add_column3(rbind(get_cond_sub(ypd24_doubles),
                                                             get_cond_sub(ypd48_doubles),
                                                             get_cond_sub(ypeg_doubles),
                                                             get_cond_sub(ypd37_doubles),
                                                             get_cond_sub(ura_doubles)))

#look at all other combinations of 4
array_similarity$spearman.1234.permuted = add_column3(rbind(get_cond_sub(ypd24_doubles),
                                                           get_cond_sub(ypd48_doubles),
                                                           get_cond_sub(ypeg_doubles),
                                                           get_cond_sub(ypd37_doubles)))
array_similarity$spearman.1235.permuted = add_column3(rbind(get_cond_sub(ypd24_doubles),
                                                           get_cond_sub(ypd48_doubles),
                                                           get_cond_sub(ypeg_doubles),
                                                           get_cond_sub(ura_doubles)))
array_similarity$spearman.1245.permuted = add_column3(rbind(get_cond_sub(ypd24_doubles),
                                                           get_cond_sub(ypd48_doubles),
                                                           get_cond_sub(ypd37_doubles),
                                                           get_cond_sub(ura_doubles)))
array_similarity$spearman.1345.permuted = add_column3(rbind(get_cond_sub(ypd24_doubles),
                                                           get_cond_sub(ypeg_doubles),
                                                           get_cond_sub(ypd37_doubles),
                                                           get_cond_sub(ura_doubles)))
array_similarity$spearman.2345.permuted = add_column3(rbind(get_cond_sub(ypd48_doubles),
                                                           get_cond_sub(ypeg_doubles),
                                                           get_cond_sub(ypd37_doubles),
                                                           get_cond_sub(ura_doubles)))

#check all other combinations of 3
array_similarity$spearman.123.permuted = add_column3(rbind(get_cond_sub(ypd24_doubles),
                                                          get_cond_sub(ypd48_doubles),
                                                          get_cond_sub(ypeg_doubles)))
array_similarity$spearman.124.permuted = add_column3(rbind(get_cond_sub(ypd24_doubles),
                                                          get_cond_sub(ypd48_doubles),
                                                          get_cond_sub(ypd37_doubles)))
array_similarity$spearman.125.permuted = add_column3(rbind(get_cond_sub(ypd24_doubles),
                                                          get_cond_sub(ypd48_doubles),
                                                          get_cond_sub(ura_doubles)))
array_similarity$spearman.134.permuted = add_column3(rbind(get_cond_sub(ypd24_doubles),
                                                          get_cond_sub(ypeg_doubles),
                                                          get_cond_sub(ypd37_doubles)))
array_similarity$spearman.135.permuted = add_column3(rbind(get_cond_sub(ypd24_doubles),
                                                          get_cond_sub(ypeg_doubles),
                                                          get_cond_sub(ura_doubles)))
array_similarity$spearman.145.permuted = add_column3(rbind(get_cond_sub(ypd24_doubles),
                                                          get_cond_sub(ypd37_doubles),
                                                          get_cond_sub(ura_doubles)))
array_similarity$spearman.234.permuted = add_column3(rbind(get_cond_sub(ypd48_doubles),
                                                          get_cond_sub(ypeg_doubles),
                                                          get_cond_sub(ypd37_doubles)))
array_similarity$spearman.235.permuted = add_column3(rbind(get_cond_sub(ypd48_doubles),
                                                          get_cond_sub(ypeg_doubles),
                                                          get_cond_sub(ura_doubles)))
array_similarity$spearman.245.permuted = add_column3(rbind(get_cond_sub(ypd48_doubles),
                                                          get_cond_sub(ypd37_doubles),
                                                          get_cond_sub(ura_doubles)))
array_similarity$spearman.345.permuted = add_column3(rbind(get_cond_sub(ypeg_doubles),
                                                          get_cond_sub(ypd37_doubles),
                                                          get_cond_sub(ura_doubles)))

#check other combinations of 2 conditions
array_similarity$spearman.12.permuted = add_column3(rbind(get_cond_sub(ypd24_doubles),
                                                         get_cond_sub(ypd48_doubles)))
array_similarity$spearman.13.permuted = add_column3(rbind(get_cond_sub(ypd24_doubles),
                                                         get_cond_sub(ypeg_doubles)))
array_similarity$spearman.14.permuted = add_column3(rbind(get_cond_sub(ypd24_doubles),
                                                         get_cond_sub(ypd37_doubles)))
array_similarity$spearman.15.permuted = add_column3(rbind(get_cond_sub(ypd24_doubles),
                                                         get_cond_sub(ura_doubles)))
array_similarity$spearman.23.permuted = add_column3(rbind(get_cond_sub(ypd48_doubles),
                                                         get_cond_sub(ypeg_doubles)))
array_similarity$spearman.24.permuted = add_column3(rbind(get_cond_sub(ypd48_doubles),
                                                         get_cond_sub(ypd37_doubles)))
array_similarity$spearman.25.permuted = add_column3(rbind(get_cond_sub(ypd48_doubles),
                                                         get_cond_sub(ura_doubles)))
array_similarity$spearman.34.permuted = add_column3(rbind(get_cond_sub(ypeg_doubles),
                                                         get_cond_sub(ypd37_doubles)))
array_similarity$spearman.35.permuted = add_column3(rbind(get_cond_sub(ypeg_doubles),
                                                         get_cond_sub(ura_doubles)))
array_similarity$spearman.45.permuted = add_column3(rbind(get_cond_sub(ypd37_doubles),
                                                         get_cond_sub(ura_doubles)))


#Check all single conditions
array_similarity$spearman.24h.permuted = add_column3(get_cond_sub(ypd24_doubles))
array_similarity$spearman.48h.permuted = add_column3(get_cond_sub(ypd48_doubles))
array_similarity$spearman.ypeg.permuted = add_column3(get_cond_sub(ypeg_doubles))
array_similarity$spearman.37C.permuted = add_column3(get_cond_sub(ypd37_doubles))
array_similarity$spearman.ura.permuted = add_column3(get_cond_sub(ura_doubles))
```

Finalize data frame and save for future use

```
#remove rows from data frames that are redundant (i.e. from the top right of correlation matrix or comparing the similarity of exact guide)
array_similarity = array_similarity[-which(array_similarity$spearman.24==-100),]

#record whether each guide pair targets the same bioprocess
array_similarity$same_process = "no"
for(i in 1:dim(array_go)[1]){
  print(array_go[i,1])
  glist=array_go[i,4]
  glist=unlist(strsplit(glist,split=","))
  array_similarity$same_process[which(apply(array_similarity[,3:4],1,function(x)length(which(x%in%glist))==2))]="yes"
  }

write.table(array_similarity, file = "~/Desktop/SherlockLab2/manuscript_aug2017/array_similarity_nov2017.txt",sep="\t")
```

Part 2 Perform analysis to determine whether power to predict function increases with number of conditions screened

```
#load data
#load in pairwise comparison results computed in part 1
array_similarity=read.table(file = "~/Desktop/SherlockLab2/manuscript_aug2017/array_similarity_nov2017.txt",sep="\t",header=TRUE,stringsAsFactors = FALSE)

#mark whether guide pair targets same gene, or a gene in the same bioprocess
array_similarity$group = "different"
array_similarity$group[which(array_similarity$same_process=="yes")]="same_process"
array_similarity$group[which(array_similarity$same_gene==TRUE)]="same_gene"
```

Plot distributions of correlation coefficients for guide pairs with same or different (gene or process) targets

```
table(array_similarity$group)
```

```
## 
##    different    same_gene same_process 
##       239202          422        36029
```

```
names(array_similarity)[c(6,32,105)]
```

```
## [1] "spearman.all"   "spearman.ypd24" "group"
```

```
ggplot(array_similarity[,c(6,105)],aes(x=spearman.all,fill=group))+geom_density(alpha=0.5)+xlim(-1,1)+
  scale_fill_manual(values=c("grey19","red","blue"))+theme(legend.position="none")+xlab("")+ylab("")
```

```
ggplot(array_similarity[,c(32,105)],aes(x=spearman.ypd24,fill=group))+geom_density(alpha=0.5)+
  scale_fill_manual(values=c("grey19","red","blue"))+theme(legend.position="none")+xlab("")+ylab("")+
  xlim(-1,1)
```

```
#compare values for each group
wilcox.test(array_similarity$spearman.all[which(array_similarity$group=="different")],
            array_similarity$spearman.all[which(array_similarity$group=="same_gene")])
```

```
## 
##  Wilcoxon rank sum test with continuity correction
## 
## data:  array_similarity$spearman.all[which(array_similarity$group ==  and array_similarity$spearman.all[which(array_similarity$group ==     "different")] and     "same_gene")]
## W = 10041962, p-value < 2.2e-16
## alternative hypothesis: true location shift is not equal to 0
```

```
wilcox.test(array_similarity$spearman.all[which(array_similarity$group=="different")],
            array_similarity$spearman.all[which(array_similarity$group=="same_process")])
```

```
## 
##  Wilcoxon rank sum test with continuity correction
## 
## data:  array_similarity$spearman.all[which(array_similarity$group ==  and array_similarity$spearman.all[which(array_similarity$group ==     "different")] and     "same_process")]
## W = 3702275796, p-value < 2.2e-16
## alternative hypothesis: true location shift is not equal to 0
```

```
wilcox.test(array_similarity$spearman.all[which(array_similarity$group=="same_gene")],
            array_similarity$spearman.all[which(array_similarity$group=="same_process")])
```

```
## 
##  Wilcoxon rank sum test with continuity correction
## 
## data:  array_similarity$spearman.all[which(array_similarity$group ==  and array_similarity$spearman.all[which(array_similarity$group ==     "same_gene")] and     "same_process")]
## W = 13114042, p-value < 2.2e-16
## alternative hypothesis: true location shift is not equal to 0
```

ROC and PR analysis

```
#for each sample calculate sensitivity/recall, precision and 1 - specificity for calling whether a guide pair
#has the same gene target for different threshold values of spearman's rho
calc_roc_sample=function(test,sample_name){
  #subtract rows with missing rho values, or values of rho (-1,1) which indicate there wasn't enough GI score data to compute a correlation between two profiles
  if(length(which(is.na(test[,2])))>0){test = test[-which(is.na(test[,2])),]}
  if(length(which(test[,2]==1))>0){test = test[-which(test[,2]==1),]}
  if(length(which(test[,2]==-1))>0){test = test[-which(test[,2]==-1),]}
  
  #compute number of potential positives and negatives in the sample
  pos_label = table(test[,1])[2]
  neg_label = table(test[,1])[1]
  
  #build dataframe to store results
  dat = data.frame(matrix(nrow=40,ncol=5))
  names(dat)=c("thresh","sensitivity","one_minus_specificity","precision","sample")
  dat$thresh = seq(from=0.95,to=-1,by=-0.05)
  
  #loop through each threshold and calculate values for ROC and precision/recall
  for(i in 1:dim(dat)[1]){
    tp = length(which(test[,2]>dat$thresh[i]&test[,1]==TRUE)) #calculate true positive calls
    tn = length(which(test[,2]<=dat$thresh[i]&test[,1]==FALSE)) #calculate true negative calls
    fp = length(which(test[,2]>dat$thresh[i]&test[,1]==FALSE)) #calculate false positive calls
    dat$sensitivity[i] = tp/pos_label
    dat$one_minus_specificity[i] = 1-(tn/neg_label)
    dat$precision[i] = tp/(tp+fp)
    }
  dat$sample = sample_name #record sample name
  dat$auc_roc = trapz(dat$one_minus_specificity, dat$sensitivity) #record ROC AUC for this sample
  dat$auc_pr = trapz(dat$sensitivity[-which(is.na(dat$precision))], 
                     dat$precision[-which(is.na(dat$precision))]) #record AUC for precision recall
  return(dat)
}

#calculate values for all samples generated for all possible combinations of data from 1-5 conditions
temp = calc_roc_sample(array_similarity[,5:6],names(array_similarity)[6])
for(i in 7:36){
  temp = rbind(temp,calc_roc_sample(array_similarity[,c(5,i)],names(array_similarity)[i]))
}
#record number of conditions each rows data sample was derived from
temp$no_cond = unlist(lapply(strsplit(gsub(".+\\.","",temp$sample),split=""),length)) #automatically fill in number of conditions each row's data corresponds to from the sample name (only works for 2-4 conditions)
temp$no_cond[1:40]=5 #The first 40 rows are from 5 condition data set
temp$no_cond[1041:1240]=1 #The last 200 rows are from 1 condition data sets

#Calculate values for samples derived from random samples of 20,40,60 or 80 rows (ignoring conditions labels)
temp2 = calc_roc_sample(array_similarity[,c(5,37)],names(array_similarity)[37])
for(i in 38:68){
  temp2 = rbind(temp2,calc_roc_sample(array_similarity[,c(5,i)],names(array_similarity)[i]))
}
#record amount of information each rows data sample was derived from
temp2$no_cond = gsub("\\..+","",temp2$sample)
temp2$no_cond[which(temp2$no_cond=="random20")]=1
temp2$no_cond[which(temp2$no_cond=="random40")]=2
temp2$no_cond[which(temp2$no_cond=="random60")]=3
temp2$no_cond[which(temp2$no_cond=="random80")]=4
temp2$no_cond=as.numeric(temp2$no_cond)

#Calculate values for permuted data
temp3 = calc_roc_sample(array_similarity[,c(5,69)],names(array_similarity)[69])
for(i in 70:103){
  temp3 = rbind(temp3,calc_roc_sample(array_similarity[,c(5,i)],names(array_similarity)[i]))
}
#record number of conditions each rows data sample was derived from
#automatically fill in number of conditions each row's data corresponds to from the sample name (only works for 2-4 conditions)
temp3$no_cond = gsub("\\.permuted","",temp3$sample)
temp3$no_cond = unlist(lapply(strsplit(gsub(".+\\.","",temp3$no_cond),split=""),length))
temp3$no_cond[1:200]=5 #The first 200 rows are from 5 condition data set
temp3$no_cond[1201:1400]=1 #The last 200 rows are from 1 condition data sets

#combine data tables after labelling what subsets of the data are represented
temp$type = "by_cond"
temp2$type = "by_query"
temp3$type = "permuted"
temp5 = rbind(temp,temp2,temp3)

#plot all ROC curves, for each subset of data (1-specificity vs sensitivity)
p=ggplot(temp5,aes(x=one_minus_specificity,y=sensitivity,color=type,group=sample))+geom_line(alpha=0.7)+
  xlim(0,1)+ylim(0,1)+scale_color_manual(values=c("purple","seagreen","grey48"))+theme(legend.position="none")
p+facet_grid(type~no_cond)+geom_vline(xintercept=0.25,linetype="dashed",alpha=0.6)+
  geom_hline(yintercept=0.75,linetype="dashed",alpha=0.6)
```

```
#plot all precision-recall curves, for each subset of data (precision vs recall/sensitivity)
p=ggplot(temp5,aes(x=sensitivity,y=precision,color=type,group=sample))+geom_line(alpha=0.7)+
  xlim(0,1)+ylim(0,1)+scale_color_manual(values=c("purple","seagreen","grey48"))+theme(legend.position="none")
p+facet_grid(type~no_cond)+geom_vline(xintercept=0.25,linetype="dashed",alpha=0.6)+
  geom_hline(yintercept=0.1,linetype="dashed",alpha=0.6)
```

```
## Warning: Removed 3 rows containing missing values (geom_path).
```

```
## Warning: Removed 21 rows containing missing values (geom_path).
```

```
## Warning: Removed 30 rows containing missing values (geom_path).
```

```
## Warning: Removed 18 rows containing missing values (geom_path).
```

```
## Warning: Removed 4 rows containing missing values (geom_path).
```

```
## Warning: Removed 7 rows containing missing values (geom_path).
```

```
## Warning: Removed 20 rows containing missing values (geom_path).
```

```
## Warning: Removed 24 rows containing missing values (geom_path).
```

```
## Warning: Removed 33 rows containing missing values (geom_path).
```

```
## Warning: Removed 8 rows containing missing values (geom_path).
```

```
## Warning: Removed 52 rows containing missing values (geom_path).
```

```
## Warning: Removed 70 rows containing missing values (geom_path).
```

```
## Warning: Removed 43 rows containing missing values (geom_path).
```

```
## Warning: Removed 49 rows containing missing values (geom_path).
```

```
#Figure for main text (only 3 types of subsets, and excluding random data sets)
p=ggplot(subset(temp5,type!="by_query"&no_cond%in%c(1,3,5)),
         aes(x=one_minus_specificity,y=sensitivity,color=type,group=sample))+geom_line(alpha=0.9)+
  xlim(0,1)+ylim(0,1)+scale_color_manual(values=c("purple","grey48"))+theme(legend.position="none")
p+facet_wrap(~no_cond,nrow=1)+geom_vline(xintercept=0.25,linetype="dashed",alpha=0.6)+
  geom_hline(yintercept=0.75,linetype="dashed",alpha=0.6)
```

Plot AUC versus number of conditions for main and supplemental figures (ROC and PR stats)

```
#Figure 3C
ggplot(subset(temp5,type!="by_query"&thresh==0.95),aes(x=no_cond,y=auc_roc,color=type))+
  geom_point(size=6,alpha=0.6)+
  geom_point(size=6,color="black",shape=1)+xlab("")+ylab("")+
  ylim(0,1)+scale_color_manual(values=c("purple","black"))+geom_smooth(method="lm")
```

```
#All data types ROC for supplement
p=ggplot(subset(temp5,thresh==0.95),aes(x=no_cond,y=auc_roc,color=type))+
  geom_point(size=6,alpha=0.6)+
  geom_point(size=6,color="black",shape=1)+xlab("")+ylab("")+
  ylim(0,1)+scale_color_manual(values=c("purple","seagreen","black"))+geom_smooth(method="lm")
p+facet_wrap(~type)
```

```
#Precision recall AUC for supplement
p=ggplot(subset(temp5,thresh==0.95),aes(x=no_cond,y=auc_pr,color=type))+
  geom_point(size=6,alpha=0.6)+
  geom_point(size=6,color="black",shape=1)+xlab("")+ylab("")+
  ylim(0,0.2)+scale_color_manual(values=c("purple","seagreen","black"))+geom_smooth(method="lm")
p+facet_wrap(~type)
```

Part 3 Make network diagrams for correlation data across all conditions versus data from just YPD24h condition Control number False Positive connections in each network by finding the 99.5 percentile value of rho for the group of guide pairs with different targets. Then, when building each network, only connect pairs of guides with correlations greater than that threshold.

```
#set seed for reproducibility
set.seed(8888)

#Loop through index values of columns storing relevant correlation values
for(i in c(6,32:36)){
  sub_data = array_similarity[,c(1:5,i,104:105)] #subset data
  
  #remove rows where correlation values indicate not enough data to compare the gene pair
  if(length(which(sub_data[,6]==1))>0|length(which(sub_data[,6]==-1))>0|length(which(is.na(sub_data[,6])))>0){
    sub_data = sub_data[-which(sub_data[,6]==1|sub_data[,6]==-1|is.na(sub_data[,6])),]
    }
  
  #record sample name
  titles=names(array_similarity)[i]
  
  #find threshold value for Spearman's rho
  threshold=quantile(subset(sub_data,group=="different")[,6], 0.995) 
  names(sub_data)[6]=c("correlation")#rename column containing sample data
  #QC visualize chosen threshold and print number of pairs in each group passing threshold
  print(ggplot(sub_data)+geom_density(aes(x=correlation,fill=group),alpha=0.5)+geom_vline(xintercept=threshold)+ggtitle(titles)+scale_fill_manual(values=c("grey19","red","blue")))
  print(threshold)
  print(table(subset(sub_data,correlation>threshold)$group))

  #generate dataframes to input to igraph
  temp = sub_data[which(sub_data$correlation>threshold),-8]
  names(temp)[1:2]=c("from","to")
  temp$weight = abs(temp[,6])
  temp$color1 = 1
  temp$color1[which(temp$same_process=="yes")]=3
  temp$color1[which(temp$same_gene==TRUE)]=2
  
  temp2 = data.frame(unique(c(temp$from,temp$to)),
                     rep("array",length(unique(c(temp$from,temp$to)))),
                     rep(1,length(unique(c(temp$from,temp$to)))))
  names(temp2)=c("id","guide.label","guide.type")
  temp2$names = gsub("-.+","",temp2$id)
  temp2$p1 = "no"#p1 is mitotic cell cycle
  temp2$p1[which(temp2$names%in%unlist(strsplit(array_go[9,4],split=",")))] = "yes"
  temp2$guide.type[which(temp2$p1=="yes")]=2
  temp2$p2 = "no"#p2 is transmembrane transport
  temp2$p2[which(temp2$names%in%unlist(strsplit(array_go[34,4],split=",")))] = "yes"
  temp2$guide.type[which(temp2$p2=="yes")]=3
  temp2$p3 = "no"#p3 is proteolysis
  temp2$p3[which(temp2$names%in%unlist(strsplit(array_go[22,4],split=",")))] = "yes"
  temp2$guide.type[which(temp2$p3=="yes")]=4
  temp2$p4 = "no"#p4 is small ribosome
  temp2$p4[which(temp2$names%in%unlist(strsplit(array_go[5,4],split=",")))] = "yes"
  temp2$guide.type[which(temp2$p4=="yes")]=5
  temp2$p5 = "no"#p5 is lipid metabolism
  temp2$p5[which(temp2$names%in%unlist(strsplit(array_go[16,4],split=",")))] = "yes"
  temp2$guide.type[which(temp2$p5=="yes")]=6
  temp2$p6 = "no"#p6 is translation
  temp2$p6[which(temp2$names%in%unlist(strsplit(array_go[24,4],split=",")))] = "yes"
  temp2$guide.type[which(temp2$p6=="yes")]=7
  temp2$p7 = "no"
  
  #put data into network data type
  net = graph.data.frame(d=temp,vertices=temp2,directed=F)
  
  #record aesthetics of network and plot
  colrs <- c("gray50", "tomato","darkorchid1","khaki1","lightpink","dodgerblue","darkseagreen3")
  V(net)$color <- colrs[V(net)$guide.type]
  E(net)$width=E(net)$weight
  colrs2 <- c("grey19","red","blue")
  E(net)$color = colrs2[E(net)$color1]
  
  #plot network
  l <- layout.fruchterman.reingold(net)
  print(plot(net, layout=l,vertex.size=3,vertex.label=NA,main=titles))
  
}
```

```
##     99.5% 
## 0.4799995 
## 
##    different    same_gene same_process 
##         1197          106          483
```

```
## NULL
```

```
##  99.5% 
## 0.7098 
## 
##    different    same_gene same_process 
##         1175           53          294
```

```
## NULL
```

```
## 99.5% 
##   0.8 
## 
##    different    same_gene same_process 
##          922           51          268
```

```
## NULL
```

```
##  99.5% 
## 0.7719 
## 
##    different    same_gene same_process 
##         1190           28          240
```

```
## NULL
```

```
##  99.5% 
## 0.6496 
## 
##    different    same_gene same_process 
##         1188           24          224
```

```
## NULL
```

```
##  99.5% 
## 0.6767 
## 
##    different    same_gene same_process 
##         1184           17          268
```

```
## NULL
```
