# Supplementary material for: Improved discovery of genetic interactions using CRISPRiSeq across multiple environments
Source: Genome Res. 2019 Apr;29(4):668–81. doi: 10.1101/gr.246603.118 (PMC6442382; doi:10.1101/gr.246603.118)
Supplement: Supplemental Material [file supp_gr.246603.118_Supplemental_CRISPRiSeq-master.zip › CRISPRiSeq-master/figures/figure4.html]

fig.4


# fig.4

```
require(ggplot2)
```

```
## Loading required package: ggplot2
```

```
require(reshape2)
```

```
## Loading required package: reshape2
```

```
require(RColorBrewer)
```

```
## Loading required package: RColorBrewer
```

```
require(scales)
```

```
## Loading required package: scales
```

```
theme_dbc <- theme_set(theme_gray())
theme_dbc <- theme_update(
  panel.background = element_rect(fill = "white"),
  panel.border = element_rect( colour = "black",fill=NA,size=2),
  panel.grid.major = element_line(colour = "gray93",size=1),
  panel.grid.minor = element_line(colour = "gray98",size=1),
  strip.text.x = element_text(size=12,face='bold'),
  axis.title = element_text(size=16),
  strip.background = element_rect(colour="black", fill="white",size = 1),
  axis.text = element_text(colour = "black",face="bold",size=16),
  axis.ticks=element_line(color="black",size=2))

#load data
ypd24_doubles=read.table(file="~/Desktop/SherlockLab2/manuscript_aug2017/code/final_fitness_GI_estimates/ypd24_short.txt",sep="\t",header=TRUE,stringsAsFactors = FALSE)

ypd48_doubles=read.table(file="~/Desktop/SherlockLab2/manuscript_aug2017/code/final_fitness_GI_estimates/ypd48_short.txt",sep="\t",header=TRUE,stringsAsFactors = FALSE)

ypd37_doubles=read.table(file="~/Desktop/SherlockLab2/manuscript_aug2017/code/final_fitness_GI_estimates/ypd37_short.txt",sep="\t",header=TRUE,stringsAsFactors = FALSE)

ypeg_doubles=read.table(file="~/Desktop/SherlockLab2/manuscript_aug2017/code/final_fitness_GI_estimates/ypeg_short.txt",sep="\t",header=TRUE,stringsAsFactors = FALSE)

ura_doubles=read.table(file="~/Desktop/SherlockLab2/manuscript_aug2017/code/final_fitness_GI_estimates/ura_short.txt",sep="\t",header=TRUE,stringsAsFactors = FALSE)
```

Generate Heatmap of GI scores across conditions from pooled screen for condition-specific interactions validated in low throughput

```
temp=data.frame(ypd24_doubles$gi_score,
                ypd48_doubles$gi_score,
                ypeg_doubles$gi_score,
                ypd37_doubles$gi_score,
                ura_doubles$gi_score)
names(temp)=c("YPD24hr","YPD48hr","YPEG","YPD37","SCURA")

#calculate sd of interaction scores across all conditions
#calculate sd of interaction scores across all conditions
gi_sd = sd(melt(temp)$value,na.rm=TRUE)
```

```
## No id variables; using all as measure variables
```

```
gi_mean = mean(melt(temp)$value,na.rm=TRUE)
```

```
## No id variables; using all as measure variables
```

```
#set thresholds for z score
zval = 2
zthresh_upper = (zval*gi_sd)+gi_mean
zthresh_lower = (-zval*gi_sd)+gi_mean
gi_sd
```

```
## [1] 0.0781284
```

```
gi_mean
```

```
## [1] 0.003516402
```

```
zthresh_upper
```

```
## [1] 0.1597732
```

```
zthresh_lower
```

```
## [1] -0.1527404
```

```
temp$strain = ypd24_doubles$strain
temp$query = ypd24_doubles$query
temp$array = ypd24_doubles$array

temp$query_target = gsub("g.+","",temp$query)
temp$array_target = gsub("-.+","",temp$array)
temp$gene_pair = paste(temp$query_targe,temp$array_target,sep="_")

temp3 = subset(temp,gene_pair%in%c("COG8_CYR1","COG8_CDC25","YCR016W_CYR1","YCR016W_CDC25",
                                   "PRE4_LSM2","PRE4_LSM4","PRE7_LSM2","PRE7_LSM4",
                                   "RPN5_LSM2","RPN5_LSM4"))

temp3=melt(temp3,id.vars=c("strain","query","array","query_target","array_target","gene_pair"))
temp3$strain = factor(temp3$strain,levels=c("COG8g2-CDC25-TRg-1","COG8g2-CDC25-TRg-9","COG8g2-CYR1-TRg-4","COG8g2-CYR1-TRg-7","YCR016Wg4-CDC25-TRg-1","YCR016Wg4-CDC25-TRg-9","YCR016Wg4-CYR1-TRg-4","YCR016Wg4-CYR1-TRg-7","RPN5g1-LSM4-TRg-1","RPN5g1-LSM2-TRg-1","PRE4g9-LSM4-TRg-1","PRE4g9-LSM2-TRg-1","PRE4g3-LSM4-TRg-1","PRE4g3-LSM2-TRg-1","PRE7g7-LSM4-TRg-1","PRE7g7-LSM2-TRg-1"))
temp3$sig = ""
temp3$sig[which((temp3$value > zthresh_upper)|(temp3$value < zthresh_lower))]="*"
ggplot(temp3,aes(x=strain,y=variable,fill=value))+
  geom_tile()+
  theme(axis.text.x=element_text(angle=90,hjust=1,vjust=0.5))+
  geom_text(aes(label=sig),color="white")+
  scale_fill_gradientn(colours=c("steelblue","black","goldenrod"),values=rescale(c(-0.25,zthresh_lower,0,zthresh_upper,0.85)),guide="colorbar",limits=c(-0.25,0.85))
```

Analyze data from OD-based validation assay For Figure 3C

```
#Read in raw data
dat = read.table(file="~/Desktop/SherlockLab2/manuscript_aug2017/code/figures/supp_data/tecan_04082017/04082017.txt",sep="\t",header=FALSE,stringsAsFactors = FALSE)
samples = read.table(file="~/Desktop/SherlockLab2/manuscript_aug2017/code/figures/supp_data/tecan_04082017/samples.csv",sep=",",header=FALSE,stringsAsFactors = FALSE)

#Clean up data
names(dat)[2:97] = samples$V7 #add sample names
dat$V1 = as.numeric(gsub("s","",dat$V1)) #change time stamp to integer value
dat=dat[,-c(53:61,65:97)] #remove empty wells
dat = dat[-c(1:5),] #remove first 5 time points

#reformat to long format and record strain, dose and replicate of each data point
dat_l = melt(dat,id.vars="V1")
dat_l$sample = NA
dat_l$dose = NA
dat_l$rep = NA
for(i in 1:dim(dat_l)[1]){
  temp = unlist(strsplit(as.character(dat_l[i,2]),split="-"))
  dat_l$strain[i] = temp[1]
  dat_l$dose[i] = temp[2]
  dat_l$rep[i] = temp[3]
}
dat_l$sample = gsub("-r.+","",dat_l$variable)
```

QC: Visualize raw OD growth curves for YPD-ATC and YPD+ATC samples for each strain (3 reps each)

```
temp = subset(dat_l,strain%in%c("wt","cog8","cdc25","cog8_cdc25"))
temp$strain = factor(temp$strain,levels=c("wt","cog8","cdc25","cog8_cdc25"))
p=ggplot(temp,aes(x=V1,y=value))+geom_line(aes(color=dose,group=variable),alpha=0.5,size=1.5)+
  scale_x_continuous(breaks = seq(from=0,to=162000,by=18000),
                     labels=seq(from=0,to=45,by=5))+xlab("Hours")+ylab("OD595")
p+facet_wrap(~strain)
```

```
temp = subset(dat_l,strain%in%c("wt","ycr016w","cdc25","ycr_cdc25"))
temp$strain = factor(temp$strain,levels=c("wt","ycr016w","cdc25","ycr_cdc25"))
p=ggplot(temp,aes(x=V1,y=value))+geom_line(aes(color=dose,group=variable),alpha=0.5,size=1.5)+
  scale_x_continuous(breaks = seq(from=0,to=162000,by=18000),
                     labels=seq(from=0,to=45,by=5))+xlab("Hours")+ylab("OD595")
p+facet_wrap(~strain)
```

```
temp = subset(dat_l,strain%in%c("wt","cog8","cyr1","cog8_cyr1"))
temp$strain = factor(temp$strain,levels=c("wt","cog8","cyr1","cog8_cyr1"))
p=ggplot(temp,aes(x=V1,y=value))+geom_line(aes(color=dose,group=variable),alpha=0.5,size=1.5)+
  scale_x_continuous(breaks = seq(from=0,to=162000,by=18000),
                     labels=seq(from=0,to=45,by=5))+xlab("Hours")+ylab("OD595")
p+facet_wrap(~strain)
```

```
temp = subset(dat_l,strain%in%c("wt","ycr016w","cyr1","ycr_cyr1"))
temp$strain = factor(temp$strain,levels=c("wt","ycr016w","cyr1","ycr_cyr1"))
p=ggplot(temp,aes(x=V1,y=value))+geom_line(aes(color=dose,group=variable),alpha=0.5,size=1.5)+
  scale_x_continuous(breaks = seq(from=0,to=162000,by=18000),
                     labels=seq(from=0,to=45,by=5))+xlab("Hours")+ylab("OD595")
p+facet_wrap(~strain)
```

Calculate maximum doubling time for each sample using window tiling method (code from Jaffe et al, G3, 2017 analysis, which was based on St. Onge. Nat. Genet., 2007) \* Doubling time D = (Tf - Ti)/2 \* Time final (at abitrary maximum ODmax), Tf = (ODmax - b)/m \* m and b, are given from the linear regression fitting of the data \* Time initial (2 generations earlier at OD(max-2)), Ti = (OD(max-2)-b)m \* Will use 0.95 for ODmax, 0.2375 for OD(max-2), and the values of m and b from the linear regression fitting of the points between 0.35 and 0.95 \* Fitness is then calculated by comparing Doubling time of each strain to the WT

```
#subset data to just the first 92 time points (24hrs)
dat2 = dat[1:92,]

#loop through each sample and look at each window of 10 timepoints and record maximum slope
time=dat2$V1[1:92]
log_reg_data = data.frame(matrix(ncol=54,nrow=3))#54 columns because there are 54 samples
names(log_reg_data)=names(dat2[2:55])#skip first column, because this is not a sample, it is the time values
for (col in c(2:55)){#loop through each sample
  max_m = 0
  end = length(time) - 9
  for (start_index in c(1:end)){#loop through each 10 time point window
    end_index = start_index + 9
    sub_OD = log2(dat2[start_index:end_index,col])
    sub_time = time[start_index:end_index]
    lm_result = coefficients(lm(sub_OD~sub_time))
    b = lm_result[1]
    m = lm_result[2]
    if (m > max_m){
      max_m = m
      max_b = b
      max_window = c(start_index:end_index)
    }
  }
  #print(max_m)
  log_reg_data[,(col-1)] = c(max_m,max_b,max_window[1])
}

#calculate doubling time for each strain
doublings_log = apply(log_reg_data, 2, function(x) (((log2(0.95)-x[2])/x[1])-(log2(0.2375)-x[2])/x[1])/2/60/60) 

#get doubling time data into correct form for faceted plotting
doublings_long = melt(doublings_log)
doublings_long$variable = names(doublings_log)
doublings_long$sample=gsub("-r.+","",doublings_long$variable)
doublings_long$strain = NA
doublings_long$dose = NA
doublings_long$rep = NA
for(i in 1:dim(doublings_long)[1]){
  temp = unlist(strsplit(as.character(doublings_long[i,2]),split="-"))
  doublings_long$strain[i] = temp[1]
  doublings_long$dose[i] = temp[2]
  doublings_long$rep[i] = temp[3]
}

#plot!
db = ggplot(doublings_long)+geom_point(aes(x=dose, y=value,color=dose),size=5,alpha=0.5)+
  ylab("Doubling time (hr)")+xlab("")+theme(axis.text.x=element_blank(), axis.ticks.x=element_blank())
db + facet_wrap(~strain)
```

Calculate fitness for each genotype by comparing doubling times in the presence vs absence of inducing drug

```
#get unique strain sample names and put together a table to store fitness data
usamp = unique(doublings_long$sample[c(13:24,37:48,52:54)])
doubling_fit = data.frame(usamp)
names(doubling_fit)="sample"
doubling_fit$strain = gsub("-.+","",doubling_fit$sample)
doubling_fit$dose = gsub(".+-","",doubling_fit$sample)
doubling_fit$mean = NA
doubling_fit$sd = NA
doubling_fit=cbind(doubling_fit,data.frame(matrix(nrow=9,ncol=9)))
for(i in 1:length(usamp)){#loop through all strain samples
  
  #get doubling times for all treated samples for this genotype
  temp = doublings_long[which(doublings_long$sample==usamp[i]),]
  treat_samp = temp$value
  
  #get doubling times for all untreated samples for this genotype
  control_samp = doublings_long$value[which(doublings_long$dose=="no_atc"&doublings_long$strain==unique(temp$strain))]
  
  #loop through each pairwise combination of treated/control and record fitness
  values = NA
  count = 0
  for(j in 1:length(treat_samp)){
    for(k in 1:length(control_samp)){
      count = count+1
      values[count]=control_samp[k]/treat_samp[j] #fitness is control over treated doubling time 
    }
  }
  
  #record results
  doubling_fit$mean[i]=mean(values)
  doubling_fit$sd[i]=sd(values)
  doubling_fit[i,6:14]=values
}
doubling_fit$strain = factor(doubling_fit$strain,levels=c("wt","cog8","ycr016w","cdc25","cog8_cdc25","ycr_cdc25","cyr1","cog8_cyr1","ycr_cyr1"))

#save n-values to be used for building 95% confidence intervals
no_strains = 3


#function to plot fitness for 4 strains required to infer a GI between a gene pair
plot_pair=function(gene1,gene2,gene3,adjustment){
  row_i = which(doubling_fit$strain==gene1)
  row_j = which(doubling_fit$strain==gene2)
  
  #calculate expected double mutant fitness based on 95% CI of all pairwise products of single mutant fitness
  expected = NA
  count = 0
  for(i in 6:14){
    for(j in 6:14){
      count = count + 1
      expected[count]=doubling_fit[row_i,i]*doubling_fit[row_j,j]
    }
  }
  temp = subset(doubling_fit,strain%in%c("wt",gene1,gene2,gene3))
  
  
  #get 95% CI around mean
  temp$ymin = temp$mean-1.96*(temp$sd/sqrt(no_strains))
  temp$ymax = temp$mean+1.96*(temp$sd/sqrt(no_strains))
  
  print(temp)
  
  ylimits = aes(ymin=ymin,ymax=ymax)
  print(ggplot(temp,aes(x=strain,y=mean))+
          geom_bar(aes(fill=strain),alpha=0.7,color="black",stat="identity")+
          geom_errorbar(ylimits,width=0.5,size=1)+scale_y_continuous(breaks=c(0.25,0.5,0.75,1),limits=c(0,1.1))+
          annotate("rect",xmin=as.numeric(temp$strain[[1]])-0.6,
                   xmax=as.numeric(temp$strain[[4]])+adjustment,
                   ymax=(mean(expected)+1.96*(sd(expected)/sqrt(no_strains))),
                   ymin=(mean(expected)-1.96*(sd(expected)/sqrt(no_strains))),alpha=0.7)+
          geom_hline(yintercept=mean(expected),size=2)+
          geom_hline(yintercept=(mean(expected)+1.96*(sd(expected)/sqrt(no_strains))),linetype="dashed")+
          geom_hline(yintercept=(mean(expected)-1.96*(sd(expected)/sqrt(no_strains))),linetype="dashed"))

  #print limits of expectation
  print(mean(expected)-1.96*(sd(expected)/sqrt(no_strains)))
  print(mean(expected)+1.96*(sd(expected)/sqrt(no_strains)))
  
  }
plot_pair("cog8","cdc25","cog8_cdc25",-0.4)
```

```
##           sample     strain dose      mean          sd        X1        X2
## 1         wt-atc         wt  atc 0.9909779 0.016941775 0.9604421 0.9876691
## 2       cog8-atc       cog8  atc 0.8994409 0.005863485 0.8902884 0.8999810
## 4      cdc25-atc      cdc25  atc 0.7086104 0.014660371 0.7233764 0.7286813
## 5 cog8_cdc25-atc cog8_cdc25  atc 0.6722055 0.016265272 0.7022912 0.6808311
##          X3        X4        X5        X6        X7        X8        X9
## 1 0.9839895 0.9761782 1.0038512 1.0001113 0.9848321 1.0127505 1.0089774
## 2 0.8954638 0.8995132 0.9093062 0.9047422 0.8935835 0.9033119 0.8987780
## 4 0.7106477 0.7144083 0.7196475 0.7018374 0.6953436 0.7004430 0.6831082
## 5 0.6757044 0.6769543 0.6562684 0.6513266 0.6844329 0.6635185 0.6585221
##        ymin      ymax
## 1 0.9718065 1.0101494
## 2 0.8928058 0.9060761
## 4 0.6920206 0.7252002
## 5 0.6537996 0.6906114
```

```
## [1] 0.622511
## [1] 0.6521953
```

```
plot_pair("cog8","cyr1","cog8_cyr1",-3.3)
```

```
##          sample    strain dose      mean          sd        X1        X2
## 1        wt-atc        wt  atc 0.9909779 0.016941775 0.9604421 0.9876691
## 2      cog8-atc      cog8  atc 0.8994409 0.005863485 0.8902884 0.8999810
## 7      cyr1-atc      cyr1  atc 0.8363642 0.010429350 0.8483605 0.8470970
## 8 cog8_cyr1-atc cog8_cyr1  atc 0.7885174 0.014240738 0.7751032 0.7744858
##          X3        X4        X5        X6        X7        X8        X9
## 1 0.9839895 0.9761782 1.0038512 1.0001113 0.9848321 1.0127505 1.0089774
## 2 0.8954638 0.8995132 0.9093062 0.9047422 0.8935835 0.9033119 0.8987780
## 7 0.8458274 0.8398082 0.8385575 0.8373007 0.8246724 0.8234442 0.8222101
## 8 0.7808813 0.7801826 0.7795612 0.7859985 0.8050281 0.8043869 0.8110292
##        ymin      ymax
## 1 0.9718065 1.0101494
## 2 0.8928058 0.9060761
## 7 0.8245623 0.8481661
## 8 0.7724025 0.8046323
```

```
## [1] 0.7408965
## [1] 0.7636239
```

```
plot_pair("ycr016w","cyr1","ycr_cyr1",-4.4)
```

```
##         sample   strain dose      mean          sd        X1        X2
## 1       wt-atc       wt  atc 0.9909779 0.016941775 0.9604421 0.9876691
## 3  ycr016w-atc  ycr016w  atc 0.9104378 0.010180924 0.9025546 0.9219801
## 7     cyr1-atc     cyr1  atc 0.8363642 0.010429350 0.8483605 0.8470970
## 9 ycr_cyr1-atc ycr_cyr1  atc 0.8115147 0.009221023 0.8082514 0.8161214
##          X3        X4        X5        X6        X7        X8        X9
## 1 0.9839895 0.9761782 1.0038512 1.0001113 0.9848321 1.0127505 1.0089774
## 3 0.9038537 0.8996463 0.9190092 0.9009412 0.9083658 0.9279164 0.9096732
## 7 0.8458274 0.8398082 0.8385575 0.8373007 0.8246724 0.8234442 0.8222101
## 9 0.8147498 0.8156145 0.8235562 0.8221720 0.7963367 0.8040907 0.8027392
##        ymin      ymax
## 1 0.9718065 1.0101494
## 3 0.8989170 0.9219586
## 7 0.8245623 0.8481661
## 9 0.8010801 0.8219492
```

```
## [1] 0.7477653
## [1] 0.7751499
```

```
plot_pair("ycr016w","cdc25","ycr_cdc25",-1.4)
```

```
##          sample    strain dose      mean         sd        X1        X2
## 1        wt-atc        wt  atc 0.9909779 0.01694178 0.9604421 0.9876691
## 3   ycr016w-atc   ycr016w  atc 0.9104378 0.01018092 0.9025546 0.9219801
## 4     cdc25-atc     cdc25  atc 0.7086104 0.01466037 0.7233764 0.7286813
## 6 ycr_cdc25-atc ycr_cdc25  atc 0.6869255 0.00416238 0.6879386 0.6946109
##          X3        X4        X5        X6        X7        X8        X9
## 1 0.9839895 0.9761782 1.0038512 1.0001113 0.9848321 1.0127505 1.0089774
## 3 0.9038537 0.8996463 0.9190092 0.9009412 0.9083658 0.9279164 0.9096732
## 4 0.7106477 0.7144083 0.7196475 0.7018374 0.6953436 0.7004430 0.6831082
## 6 0.6898311 0.6825911 0.6892116 0.6844690 0.6817300 0.6883421 0.6836055
##        ymin      ymax
## 1 0.9718065 1.0101494
## 3 0.8989170 0.9219586
## 4 0.6920206 0.7252002
## 6 0.6822153 0.6916357
```

```
## [1] 0.628857
## [1] 0.6614344
```
