# Supplementary material for: Improved discovery of genetic interactions using CRISPRiSeq across multiple environments
Source: Genome Res. 2019 Apr;29(4):668–81. doi: 10.1101/gr.246603.118 (PMC6442382; doi:10.1101/gr.246603.118)
Supplement: Supplemental Material [file supp_gr.246603.118_Supplemental_CRISPRiSeq-master.zip › CRISPRiSeq-master/figures/GFP_figures_1A_S1.html]

 

 

 

 
 
 


 GFP analysis 

 
 
 
 
 

 
 
 
 
 


 

 

 
 


 
 GFP analysis 
 


 Load packages 
  require(ggplot2)  
  ## Loading required package: ggplot2  
  require(reshape2)  
  ## Loading required package: reshape2  
  require(RColorBrewer)  
  ## Loading required package: RColorBrewer  
  require(mgcv)  
  ## Loading required package: mgcv
## Loading required package: nlme
## This is mgcv 1.8-3. For overview type 'help("mgcv-package")'.  
  theme_dbc <- theme_set(theme_gray())
theme_dbc <- theme_update(
  panel.background = element_rect(fill = "white"),
  panel.border = element_rect( colour = "black",fill=NA,size=2),
  panel.grid.major = element_line(colour = "gray93",size=1),
  panel.grid.minor = element_line(colour = "gray98",size=1),
  strip.text.x = element_text(size=12,face='bold'),
  axis.title = element_text(size=16),
  strip.background = element_rect(colour="black", fill="white",size = 1),
  axis.text = element_text(colour = "black",face="bold",size=16),
  axis.ticks=element_line(color="black",size=2))  
 Load and format in FACs data from 5 days of experiments 
  #Function to read in data
load_data = function(path_name,samples,dat_names){
  no_samples = dim(samples)[1]
  fsc_dat = data.frame(matrix(nrow=10000,ncol=no_samples))
  names(fsc_dat)=samples[,2]
  blu1_dat = data.frame(matrix(nrow=10000,ncol=no_samples))
  names(blu1_dat)=samples[,2]
  
  for(i in 1:dim(samples)[1]){
    file_name = paste(path_name,samples[i,1],sep="")
    sample_name = samples[[i,2]]
    print(sample_name)
    temp = read.table(file_name,sep=",",header=FALSE)
    names(temp)=dat_names[,2]
    
    #remove sample with max FSC value
    rem = unique(c(which(temp[,2]==261621),which(temp[,2]<2000),which(temp[,3]<1000)))
    print(length(rem))
    if(length(rem>=1)){temp = temp[-rem,]}
    
    #add data to matrix
    size = dim(temp)[1]
    fsc_dat[1:size,i]=temp[,2]
    blu1_dat[1:size,i]=temp[,4]
    
    }
  return(cbind(fsc_dat,blu1_dat))
}

all_fsc = data.frame(matrix(nrow = 11500,ncol=61))
all_blu1 = data.frame(matrix(nrow = 11500,ncol=61))

#load data from 12/7/17
samples_exp12_7 = read.table("~/Desktop/SherlockLab2/gfp/Data-12-7-16/name_key.txt",sep="\t",header=FALSE,stringsAsFactors = FALSE)
dat_names_exp12_7 = read.table("~/Desktop/SherlockLab2/gfp/Data-12-7-16/files/file=1-2003.info",sep="\t",header=FALSE)
path_name_exp12_7 = "~/Desktop/SherlockLab2/gfp/Data-12-7-16/files/file="
temp = load_data(path_name_exp12_7,samples_exp12_7,dat_names_exp12_7)  
  ## [1] "GET2g1_4o-1"
## [1] 182
## [1] "GET2g2_4o-1"
## [1] 635
## [1] "NT_4o-1"
## [1] 208
## [1] "GET2g1_4+1"
## [1] 201
## [1] "GET2g1_4+2"
## [1] 170
## [1] "GET2g1_4+3"
## [1] 176
## [1] "GET2g1_4-1"
## [1] 168
## [1] "GET2g1_4-2"
## [1] 845
## [1] "GET2g1_4-3"
## [1] 399
## [1] "GET2g2_4+1"
## [1] 171
## [1] "GET2g2_4+2"
## [1] 142
## [1] "GET2g2_4+3"
## [1] 499
## [1] "GET2g2_4-1"
## [1] 275
## [1] "GET2g2_4-2"
## [1] 953
## [1] "GET2g2_4-3"
## [1] 178
## [1] "NT_4+1"
## [1] 214
## [1] "NT_4+2"
## [1] 191
## [1] "NT_4+3"
## [1] 166
## [1] "GET2g1_8+1"
## [1] 249
## [1] "GET2g1_8+2"
## [1] 283
## [1] "GET2g1_8+3"
## [1] 228
## [1] "GET2g1_8-1"
## [1] 200
## [1] "GET2g1_8-2"
## [1] 205
## [1] "GET2g1_8-3"
## [1] 217
## [1] "GET2g2_8+1"
## [1] 266
## [1] "GET2g2_8+2"
## [1] 293
## [1] "GET2g2_8+3"
## [1] 274
## [1] "GET2g2_8-1"
## [1] 268
## [1] "GET2g2_8-2"
## [1] 184
## [1] "GET2g2_8-3"
## [1] 232
## [1] "NT_8+1"
## [1] 198
## [1] "NT_8+2"
## [1] 196
## [1] "NT_8+3"
## [1] 179
## [1] "GET2g1_25_BY4741_75"
## [1] 135
## [1] "GET2g1_50_BY4741_50"
## [1] 176
## [1] "GET2g1_75_BY4741_25"
## [1] 182
## [1] "GET2g2_25_BY4741_75"
## [1] 100
## [1] "GET2g2_50_BY4741_50"
## [1] 168
## [1] "GET2g2_75_BY4741_25"
## [1] 197  
  names(temp)[c(3,10:18,38)]  
  ##  [1] "NT_4o-1"             "GET2g2_4+1"          "GET2g2_4+2"         
##  [4] "GET2g2_4+3"          "GET2g2_4-1"          "GET2g2_4-2"         
##  [7] "GET2g2_4-3"          "NT_4+1"              "NT_4+2"             
## [10] "NT_4+3"              "GET2g2_50_BY4741_50"  
  names(temp)[c(42,49:57,77)]  
  ##  [1] "NT_4o-1"             "GET2g2_4+1"          "GET2g2_4+2"         
##  [4] "GET2g2_4+3"          "GET2g2_4-1"          "GET2g2_4-2"         
##  [7] "GET2g2_4-3"          "NT_4+1"              "NT_4+2"             
## [10] "NT_4+3"              "GET2g2_50_BY4741_50"  
  names(temp)[c(3,10:18,38)]==names(temp)[c(42,49:57,77)]  
  ##  [1] TRUE TRUE TRUE TRUE TRUE TRUE TRUE TRUE TRUE TRUE TRUE  
  fsc_temp = temp[,c(3,10:18,38)]
blu1_temp = temp[,c(42,49:57,77)]
no_samples = dim(fsc_temp)[2]

all_fsc[1:dim(fsc_temp)[1],1:no_samples]=fsc_temp
names(all_fsc)[1:no_samples]=names(fsc_temp)

all_blu1[1:dim(blu1_temp)[1],1:no_samples]=blu1_temp
names(all_blu1)[1:no_samples]=names(blu1_temp)

col_count = 1 + no_samples

#load data from 1/11/17
samples_exp1_11 = read.table("~/Desktop/SherlockLab2/gfp/Data-1-11-17/name_key.txt",sep="\t",header=FALSE,stringsAsFactors = FALSE)
dat_names_exp1_11 = read.table("~/Desktop/SherlockLab2/gfp/Data-1-11-17/files/file=2-2004.info",sep="\t",header=FALSE)
path_name_exp1_11 = "~/Desktop/SherlockLab2/gfp/Data-1-11-17/files/file="
temp = load_data(path_name_exp1_11,samples_exp1_11,dat_names_exp1_11)  
  ## [1] "MIA165_No"
## [1] 133
## [1] "MIA183_No"
## [1] 110
## [1] "MIA187_No"
## [1] 126
## [1] "MIA165 GFP_BY4741_2575_No"
## [1] 370
## [1] "MIA165 GFP_BY4741_5050_No"
## [1] 80
## [1] "MIA165 GFP_BY4741_7525_No"
## [1] 126
## [1] "MIA183 GFP_BY4741_2575_No"
## [1] 72
## [1] "MIA183 GFP_BY4741_5050_No"
## [1] 70
## [1] "MIA183 GFP_BY4741_7525_No"
## [1] 92
## [1] "MIA187 GFP_BY4741_2575_No"
## [1] 67
## [1] "MIA187 GFP_BY4741_5050_No"
## [1] 116
## [1] "MIA187 GFP_BY4741_7525_No"
## [1] 133
## [1] "MIA153_MIA151_COG8g1_Yes"
## [1] 166
## [1] "MIA153_MIA151_COG8g1_No"
## [1] 167
## [1] "MIA155_MIA151_COG8g2_Yes"
## [1] 102
## [1] "MIA155_MIA151_COG8g2_No"
## [1] 121
## [1] "MIA157_MIA151_COG8g3_Yes"
## [1] 128
## [1] "MIA157_MIA151_COG8g3_No"
## [1] 118
## [1] "MIA159_MIA151_COG8g4_Yes"
## [1] 113
## [1] "MIA159_MIA151_COG8g4_No"
## [1] 114
## [1] "MIA161_MIA151_COG8g5_Yes"
## [1] 114
## [1] "MIA161_MIA151_COG8g5_No"
## [1] 73
## [1] "MIA163_MIA151_COG8g6_Yes"
## [1] 95
## [1] "MIA163_MIA151_COG8g6_No"
## [1] 107
## [1] "MIA165_MIA151_CC16_Yes"
## [1] 142
## [1] "MIA167_MIA148_SAP30g1_Yes"
## [1] 157
## [1] "MIA167_MIA148_SAP30g1_No"
## [1] 127
## [1] "MIA169_MIA148_SAP30g2_Yes"
## [1] 133
## [1] "MIA169_MIA148_SAP30g2_No"
## [1] 148
## [1] "MIA171_MIA148_SAP30g3_Yes"
## [1] 102
## [1] "MIA171_MIA148_SAP30g3_No"
## [1] 105
## [1] "MIA173_MIA148_SAP30g4_Yes"
## [1] 96
## [1] "MIA173_MIA148_SAP30g4_No"
## [1] 105
## [1] "MIA175_MIA148_SAP30g5_Yes"
## [1] 140
## [1] "MIA175_MIA148_SAP30g5_No"
## [1] 91
## [1] "MIA177_MIA148_SAP30g6_Yes"
## [1] 126
## [1] "MIA177_MIA148_SAP30g6_No"
## [1] 102
## [1] "MIA179_MIA148_SAP30g7_Yes"
## [1] 101
## [1] "MIA179_MIA148_SAP30g7_No"
## [1] 137
## [1] "MIA181_MIA148_SAP30g8_Yes"
## [1] 77
## [1] "MIA181_MIA148_SAP30g8_No"
## [1] 127
## [1] "MIA183_MIA148_CC16_Yes"
## [1] 135
## [1] "MIA185_MIA141_RPN5g1_Yes"
## [1] 285
## [1] "MIA185_MIA141_RPN5g1_No"
## [1] 146
## [1] "MIA187_MIA141_CC16_Yes"
## [1] 175  
  names(temp)[c(5,8,11,15,16,25,38,39,42:45)]  
  ##  [1] "MIA165 GFP_BY4741_5050_No" "MIA183 GFP_BY4741_5050_No"
##  [3] "MIA187 GFP_BY4741_5050_No" "MIA155_MIA151_COG8g2_Yes" 
##  [5] "MIA155_MIA151_COG8g2_No"   "MIA165_MIA151_CC16_Yes"   
##  [7] "MIA179_MIA148_SAP30g7_Yes" "MIA179_MIA148_SAP30g7_No" 
##  [9] "MIA183_MIA148_CC16_Yes"    "MIA185_MIA141_RPN5g1_Yes" 
## [11] "MIA185_MIA141_RPN5g1_No"   "MIA187_MIA141_CC16_Yes"  
  names(temp)[c(50,53,56,60,61,70,83,84,87:90)]  
  ##  [1] "MIA165 GFP_BY4741_5050_No" "MIA183 GFP_BY4741_5050_No"
##  [3] "MIA187 GFP_BY4741_5050_No" "MIA155_MIA151_COG8g2_Yes" 
##  [5] "MIA155_MIA151_COG8g2_No"   "MIA165_MIA151_CC16_Yes"   
##  [7] "MIA179_MIA148_SAP30g7_Yes" "MIA179_MIA148_SAP30g7_No" 
##  [9] "MIA183_MIA148_CC16_Yes"    "MIA185_MIA141_RPN5g1_Yes" 
## [11] "MIA185_MIA141_RPN5g1_No"   "MIA187_MIA141_CC16_Yes"  
  names(temp)[c(5,8,11,15,16,25,38,39,42:45)]==names(temp)[c(50,53,56,60,61,70,83,84,87:90)]  
  ##  [1] TRUE TRUE TRUE TRUE TRUE TRUE TRUE TRUE TRUE TRUE TRUE TRUE  
  fsc_temp = temp[,c(5,8,11,15,16,25,38,39,42:45)]
blu1_temp = temp[,c(50,53,56,60,61,70,83,84,87:90)]
no_samples = dim(fsc_temp)[2]
col_range = col_count:(col_count-1+no_samples)

all_fsc[1:dim(fsc_temp)[1],col_range]=fsc_temp
names(all_fsc)[col_range]=names(fsc_temp)

all_blu1[1:dim(blu1_temp)[1],col_range]=blu1_temp
names(all_blu1)[col_range]=names(blu1_temp)
col_count = col_count + no_samples


#load data from 1/25/17
samples_exp1_25 = read.table("~/Desktop/SherlockLab2/gfp/Data-1-25-17/name_key.txt",sep="\t",header=FALSE,stringsAsFactors = FALSE)
dat_names_exp1_25 = read.table("~/Desktop/SherlockLab2/gfp/Data-1-25-17/files/file=1-2003.info",sep="\t",header=FALSE)
path_name_exp1_25 = "~/Desktop/SherlockLab2/gfp/Data-1-25-17/files/file="
temp = load_data(path_name_exp1_25,samples_exp1_25,dat_names_exp1_25)  
  ## [1] "COG8g2_YPD1"
## [1] 44
## [1] "SAP30g7_YPD1"
## [1] 69
## [1] "RPN5g1_YPD1"
## [1] 104
## [1] "RPN5g1_SC1"
## [1] 83
## [1] "COG8g2_BY"
## [1] 42
## [1] "SAP30g7_BY"
## [1] 65
## [1] "RPN5g1YPD_BY"
## [1] 38
## [1] "RPN5g1SC_BY"
## [1] 61
## [1] "RPNg1_YPDplus1"
## [1] 182
## [1] "RPNg1_YPDplus2"
## [1] 171
## [1] "RPNg1_YPDplus3"
## [1] 156
## [1] "RPNg1_YPDminus1"
## [1] 161
## [1] "RPNg1_YPDminus2"
## [1] 154
## [1] "RPNg1_YPDminus3"
## [1] 180
## [1] "RPNg1_SCplus1"
## [1] 81
## [1] "RPNg1_SCplus2"
## [1] 86
## [1] "RPNg1_SCplus3"
## [1] 75
## [1] "RPNg1_SCminus1"
## [1] 78
## [1] "RPNg1_SCminus2"
## [1] 73
## [1] "RPNg1_SCminus3"
## [1] 80
## [1] "COG8g2_YPDplus1"
## [1] 93
## [1] "COG8g2_YPDplus2"
## [1] 71
## [1] "COG8g2_YPDplus3"
## [1] 89
## [1] "COG8g2_YPDminus1"
## [1] 77
## [1] "COG8g2_YPDminus2"
## [1] 63
## [1] "COG8g2_YPDminus3"
## [1] 64
## [1] "SAP30g7_YPDplus1"
## [1] 86
## [1] "SAP30g7_YPDplus2"
## [1] 92
## [1] "SAP30g7_YPDplus3"
## [1] 102
## [1] "SAP30g7_YPDminus1"
## [1] 99
## [1] "SAP30g7_YPDminus2"
## [1] 87
## [1] "SAP30g7_YPDminus3"
## [1] 98  
  names(temp)[c(5:7,9:14,21:32)]  
  ##  [1] "COG8g2_BY"         "SAP30g7_BY"        "RPN5g1YPD_BY"     
##  [4] "RPNg1_YPDplus1"    "RPNg1_YPDplus2"    "RPNg1_YPDplus3"   
##  [7] "RPNg1_YPDminus1"   "RPNg1_YPDminus2"   "RPNg1_YPDminus3"  
## [10] "COG8g2_YPDplus1"   "COG8g2_YPDplus2"   "COG8g2_YPDplus3"  
## [13] "COG8g2_YPDminus1"  "COG8g2_YPDminus2"  "COG8g2_YPDminus3" 
## [16] "SAP30g7_YPDplus1"  "SAP30g7_YPDplus2"  "SAP30g7_YPDplus3" 
## [19] "SAP30g7_YPDminus1" "SAP30g7_YPDminus2" "SAP30g7_YPDminus3"  
  names(temp)[c(37:39,41:46,53:64)]  
  ##  [1] "COG8g2_BY"         "SAP30g7_BY"        "RPN5g1YPD_BY"     
##  [4] "RPNg1_YPDplus1"    "RPNg1_YPDplus2"    "RPNg1_YPDplus3"   
##  [7] "RPNg1_YPDminus1"   "RPNg1_YPDminus2"   "RPNg1_YPDminus3"  
## [10] "COG8g2_YPDplus1"   "COG8g2_YPDplus2"   "COG8g2_YPDplus3"  
## [13] "COG8g2_YPDminus1"  "COG8g2_YPDminus2"  "COG8g2_YPDminus3" 
## [16] "SAP30g7_YPDplus1"  "SAP30g7_YPDplus2"  "SAP30g7_YPDplus3" 
## [19] "SAP30g7_YPDminus1" "SAP30g7_YPDminus2" "SAP30g7_YPDminus3"  
  names(temp)[c(5:7,9:14,21:32)]==names(temp)[c(37:39,41:46,53:64)]  
  ##  [1] TRUE TRUE TRUE TRUE TRUE TRUE TRUE TRUE TRUE TRUE TRUE TRUE TRUE TRUE
## [15] TRUE TRUE TRUE TRUE TRUE TRUE TRUE  
  fsc_temp = temp[,c(5:7,9:14,21:32)]
blu1_temp = temp[,c(37:39,41:46,53:64)]

no_samples = dim(fsc_temp)[2]
col_range = col_count:(col_count-1+no_samples)

all_fsc[1:dim(fsc_temp)[1],col_range]=fsc_temp
names(all_fsc)[col_range]=names(fsc_temp)

all_blu1[1:dim(blu1_temp)[1],col_range]=blu1_temp
names(all_blu1)[col_range]=names(blu1_temp)
col_count = col_count + no_samples

#load data from 2/2/17
samples_exp2_2 = read.table("~/Desktop/SherlockLab2/gfp/Data-2-2-17/name_key.txt",sep="\t",header=FALSE,stringsAsFactors = FALSE)
dat_names_exp2_2 = read.table("~/Desktop/SherlockLab2/gfp/Data-2-2-17/files/file=1-2003.info",sep="\t",header=FALSE)
path_name_exp2_2="~/Desktop/SherlockLab2/gfp/Data-2-2-17/files/file="
temp = load_data(path_name_exp2_2,samples_exp2_2,dat_names_exp2_2)  
  ## [1] "YLR050C_CC8_YPD1"
## [1] 69
## [1] "YCR016W_CC8_YPD1"
## [1] 56
## [1] "RPN5_g1_YPD1"
## [1] 100
## [1] "YLR_BY_25_75"
## [1] 35
## [1] "YLR_BY_50_50"
## [1] 55
## [1] "YLR_BY_75_25"
## [1] 50
## [1] "YCR016W_BY_25_75"
## [1] 27
## [1] "YCR016W_BY_50_50"
## [1] 40
## [1] "YCR016W_BY_75_25"
## [1] 38
## [1] "YLR050C_g1_treated"
## [1] 116
## [1] "YLR050C_g1_untreated"
## [1] 94
## [1] "YLR050C_g2_treated"
## [1] 86
## [1] "YLR050C_g2_untreated"
## [1] 68
## [1] "YLR050C_g3_treated"
## [1] 89
## [1] "YLR050C_g3_untreated"
## [1] 76
## [1] "YLR050C_g4_treated"
## [1] 62
## [1] "YLR050C_g4_untreated"
## [1] 71
## [1] "YLR050C_CC8_treated"
## [1] 115
## [1] "YLR050C_CC8_untreated"
## [1] 98
## [1] "YCR016C_g1_treated"
## [1] 95
## [1] "YCR016C_g1_untreated"
## [1] 85
## [1] "YCR016C_g2_treated"
## [1] 78
## [1] "YCR016C_g2_untreated"
## [1] 75
## [1] "YCR016C_g3_treated"
## [1] 61
## [1] "YCR016C_g3_untreated"
## [1] 77
## [1] "YCR016C_g4_treated"
## [1] 57
## [1] "YCR016C_g4_untreated"
## [1] 85
## [1] "YCR016C_CC8_treated"
## [1] 87
## [1] "YCR016C_CC8_untreated"
## [1] 73  
  names(temp)[c(5,8,10:11,18:19,26:29)]  
  ##  [1] "YLR_BY_50_50"          "YCR016W_BY_50_50"     
##  [3] "YLR050C_g1_treated"    "YLR050C_g1_untreated" 
##  [5] "YLR050C_CC8_treated"   "YLR050C_CC8_untreated"
##  [7] "YCR016C_g4_treated"    "YCR016C_g4_untreated" 
##  [9] "YCR016C_CC8_treated"   "YCR016C_CC8_untreated"  
  names(temp)[c(34,37,39:40,47:48,55:58)]  
  ##  [1] "YLR_BY_50_50"          "YCR016W_BY_50_50"     
##  [3] "YLR050C_g1_treated"    "YLR050C_g1_untreated" 
##  [5] "YLR050C_CC8_treated"   "YLR050C_CC8_untreated"
##  [7] "YCR016C_g4_treated"    "YCR016C_g4_untreated" 
##  [9] "YCR016C_CC8_treated"   "YCR016C_CC8_untreated"  
  names(temp)[c(5,8,10:11,18:19,26:29)]==names(temp)[c(34,37,39:40,47:48,55:58)]  
  ##  [1] TRUE TRUE TRUE TRUE TRUE TRUE TRUE TRUE TRUE TRUE  
  fsc_temp = temp[,c(5,8,10:11,18:19,26:29)]
blu1_temp = temp[,c(34,37,39:40,47:48,55:58)]

no_samples = dim(fsc_temp)[2]
col_range = col_count:(col_count-1+no_samples)

all_fsc[1:dim(fsc_temp)[1],col_range]=fsc_temp
names(all_fsc)[col_range]=names(fsc_temp)

all_blu1[1:dim(blu1_temp)[1],col_range]=blu1_temp
names(all_blu1)[col_range]=names(blu1_temp)
col_count = col_count + no_samples

#load data from 2/14/17
samples_exp2_14 = read.table("~/Desktop/SherlockLab2/gfp/Data-2-14-17/name_key.txt",sep="\t",header=FALSE,stringsAsFactors = FALSE)
dat_names_exp2_14 = read.table("~/Desktop/SherlockLab2/gfp/Data-2-14-17/files/file=1-2004.info",sep="\t",header=FALSE)
path_name_exp2_14 = "~/Desktop/SherlockLab2/gfp/Data-2-14-17/files/file="
temp = load_data(path_name_exp2_14,samples_exp2_14,dat_names_exp2_14)  
  ## [1] "BY4741"
## [1] 183
## [1] "MIA193_RPD3_NT"
## [1] 237
## [1] "MIA196_PRE4_NT"
## [1] 142
## [1] "MIA213_SIN3_NT"
## [1] 115
## [1] "MIA216_MRE11_NT"
## [1] 169
## [1] "MIA185_RPN5_g1"
## [1] 263
## [1] "RPD_BY_50_50"
## [1] 142
## [1] "PRE_BY_50_50"
## [1] 123
## [1] "SIN_BY_50_50"
## [1] 178
## [1] "MRE_BY_50_50"
## [1] 157
## [1] "RPD_g1_plus"
## [1] 237
## [1] "RPD_g1_minus"
## [1] 172
## [1] "RPD_g2_plus"
## [1] 249
## [1] "RPD_g2_minus"
## [1] 209
## [1] "RPD_nt_plus"
## [1] 150
## [1] "RPD_nt_minus"
## [1] 146
## [1] "PRE_g3_plus"
## [1] 92
## [1] "PRE_g3_minus"
## [1] 115
## [1] "PRE_g9_plus"
## [1] 145
## [1] "PRE_g9_minus"
## [1] 177
## [1] "PRE_nt_plus"
## [1] 116
## [1] "PRE_nt_minus"
## [1] 118
## [1] "SIN_g1_plus"
## [1] 159
## [1] "SIN_g1_minus"
## [1] 140
## [1] "SIN_g2_plus"
## [1] 162
## [1] "SIN_g2_minus"
## [1] 186
## [1] "SIN_nt_plus"
## [1] 176
## [1] "SIN_nt_minus"
## [1] 225
## [1] "MRE_g1_plus"
## [1] 193
## [1] "MRE_g1_minus"
## [1] 88
## [1] "MRE_g2_plus"
## [1] 179
## [1] "MRE_g2_minus"
## [1] 194
## [1] "MRE_nt_plus"
## [1] 156
## [1] "MRE_nt_minus"
## [1] 161
## [1] "MRE_nt_minus_b"
## [1] 165
## [1] "MRE_nt_minus_c"
## [1] 170
## [1] "MRE_nt_minus_d"
## [1] 166
## [1] "MRE_nt_minus_e"
## [1] 161  
  names(temp)[c(8,17:22)]  
  ## [1] "PRE_BY_50_50" "PRE_g3_plus"  "PRE_g3_minus" "PRE_g9_plus" 
## [5] "PRE_g9_minus" "PRE_nt_plus"  "PRE_nt_minus"  
  names(temp)[c(46,55:60)]  
  ## [1] "PRE_BY_50_50" "PRE_g3_plus"  "PRE_g3_minus" "PRE_g9_plus" 
## [5] "PRE_g9_minus" "PRE_nt_plus"  "PRE_nt_minus"  
  names(temp)[c(8,17:22)]==names(temp)[c(46,55:60)]  
  ## [1] TRUE TRUE TRUE TRUE TRUE TRUE TRUE  
  fsc_temp = temp[,c(8,17:22)]
blu1_temp = temp[,c(46,55:60)]

no_samples = dim(fsc_temp)[2]
col_range = col_count:(col_count-1+no_samples)

all_fsc[1:dim(fsc_temp)[1],col_range]=fsc_temp
names(all_fsc)[col_range]=names(fsc_temp)

all_blu1[1:dim(blu1_temp)[1],col_range]=blu1_temp
names(all_blu1)[col_range]=names(blu1_temp)
col_count = col_count + no_samples  
 Figure S1A and 1A Make scatter plots to compare 50/50 mix to treated and untreated 
  #One example for RPN5 (Have 4 replicates)
names(all_blu1)[14] #MIA187 strain is the GFP RPN5 strain carrying the control guide  
  ## [1] "MIA187 GFP_BY4741_5050_No"  
  names(all_blu1)[21:22]  
  ## [1] "MIA185_MIA141_RPN5g1_Yes" "MIA185_MIA141_RPN5g1_No"  
  names(all_fsc)[14]  
  ## [1] "MIA187 GFP_BY4741_5050_No"  
  names(all_fsc)[21:22]  
  ## [1] "MIA185_MIA141_RPN5g1_Yes" "MIA185_MIA141_RPN5g1_No"  
  ggplot()+geom_point(aes(y=all_blu1[,14],x=all_fsc[,14]),alpha=0.6)+
  geom_point(aes(y=all_blu1[,22],x=all_fsc[,22]),alpha=0.2,color="turquoise")+
  geom_point(aes(y=all_blu1[,21],x=all_fsc[,21]),alpha=0.2,color="indianred2")+
  scale_x_log10(limits=c(30000,3e5))+scale_y_log10(limits=c(20,10000))+
  xlab("FSC")+ylab("BluFL1")+annotation_logticks(size=2)+ggtitle("RPN5g1")  
  ## Warning in scale$trans$trans(x): NaNs produced  
  ## Warning in scale$trans$trans(x): NaNs produced  
  ## Warning: Removed 1479 rows containing missing values (geom_point).  
  ## Warning: Removed 1451 rows containing missing values (geom_point).  
  ## Warning: Removed 1153 rows containing missing values (geom_point).  
   
  ggplot()+geom_point(aes(y=all_blu1[,14],x=all_fsc[,14]),alpha=0.6)+
  scale_x_log10(limits=c(30000,3e5))+scale_y_log10(limits=c(20,10000))+
  xlab("FSC")+ylab("BluFL1")+annotation_logticks(size=2)+ggtitle("RPN5g1")  
  ## Warning in scale$trans$trans(x): NaNs produced  
  ## Warning: Removed 1479 rows containing missing values (geom_point).  
   
  #One example for GET2g2 (have 3 total)
names(all_blu1)[c(11,5,2)]   
  ## [1] "GET2g2_50_BY4741_50" "GET2g2_4-1"          "GET2g2_4+1"  
  names(all_fsc)[c(11,5,2)]   
  ## [1] "GET2g2_50_BY4741_50" "GET2g2_4-1"          "GET2g2_4+1"  
  ggplot()+geom_point(aes(y=all_blu1[,11],x=all_fsc[,11]),alpha=0.6)+
  geom_point(aes(y=all_blu1[,5],x=all_fsc[,5]),alpha=0.2,color="turquoise")+
  geom_point(aes(y=all_blu1[,2],x=all_fsc[,2]),alpha=0.2,color="indianred2")+
  scale_x_log10(limits=c(30000,3e5))+scale_y_log10(limits=c(20,10000))+
  xlab("FSC")+ylab("BluFL1")+annotation_logticks(size=2)+ggtitle("GET2g2")  
  ## Warning in scale$trans$trans(x): NaNs produced  
  ## Warning: Removed 2000 rows containing missing values (geom_point).  
  ## Warning: Removed 2103 rows containing missing values (geom_point).  
  ## Warning: Removed 2016 rows containing missing values (geom_point).  
   
  ggplot()+geom_point(aes(y=all_blu1[,11],x=all_fsc[,11]),alpha=0.6)+
  scale_x_log10(limits=c(30000,3e5))+scale_y_log10(limits=c(20,10000))+
  xlab("FSC")+ylab("BluFL1")+annotation_logticks(size=2)+ggtitle("GET2g2")  
  ## Warning in scale$trans$trans(x): NaNs produced  
  ## Warning: Removed 2000 rows containing missing values (geom_point).  
   
  #One example for COG8g2 (have 4 total)
names(all_blu1)[c(12,16,15)] #MIA165 is COG8 gfp strain with non targeting control guide  
  ## [1] "MIA165 GFP_BY4741_5050_No" "MIA155_MIA151_COG8g2_No"  
## [3] "MIA155_MIA151_COG8g2_Yes"  
  names(all_fsc)[c(12,16,15)]   
  ## [1] "MIA165 GFP_BY4741_5050_No" "MIA155_MIA151_COG8g2_No"  
## [3] "MIA155_MIA151_COG8g2_Yes"  
  ggplot()+geom_point(aes(y=all_blu1[,12],x=all_fsc[,12]),alpha=0.6)+
  geom_point(aes(y=all_blu1[,16],x=all_fsc[,16]),alpha=0.2,color="turquoise")+
  geom_point(aes(y=all_blu1[,15],x=all_fsc[,15]),alpha=0.2,color="indianred2")+
  scale_x_log10(limits=c(30000,3e5))+scale_y_log10(limits=c(20,10000))+
  xlab("FSC")+ylab("BluFL1")+annotation_logticks(size=2)+ggtitle("COG8g2")  
  ## Warning in scale$trans$trans(x): NaNs produced  
  ## Warning in scale$trans$trans(x): NaNs produced  
  ## Warning in scale$trans$trans(x): NaNs produced  
  ## Warning: Removed 1567 rows containing missing values (geom_point).  
  ## Warning: Removed 1377 rows containing missing values (geom_point).  
  ## Warning: Removed 1498 rows containing missing values (geom_point).  
   
  ggplot()+geom_point(aes(y=all_blu1[,12],x=all_fsc[,12]),alpha=0.6)+
  scale_x_log10(limits=c(30000,3e5))+scale_y_log10(limits=c(20,10000))+
  xlab("FSC")+ylab("BluFL1")+annotation_logticks(size=2)+ggtitle("COG8g2")  
  ## Warning in scale$trans$trans(x): NaNs produced  
  ## Warning: Removed 1567 rows containing missing values (geom_point).  
   
  #One example for SAP30g7 (have 4 total)
names(all_blu1)[c(13,19,18)] #MIA183 is SAP30 gfp strain with non targeting control guide  
  ## [1] "MIA183 GFP_BY4741_5050_No" "MIA179_MIA148_SAP30g7_No" 
## [3] "MIA179_MIA148_SAP30g7_Yes"  
  names(all_fsc)[c(13,19,18)]   
  ## [1] "MIA183 GFP_BY4741_5050_No" "MIA179_MIA148_SAP30g7_No" 
## [3] "MIA179_MIA148_SAP30g7_Yes"  
  ggplot()+geom_point(aes(y=all_blu1[,13],x=all_fsc[,13]),alpha=0.6)+
  geom_point(aes(y=all_blu1[,19],x=all_fsc[,19]),alpha=0.2,color="turquoise")+
  geom_point(aes(y=all_blu1[,18],x=all_fsc[,18]),alpha=0.2,color="indianred2")+
  scale_x_log10(limits=c(30000,3e5))+scale_y_log10(limits=c(20,10000))+
  xlab("FSC")+ylab("BluFL1")+annotation_logticks(size=2)+ggtitle("SAP30g7")  
  ## Warning in scale$trans$trans(x): NaNs produced  
  ## Warning in scale$trans$trans(x): NaNs produced  
  ## Warning in scale$trans$trans(x): NaNs produced  
  ## Warning: Removed 1531 rows containing missing values (geom_point).  
  ## Warning: Removed 1384 rows containing missing values (geom_point).  
  ## Warning: Removed 1436 rows containing missing values (geom_point).  
   
  ggplot()+geom_point(aes(y=all_blu1[,13],x=all_fsc[,13]),alpha=0.6)+
  scale_x_log10(limits=c(30000,3e5))+scale_y_log10(limits=c(20,10000))+
  xlab("FSC")+ylab("BluFL1")+annotation_logticks(size=2)+ggtitle("SAP30g7")  
  ## Warning in scale$trans$trans(x): NaNs produced  
  ## Warning: Removed 1531 rows containing missing values (geom_point).  
   
  #One example for YCR016W (have 1 total)
names(all_blu1)[c(46,52,51)]   
  ## [1] "YCR016W_BY_50_50"     "YCR016C_g4_untreated" "YCR016C_g4_treated"  
  names(all_fsc)[c(46,52,51)]   
  ## [1] "YCR016W_BY_50_50"     "YCR016C_g4_untreated" "YCR016C_g4_treated"  
  ggplot()+geom_point(aes(y=all_blu1[,46],x=all_fsc[,46]),alpha=0.6)+
  geom_point(aes(y=all_blu1[,52],x=all_fsc[,52]),alpha=0.2,color="turquoise")+
  geom_point(aes(y=all_blu1[,51],x=all_fsc[,51]),alpha=0.2,color="indianred2")+
  scale_x_log10(limits=c(30000,3e5))+scale_y_log10(limits=c(20,10000))+
  xlab("FSC")+ylab("BluFL1")+annotation_logticks(size=2)+ggtitle("YCR016Wg4")  
  ## Warning in scale$trans$trans(x): NaNs produced  
  ## Warning: Removed 1286 rows containing missing values (geom_point).  
  ## Warning: Removed 882 rows containing missing values (geom_point).  
  ## Warning: Removed 877 rows containing missing values (geom_point).  
   
  ggplot()+geom_point(aes(y=all_blu1[,46],x=all_fsc[,46]),alpha=0.6)+
  scale_x_log10(limits=c(30000,3e5))+scale_y_log10(limits=c(20,10000))+
  xlab("FSC")+ylab("BluFL1")+annotation_logticks(size=2)+ggtitle("YCR016Wg4")  
  ## Warning in scale$trans$trans(x): NaNs produced  
  ## Warning: Removed 1286 rows containing missing values (geom_point).  
   
  #one example for YLR050C (have 1 total)
names(all_blu1)[c(45,48,47)]   
  ## [1] "YLR_BY_50_50"         "YLR050C_g1_untreated" "YLR050C_g1_treated"  
  names(all_fsc)[c(45,48,47)]   
  ## [1] "YLR_BY_50_50"         "YLR050C_g1_untreated" "YLR050C_g1_treated"  
  ggplot()+geom_point(aes(y=all_blu1[,45],x=all_fsc[,45]),alpha=0.6)+
  geom_point(aes(y=all_blu1[,48],x=all_fsc[,48]),alpha=0.2,color="turquoise")+
  geom_point(aes(y=all_blu1[,47],x=all_fsc[,47]),alpha=0.2,color="indianred2")+
  scale_x_log10(limits=c(30000,3e5))+scale_y_log10(limits=c(20,10000))+
  xlab("FSC")+ylab("BluFL1")+annotation_logticks(size=2)+ggtitle("YLR050Cg1")  
  ## Warning in scale$trans$trans(x): NaNs produced  
  ## Warning in scale$trans$trans(x): NaNs produced  
  ## Warning: Removed 1271 rows containing missing values (geom_point).  
  ## Warning: Removed 639 rows containing missing values (geom_point).  
  ## Warning: Removed 537 rows containing missing values (geom_point).  
   
  ggplot()+geom_point(aes(y=all_blu1[,45],x=all_fsc[,45]),alpha=0.6)+
  scale_x_log10(limits=c(30000,3e5))+scale_y_log10(limits=c(20,10000))+
  xlab("FSC")+ylab("BluFL1")+annotation_logticks(size=2)+ggtitle("YLR050Cg1")  
  ## Warning: Removed 1271 rows containing missing values (geom_point).  
   
  #One example for PRE4g3 (have 1 total)
names(all_blu1)[c(55,57,56)]  
  ## [1] "PRE_BY_50_50" "PRE_g3_minus" "PRE_g3_plus"  
  names(all_fsc)[c(55,57,56)]   
  ## [1] "PRE_BY_50_50" "PRE_g3_minus" "PRE_g3_plus"  
  ggplot()+geom_point(aes(y=all_blu1[,55],x=all_fsc[,55]),alpha=0.6)+
  geom_point(aes(y=all_blu1[,57],x=all_fsc[,57]),alpha=0.2,color="turquoise")+
  geom_point(aes(y=all_blu1[,56],x=all_fsc[,56]),alpha=0.2,color="indianred2")+
  scale_x_log10(limits=c(30000,3e5))+scale_y_log10(limits=c(20,10000))+
  xlab("FSC")+ylab("BluFL1")+annotation_logticks(size=2)+ggtitle("PRE4g3")  
  ## Warning in scale$trans$trans(x): NaNs produced  
  ## Warning: Removed 1025 rows containing missing values (geom_point).  
  ## Warning: Removed 1077 rows containing missing values (geom_point).  
  ## Warning: Removed 1102 rows containing missing values (geom_point).  
   
  ggplot()+geom_point(aes(y=all_blu1[,55],x=all_fsc[,55]),alpha=0.6)+
  scale_x_log10(limits=c(30000,3e5))+scale_y_log10(limits=c(20,10000))+
  xlab("FSC")+ylab("BluFL1")+annotation_logticks(size=2)+ggtitle("PRE4g3")  
  ## Warning in scale$trans$trans(x): NaNs produced  
  ## Warning: Removed 1025 rows containing missing values (geom_point).  
   
  #For PRE4 also show non-targeting plus and minus atc
names(all_blu1)[c(55,61,60)]  
  ## [1] "PRE_BY_50_50" "PRE_nt_minus" "PRE_nt_plus"  
  names(all_fsc)[c(55,61,60)]   
  ## [1] "PRE_BY_50_50" "PRE_nt_minus" "PRE_nt_plus"  
  ggplot()+geom_point(aes(y=all_blu1[,55],x=all_fsc[,55]),alpha=0.6)+
  geom_point(aes(y=all_blu1[,61],x=all_fsc[,61]),alpha=0.2,color="turquoise")+
  geom_point(aes(y=all_blu1[,60],x=all_fsc[,60]),alpha=0.2,color="indianred2")+
  scale_x_log10(limits=c(30000,3e5))+scale_y_log10(limits=c(20,10000))+
  xlab("FSC")+ylab("BluFL1")+annotation_logticks(size=2)+ggtitle("PRE4 non-targeting")  
  ## Warning in scale$trans$trans(x): NaNs produced  
  ## Warning: Removed 1025 rows containing missing values (geom_point).  
  ## Warning: Removed 882 rows containing missing values (geom_point).  
  ## Warning: Removed 1060 rows containing missing values (geom_point).  
   
  #One example for PRE4g9 (have 1 total)
names(all_blu1)[c(55,59,58)]  
  ## [1] "PRE_BY_50_50" "PRE_g9_minus" "PRE_g9_plus"  
  names(all_fsc)[c(55,59,58)]   
  ## [1] "PRE_BY_50_50" "PRE_g9_minus" "PRE_g9_plus"  
  ggplot()+geom_point(aes(y=all_blu1[,55],x=all_fsc[,55]),alpha=0.6)+
  geom_point(aes(y=all_blu1[,59],x=all_fsc[,59]),alpha=0.2,color="turquoise")+
  geom_point(aes(y=all_blu1[,58],x=all_fsc[,58]),alpha=0.2,color="indianred2")+
  scale_x_log10(limits=c(30000,3e5))+scale_y_log10(limits=c(20,10000))+
  xlab("FSC")+ylab("BluFL1")+annotation_logticks(size=2)+ggtitle("PRE4g9")  
  ## Warning in scale$trans$trans(x): NaNs produced  
  ## Warning: Removed 1025 rows containing missing values (geom_point).  
  ## Warning: Removed 856 rows containing missing values (geom_point).  
  ## Warning: Removed 904 rows containing missing values (geom_point).  
   
 Figure S1B Generate violin plots of residuals 
  return_resids = function(training_i, test_i, guide){
  training = data.frame(all_fsc[,training_i],all_blu1[,training_i])
  names(training)=c("FSC","BluFL1")
  
  #subtract missing values and small cell sizes
  training = training[-which(is.na(training$BluFL1)),] #remove rows with values of NA
  training = training[-which(training$FSC<40000),] #remove small cells where data is sparse
  
  #save model and use to predict fluorescence
  mdl = gam(BluFL1~s(FSC,bs="cs"),data=training)
  print(summary(mdl))
  pBluFL1=predict(mdl, data.frame(FSC=training$FSC))
  
  #calculate residuals
  residuals = training$BluFL1-pBluFL1
  print(head(residuals))
  
  #get data for treated sample
  temp = data.frame(all_fsc[,test_i],all_blu1[,test_i])
  names(temp)=c("FSC","BluFL1")
  
  #remove na values or small cell sizes
  if(length(which(is.na(temp$FSC)))!=0){temp = temp[-which(is.na(temp$FSC)),]}
  if(length(which(temp$FSC<40000))){temp = temp[-which(temp$FSC<40000),]}
  
  #calculate residuals
  pBluFL1_test = predict(mdl, data.frame(FSC=temp$FSC))
  residuals_test = temp$BluFL1-pBluFL1_test
  print(head(residuals_test))
  
  #return training and test residuals
  resids = data.frame(matrix(ncol=2,nrow=11820))
  names(resids) = c(paste(guide,"resid_minus",sep="_"),paste(guide,"resid_plus",sep="_"))
  print
  resids[1:length(residuals),1]=residuals
  resids[1:length(residuals_test),2]=residuals_test
  return(resids)
  
  }

all_resids = cbind(return_resids(16,15,"COG8g2"),
                   return_resids(5,2,"GET2g2"),
                   return_resids(57,56,"PRE4g3"),
                   return_resids(59,58,"PRE4g9"),
                   return_resids(22,21,"RPN5g1"),
                   return_resids(19,18,"SAP30g7"),
                   return_resids(52,51,"YCR016Wg4"),
                   return_resids(48,47,"YLR050Cg1"))  
  ## 
## Family: gaussian 
## Link function: identity 
## 
## Formula:
## BluFL1 ~ s(FSC, bs = "cs")
## <environment: 0x11454fb88>
## 
## Parametric coefficients:
##             Estimate Std. Error t value Pr(>|t|)    
## (Intercept)   416.99       1.27   328.3   <2e-16 ***
## ---
## Signif. codes:  0 '***' 0.001 '**' 0.01 '*' 0.05 '.' 0.1 ' ' 1
## 
## Approximate significance of smooth terms:
##         edf Ref.df    F p-value    
## s(FSC) 5.72  6.648 4806  <2e-16 ***
## ---
## Signif. codes:  0 '***' 0.001 '**' 0.01 '*' 0.05 '.' 0.1 ' ' 1
## 
## R-sq.(adj) =  0.755   Deviance explained = 75.5%
## GCV =  16768  Scale est. = 16757     n = 10386
##         1         2         3         4         5         6 
##  29.86883 -68.18100 -77.64325 150.10505 -40.88830  13.70448 
##          1          2          3          4          5          6 
##  -63.64514 -161.03329    2.03349   33.82400  129.85406 -115.94656 
## 
## Family: gaussian 
## Link function: identity 
## 
## Formula:
## BluFL1 ~ s(FSC, bs = "cs")
## <environment: 0x10cb93120>
## 
## Parametric coefficients:
##             Estimate Std. Error t value Pr(>|t|)    
## (Intercept)  447.001      1.707   261.8   <2e-16 ***
## ---
## Signif. codes:  0 '***' 0.001 '**' 0.01 '*' 0.05 '.' 0.1 ' ' 1
## 
## Approximate significance of smooth terms:
##          edf Ref.df    F p-value    
## s(FSC) 6.799  7.752 1453  <2e-16 ***
## ---
## Signif. codes:  0 '***' 0.001 '**' 0.01 '*' 0.05 '.' 0.1 ' ' 1
## 
## R-sq.(adj) =  0.537   Deviance explained = 53.8%
## GCV =  28290  Scale est. = 28267     n = 9699
##         1         2         3         4         5         6 
##  54.00150 -38.58858 216.16070  49.51831 -26.52656 -13.69264 
##          1          2          3          4          5          6 
##  -76.97394 -123.05047  -98.87559   24.10754 -276.92346  -22.41213 
## 
## Family: gaussian 
## Link function: identity 
## 
## Formula:
## BluFL1 ~ s(FSC, bs = "cs")
## <environment: 0x111f76b78>
## 
## Parametric coefficients:
##             Estimate Std. Error t value Pr(>|t|)    
## (Intercept) 2534.798      3.172   799.2   <2e-16 ***
## ---
## Signif. codes:  0 '***' 0.001 '**' 0.01 '*' 0.05 '.' 0.1 ' ' 1
## 
## Approximate significance of smooth terms:
##          edf Ref.df     F p-value    
## s(FSC) 6.254  7.224 16376  <2e-16 ***
## ---
## Signif. codes:  0 '***' 0.001 '**' 0.01 '*' 0.05 '.' 0.1 ' ' 1
## 
## R-sq.(adj) =  0.917   Deviance explained = 91.7%
## GCV = 1.0765e+05  Scale est. = 1.0757e+05  n = 10694
##         1         2         3         4         5         6 
## -417.0336 -128.1839 -295.2510  152.1785  611.1883  213.4633 
##           1           2           3           4           5           6 
##  -605.12472   -38.36933  -611.34118   -82.72733 -1701.75714 -1016.75894 
## 
## Family: gaussian 
## Link function: identity 
## 
## Formula:
## BluFL1 ~ s(FSC, bs = "cs")
## <environment: 0x10ca3f670>
## 
## Parametric coefficients:
##             Estimate Std. Error t value Pr(>|t|)    
## (Intercept) 2494.089      3.266   763.6   <2e-16 ***
## ---
## Signif. codes:  0 '***' 0.001 '**' 0.01 '*' 0.05 '.' 0.1 ' ' 1
## 
## Approximate significance of smooth terms:
##          edf Ref.df     F p-value    
## s(FSC) 5.374  6.315 18095  <2e-16 ***
## ---
## Signif. codes:  0 '***' 0.001 '**' 0.01 '*' 0.05 '.' 0.1 ' ' 1
## 
## R-sq.(adj) =  0.913   Deviance explained = 91.3%
## GCV = 1.1644e+05  Scale est. = 1.1637e+05  n = 10909
##          1          2          3          4          5          6 
##  40.842442 -60.784492 105.183707 -95.910874  -8.773327  56.245267 
##         1         2         3         4         5         6 
## -524.1695 -384.2912 -304.0200 -369.9072 -388.6042 -327.1675 
## 
## Family: gaussian 
## Link function: identity 
## 
## Formula:
## BluFL1 ~ s(FSC, bs = "cs")
## <environment: 0x10b776510>
## 
## Parametric coefficients:
##             Estimate Std. Error t value Pr(>|t|)    
## (Intercept) 1377.165      3.045   452.3   <2e-16 ***
## ---
## Signif. codes:  0 '***' 0.001 '**' 0.01 '*' 0.05 '.' 0.1 ' ' 1
## 
## Approximate significance of smooth terms:
##          edf Ref.df    F p-value    
## s(FSC) 5.536  6.515 4930  <2e-16 ***
## ---
## Signif. codes:  0 '***' 0.001 '**' 0.01 '*' 0.05 '.' 0.1 ' ' 1
## 
## R-sq.(adj) =  0.756   Deviance explained = 75.7%
## GCV =  96028  Scale est. = 95967     n = 10351
##           1           2           3           4           5           6 
##   25.987635 -765.467472  -84.044003  100.039688  272.688275   -2.852894 
##          1          2          3          4          5          6 
##  -820.3839   121.2901 -1104.6904 -1019.1890 -1679.8391  -900.2575 
## 
## Family: gaussian 
## Link function: identity 
## 
## Formula:
## BluFL1 ~ s(FSC, bs = "cs")
## <environment: 0x10d4add40>
## 
## Parametric coefficients:
##             Estimate Std. Error t value Pr(>|t|)    
## (Intercept)   324.50       1.02   318.1   <2e-16 ***
## ---
## Signif. codes:  0 '***' 0.001 '**' 0.01 '*' 0.05 '.' 0.1 ' ' 1
## 
## Approximate significance of smooth terms:
##          edf Ref.df    F p-value    
## s(FSC) 5.421  6.311 4460  <2e-16 ***
## ---
## Signif. codes:  0 '***' 0.001 '**' 0.01 '*' 0.05 '.' 0.1 ' ' 1
## 
## R-sq.(adj) =  0.731   Deviance explained = 73.1%
## GCV =  10776  Scale est. = 10770     n = 10352
##         1         2         3         4         5         6 
##  29.44655  45.12521 -56.39731  86.78551  38.11146  29.43659 
##         1         2         3         4         5         6 
## -42.54130 -77.03074 -13.54585 -20.98839 -58.07486 -53.88578 
## 
## Family: gaussian 
## Link function: identity 
## 
## Formula:
## BluFL1 ~ s(FSC, bs = "cs")
## <environment: 0x10fd78f10>
## 
## Parametric coefficients:
##             Estimate Std. Error t value Pr(>|t|)    
## (Intercept)  609.403      1.351   451.1   <2e-16 ***
## ---
## Signif. codes:  0 '***' 0.001 '**' 0.01 '*' 0.05 '.' 0.1 ' ' 1
## 
## Approximate significance of smooth terms:
##         edf Ref.df    F p-value    
## s(FSC) 4.96  5.854 7659  <2e-16 ***
## ---
## Signif. codes:  0 '***' 0.001 '**' 0.01 '*' 0.05 '.' 0.1 ' ' 1
## 
## R-sq.(adj) =  0.805   Deviance explained = 80.5%
## GCV =  19807  Scale est. = 19797     n = 10847
##          1          2          3          4          5          6 
##  -38.86078 -170.77904 -103.97367   30.33548  -12.01668   24.86364 
##         1         2         3         4         5         6 
## -191.1028 -366.4223 -447.0799 -236.1767 -150.8516 -107.2404 
## 
## Family: gaussian 
## Link function: identity 
## 
## Formula:
## BluFL1 ~ s(FSC, bs = "cs")
## <environment: 0x10fef4f88>
## 
## Parametric coefficients:
##             Estimate Std. Error t value Pr(>|t|)    
## (Intercept)  459.865      1.577   291.6   <2e-16 ***
## ---
## Signif. codes:  0 '***' 0.001 '**' 0.01 '*' 0.05 '.' 0.1 ' ' 1
## 
## Approximate significance of smooth terms:
##          edf Ref.df    F p-value    
## s(FSC) 5.792  6.715 3579  <2e-16 ***
## ---
## Signif. codes:  0 '***' 0.001 '**' 0.01 '*' 0.05 '.' 0.1 ' ' 1
## 
## R-sq.(adj) =  0.685   Deviance explained = 68.5%
## GCV =  27534  Scale est. = 27517     n = 11061
##          1          2          3          4          5          6 
## -86.230288  14.994270  85.322728 206.149942   7.692613 -71.297696 
##           1           2           3           4           5           6 
##    4.249164   37.630798  -47.508118 -117.109274 -132.460730 -113.909139  
  med_resid = data.frame(apply(all_resids,2,function(x)median(x,na.rm=TRUE)))
names(med_resid) = c("median")
med_resid$sample = rownames(med_resid)
med_resid$xval=NA
temp = melt(all_resids)  
  ## No id variables; using all as measure variables  
  temp$xval=NA
samples = unique(temp$variable)
for(i in 1:length(samples)){
  temp$xval[which(temp$variable==samples[i])]=i
  med_resid$xval[which(med_resid$sample==samples[i])]=i
  }
ggplot(temp,aes(x=xval,y=value))+geom_hline()+ylim(-2000,1000)+
  annotate("rect",xmin=0.5,xmax=2.5,ymin=-2000,ymax=1000,alpha=0.3)+
  annotate("rect",xmin=4.5,xmax=6.5,ymin=-2000,ymax=1000,alpha=0.3)+
  annotate("rect",xmin=8.5,xmax=10.5,ymin=-2000,ymax=1000,alpha=0.3)+
  annotate("rect",xmin=12.5,xmax=14.5,ymin=-2000,ymax=1000,alpha=0.3)+
  geom_violin(aes(fill=gsub(".+_","",variable),group=variable))+
  scale_x_continuous(breaks=c(1.5,3.5,5.5,7.5,9.5,11.5,13.5,15.5),
                     labels=c("COG8g2","GET2g2","PRE4g3","PRE4g9",
                              "RPN5g1","SAP30g7","YCR016Wg4","YLR050Cg1"))+
  theme(axis.text.x=element_text(angle=70,hjust=1))+ylab("")+xlab("")+
  geom_point(data=med_resid,aes(x=xval,y=median),shape=95,size=20)+
  scale_fill_manual(values=c("#00BFC4","#F8766D"))  
  ## Warning: Removed 21501 rows containing non-finite values (stat_ydensity).  
   
  cont_resids = cbind(return_resids(1,8,"GET2nt"),
                    return_resids(61,60,"PRE4nt"),
                    return_resids(54,53,"YCR016Wnt"),
                    return_resids(50,49,"YLR050Cnt"))  
  ## 
## Family: gaussian 
## Link function: identity 
## 
## Formula:
## BluFL1 ~ s(FSC, bs = "cs")
## <environment: 0x10b5c4980>
## 
## Parametric coefficients:
##             Estimate Std. Error t value Pr(>|t|)    
## (Intercept)   470.87       1.59   296.2   <2e-16 ***
## ---
## Signif. codes:  0 '***' 0.001 '**' 0.01 '*' 0.05 '.' 0.1 ' ' 1
## 
## Approximate significance of smooth terms:
##          edf Ref.df    F p-value    
## s(FSC) 5.513  6.467 2308  <2e-16 ***
## ---
## Signif. codes:  0 '***' 0.001 '**' 0.01 '*' 0.05 '.' 0.1 ' ' 1
## 
## R-sq.(adj) =  0.605   Deviance explained = 60.5%
## GCV =  24693  Scale est. = 24676     n = 9766
##          1          2          3          4          5          6 
## -14.465075 -60.469310  43.722448   7.554213 -52.634042  16.603320 
##            1            2            3            4            5 
##  -39.5298659 -107.3539226   67.3649508  -25.5018437    0.5292885 
##            6 
##   65.1571779 
## 
## Family: gaussian 
## Link function: identity 
## 
## Formula:
## BluFL1 ~ s(FSC, bs = "cs")
## <environment: 0x111028028>
## 
## Parametric coefficients:
##             Estimate Std. Error t value Pr(>|t|)    
## (Intercept) 2605.162      3.274   795.6   <2e-16 ***
## ---
## Signif. codes:  0 '***' 0.001 '**' 0.01 '*' 0.05 '.' 0.1 ' ' 1
## 
## Approximate significance of smooth terms:
##          edf Ref.df     F p-value    
## s(FSC) 6.398  7.368 15784  <2e-16 ***
## ---
## Signif. codes:  0 '***' 0.001 '**' 0.01 '*' 0.05 '.' 0.1 ' ' 1
## 
## R-sq.(adj) =  0.914   Deviance explained = 91.4%
## GCV = 1.1685e+05  Scale est. = 1.1677e+05  n = 10891
##          1          2          3          4          5          6 
##  -36.43497  198.22369  505.26716 -237.75045  211.19260  -54.78146 
##          1          2          3          4          5          6 
##  -86.45027   42.49931  315.35720 -273.88236   77.84866  138.94085 
## 
## Family: gaussian 
## Link function: identity 
## 
## Formula:
## BluFL1 ~ s(FSC, bs = "cs")
## <environment: 0x1113555f8>
## 
## Parametric coefficients:
##             Estimate Std. Error t value Pr(>|t|)    
## (Intercept)  600.028      1.432     419   <2e-16 ***
## ---
## Signif. codes:  0 '***' 0.001 '**' 0.01 '*' 0.05 '.' 0.1 ' ' 1
## 
## Approximate significance of smooth terms:
##          edf Ref.df    F p-value    
## s(FSC) 5.656  6.572 6303  <2e-16 ***
## ---
## Signif. codes:  0 '***' 0.001 '**' 0.01 '*' 0.05 '.' 0.1 ' ' 1
## 
## R-sq.(adj) =  0.792   Deviance explained = 79.3%
## GCV =  22267  Scale est. = 22253     n = 10853
##         1         2         3         4         5         6 
##  49.91418  49.20313 -51.86718  33.68658 189.55770 174.55648 
##          1          2          3          4          5          6 
##   75.61202 -156.02561  -94.55037 -114.50908  -31.78370   16.42874 
## 
## Family: gaussian 
## Link function: identity 
## 
## Formula:
## BluFL1 ~ s(FSC, bs = "cs")
## <environment: 0x112b220d0>
## 
## Parametric coefficients:
##             Estimate Std. Error t value Pr(>|t|)    
## (Intercept)  442.113      1.307   338.2   <2e-16 ***
## ---
## Signif. codes:  0 '***' 0.001 '**' 0.01 '*' 0.05 '.' 0.1 ' ' 1
## 
## Approximate significance of smooth terms:
##         edf Ref.df    F p-value    
## s(FSC) 5.65  6.565 4844  <2e-16 ***
## ---
## Signif. codes:  0 '***' 0.001 '**' 0.01 '*' 0.05 '.' 0.1 ' ' 1
## 
## R-sq.(adj) =  0.744   Deviance explained = 74.4%
## GCV =  18748  Scale est. = 18737     n = 10961
##          1          2          3          4          5          6 
##  -54.24026   22.91573   85.70131  178.43397  -96.85212 -112.79623 
##         1         2         3         4         5         6 
## -95.89807 -60.26999 -46.55477 391.03070 -24.18579  20.90696  
  med_resid = data.frame(apply(cont_resids,2,function(x)median(x,na.rm=TRUE)))
names(med_resid) = c("median")
med_resid$sample = rownames(med_resid)
med_resid$xval=NA
temp = melt(cont_resids)  
  ## No id variables; using all as measure variables  
  temp$xval=NA
samples = unique(temp$variable)
for(i in 1:length(samples)){
  temp$xval[which(temp$variable==samples[i])]=i
  med_resid$xval[which(med_resid$sample==samples[i])]=i
  }


ggplot(temp,aes(x=xval,y=value))+geom_hline()+ylim(-2000,1000)+
  annotate("rect",xmin=0.5,xmax=2.5,ymin=-2000,ymax=1000,alpha=0.3)+
  annotate("rect",xmin=4.5,xmax=6.5,ymin=-2000,ymax=1000,alpha=0.3)+
  geom_violin(aes(fill=gsub(".+_","",variable),group=variable))+
  scale_x_continuous(breaks=c(1.5,3.5,5.5,7.5),
                     labels=c("GET2nt","PRE4nt","YCR016Wnt","YLR050Cnt"))+
  theme(axis.text.x=element_text(angle=70,hjust=1))+ylab("")+xlab("")+
  geom_point(data=med_resid,aes(x=xval,y=median),shape=95,size=20)+
  scale_fill_manual(values=c("#00BFC4","#F8766D"))  
  ## Warning: Removed 10144 rows containing non-finite values (stat_ydensity).  
   


 

 

 
 

 
 
