# Supplementary material for: Improved discovery of genetic interactions using CRISPRiSeq across multiple environments
Source: Genome Res. 2019 Apr;29(4):668–81. doi: 10.1101/gr.246603.118 (PMC6442382; doi:10.1101/gr.246603.118)
Supplement: Supplemental Material [file supp_gr.246603.118_Supplemental_CRISPRiSeq-master.zip › CRISPRiSeq-master/figures/supp_figs.html]

Suppfigs


# Suppfigs

```
require(ggplot2)
```

```
## Loading required package: ggplot2
```

```
require(reshape2)
```

```
## Loading required package: reshape2
```

```
require(RColorBrewer)
```

```
## Loading required package: RColorBrewer
```

```
theme_dbc <- theme_set(theme_gray())
theme_dbc <- theme_update(
  panel.background = element_rect(fill = "white"),
  panel.border = element_rect( colour = "black",fill=NA,size=2),
  panel.grid.major = element_line(colour = "gray93",size=1),
  panel.grid.minor = element_line(colour = "gray98",size=1),
  strip.text.x = element_text(size=12,face='bold'),
  axis.title = element_text(size=16),
  strip.background = element_rect(colour="black", fill="white",size = 1),
  axis.text = element_text(colour = "black",face="bold",size=16),
  axis.ticks=element_line(color="black",size=2))

#Load in GI scores (short file)
ypd24_doubles=read.table(file="~/Desktop/SherlockLab2/manuscript_aug2017/code/final_fitness_GI_estimates/ypd24_short.txt",sep="\t",header=TRUE,stringsAsFactors = FALSE)
ypd48_doubles=read.table(file="~/Desktop/SherlockLab2/manuscript_aug2017/code/final_fitness_GI_estimates/ypd48_short.txt",sep="\t",header=TRUE,stringsAsFactors = FALSE)
ypd37_doubles=read.table(file="~/Desktop/SherlockLab2/manuscript_aug2017/code/final_fitness_GI_estimates/ypd37_short.txt",sep="\t",header=TRUE,stringsAsFactors = FALSE)
ypeg_doubles=read.table(file="~/Desktop/SherlockLab2/manuscript_aug2017/code/final_fitness_GI_estimates/ypeg_short.txt",sep="\t",header=TRUE,stringsAsFactors = FALSE)
ura_doubles=read.table(file="~/Desktop/SherlockLab2/manuscript_aug2017/code/final_fitness_GI_estimates/ura_short.txt",sep="\t",header=TRUE,stringsAsFactors = FALSE)

#Load in GI scores (long file with single mutant fitness)
ypd24_all_dat=read.table(file="~/Desktop/SherlockLab2/manuscript_aug2017/code/final_fitness_GI_estimates/ypd24_data.txt",sep="\t",header=TRUE,stringsAsFactors = FALSE)
ypd48_all_dat=read.table(file="~/Desktop/SherlockLab2/manuscript_aug2017/code/final_fitness_GI_estimates/ypd48_data.txt",sep="\t",header=TRUE,stringsAsFactors = FALSE)
ypd37_all_dat=read.table(file="~/Desktop/SherlockLab2/manuscript_aug2017/code/final_fitness_GI_estimates/ypd37_data.txt",sep="\t",header=TRUE,stringsAsFactors = FALSE)
ypeg_all_dat=read.table(file="~/Desktop/SherlockLab2/manuscript_aug2017/code/final_fitness_GI_estimates/ypeg_data.txt",sep="\t",header=TRUE,stringsAsFactors = FALSE)
ura_all_dat=read.table(file="~/Desktop/SherlockLab2/manuscript_aug2017/code/final_fitness_GI_estimates/ura_data.txt",sep="\t",header=TRUE,stringsAsFactors = FALSE)

#record names of guides that are carrying both in the query plasmid and in a starting pool strain
rep_query_names=c("PRE7g7","PRE4g9","PRE4g3","RPN5g1","COG3g1","SED5g5","SEC22g2","IMP4g6","DIP2g5","PWP2g2_BC1","TIF6g8","RPF1g3","MAK16g1","PWP2g2_BC2")
rep_array_names=c("PRE7-TRg-7","PRE4-NRg-9","PRE4-TRg-3","RPN5-NRg-1","COG3-TRg-1","SED5-TRg-5","SEC22-NRg-8","IMP4-TRg-6","DIP2-TRg-5","PWP2-NRg-2","TIF6-NRg-8","RPF1-TRg-3","MAK16-TRg-1")

#record sd and mean of all gi_scores
gi_sd = sd(c(ypd24_doubles$gi_score,ypd48_doubles$gi_score,ypeg_doubles$gi_score,ypd37_doubles$gi_score,ura_doubles$gi_score),na.rm=TRUE)
#record mean of all gi_scores
gi_mean = mean(c(ypd24_doubles$gi_score,ypd48_doubles$gi_score,ypeg_doubles$gi_score,ypd37_doubles$gi_score,ura_doubles$gi_score),na.rm=TRUE)

#set thresholds for z score
zval = 2
zthresh_upper = (zval*gi_sd)+gi_mean
zthresh_lower = (-zval*gi_sd)+gi_mean
gi_sd
```

```
## [1] 0.0781284
```

```
gi_mean
```

```
## [1] 0.003516402
```

```
zthresh_upper
```

```
## [1] 0.1597732
```

```
zthresh_lower
```

```
## [1] -0.1527404
```

```
#Record which bioprocess each query guide belongs to
proteosome = c("PRE7g7","PRE4g9","PRE4g3","RPN5g1")
secretory = c("COG3g1","SED5g5","SEC22g1","SEC22g2","COG8g2","GET2g2")
small_ribo = c("IMP4g6","DIP2g5","PWP2g2_BC1","PWP2g2_BC2")
large_ribo = c("TIF6g8","RPF1g3","MAK16g1")
misc = c("YCR016Wg4","YLR050Cg1","SAP30g7")
```

Supp Fig 1 All GFP analysis is in a separate RMarkdown file

Supp fig 2A-B Visualizing count data for each strain at T0 (Prior to start of pooled fitness assay)

```
#read in count data normalized to remove chimeric PCR products
load("~/Desktop/SherlockLab2/manuscript_aug2017/code/processing_data/Data_R_counts/seqlib11_chimera_normalized_counts.RData")

#record strain name, query guide name for each row
dat5_norm$strain = rownames(dat5_norm)
dat5_norm$query = gsub("-.+","",dat5_norm$strain)

#remove control guide sequences from analysis which had measured fitness defect in pilot screen
dat5_norm$array = gsub(".+-","",dat5_norm$strain)
rem_i = which(dat5_norm$array=="CC3"|dat5_norm$array=="CC33")
dat5_norm=dat5_norm[-rem_i,]

#plot distribution of coverage at T0
ggplot(dat5_norm)+geom_histogram(aes(x=Batch1T0),binwidth=5)+
  geom_vline(xintercept=222)+geom_vline(xintercept=543)+xlab("")+ylab("")
```

```
#plot coverage at T0 for each group of double CRISPRi strains carrying the same query guide
ggplot(dat5_norm,aes(x=query,y=Batch1T0,color=query))+
  geom_jitter(position = position_jitter(width = .2),size=1,alpha=0.4)+
  theme(axis.text.x=element_text(angle=70,hjust=1),legend.position="none")+
  geom_hline(yintercept=10)+xlab("")+ylab("")
```

```
#print summary stats listed in manuscript text
length(which(dat5_norm$Batch1T0<10)) #number of strains undetectable in starting pool (coverage less than 10)
```

```
## [1] 14
```

```
14/17069*100 #percent strains undetectable in starting strains
```

```
## [1] 0.08202004
```

```
summary(dat5_norm$Batch1T0) #quartiles of starting coverage for all strains in pool
```

```
##      Min.   1st Qu.    Median      Mean   3rd Qu.      Max. 
##   -0.1784  222.6000  347.5000  391.5000  543.0000 1357.0000
```

```
543/222 #fold difference between 1st and 3rd quartile
```

```
## [1] 2.445946
```

```
#print percent of strains missing fitness estimates in each condition
length(which(is.na(ypd24_all_dat$mean)))/dim(ypd24_all_dat)[1]*100
```

```
## [1] 0.5421651
```

```
length(which(is.na(ypd48_all_dat$mean)))/dim(ypd24_all_dat)[1]*100
```

```
## [1] 11.66834
```

```
length(which(is.na(ypeg_all_dat$mean)))/dim(ypd24_all_dat)[1]*100
```

```
## [1] 6.069892
```

```
length(which(is.na(ypd37_all_dat$mean)))/dim(ypd24_all_dat)[1]*100
```

```
## [1] 1.714892
```

```
length(which(is.na(ura_all_dat$mean)))/dim(ypd24_all_dat)[1]*100
```

```
## [1] 0.7307443
```

```
#print percent of strains with estimates in all 5 conditions
temp = data.frame(ypd24_all_dat$mean,
                  ypd48_all_dat$mean,
                  ypeg_all_dat$mean,
                  ypd37_all_dat$mean,
                  ura_all_dat$mean)
temp$no_miss=apply(temp,1,function(x)length(which(is.na(x))))
100-length(which(temp$no_miss>0))/dim(temp)[1]*100
```

```
## [1] 85.21421
```

```
rm(dat5_norm)
rm(rem_i)
```

Supp Fig 3 Plots rendered in R markdown file used to calculate fitness and interaction score estimates

Supp fig 4A Fitness across replicate single mutant strains

```
#function to take data from one condition and make plot of single mutant fitness for replicate strains in reverse orientations
plot_singles = function(data){
  all_dat = data.frame()
  
  #loop through guide names for replicate strains and add information to data table
  for(i in 1:13){
    #get information for single mutants of query guide
    q_temp = subset(data,query==rep_query_names[i]&category=="single")[,c(32,33,35)]
    q_temp$type =  "query"
    q_temp$guide = q_temp$query
    
    #get information for single mutants array guide
    a_temp = subset(data,array==rep_array_names[i]&category=="single")[,c(32,33,35)]
    a_temp$type="array"
    a_temp$guide = rep_query_names[i]
    
    #combine information
    all_dat = rbind(all_dat,q_temp,a_temp)
  }  
  
  #edit guide name of PWPg2_BC1 to remove "BC1"
  all_dat$guide[which(all_dat$guide=="PWP2g2_BC1")]="PWP2g2"
  
  #add barcode replicate of PWP2g2 to data table
  all_dat$BC = 1
  temp = subset(data,query==rep_query_names[14]&category=="single")[,c(32,33,35)]
  temp$type="query"
  temp$guide = "PWP2g2"
  temp$BC = 2
  all_dat = rbind(all_dat,temp)
  
  #Make all pairwise comparisons of single mutants with guides targeting the same gene
  compare_dat = data.frame(matrix(ncol=5))
  names(compare_dat) = c("guide","strain1","strain2","mean1","mean2")
  count = 1
  for(name in unique(all_dat$guide)){
    query_i = which(all_dat$type=="query"&all_dat$guide==name)
    array_i = which(all_dat$type=="array"&all_dat$guide==name)
    
    for(i in query_i){
      for(j in array_i){
        compare_dat[count,1:3]=c(name,rownames(all_dat)[i],rownames(all_dat)[j])
        compare_dat[count,4:5]=c(all_dat$mean[i],all_dat$mean[j])
        count = count + 1
      }
    }
  }
  return(compare_dat)
}
all_singles = rbind(plot_singles(ypd24_all_dat),
                    plot_singles(ypd48_all_dat),
                    plot_singles(ypeg_all_dat),
                    plot_singles(ypd37_all_dat),
                    plot_singles(ura_all_dat))

#plot data
ggplot(all_singles,aes(x=mean1,y=mean2))+geom_point(size=5,alpha=0.2,color="black")+
  geom_point(shape=1,size=5,color="black",alpha=0.4)+geom_abline(color="red",size=1.5)+
  xlim(-0.4,1.2)+ylim(-0.4,1.2)+xlab("Strain 1")+ylab("Strain 2")
```

```
## Warning: Removed 2 rows containing missing values (geom_point).
```

```
## Warning: Removed 2 rows containing missing values (geom_point).
```

```
cor.test(all_singles$mean1,all_singles$mean2,method="spearman")
```

```
## Warning in cor.test.default(all_singles$mean1, all_singles$mean2, method =
## "spearman"): Cannot compute exact p-value with ties
```

```
## 
##  Spearman's rank correlation rho
## 
## data:  all_singles$mean1 and all_singles$mean2
## S = 38573780, p-value < 2.2e-16
## alternative hypothesis: true rho is not equal to 0
## sample estimates:
##       rho 
## 0.9188264
```

```
#Print summary statistics listed in manuscript text
#compute SD across replicate strains for replicate query single mutant strains
sds = NA
count = 1
for(name in unique(ypd24_all_dat$query)[1:20]){
  sds[count:(count+4)] = c(sd(subset(ypd24_all_dat,query==name&category=="single")$mean,na.rm=TRUE),
                           sd(subset(ypd48_all_dat,query==name&category=="single")$mean,na.rm=TRUE),
                           sd(subset(ypeg_all_dat,query==name&category=="single")$mean,na.rm=TRUE),
                           sd(subset(ypd37_all_dat,query==name&category=="single")$mean,na.rm=TRUE),
                           sd(subset(ura_all_dat,query==name&category=="single")$mean,na.rm=TRUE))
  count = count + 5
  }

print(median(sds,na.rm=TRUE))
```

```
## [1] 0.03435423
```

Supp Fig 4B Comparison of single mutant fitness in this study versus Smith et al Mol Sys Biol 2017

```
#YPD comparison
tecan = read.table(file="~/Desktop/SherlockLab2/manuscript_aug2017/code/figures/supp_data/smith2017_rawdata/YPD_DHS_all_BiDir200_annotated_DESeq2.txt",stringsAsFactors = FALSE,header=TRUE,sep="\t",quote="")

#subset published data to only those guides measured in this study
dat = tecan[which(tecan$guide_id%in%unique(ypd24_all_dat$array)),c(1,15:16,31)]

#record fitness values for two replicate strains in this study
dat$cc16.1 = NA
dat$cc17.1 = NA
for(i in 1:dim(dat)[1]){
  temp = subset(ypd24_all_dat,array==dat$guide_id[i]&category=="single")
  row.cc16 = which(temp$query=="CC16")
  dat[i,5] = temp$mean[row.cc16]
  row.cc17 = which(temp$query=="CC17")
  dat[i,6] = temp$mean[row.cc17]
}

#plot results
ggplot(dat)+geom_point(aes(x=cc16.1,y=log2FoldChange),size=4,alpha=0.6)+
  geom_point(aes(x=cc17.1,y=log2FoldChange),size=4,alpha=0.6)+
  geom_point(aes(x=cc16.1,y=log2FoldChange),size=4,shape=1)+
  geom_point(aes(x=cc17.1,y=log2FoldChange),size=4,shape=1)+
  xlab("double CRISPRi fitness in YPD")+ylab("log2(fold change) in YPD")+ggtitle("YPD")
```

```
## Warning: Removed 1 rows containing missing values (geom_point).
```

```
## Warning: Removed 1 rows containing missing values (geom_point).
```

```
cor.test(c(dat$log2FoldChange,dat$log2FoldChange),c(dat$cc16.1,dat$cc17.1),method="spearman")
```

```
## Warning in cor.test.default(c(dat$log2FoldChange, dat$log2FoldChange),
## c(dat$cc16.1, : Cannot compute exact p-value with ties
```

```
## 
##  Spearman's rank correlation rho
## 
## data:  c(dat$log2FoldChange, dat$log2FoldChange) and c(dat$cc16.1, dat$cc17.1)
## S = 273834690, p-value < 2.2e-16
## alternative hypothesis: true rho is not equal to 0
## sample estimates:
##       rho 
## 0.5312227
```

```
rm(tecan)
rm(dat)

#Do same analysis for respiratory growth condition (YPEG)
tecan = read.table(file="~/Desktop/SherlockLab2/manuscript_aug2017/code/figures/supp_data/smith2017_rawdata/YPEG_DHS_all_BiDir200_annotated_DESeq2.txt",stringsAsFactors = FALSE,header=TRUE,sep="\t",quote="")

#subset published to those guides measured here
dat = tecan[which(tecan$guide_id%in%unique(ypeg_all_dat$array)),c(1,15:16,31)]

#record fitness from this study
dat$cc16.1 = NA
dat$cc17.1 = NA
for(i in 1:dim(dat)[1]){
  temp = subset(ypeg_all_dat,array==dat$guide_id[i]&category=="single")
  row.cc16 = which(temp$query=="CC16")
  dat[i,5] = temp$mean[row.cc16]
  row.cc17 = which(temp$query=="CC17")
  dat[i,6] = temp$mean[row.cc17]
}

#plot results
ggplot(dat)+geom_point(aes(x=cc16.1,y=log2FoldChange),size=4,alpha=0.6)+
  geom_point(aes(x=cc17.1,y=log2FoldChange),size=4,alpha=0.6)+
  geom_point(aes(x=cc16.1,y=log2FoldChange),size=4,shape=1)+
  geom_point(aes(x=cc17.1,y=log2FoldChange),size=4,shape=1)+
  xlab("double CRISPRi fitness in YPEG")+ylab("log2(fold change) in YPEG")+ggtitle("YPEG")
```

```
## Warning: Removed 4 rows containing missing values (geom_point).
```

```
## Warning: Removed 5 rows containing missing values (geom_point).
```

```
## Warning: Removed 4 rows containing missing values (geom_point).
```

```
## Warning: Removed 5 rows containing missing values (geom_point).
```

```
cor.test(c(dat$log2FoldChange,dat$log2FoldChange),c(dat$cc16.1,dat$cc17.1),method="spearman")
```

```
## Warning in cor.test.default(c(dat$log2FoldChange, dat$log2FoldChange),
## c(dat$cc16.1, : Cannot compute exact p-value with ties
```

```
## 
##  Spearman's rank correlation rho
## 
## data:  c(dat$log2FoldChange, dat$log2FoldChange) and c(dat$cc16.1, dat$cc17.1)
## S = 181802573, p-value < 2.2e-16
## alternative hypothesis: true rho is not equal to 0
## sample estimates:
##       rho 
## 0.6838028
```

```
rm(tecan)
rm(dat)
```

Supp Fig 4C Fitness of double mutant carrying two gRNAs with same sequence compared to each corresponding single mutant

```
#function to extract fitness data from a given condition's dataframe
plot_two_same = function(data,condition){
  
  #extract single and double mutant info from strains carrying two of the same guide sequence
  same_guides = data.frame(matrix(nrow=13,ncol=4))
  names(same_guides)=c("guide","qmean","amean","dmean")
  for(i in 1:13){
    same_guides$guide[i]=rep_query_names[i] #record guide name
    sm_query = subset(data,query==rep_query_names[i]&category=="single") #get single mutant fitness 1
    same_guides$qmean[i] = mean(sm_query$mean,na.rm=TRUE) #record single mutant fitness 1
    sm_array = subset(data,array==rep_array_names[i]&category=="single")#get single mutant fitness 2
    same_guides$amean[i] = mean(sm_array$mean,na.rm=TRUE) #record single mutant fitness 2
    dm = subset(data,query==rep_query_names[i]&array==rep_array_names[i]) #get double mutant fitness
    same_guides$dmean[i]=dm$mean[1] #record double mutant fitness
  }
  
  same_guides$condition=condition #record condition
  return(same_guides)
  }

#call and record results from function for each condition
all_same = rbind(plot_two_same(ypd24_all_dat,"YPD24hr"),
                 plot_two_same(ypd48_all_dat,"YPD48hr"),
                 plot_two_same(ypeg_all_dat,"YPEG"),
                 plot_two_same(ypd37_all_dat,"YPD37C"),
                 plot_two_same(ura_all_dat,"SCURA"))
                 
#plot results
print(ggplot(all_same,aes(x=qmean,y=dmean))+geom_abline(color="red",size=1.5)+
  geom_point(size=8,alpha=0.4,color="black")+
  geom_point(shape=1,size=8,color="black",alpha=0.6)+
  xlim(-0.1,1.3)+ylim(-0.1,1.3)+xlab("Single Mutant (Query)")+ylab("Double Mutant"))
```

```
print(ggplot(all_same,aes(x=amean,y=dmean))+geom_abline(color="red",size=1.5)+
  geom_point(size=8,alpha=0.4,color="black")+
  geom_point(shape=1,size=8,color="black",alpha=0.6)+
  xlim(-0.1,1.3)+ylim(-0.1,1.3)+xlab("Single Mutant (Array)")+ylab("Double Mutant"))
```

```
#print summary stats listed in manuscript text
print(length(which(all_same$amean<all_same$dmean))) #cases where single mutant 1 fitness is lower than double mutant
```

```
## [1] 3
```

```
print(length(which(all_same$qmean<all_same$dmean))) #cases where single mutant 2 fitness is lower than double mutant
```

```
## [1] 1
```

```
print(length(all_same$qmean)+length(all_same$dmean)) #total number of comparisons
```

```
## [1] 130
```

```
(130-4)/130*100
```

```
## [1] 96.92308
```

Supp Fig 5A-E Estimating GI score from fitness For each condition, and each query guide, plot observed double mutant fitness against single mutant fitness of guide derived from starting pool

```
psize=2
lsize=1.5

p=ggplot(subset(ypd24_all_dat,category=="double"),aes(x=array_mean,y=mean))+geom_point(alpha=0.3,size=psize)+
  geom_abline(aes(slope=query_mean),color="red",size=lsize)+xlim(-0.4,1.2)+ylim(-0.5,1.5)+
  ggtitle("YPD24hr")+geom_smooth(method="lm",size=lsize)+xlab("")+ylab("")
p+facet_wrap(~query)
```

```
p=ggplot(subset(ypd48_all_dat,category=="double"),aes(x=array_mean,y=mean))+geom_point(alpha=0.3,size=psize)+
  geom_abline(aes(slope=query_mean),color="red",size=lsize)+xlim(-0.4,1.2)+ylim(-0.5,1.5)+
  ggtitle("YPD48hr")+geom_smooth(method="lm",size=lsize)+xlab("")+ylab("")
p+facet_wrap(~query)
```

```
p=ggplot(subset(ypeg_all_dat,category=="double"),aes(x=array_mean,y=mean))+geom_point(alpha=0.3,size=psize)+
  geom_abline(aes(slope=query_mean),color="red",size=lsize)+xlim(-0.4,1.2)+ylim(-0.5,1.5)+
  ggtitle("YPEG")+geom_smooth(method="lm",size=lsize)+xlab("")+ylab("")
p+facet_wrap(~query)
```

```
p=ggplot(subset(ypd37_all_dat,category=="double"),aes(x=array_mean,y=mean))+geom_point(alpha=0.3,size=psize)+
  geom_abline(aes(slope=query_mean),color="red",size=lsize)+xlim(-0.4,1.2)+ylim(-0.5,1.5)+
  ggtitle("YPD37C")+geom_smooth(method="lm",size=lsize)+xlab("")+ylab("")
p+facet_wrap(~query)
```

```
p=ggplot(subset(ura_all_dat,category=="double"),aes(x=array_mean,y=mean))+geom_point(alpha=0.3,size=psize)+
  geom_abline(aes(slope=query_mean),color="red",size=lsize)+xlim(-0.4,1.2)+ylim(-0.5,1.5)+
  ggtitle("SC-URA")+geom_smooth(method="lm",size=lsize)+xlab("")+ylab("")
p+facet_wrap(~query)
```

Supp Fig 5F GI score reproducibility in pairs of replicate strains carrying the same gRNAs in reverse orientation

```
#get data for all pairwise combos of strains carrying the same pair of guides
plot_rev_double = function(data,condition){
  all_dat = data.frame(matrix(nrow=78,ncol=4))
  names(all_dat) = c("guide1","guide2","gi1","gi2")
  
  count = 0
  for(i in 1:12){
    for(j in (i+1):13){
      count = count + 1
      all_dat[count,1:2] = c(rep_query_names[i],rep_query_names[j])
      all_dat[count,3:4]=c(subset(data,query==rep_query_names[i]&array==rep_array_names[j])[,9],
                           subset(data,query==rep_query_names[j]&array==rep_array_names[i])[,9])
    }
  }
  all_dat$condition = condition
  return(all_dat)
}

#call function and combine data for all 5 conditions
all_rev = rbind(plot_rev_double(ypd24_doubles,"YPD24hr"),
                plot_rev_double(ypd48_doubles,"YPD48hr"),
                plot_rev_double(ypeg_doubles,"YPEG"),
                plot_rev_double(ypd37_doubles,"YPD37C"),
                plot_rev_double(ura_doubles,"SCURA"))

#plot results
ggplot(all_rev,aes(x=gi1,y=gi2))+geom_point(alpha=0.5,size=4,aes(color=condition))+
  geom_abline()+annotate("rect",xmin=zthresh_lower,xmax=zthresh_upper,ymin=zthresh_lower,ymax=zthresh_upper,alpha=0.3)
```

```
## Warning: Removed 25 rows containing missing values (geom_point).
```

```
cor.test(all_rev$gi1,all_rev$gi2,method="spearman")
```

```
## 
##  Spearman's rank correlation rho
## 
## data:  all_rev$gi1 and all_rev$gi2
## S = 4972602, p-value = 2.105e-14
## alternative hypothesis: true rho is not equal to 0
## sample estimates:
##       rho 
## 0.3864364
```

Supp Fig 6A GI score comparisons across conditions

```
#generate dataframe carrying gi scores in each condition for each guide pair
temp=data.frame(ypd24_doubles$gi_score,
                ypd48_doubles$gi_score,
                ypeg_doubles$gi_score,
                ypd37_doubles$gi_score,
                ura_doubles$gi_score)
names(temp)=c("YPD24hr","YPD48hr","YPEG","YPD37","SCURA")

#loop through each possible pair of conditions and plot correlation of gi_scores
for(i in 1:4){
  for(j in (i+1):5){
    print(ggplot(temp)+geom_point(aes(x=temp[,i],y=temp[,j]),alpha=0.5,size=psize,color="grey19")+
      geom_point(aes(x=temp[,i],y=temp[,j]),shape=1,size=psize)+
      annotate("rect",xmin=zthresh_lower,xmax=zthresh_upper,
               ymin=zthresh_lower,ymax=zthresh_upper,alpha=0.8)+
      geom_abline(color="black",size=2)+ylab(names(temp)[j])+
      xlab(names(temp)[i])+xlim(-0.75,1.25)+ylim(-0.75,1.25)+
      geom_vline(xintercept=zthresh_lower,linetype="dashed",size=1.5)+
      geom_vline(xintercept=zthresh_upper,linetype="dashed",size=1.5)+
      geom_hline(yintercept=zthresh_lower,linetype="dashed",size=1.5)+
      geom_hline(yintercept=zthresh_upper,linetype="dashed",size=1.5))
    }
}
```

```
## Warning: Removed 1962 rows containing missing values (geom_point).
```

```
## Warning: Removed 1962 rows containing missing values (geom_point).
```

```
## Warning: Removed 1034 rows containing missing values (geom_point).
```

```
## Warning: Removed 1034 rows containing missing values (geom_point).
```

```
## Warning: Removed 304 rows containing missing values (geom_point).
```

```
## Warning: Removed 304 rows containing missing values (geom_point).
```

```
## Warning: Removed 149 rows containing missing values (geom_point).
```

```
## Warning: Removed 149 rows containing missing values (geom_point).
```

```
## Warning: Removed 2440 rows containing missing values (geom_point).
```

```
## Warning: Removed 2440 rows containing missing values (geom_point).
```

```
## Warning: Removed 2052 rows containing missing values (geom_point).
```

```
## Warning: Removed 2052 rows containing missing values (geom_point).
```

```
## Warning: Removed 1974 rows containing missing values (geom_point).
```

```
## Warning: Removed 1974 rows containing missing values (geom_point).
```

```
## Warning: Removed 1091 rows containing missing values (geom_point).
```

```
## Warning: Removed 1091 rows containing missing values (geom_point).
```

```
## Warning: Removed 1041 rows containing missing values (geom_point).
```

```
## Warning: Removed 1041 rows containing missing values (geom_point).
```

```
## Warning: Removed 310 rows containing missing values (geom_point).
```

```
## Warning: Removed 310 rows containing missing values (geom_point).
```

```
#print key diagram (no data) for figure legend
ggplot()+
  xlim(-0.75,1.25)+ylim(-0.75,1.25)+
  geom_vline(xintercept=zthresh_lower,linetype="dashed",size=1.5)+
  geom_vline(xintercept=zthresh_upper,linetype="dashed",size=1.5)+
  geom_hline(yintercept=zthresh_lower,linetype="dashed",size=1.5)+
  geom_hline(yintercept=zthresh_upper,linetype="dashed",size=1.5)+
  annotate("rect",xmin=zthresh_lower,xmax=zthresh_upper,ymin=zthresh_lower,ymax=zthresh_upper,alpha=0.8)+
  annotate("rect",xmin=zthresh_upper,xmax=1.25,ymin=zthresh_upper,ymax=1.25,alpha=0.4,fill="red")+
  annotate("rect",xmax=zthresh_lower,xmin=-0.75,ymax=zthresh_lower,ymin=-0.75,alpha=0.4,fill="red")+
  annotate("rect",xmax=zthresh_lower,xmin=-0.75,ymin=zthresh_upper,ymax=1.25,alpha=0.4,fill="blue")+
  annotate("rect",xmin=zthresh_upper,xmax=1.25,ymax=zthresh_lower,ymin=-0.75,alpha=0.4,fill="blue")+
  annotate("rect",xmin=zthresh_lower,xmax=zthresh_upper,ymin=zthresh_upper,ymax=1.25,alpha=0.4,fill="orange")+
  annotate("rect",xmin=zthresh_lower,xmax=zthresh_upper,ymax=zthresh_lower,ymin=-0.75,alpha=0.4,fill="orange")+
  annotate("rect",xmin=-0.75,xmax=zthresh_lower,ymin=zthresh_lower,ymax=zthresh_upper,alpha=0.4,fill="green")+
  annotate("rect",xmin=zthresh_upper,xmax=1.25,ymin=zthresh_lower,ymax=zthresh_upper,alpha=0.4,fill="green")+
  geom_abline(color="black",size=2)
```

```
#print summary stats mentioned in manuscript text
print(cor.test(temp$YPD24hr,temp$YPEG)) #correlations
```

```
## 
##  Pearson's product-moment correlation
## 
## data:  temp$YPD24hr and temp$YPEG
## t = 73.0397, df = 14164, p-value < 2.2e-16
## alternative hypothesis: true correlation is not equal to 0
## 95 percent confidence interval:
##  0.5109984 0.5349245
## sample estimates:
##       cor 
## 0.5230645
```

```
print(cor.test(temp$YPD48hr,temp$YPEG))
```

```
## 
##  Pearson's product-moment correlation
## 
## data:  temp$YPD48hr and temp$YPEG
## t = 80.9286, df = 12758, p-value < 2.2e-16
## alternative hypothesis: true correlation is not equal to 0
## 95 percent confidence interval:
##  0.5708421 0.5937752
## sample estimates:
##       cor 
## 0.5824245
```

Supp Fig 6B Code for rendering heatmap can be found in Figure 2 R markdown file

Supp fig 7 Query guide GI profile comparisons

```
#function to re-format data (rows as starting pool guides and columns as query guides)
get_gi_mat3=function(data){
  gi_mat = data.frame(matrix(nrow=760,ncol=20))
  queries = unique(data$query)
  names(gi_mat)=queries
  for(i in 1:20){
    gi_mat[,i]=subset(data,query==names(gi_mat)[i])$gi_score
  }
  rownames(gi_mat)=subset(data,query=="SAP30g7")$array
  return(gi_mat)
}

#concatenate data across all five growth conditions
gi_dat = rbind(get_gi_mat3(ypd24_doubles),
               get_gi_mat3(ypd48_doubles),
               get_gi_mat3(ypeg_doubles),
               get_gi_mat3(ypd37_doubles),
               get_gi_mat3(ura_doubles))

#perform hierarchical clustering and plot dendrogram
coldist=as.dist(1-cor(gi_dat,use="pairwise.complete.obs"))
colclust = hclust(coldist,method="average")
colclust.d = as.dendrogram(colclust)
colorder=colclust$labels[colclust$order]
par(mar=c(10,4,4,2))
plot(colclust.d,edgePar=list(lwd=6))
```

```
par(mar=c(5,4,4,2))
```

```
#prepare colorbar for dendrogram
plot_color_bar=function(bio_colorder,condition){
  bio_colorder[which(bio_colorder%in%proteosome)] = "proteosome"
  bio_colorder[which(bio_colorder%in%secretory)]="secretory"
  bio_colorder[which(bio_colorder%in%small_ribo)]="small_ribo"
  bio_colorder[which(bio_colorder%in%large_ribo)]="large_ribo"
  bio_colorder[which(bio_colorder%in%misc)]="misc"
  return(bio_colorder)
}
temp=plot_color_bar(colorder,"all conditions")
print(ggplot()+geom_tile(aes(x=1:20,y=rep(1,20),fill=temp))+
    scale_fill_brewer(palette="Accent")+ggtitle("all conditions"))
```

```
#prepare heatmap showing correlations of gi profiles
gi_dat = gi_dat[,match(colorder,names(gi_dat))]
cor_dat = as.data.frame(cor(gi_dat,use="pairwise.complete.obs"))
cor_dat$query = rownames(cor_dat)
cor_dat$query = factor(rownames(cor_dat),levels = rownames(cor_dat))
print(qplot(x=query, y=variable, data=melt(cor_dat[,1:21],id.vars="query"),
            fill=value,geom="tile")+
        scale_fill_gradient2(limits=c(-1,1),low="mediumpurple4",high="orangered1")+
        geom_text(aes(label=round(value,digits=2)))+
        theme(axis.text.x=element_text(angle=50,hjust=1))+xlab("")+ylab(""))
```

```
#plot correlation values for pairs of guides targeting the same bioprocess vs different

cor_dat_l = cor_dat #initialize variable to store correlation values for all unique pairs of query guides
#first fill in half of the correlation matrix with -100 in order to later remove duplicate comparisons
for(i in 1:20){
  for(j in i:20){
    cor_dat_l[i,j]=-100
  }
}
cor_dat_l = melt(cor_dat_l) #reformat to long format
```

```
## Using query as id variables
```

```
cor_dat_l = cor_dat_l[-which(cor_dat_l$value==-100),] #remove duplicate values (noted by -100 above)

#record whether the pair targets the same bioprocess
cor_dat_l$target = "different"
for(i in 1:dim(cor_dat_l)[1]){
  if(cor_dat_l$query[i]%in%proteosome&cor_dat_l$variable[i]%in%proteosome){cor_dat_l$target[i]="proteosome"}
  if(cor_dat_l$query[i]%in%secretory&cor_dat_l$variable[i]%in%secretory){cor_dat_l$target[i]="secretory"}
  if(cor_dat_l$query[i]%in%small_ribo&cor_dat_l$variable[i]%in%small_ribo){cor_dat_l$target[i]="small_ribo"}
  if(cor_dat_l$query[i]%in%large_ribo&cor_dat_l$variable[i]%in%large_ribo){cor_dat_l$target[i]="large_ribo"}
}
#note guide pairs targeting the same gene
cor_dat_l$target[which(cor_dat_l$query=="PRE4g9"&cor_dat_l$variable=="PRE4g3")]="same_gene"
cor_dat_l$target[which(cor_dat_l$query=="SEC22g2"&cor_dat_l$variable=="SEC22g1")]="same_gene"
cor_dat_l$target[which(cor_dat_l$query=="PWP2g2_BC2"&cor_dat_l$variable=="PWP2g2_BC1")]="same_gene"

cor_dat_l$target2 = cor_dat_l$target
cor_dat_l$target2[which(cor_dat_l$target2%in%c("proteosome","secretory","small_ribo","large_ribo"))]="same_process"

#plot pairs targeting the different versus the same bioprocesses
cor_dat_l$xval = 0
cor_dat_l$xval[which(cor_dat_l$target2=="different")]=1
cor_dat_l$xval[which(cor_dat_l$target2=="same_process")]=2
set.seed(8723)
cor_dat_l$xval = cor_dat_l$xval + rnorm(length(cor_dat_l$xval),mean=0,sd=0.05)
cor_dat_l$target = factor(cor_dat_l$target,levels=c("large_ribo","different","proteosome","secretory","small_ribo"))
ggplot(subset(cor_dat_l,target2!="same_gene"),aes(x=xval,y=value))+
  geom_point(size=5,alpha=0.8,aes(color=target))+scale_color_brewer(palette="Accent")+
  geom_point(size=5,shape=1)+xlim(0.5,2.5)
```

```
#compare groups with wilcoxon rank sum test
wilcox.test(subset(cor_dat_l,target2=="same_process")$value,subset(cor_dat_l,target2=="different")$value)
```

```
## 
##  Wilcoxon rank sum test with continuity correction
## 
## data:  subset(cor_dat_l, target2 == "same_process")$value and subset(cor_dat_l, target2 == "different")$value
## W = 3698, p-value = 3.422e-09
## alternative hypothesis: true location shift is not equal to 0
```

```
#print summary stats used in manuscript
print(subset(cor_dat_l,target2=="same_gene"))
```

```
##          query   variable     value target   target2        xval
## 65  PWP2g2_BC2 PWP2g2_BC1 0.5353186   <NA> same_gene -0.04132417
## 191     PRE4g9     PRE4g3 0.2590623   <NA> same_gene  0.00452301
## 296    SEC22g2    SEC22g1 0.7118553   <NA> same_gene  0.01080438
```

```
print(quantile(subset(cor_dat_l,target2!="same_gene")$value,0.74))
```

```
##       74% 
## 0.2547835
```

```
cor_dat_same_proc = subset(cor_dat_l,target2=="same_process")

#Comparison of GI profiles of YCR016W and COG8
#get lists of significant positive interactions for both query and list of shared
ycr_pos=subset(ypeg_doubles,query=="YCR016Wg4"&gi_score>zthresh_upper)$array
cog_pos=subset(ypeg_doubles,query=="COG8g2"&gi_score>zthresh_upper)$array
shared_pos = intersect(ycr_pos,cog_pos)

#load array GO annotations
array_go = read.table(file="~/Desktop/SherlockLab2/manuscript_aug2017/code/figures/GO_lists.txt",sep="\t",header=TRUE,stringsAsFactors = FALSE)

#get names of genes involved in signaling
signaling_genes = unlist(strsplit(array_go[which(array_go[,1]=="signaling"),4],split=","))

#get gene names for guides showing positive interaction with both COG8 and YCR016W
shared_pos_genes = gsub("-.+","",shared_pos)

#get names of genes targeted by all guides
array_genes = gsub("-.+","",ypd24_doubles$array[1:760])

#perform hypergeometric enrichment test for signaling annotation in the list of overlapping interactions
q = length(which(shared_pos_genes%in%signaling_genes)) #number of guides matching term in list of significant GIs
m = length(which(array_genes%in%signaling_genes)) #number of guides in background list annotated with term
n = 760 - m #number of guides in background list not annotated with term
k = length(shared_pos_genes) #length of list testing
print(phyper(q-1,m,n,k,lower.tail=FALSE))
```

```
## [1] 4.747867e-05
```

Supp fig 8 and 9 Expanded ROC analysis See markdown for Figure 3

Supp fig 10 Results from OD-based fitness assay for GI validation

```
#load in data from tecan and sample name key
dat = read.table(file="~/Desktop/SherlockLab2/manuscript_aug2017/code/figures/supp_data/tecan_15082017/15082017.csv",sep=",",header=FALSE,stringsAsFactors = FALSE)
samples = read.table(file="~/Desktop/SherlockLab2/manuscript_aug2017/code/figures/supp_data/tecan_15082017/samples.csv",sep=",",header=FALSE,stringsAsFactors = FALSE)

#format and clean up data
names(dat)[2:97] = samples$V7 #add sample sames
dat$V1 = as.numeric(gsub("s","",dat$V1)) #convert time stamps to integers
dat=dat[,-c(26:31,38:43,50:97)]#remove empty wells
dat = dat[-c(1:5),]#remove first 5 time points
#convert to long format and record sample name, dose and replicate id
dat_l = melt(dat,id.vars="V1") 
dat_l$sample = NA
dat_l$dose = NA
dat_l$rep = NA
for(i in 1:dim(dat_l)[1]){
  temp = unlist(strsplit(as.character(dat_l[i,2]),split="-"))
  dat_l$strain[i] = temp[1]
  dat_l$dose[i] = temp[2]
  dat_l$rep[i] = temp[3]
}
dat_l$sample = gsub("-r.+","",dat_l$variable)
```

Quality Control Step: Visualize raw OD growth curves for YPD-ATC to YPD+ATC for each strain

```
temp = subset(dat_l,strain%in%c("wt","rpn5","lsm2","lsm2_rpn5"))
temp$strain = factor(temp$strain,levels=c("wt","rpn5","lsm2","lsm2_rpn5"))
p=ggplot(temp,aes(x=V1,y=value))+geom_line(aes(color=dose,group=variable),alpha=0.5,size=1.5)+
  scale_x_continuous(breaks = seq(from=0,to=162000,by=18000),
                     labels=seq(from=0,to=45,by=5))
p+facet_wrap(~strain)
```

```
temp = subset(dat_l,strain%in%c("wt","rpn5","lsm4","lsm4_rpn5"))
temp$strain = factor(temp$strain,levels=c("wt","rpn5","lsm4","lsm4_rpn5"))
p=ggplot(temp,aes(x=V1,y=value))+geom_line(aes(color=dose,group=variable),alpha=0.5,size=1.5)+
  scale_x_continuous(breaks = seq(from=0,to=162000,by=18000),
                     labels=seq(from=0,to=45,by=5))
p+facet_wrap(~strain)
```

Calculate maximum doubling time using window tiling method (code from Jaffe et al, G3, 2017 analysis, which was based on St. Onge. Nat. Genet., 2007) \* Doubling time D = (Tf - Ti)/2 \* Time final (at abitrary maximum ODmax), Tf = (ODmax - b)/m \* m and b, are given from the linear regression fitting of the data \* Time initial (2 generations earlier at OD(max-2)), Ti = (OD(max-2)-b)m \* Will use 0.95 for ODmax, 0.2375 for OD(max-2), and the values of m and b from the linear regression fitting of the points between 0.35 and 0.95 \* Fitness is then calculated by comparing Doubling time of each strain to the WT

```
#do this window calculation over first 92 time points (24hrs)
dat2 = dat[1:92,]

#loop through each sample and look at each 10 time point window and record maximum slope
time=dat2$V1[1:92]
log_reg_data = data.frame(matrix(ncol=36,nrow=3))#36 columns because there are 36 samples
names(log_reg_data)=names(dat2[2:37])#skip first column, because this is not a sample, it is the time values
for (col in c(2:37)){#loop through each sample
  max_m = 0
  end = length(time) - 9
  for (start_index in c(1:end)){#loop through each 10 time point window
    end_index = start_index + 9
    sub_OD = log2(dat2[start_index:end_index,col])
    sub_time = time[start_index:end_index]
    lm_result = coefficients(lm(sub_OD~sub_time))
    b = lm_result[1]
    m = lm_result[2]
    if (m > max_m){
      max_m = m
      max_b = b
      max_window = c(start_index:end_index)
    }
  }
  #print(max_m)
  log_reg_data[,(col-1)] = c(max_m,max_b,max_window[1])
}

#calculate doubling time for each strain
doublings_log = apply(log_reg_data, 2, function(x) (((log2(0.95)-x[2])/x[1])-(log2(0.2375)-x[2])/x[1])/2/60/60) 

#get data into correct form for faceted plotting
doublings_long = melt(doublings_log)
doublings_long$variable = names(doublings_log)
doublings_long$sample=gsub("-r.+","",doublings_long$variable)
doublings_long$strain = NA
doublings_long$dose = NA
doublings_long$rep = NA
for(i in 1:dim(doublings_long)[1]){
  temp = unlist(strsplit(as.character(doublings_long[i,2]),split="-"))
  doublings_long$strain[i] = temp[1]
  doublings_long$dose[i] = temp[2]
  doublings_long$rep[i] = temp[3]
}

#Quality control step:
#plot doubling time (from max slope method above) for each sample (treated and untreated)
db = ggplot(doublings_long)+geom_point(aes(x=dose, y=value,color=dose),size=5,alpha=0.5)+
  ylab("Doubling time (hr)")+xlab("")+theme(axis.text.x=element_blank(), axis.ticks.x=element_blank())
db + facet_wrap(~strain)
```

Calculate fitness for each genotype by comparing doubling times in the presence vs absence of inducing drug

```
usamp = unique(doublings_long$sample[c(13:24,31:36)])
doubling_fit = data.frame(usamp)
names(doubling_fit)="sample"
doubling_fit$strain = gsub("-.+","",doubling_fit$sample)
doubling_fit$dose = gsub(".+-","",doubling_fit$sample)
doubling_fit$mean = NA
doubling_fit$sd = NA
doubling_fit=cbind(doubling_fit,data.frame(matrix(nrow=6,ncol=9)))
for(i in 1:length(usamp)){
  
  #get doubling times for all treated samples for this genotype
  temp = doublings_long[which(doublings_long$sample==usamp[i]),]
  treat_samp = temp$value
  
  #get doubling times for all untreated samples for this genotype
  control_samp = doublings_long$value[which(doublings_long$dose=="no_atc"&doublings_long$strain==unique(temp$strain))]
  
  #loop through each pairwise combination of treated/control and record fitness
  values = NA
  count = 0
  for(j in 1:length(treat_samp)){
    for(k in 1:length(control_samp)){
      count = count+1
      values[count]=control_samp[k]/treat_samp[j]
    }
  }
  
  #record results
  doubling_fit$mean[i]=mean(values)
  doubling_fit$sd[i]=sd(values)
  doubling_fit[i,6:14]=values
}
doubling_fit$strain = factor(doubling_fit$strain,levels=c("wt","rpn5","lsm2","lsm4","lsm2_rpn5","lsm4_rpn5"))

#save n-values to be used for building 95% confidence intervals
no_strains = 3

#function to plot fitness for 4 strains required to infer a GI between a gene pair
plot_pair=function(gene1,gene2,gene3,adjustment){
  row_i = which(doubling_fit$strain==gene1)
  row_j = which(doubling_fit$strain==gene2)
  
  #calculate expected double mutant fitness based on 95% CI of all pairwise products of single mutant fitness
  expected = NA
  count = 0
  for(i in 6:14){
    for(j in 6:14){
      count = count + 1
      expected[count]=doubling_fit[row_i,i]*doubling_fit[row_j,j]
    }
  }
  temp = subset(doubling_fit,strain%in%c("wt",gene1,gene2,gene3))
  
  
  #get 95% CI around mean
  temp$ymin = temp$mean-1.96*(temp$sd/sqrt(no_strains))
  temp$ymax = temp$mean+1.96*(temp$sd/sqrt(no_strains))
  
  print(temp)
  
  ylimits = aes(ymin=ymin,ymax=ymax)
  print(ggplot(temp,aes(x=strain,y=mean))+
          geom_bar(aes(fill=strain),alpha=0.7,color="black",stat="identity")+
          geom_errorbar(ylimits,width=0.5,size=1)+scale_y_continuous(breaks=c(0.25,0.5,0.75,1),limits=c(0,1.1))+
          annotate("rect",xmin=as.numeric(temp$strain[[1]])-0.6,
                   xmax=as.numeric(temp$strain[[4]])+adjustment,
                   ymax=(mean(expected)+1.96*(sd(expected)/sqrt(no_strains))),
                   ymin=(mean(expected)-1.96*(sd(expected)/sqrt(no_strains))),alpha=0.7)+
          geom_hline(yintercept=mean(expected),size=2)+
          geom_hline(yintercept=(mean(expected)+1.96*(sd(expected)/sqrt(no_strains))),linetype="dashed")+
          geom_hline(yintercept=(mean(expected)-1.96*(sd(expected)/sqrt(no_strains))),linetype="dashed"))

  #print limits of expectation
  print(mean(expected)-1.96*(sd(expected)/sqrt(no_strains)))
  print(mean(expected)+1.96*(sd(expected)/sqrt(no_strains)))
  
  }
plot_pair("rpn5","lsm2","lsm2_rpn5",-0.4)
```

```
##          sample    strain dose      mean          sd        X1        X2
## 1        wt-atc        wt  atc 1.0381728 0.027310618 1.0227105 1.0077016
## 2      rpn5-atc      rpn5  atc 1.0001154 0.035807044 1.0228446 1.0347660
## 3      lsm2-atc      lsm2  atc 0.9472199 0.005681001 0.9428493 0.9510010
## 4 lsm2_rpn5-atc lsm2_rpn5  atc 0.9603460 0.015204900 0.9555117 0.9471819
##          X3        X4        X5        X6        X7        X8        X9
## 1 0.9909305 1.0684750 1.0527944 1.0352728 1.0715648 1.0558388 1.0382666
## 2 1.0581723 0.9830385 0.9944960 1.0169914 0.9489646 0.9600250 0.9817407
## 3 0.9539212 0.9371614 0.9452639 0.9481664 0.9424668 0.9506152 0.9535341
## 4 0.9420508 0.9614096 0.9530284 0.9478657 0.9861855 0.9775883 0.9722926
##        ymin      ymax
## 1 1.0072679 1.0690776
## 2 0.9595960 1.0406349
## 3 0.9407913 0.9536486
## 4 0.9431401 0.9775520
```

```
## [1] 0.9104101
## [1] 0.9842485
```

```
plot_pair("rpn5","lsm4","lsm4_rpn5",-1.4)
```

```
##          sample    strain dose      mean         sd       X1        X2
## 1        wt-atc        wt  atc 1.0381728 0.02731062 1.022711 1.0077016
## 2      rpn5-atc      rpn5  atc 1.0001154 0.03580704 1.022845 1.0347660
## 5      lsm4-atc      lsm4  atc 1.0240778 0.00695197 1.015453 1.0189271
## 6 lsm4_rpn5-atc lsm4_rpn5  atc 0.9959268 0.01153071 1.011912 0.9890343
##          X3        X4        X5        X6        X7       X8        X9
## 1 0.9909305 1.0684750 1.0527944 1.0352728 1.0715648 1.055839 1.0382666
## 2 1.0581723 0.9830385 0.9944960 1.0169914 0.9489646 0.960025 0.9817407
## 5 1.0234385 1.0170918 1.0205719 1.0250906 1.0281745 1.031693 1.0362605
## 6 0.9891552 1.0088202 0.9860126 0.9861331 1.0126472 0.989753 0.9898740
##        ymin     ymax
## 1 1.0072679 1.069078
## 2 0.9595960 1.040635
## 5 1.0162109 1.031945
## 6 0.9828786 1.008975
```

```
## [1] 0.9841281
## [1] 1.064264
```
