# Supplementary material for: Improved discovery of genetic interactions using CRISPRiSeq across multiple environments
Source: Genome Res. 2019 Apr;29(4):668–81. doi: 10.1101/gr.246603.118 (PMC6442382; doi:10.1101/gr.246603.118)
Supplement: Supplemental Material [file supp_gr.246603.118_Supplemental_CRISPRiSeq-master.zip › CRISPRiSeq-master/figures/supp_note_1.html]

 

 

 

 
 
 


 suppnote1 

 
 
 
 
 

 
 
 
 
 


 

 

 
 


 
 suppnote1 
 


  #load packages and set theme
require(ggplot2)  
  ## Loading required package: ggplot2  
  require(reshape2)  
  ## Loading required package: reshape2  
  theme_dbc <- theme_set(theme_gray())
theme_dbc <- theme_update(
  panel.background = element_rect(fill = "white"),
  panel.border = element_rect( colour = "black",fill=NA,size=2),
  panel.grid.major = element_line(colour = "gray93",size=1),
  panel.grid.minor = element_line(colour = "gray98",size=1),
  strip.text.x = element_text(size=12,face='bold'),
  axis.title = element_text(size=16),
  strip.background = element_rect(colour="black", fill="white",size = 1),
  axis.text = element_text(colour = "black",face="bold",size=16),
  axis.ticks=element_line(color="black",size=2))

ypd24_all_dat = read.table(file="~/Desktop/SherlockLab2/manuscript_aug2017/code/final_fitness_GI_estimates/ypd24_data.txt",sep="\t",header=TRUE,stringsAsFactors = FALSE)
ypd48_all_dat = read.table(file="~/Desktop/SherlockLab2/manuscript_aug2017/code/final_fitness_GI_estimates/ypd48_data.txt",sep="\t",header=TRUE,stringsAsFactors = FALSE)
ypeg_all_dat = read.table(file="~/Desktop/SherlockLab2/manuscript_aug2017/code/final_fitness_GI_estimates/ypeg_data.txt",sep="\t",header=TRUE,stringsAsFactors = FALSE)
ypd37_all_dat = read.table(file="~/Desktop/SherlockLab2/manuscript_aug2017/code/final_fitness_GI_estimates/ypd37_data.txt",sep="\t",header=TRUE,stringsAsFactors = FALSE)
ura_all_dat = read.table(file="~/Desktop/SherlockLab2/manuscript_aug2017/code/final_fitness_GI_estimates/ura_data.txt",sep="\t",header=TRUE,stringsAsFactors = FALSE)  
 Calculate mean single mutant fitness across replicate strains for each replicate of each condition 
  arrays = unique(subset(ypd24_all_dat,category=="double")$array)
queries = c("PRE7g7","PRE4g9","PRE4g3","RPN5g1","COG3g1","SED5g5","SEC22g1","SEC22g2","COG8g2","GET2g2","IMP4g6","DIP2g5","PWP2g2_BC1","PWP2g2_BC2","TIF6g8","RPF1g3","MAK16g1","YCR016Wg4","YLR050Cg1","SAP30g7")

get_sm_fit = function(dat,all_dat){
  dat$r1_array_mean = NA
  dat$r2_array_mean = NA
  dat$r3_array_mean = NA
  for(guide in arrays){
    rows = which(dat$array == guide)
    sub = subset(all_dat,category=="single"&array == guide)
    dat$r1_array_mean[rows]=mean(sub$r1,na.rm=TRUE)
    dat$r2_array_mean[rows]=mean(sub$r2,na.rm=TRUE)
    dat$r3_array_mean[rows]=mean(sub$r3,na.rm=TRUE)
  }
  
  dat$r1_query_mean = NA
  dat$r2_query_mean = NA
  dat$r3_query_mean = NA
  for(guide in queries){
    rows = which(dat$query == guide)
    sub = subset(all_dat,category=="single"&query == guide)
    dat$r1_query_mean[rows]=mean(sub$r1,na.rm=TRUE)
    dat$r2_query_mean[rows]=mean(sub$r2,na.rm=TRUE)
    dat$r3_query_mean[rows]=mean(sub$r3,na.rm=TRUE)
  }
  
  return(dat)
}


ypd24_doubles = get_sm_fit(subset(ypd24_all_dat,category=="double")[,c(31:33,28:30)],ypd24_all_dat)
ypd48_doubles = get_sm_fit(subset(ypd48_all_dat,category=="double")[,c(31:33,28:30)],ypd48_all_dat)
ypeg_doubles = get_sm_fit(subset(ypeg_all_dat,category=="double")[,c(31:33,28:30)],ypeg_all_dat)
ypd37_doubles = get_sm_fit(subset(ypd37_all_dat,category=="double")[,c(31:33,28:30)],ypd37_all_dat)
ura_doubles = get_sm_fit(subset(ura_all_dat,category=="double")[,c(31:33,28:30)],ura_all_dat)  
 Calculated genetic interaction scores empirically, using a linear model to estimate expectation for each group of double mutants generated from the same query guide measured in the same condition 
  get_corrected = function(dat,cond_name){
  
  dat$corrected_i_score_r1 = NA
  dat$corrected_i_score_r2 = NA
  dat$corrected_i_score_r3 = NA
  dat$corrected_expected_r1 = NA
  dat$corrected_expected_r2 = NA
  dat$corrected_expected_r3 = NA
  
  #loop through each query guide
  for(i in 1:20){
    
    rows = which(dat$query==queries[i])#find rows with query guide
    sub = subset(dat,query==queries[i])#subset data to these rows
    lin_fit_r1 = lm(sub$r1~sub$r1_array_mean)#fit linear model from data for this query guide
    lin_fit_r2 = lm(sub$r2~sub$r2_array_mean)#fit linear model from data for this query guide
    lin_fit_r3 = lm(sub$r3~sub$r3_array_mean)#fit linear model from data for this query guide
    #for each strain carrying this query guide,
    #calculate and record expectation and deviation from expectation
    for(row in rows){
      xval_r1 = dat$r1_array_mean[row]
      expected_r1 = lin_fit_r1$coefficients[2]*xval_r1+lin_fit_r1$coefficients[1]
      observed_r1 = dat$r1[row]
      dat$corrected_expected_r1[row]=expected_r1
      dat$corrected_i_score_r1[row]=observed_r1-expected_r1
      
      xval_r2 = dat$r2_array_mean[row]
      expected_r2 = lin_fit_r2$coefficients[2]*xval_r2+lin_fit_r2$coefficients[1]
      observed_r2 = dat$r2[row]
      dat$corrected_expected_r2[row]=expected_r2
      dat$corrected_i_score_r2[row]=observed_r2-expected_r2
      
      xval_r3 = dat$r3_array_mean[row]
      expected_r3 = lin_fit_r3$coefficients[2]*xval_r3+lin_fit_r3$coefficients[1]
      observed_r3 = dat$r3[row]
      dat$corrected_expected_r3[row]=expected_r3
      dat$corrected_i_score_r3[row]=observed_r3-expected_r3
      }
    }
    
  #calculate mean and sd of gi scores across replicate cultures
  dat$gi_mean = apply(dat[,13:15],1,mean)
  dat$gi_sd = apply(dat[,13:15],1,sd)
    
    
  #plot pairwise comparisons of i scores across replicate cultures
  p=ggplot(dat)+ggtitle(cond_name)+geom_point(aes(x=corrected_i_score_r1,y=corrected_i_score_r2),alpha=0.7)
  print(p+facet_wrap(~query))
  p=ggplot(dat)+ggtitle(cond_name)+geom_point(aes(x=corrected_i_score_r1,y=corrected_i_score_r3),alpha=0.7)
  print(p+facet_wrap(~query))
  p=ggplot(dat)+ggtitle(cond_name)+geom_point(aes(x=corrected_i_score_r2,y=corrected_i_score_r3),alpha=0.7)
  print(p+facet_wrap(~query))
  
  #print median and sd of gi scores
  print(median(dat$gi_mean,na.rm=TRUE))
  print(median(dat$gi_sd,na.rm=TRUE))
  
  #plot points and model used for correction (red line is multiplicative mode, blue is empirical)
  p=ggplot(dat,aes(x=r1_array_mean,y=r1))+geom_point(alpha=0.3,size=1)+
    geom_abline(aes(slope=r1_query_mean),color="red",size=1)+
    ggtitle(cond_name)+geom_smooth(method="lm",size=1)
  print(p+facet_wrap(~query))
  p=ggplot(dat,aes(x=r2_array_mean,y=r2))+geom_point(alpha=0.3,size=1)+
    geom_abline(aes(slope=r2_query_mean),color="red",size=1)+
    ggtitle(cond_name)+geom_smooth(method="lm",size=1)
  print(p+facet_wrap(~query))
  p=ggplot(dat,aes(x=r3_array_mean,y=r3))+geom_point(alpha=0.3,size=1)+
    geom_abline(aes(slope=r3_query_mean),color="red",size=1)+
    ggtitle(cond_name)+geom_smooth(method="lm",size=1)
  print(p+facet_wrap(~query))
  
  return(dat)
}

ypd24_doubles=get_corrected(ypd24_doubles,"YPD24hr")  
     
  ## [1] 0.001365467
## [1] 0.04131298  
     
  ypd48_doubles=get_corrected(ypd48_doubles,"YPD48hr")  
     
  ## [1] 0.002964696
## [1] 0.03402522  
     
  ypeg_doubles=get_corrected(ypeg_doubles,"YPEG")  
     
  ## [1] 0.004172295
## [1] 0.05586281  
     
  ypd37_doubles=get_corrected(ypd37_doubles,"YPD37C")  
     
  ## [1] 0.001208931
## [1] 0.05429139  
     
  ura_doubles=get_corrected(ura_doubles,"SCURA")  
     
  ## [1] 0.00133534
## [1] 0.0613997  
     
 For SAP30 calculate a GI score in each replicate based on the multiplicative model 
  calc_sap30 = function(dat){
  rows = which(dat$query=="SAP30g7")
  for(row in rows){
    dat$corrected_expected_r1[row] = dat$r1_query_mean[row]*dat$r1_array_mean[row]
    dat$corrected_i_score_r1[row] = dat$r1[row] - dat$corrected_expected_r1[row]
    
    dat$corrected_expected_r2[row] = dat$r2_query_mean[row]*dat$r2_array_mean[row]
    dat$corrected_i_score_r2[row] = dat$r2[row] - dat$corrected_expected_r2[row]
    
    dat$corrected_expected_r3[row] = dat$r3_query_mean[row]*dat$r3_array_mean[row]
    dat$corrected_i_score_r3[row] = dat$r3[row] - dat$corrected_expected_r3[row]
  }
  dat$gi_mean[rows]=apply(dat[rows,13:15],1,mean)
  dat$gi_sd[rows]=apply(dat[rows,13:15],1,sd)
  
  print(qplot(dat$gi_mean[rows],geom="histogram"))
  
  return(dat)
}

ypd24_doubles = calc_sap30(ypd24_doubles)  
  ## stat_bin: binwidth defaulted to range/30. Use 'binwidth = x' to adjust this.  
   
  ypd48_doubles = calc_sap30(ypd48_doubles)  
  ## stat_bin: binwidth defaulted to range/30. Use 'binwidth = x' to adjust this.  
   
  ypeg_doubles = calc_sap30(ypeg_doubles)  
  ## stat_bin: binwidth defaulted to range/30. Use 'binwidth = x' to adjust this.  
   
  ypd37_doubles = calc_sap30(ypd37_doubles)  
  ## stat_bin: binwidth defaulted to range/30. Use 'binwidth = x' to adjust this.  
   
  ura_doubles = calc_sap30(ura_doubles)  
  ## stat_bin: binwidth defaulted to range/30. Use 'binwidth = x' to adjust this.  
   
 Perform t-test for each strain in each condition (3 replicate GI scores, vs all observed GI scores) Adjust p-values using Benjamini-Hochberg 
  all_measurements = c(ypd24_doubles$corrected_i_score_r1,
                     ypd24_doubles$corrected_i_score_r2,
                     ypd24_doubles$corrected_i_score_r3,
                     ypd48_doubles$corrected_i_score_r1,
                     ypd48_doubles$corrected_i_score_r2,
                     ypd48_doubles$corrected_i_score_r3,
                     ypeg_doubles$corrected_i_score_r1,
                     ypeg_doubles$corrected_i_score_r2,
                     ypeg_doubles$corrected_i_score_r3,
                     ypd37_doubles$corrected_i_score_r1,
                     ypd37_doubles$corrected_i_score_r2,
                     ypd37_doubles$corrected_i_score_r3,
                     ura_doubles$corrected_i_score_r1,
                     ura_doubles$corrected_i_score_r2,
                     ura_doubles$corrected_i_score_r3)


ypd24_measurements = c(ypd24_doubles$corrected_i_score_r1,
                       ypd24_doubles$corrected_i_score_r2,
                       ypd24_doubles$corrected_i_score_r3)
ypd48_measurements = c(ypd48_doubles$corrected_i_score_r1,
                       ypd48_doubles$corrected_i_score_r2,
                       ypd48_doubles$corrected_i_score_r3)
ypeg_measurements = c(ypeg_doubles$corrected_i_score_r1,
                      ypeg_doubles$corrected_i_score_r2,
                      ypeg_doubles$corrected_i_score_r3)
ypd37_measurements = c(ypd37_doubles$corrected_i_score_r1,
                       ypd37_doubles$corrected_i_score_r2,
                       ypd37_doubles$corrected_i_score_r3)
ura_measurements = c(ura_doubles$corrected_i_score_r1,
                     ura_doubles$corrected_i_score_r2,
                     ura_doubles$corrected_i_score_r3)

calc_ttest=function(dat,bg){
  dat$t.pval = NA
  for(i in 1:dim(dat)[1]){
    if(i%%1000==0){print(i)}
    values = unlist(dat[i,13:15])
    if(length(which(is.na(values)))>0){next}
    dat$t.pval[i] = t.test(values,bg)$p.value
    }
  return(dat)
  }

ypd24_doubles=calc_ttest(ypd24_doubles,ypd24_measurements)  
  ## [1] 1000
## [1] 2000
## [1] 3000
## [1] 4000
## [1] 5000
## [1] 6000
## [1] 7000
## [1] 8000
## [1] 9000
## [1] 10000
## [1] 11000
## [1] 12000
## [1] 13000
## [1] 14000
## [1] 15000  
  ypd48_doubles=calc_ttest(ypd48_doubles,ypd48_measurements)  
  ## [1] 1000
## [1] 2000
## [1] 3000
## [1] 4000
## [1] 5000
## [1] 6000
## [1] 7000
## [1] 8000
## [1] 9000
## [1] 10000
## [1] 11000
## [1] 12000
## [1] 13000
## [1] 14000
## [1] 15000  
  ypeg_doubles=calc_ttest(ypeg_doubles,ypeg_measurements)  
  ## [1] 1000
## [1] 2000
## [1] 3000
## [1] 4000
## [1] 5000
## [1] 6000
## [1] 7000
## [1] 8000
## [1] 9000
## [1] 10000
## [1] 11000
## [1] 12000
## [1] 13000
## [1] 14000
## [1] 15000  
  ypd37_doubles=calc_ttest(ypd37_doubles,ypd37_measurements)  
  ## [1] 1000
## [1] 2000
## [1] 3000
## [1] 4000
## [1] 5000
## [1] 6000
## [1] 7000
## [1] 8000
## [1] 9000
## [1] 10000
## [1] 11000
## [1] 12000
## [1] 13000
## [1] 14000
## [1] 15000  
  ura_doubles=calc_ttest(ura_doubles,ura_measurements)  
  ## [1] 1000
## [1] 2000
## [1] 3000
## [1] 4000
## [1] 5000
## [1] 6000
## [1] 7000
## [1] 8000
## [1] 9000
## [1] 10000
## [1] 11000
## [1] 12000
## [1] 13000
## [1] 14000
## [1] 15000  
  qplot(c(ypd24_doubles$t.pval,ypd48_doubles$t.pval,ypeg_doubles$t.pval,ypd37_doubles$t.pval,ura_doubles$t.pval),geom="histogram")  
  ## stat_bin: binwidth defaulted to range/30. Use 'binwidth = x' to adjust this.  
   
  #correct for FDR
adjusted_p = p.adjust(c(ypd24_doubles$t.pval,
                        ypd48_doubles$t.pval,
                        ypeg_doubles$t.pval,
                        ypd37_doubles$t.pval,
                        ura_doubles$t.pval),method="BH")

#plot adjusted versus unadjusted p values
qplot(c(ypd24_doubles$t.pval,
        ypd48_doubles$t.pval,
        ypeg_doubles$t.pval,
        ypd37_doubles$t.pval,
        ura_doubles$t.pval),adjusted_p)+xlab("unadjusted p-value")+ylab("adjusted p-value")  
   
  ypd24_doubles$p.adj = adjusted_p[1:15200]
ypd48_doubles$p.adj = adjusted_p[15201:30400]
ypeg_doubles$p.adj = adjusted_p[30401:45600]
ypd37_doubles$p.adj = adjusted_p[45601:60800]
ura_doubles$p.adj = adjusted_p[60801:76000]


#plot number of guide pairs passing each FDR cutoff for each condition
thresh_values= seq(from=0,to=1,by=0.05)
dist_p = data.frame(matrix(nrow=length(thresh_values),ncol=6))
names(dist_p)=c("thresh_val","ypd24","ypd48","ypeg","ypd37","ura")
dist_p$thresh_val=thresh_values
count = 0
for(thresh in thresh_values){
  count = count + 1
  print(thresh)
  dist_p$ypd24[count]=length(which(ypd24_doubles$p.adj<thresh))
  dist_p$ypd48[count]=length(which(ypd48_doubles$p.adj<thresh))
  dist_p$ypeg[count]=length(which(ypeg_doubles$p.adj<thresh))
  dist_p$ypd37[count]=length(which(ypd37_doubles$p.adj<thresh))
  dist_p$ura[count]=length(which(ura_doubles$p.adj<thresh))
}  
  ## [1] 0
## [1] 0.05
## [1] 0.1
## [1] 0.15
## [1] 0.2
## [1] 0.25
## [1] 0.3
## [1] 0.35
## [1] 0.4
## [1] 0.45
## [1] 0.5
## [1] 0.55
## [1] 0.6
## [1] 0.65
## [1] 0.7
## [1] 0.75
## [1] 0.8
## [1] 0.85
## [1] 0.9
## [1] 0.95
## [1] 1  
  temp = melt(dist_p,id.vars=c("thresh_val"))
ggplot(temp,aes(x=thresh_val,y=value,color=variable))+geom_line(size=2,alpha=0.5)+xlab("FDR cutoff (q-value)")+ylab("guide pairs passing")+ggtitle("Threshold of FDR")+scale_color_manual(values=c("#A3A500","#00B0F6","#E76BF3","#00BF7D","#F8766D"))  
   
  #plot number of guide pairs passing each FDR cutoff and absolute value of a GI score of greater than 0.1 for each condition
thresh_gi=0.1
thresh_values= seq(from=0,to=1,by=0.05)
dist_p_add = data.frame(matrix(nrow=length(thresh_values),ncol=6))
names(dist_p_add)=c("thresh_val","ypd24","ypd48","ypeg","ypd37","ura")
dist_p_add$thresh_val=thresh_values
count = 0
for(thresh in thresh_values){
  count = count + 1
  print(thresh)
  dist_p_add$ypd24[count]=length(which(ypd24_doubles$p.adj<thresh&abs(ypd24_doubles$gi_mean)>thresh_gi))
  dist_p_add$ypd48[count]=length(which(ypd48_doubles$p.adj<thresh&abs(ypd48_doubles$gi_mean)>thresh_gi))
  dist_p_add$ypeg[count]=length(which(ypeg_doubles$p.adj<thresh&abs(ypeg_doubles$gi_mean)>thresh_gi))
  dist_p_add$ypd37[count]=length(which(ypd37_doubles$p.adj<thresh&abs(ypd37_doubles$gi_mean)>thresh_gi))
  dist_p_add$ura[count]=length(which(ura_doubles$p.adj<thresh&abs(ura_doubles$gi_mean)>thresh_gi))
}  
  ## [1] 0
## [1] 0.05
## [1] 0.1
## [1] 0.15
## [1] 0.2
## [1] 0.25
## [1] 0.3
## [1] 0.35
## [1] 0.4
## [1] 0.45
## [1] 0.5
## [1] 0.55
## [1] 0.6
## [1] 0.65
## [1] 0.7
## [1] 0.75
## [1] 0.8
## [1] 0.85
## [1] 0.9
## [1] 0.95
## [1] 1  
  temp = melt(dist_p_add,id.vars=c("thresh_val"))
ggplot(temp)+geom_line(aes(x=thresh_val,y=value,color=variable),size=2,alpha=0.5)+xlab("FDR cutoff (q-value)")+ylab("guide pairs passing")+ggtitle("with additional threshold (abs(GI)>0.1)")+scale_color_manual(values=c("#A3A500","#00B0F6","#E76BF3","#00BF7D","#F8766D"))  
   
 Compare mean GI score (across replicate cultures) to those computed previously (after combining replicate cultures) 
  ypd24_temp = read.table(file="~/Desktop/SherlockLab2/manuscript_aug2017/code/final_fitness_GI_estimates/ypd24_short.txt",sep="\t",header=TRUE,stringsAsFactors = FALSE)
ypd48_temp = read.table(file="~/Desktop/SherlockLab2/manuscript_aug2017/code/final_fitness_GI_estimates/ypd48_short.txt",sep="\t",header=TRUE,stringsAsFactors = FALSE)
ypeg_temp = read.table(file="~/Desktop/SherlockLab2/manuscript_aug2017/code/final_fitness_GI_estimates/ypeg_short.txt",sep="\t",header=TRUE,stringsAsFactors = FALSE)
ypd37_temp = read.table(file="~/Desktop/SherlockLab2/manuscript_aug2017/code/final_fitness_GI_estimates/ypd37_short.txt",sep="\t",header=TRUE,stringsAsFactors = FALSE)
ura_temp = read.table(file="~/Desktop/SherlockLab2/manuscript_aug2017/code/final_fitness_GI_estimates/ura_short.txt",sep="\t",header=TRUE,stringsAsFactors = FALSE)

ggplot()+geom_abline(color="red")+
  geom_point(aes(x=ypd24_doubles$gi_mean,y=ypd24_temp$gi_score),alpha=0.5)+
  xlim(-0.61,1.15)+ylim(-0.61,1.15)+ggtitle("YPD24hr")+
  xlab("GI computed in each replicate")+ylab("GI computed from mean across replicates")  
   
  ggplot()+geom_abline(color="red")+
  geom_point(aes(x=ypd48_doubles$gi_mean,y=ypd48_temp$gi_score),alpha=0.5)+
  xlim(-0.61,1.15)+ylim(-0.61,1.15)+ggtitle("YPD48hr")+
  xlab("GI computed in each replicate")+ylab("GI computed from mean across replicates")  
   
  ggplot()+geom_abline(color="red")+
  geom_point(aes(x=ypeg_doubles$gi_mean,y=ypeg_temp$gi_score),alpha=0.5)+
  xlim(-0.61,1.15)+ylim(-0.61,1.15)+ggtitle("YPEG")+
  xlab("GI computed in each replicate")+ylab("GI computed from mean across replicates")  
   
  ggplot()+geom_abline(color="red")+
  geom_point(aes(x=ypd37_doubles$gi_mean,y=ypd37_temp$gi_score),alpha=0.5)+
  xlim(-0.61,1.15)+ylim(-0.61,1.15)+ggtitle("YPD37C")+
  xlab("GI computed in each replicate")+ylab("GI computed from mean across replicates")  
   
  ggplot()+geom_abline(color="red")+
  geom_point(aes(x=ura_doubles$gi_mean,y=ura_temp$gi_score),alpha=0.5)+
  xlim(-0.61,1.15)+ylim(-0.61,1.15)+ggtitle("SCURA")+
  xlab("GI computed in each replicate")+ylab("GI computed from mean across replicates")  
   
  gi_sd=sd(c(ypd24_temp$gi_score,ypd48_temp$gi_score,ypeg_temp$gi_score,ypd37_temp$gi_score,ura_temp$gi_score),na.rm=TRUE)
gi_mean = mean(c(ypd24_temp$gi_score,ypd48_temp$gi_score,ypeg_temp$gi_score,ypd37_temp$gi_score,ura_temp$gi_score),na.rm=TRUE)  
 Plot GI score versus q-value and number significant 
  temp = data.frame(ypd24_doubles$p.adj,abs(ypd24_doubles$gi_mean))
names(temp)=c("qval","gi_score")
ggplot(temp)+annotate("rect",xmin=0,xmax=0.2,ymin=0.1,ymax=1.15,color="blue",fill="blue",alpha=0.5)+
  geom_point(aes(x=qval,y=gi_score),alpha=0.3)+ggtitle("YPD24hr: qval<0.2 and abs(GI score)>0.1")+ylab("Abs(GI Score)")+xlab("q-value")+
  annotate("text",label="number of significant GIs:",x=0.5,y=0.8)+
  annotate("text",label=as.character(length(which(temp$qval<0.2&temp$gi_score>0.1))),x=0.5,y=0.7)  
   
  temp = data.frame(ypd48_doubles$p.adj,abs(ypd48_doubles$gi_mean))
names(temp)=c("qval","gi_score")
ggplot(temp)+annotate("rect",xmin=0,xmax=0.2,ymin=0.1,ymax=1.15,color="blue",fill="blue",alpha=0.5)+
  geom_point(aes(x=qval,y=gi_score),alpha=0.3)+ggtitle("YPD48hr: qval<0.2 and abs(GI score)>0.1")+ylab("Abs(GI Score)")+xlab("q-value")+
  annotate("text",label="number of significant GIs:",x=0.5,y=0.8)+
  annotate("text",label=as.character(length(which(temp$qval<0.2&temp$gi_score>0.1))),x=0.5,y=0.7)  
   
  temp = data.frame(ypeg_doubles$p.adj,abs(ypeg_doubles$gi_mean))
names(temp)=c("qval","gi_score")
ggplot(temp)+annotate("rect",xmin=0,xmax=0.2,ymin=0.1,ymax=1.15,color="blue",fill="blue",alpha=0.5)+
  geom_point(aes(x=qval,y=gi_score),alpha=0.3)+ggtitle("YPEG: qval<0.2 and abs(GI score)>0.1")+ylab("Abs(GI Score)")+xlab("q-value")+
  annotate("text",label="number of significant GIs:",x=0.5,y=0.8)+
  annotate("text",label=as.character(length(which(temp$qval<0.2&temp$gi_score>0.1))),x=0.5,y=0.7)  
   
  temp = data.frame(ypd37_doubles$p.adj,abs(ypd37_doubles$gi_mean))
names(temp)=c("qval","gi_score")
ggplot(temp)+annotate("rect",xmin=0,xmax=0.2,ymin=0.1,ymax=1.15,color="blue",fill="blue",alpha=0.5)+
  geom_point(aes(x=qval,y=gi_score),alpha=0.3)+ggtitle("YPD37: qval<0.2 and abs(GI score)>0.1")+ylab("Abs(GI Score)")+xlab("q-value")+
  annotate("text",label="number of significant GIs:",x=0.5,y=0.8)+
  annotate("text",label=as.character(length(which(temp$qval<0.2&temp$gi_score>0.1))),x=0.5,y=0.7)  
   
  temp = data.frame(ura_doubles$p.adj,abs(ura_doubles$gi_mean))
names(temp)=c("qval","gi_score")
ggplot(temp)+annotate("rect",xmin=0,xmax=0.2,ymin=0.1,ymax=1.15,color="blue",fill="blue",alpha=0.5)+
  geom_point(aes(x=qval,y=gi_score),alpha=0.3)+ggtitle("SCURA: qval<0.2 and abs(GI score)>0.1")+ylab("Abs(GI Score)")+xlab("q-value")+
  annotate("text",label="number of significant GIs:",x=0.5,y=0.8)+
  annotate("text",label=as.character(length(which(temp$qval<0.2&temp$gi_score>0.1))),x=0.5,y=0.7)  
   
 Remake figure 2A with new calls of significance using FDR 
  #put GI score data and significance from each conditions into a single data frame
all_data = ypd24_doubles[,c(2,3,19)]
names(all_data)[3]="YPD24hr"
all_data$YPD48hr = ypd48_doubles[,19]
all_data$YPEG = ypeg_doubles[,19]
all_data$YPD37C = ypd37_doubles[,19]
all_data$SCURA = ura_doubles[,19]
all_data$YPD24hr.qval = ypd24_doubles[,22]
all_data$YPD48hr.qval = ypd48_doubles[,22]
all_data$YPEG.qval = ypeg_doubles[,22]
all_data$YPD37C.qval = ypd37_doubles[,22]
all_data$SCURA.qval = ura_doubles[,22]

#add columns to record whether an interaction was detected in each condition
all_data$YPD24hr.pos=0
all_data$YPD48hr.pos=0
all_data$YPEG.pos=0
all_data$YPD37.pos=0
all_data$SCURA.pos=0

all_data$YPD24hr.neg=0
all_data$YPD48hr.neg=0
all_data$YPEG.neg=0
all_data$YPD37.neg=0
all_data$SCURA.neg=0

#create columns to record how many conditions a GI was detected in
all_data$num_pos=NA
all_data$num_neg=NA

#loop through each gene pair and record whether a GI was detected in each condition
#Use FDR of 20% and absolute value of GI score greater than 0.1
for(i in 1:dim(all_data)[1]){
  if(i%%1000==0){print(i)}
  pos_count = 0
  neg_count = 0
  for(j in 3:7){
    if(is.na(all_data[i,j])){next}
    if(all_data[i,j]>0.1&all_data[i,(j+5)]<0.2){
      all_data[i,j+10]=1
      pos_count = pos_count+1
      }
    if(all_data[i,j]< -0.1&all_data[i,(j+5)]<0.2){
      all_data[i,j+15]=1
      neg_count = neg_count+1
      }
  }
  all_data$num_pos[i]=pos_count
  all_data$num_neg[i]=neg_count
}  
  ## [1] 1000
## [1] 2000
## [1] 3000
## [1] 4000
## [1] 5000
## [1] 6000
## [1] 7000
## [1] 8000
## [1] 9000
## [1] 10000
## [1] 11000
## [1] 12000
## [1] 13000
## [1] 14000
## [1] 15000  
  #check how many were in each category
xtabs(~num_pos+num_neg,data=all_data)  
  ##        num_neg
## num_pos     0     1     2     3
##       0 14017   392    35     1
##       1   547     3     0     0
##       2   151     0     0     0
##       3    51     0     0     0
##       4     3     0     0     0  
  #save gene pairs where sign change occurs
dyn_sub=subset(all_data,num_pos!=0&num_neg!=0)

#remove rows where change in sign occurs
sub_data = all_data[-which(all_data$num_pos>0&all_data$num_neg>0),]

#remove rows where no interaction was detected
sub_data = sub_data[-which(sub_data$num_pos==0&sub_data$num_neg==0),]

#record whether the interaction is condition specific
cond_data = subset(sub_data,num_pos==1|num_neg==1)#first subset to rows with just 1 GI
#remove rows with missing measurements
cond_data$num_na=apply(cond_data[,3:7],1,function(x)length(which(is.na(x))))
cond_data = cond_data[-which(cond_data$num_na!=0),-25]
#record whether GI score is condition specific (<0.1 in all other conditions for positive GI, and >-0.1 for negative GI)
cond_data$stringent_pos = apply(cond_data[,3:7],1,function(x)length(which(x > 0.1))) #number of conditions with GI score greater than threshold
cond_data$stringent_neg = apply(cond_data[,3:7],1,function(x)length(which(x < -0.1))) #number of conditions with GI score less than threshold
cond_data$stringent=FALSE
cond_data$stringent[which(cond_data$num_pos==1&cond_data$stringent_pos==1)]=TRUE
cond_data$stringent[which(cond_data$num_neg==1&cond_data$stringent_neg==1)]=TRUE
cond_data = subset(cond_data,stringent==1)

#subset all data to only guide pairs with measurements in all 5 conditions
temp2 = all_data
temp2$missing = apply(temp2[,3:7],1,function(x)length(which(is.na(x))))
temp2 = temp2[-which(temp2$missing>0),-25]
temp2$stringent = rownames(temp2)%in%rownames(cond_data)
print(dim(temp2))  
  ## [1] 12729    25  
  #record number of GIs in each condition, also note condition specific and cumulative total
int_cond = data.frame(matrix(nrow=5,ncol=8))
names(int_cond)=c("cond","neg_gi","pos_gi","pos_sap","pos_notsap","cond_spec","cumulative","both")
int_cond$cond = 1:5

count = 0
for(i in 3:7){
  count = count + 1
  int_cond$pos_gi[count]=length(which(temp2[,(i+10)] == 1))#/dim(temp2)[1]*100
  int_cond$neg_gi[count]=length(which(temp2[,(i+15)] == 1))#/dim(temp2)[1]*100
  sub = temp2[which(temp2[,(i+10)] == 1),]
  int_cond$pos_sap[count] = length(which(sub$query=="SAP30g7"))#/dim(temp2)[1]*100
  int_cond$pos_notsap[count] = (dim(sub)[1]-length(which(sub$query=="SAP30g7")))#/dim(temp2)[1]*100
  
  
  sub2 = temp2[which(temp2[,(i+10)] == 1|temp2[,(i+15)] == 1),]
  if(count ==1){runtot = rownames(sub2)}
  if(count > 1){runtot = unique(c(runtot,rownames(sub2)))}
  int_cond$cumulative[count]=length(runtot)#/dim(temp2)[1]*100
  
  int_cond$cond_spec[count]=length(which(sub2$stringent==TRUE))#/dim(temp2)[1]*100
  int_cond$both[count]=dim(sub2)[1]
  
}

#make bar graph
ggplot(melt(int_cond[,-3],id.vars=c("cond","cumulative","cond_spec","both")))+
  geom_bar(aes(x=cond,y=value,fill=as.character(cond),alpha=variable),
           stat="identity",position="stack",color="black")+
  scale_fill_manual(values=c("#A3A500","#00B0F6","#E76BF3","#00BF7D","#F8766D"))+
  scale_alpha_manual(values=c(1,0.6,0.2))+
  geom_line(size=2,alpha=0.9,aes(x=cond,y=cumulative),color="grey19")+
  geom_point(size=8,alpha=0.9,aes(x=cond,y=cumulative),color="grey19")+
  geom_line(size=2,alpha=0.9,aes(x=cond,y=cond_spec),color="grey19")+
  geom_point(size=8,alpha=0.9,aes(x=cond,y=cond_spec),color="grey19",shape=17)+
  xlab("")+ylab("")+theme(axis.text.x=element_blank())  
   
 Save additional files with qvalues and zscore significance for each gene pair 
  #make data frame with the following columns
#gi_score from method 1 (mean of reps, then gi score calculation)
#z-scores from method 1
#gi_score r1 (method2)
#gi_score r2 (method2)
#gi_score r3 (method2)
#mean gi_score (method2)
#q-value (method2)

combine_data = function(dat_method1,dat_method2){
  combined = data.frame(dat_method1$strain,dat_method1$gi_score)
  names(combined)=c("strain","gi_score_method1")
  combined$zscore_method1= (combined$gi_score_method1-gi_mean)/gi_sd
  combined$gi_score_method2 = dat_method2$gi_mean
  combined$qval_method2 = dat_method2$p.adj
  return(combined)
  }
write.table(combine_data(ypd24_temp,ypd24_doubles),file="~/Desktop/SherlockLab2/manuscript_aug2017/code/final_fitness_GI_estimates/ypd24_sig.txt",sep="\t")

write.table(combine_data(ypd48_temp,ypd48_doubles),file="~/Desktop/SherlockLab2/manuscript_aug2017/code/final_fitness_GI_estimates/ypd48_sig.txt",sep="\t")

write.table(combine_data(ypeg_temp,ypeg_doubles),file="~/Desktop/SherlockLab2/manuscript_aug2017/code/final_fitness_GI_estimates/ypeg_sig.txt",sep="\t")

write.table(combine_data(ypd37_temp,ypd37_doubles),file="~/Desktop/SherlockLab2/manuscript_aug2017/code/final_fitness_GI_estimates/ypd37_sig.txt",sep="\t")

write.table(combine_data(ura_temp,ura_doubles),file="~/Desktop/SherlockLab2/manuscript_aug2017/code/final_fitness_GI_estimates/ura_sig.txt",sep="\t")  


 

 

 
 

 
 
