# Supplementary material for: Improved discovery of genetic interactions using CRISPRiSeq across multiple environments
Source: Genome Res. 2019 Apr;29(4):668–81. doi: 10.1101/gr.246603.118 (PMC6442382; doi:10.1101/gr.246603.118)
Supplement: Supplemental Material [file supp_gr.246603.118_Supplemental_CRISPRiSeq-master.zip › CRISPRiSeq-master/processing_data/2_PCRchimera_removal_RMarkdown_files/seqlib11.html]

seqlib11


# seqlib11

```
require(ggplot2)
```

```
## Loading required package: ggplot2
```

```
require(reshape2)
```

```
## Loading required package: reshape2
```

```
theme_dbc <- theme_set(theme_gray())
theme_dbc <- theme_update(
  panel.background = element_rect(fill = "white"),
  panel.border = element_rect( colour = "black",fill=NA,size=2),
  panel.grid.major = element_line(colour = "gray93",size=1),
  panel.grid.minor = element_line(colour = "gray98",size=1),
  strip.text.x = element_text(size=12,face='bold'),
  axis.title = element_text(size=16),
  strip.background = element_rect(colour="black", fill="white",size = 1),
  axis.text = element_text(colour = "black",face="bold",size=16),
  axis.ticks=element_line(color="black",size=2))

dat = read.table(file="~/Desktop/SherlockLab2/System_test/screen2_hiseq/seqlib11_analysis/bc_counts/all_counts.txt", sep="\t",header=TRUE,row.names=1,stringsAsFactors = FALSE)
dat2 = read.table(file = "~/Desktop/SherlockLab2/System_test/screen2_hiseq/seqlib11_analysis/bc_counts/recovered_counts.txt", sep="\t",header=TRUE,row.names=1,stringsAsFactors = FALSE)
dat5 = dat+dat2

rm(dat)
rm(dat2)

key = read.table(file="~/Desktop/SherlockLab2/System_test/screen2_hiseq/data_table_row_key.txt",sep="\t",header=TRUE,stringsAsFactors = FALSE)

no_libs = 14
```

Look at counts for present barcodes vs not present barcodes

```
for(i in 1:25){
  p=ggplot()+geom_histogram(aes(x=dat5[which(key$present=="yes"),i]),alpha=0.5,binwidth=5,fill="green")+
    geom_histogram(aes(x=dat5[which(key$present=="no"),i]),alpha=0.5,binwidth=5,fill="blue")+ylim(0,500)+ggtitle(names(dat5)[i])
  print(p)
  }
```

Look at Batch 1 start vs Batch 2 start

```
ggplot(dat5,aes(x=Batch1T0,y=Batch2T0))+geom_point(alpha=0.1)+scale_x_log10()+scale_y_log10()+
  geom_abline(color="red")
```

```
ggplot(dat5,aes(x=Batch1T0,y=Batch2T0))+geom_point(alpha=0.1)+
  geom_abline(color="red")+geom_smooth(method="lm")
```

Look at coverage across libraries

```
ggplot(melt(dat5))+geom_boxplot(aes(x=variable,y=value,color=variable))+
  theme(legend.position="none",axis.text.x=element_text(angle=60,hjust=1))
```

```
## No id variables; using all as measure variables
```

Look at representation of each query guide in starting pool

```
sub = dat5
sub$category = key$category
sub$query = key$query
sub = sub[which(key$present=="yes"),]
for(i in 1:14){
  temp = sub[,c(i,26,27)]
  names(temp)[1]="count"
  p=ggplot(temp)+geom_boxplot(aes(x=query,y=count,color=query))+ggtitle(names(dat5)[i])+
    theme(legend.position="none",axis.text.x = element_text(angle = 60, hjust = 1))+scale_y_log10()
  print(p+facet_wrap(~category))
  print(names(dat5)[i])
  print(length(which(temp$count==0)))
  print(rownames(sub)[which(temp$count==0)])
}
```

```
## Warning: Removed 7 rows containing non-finite values (stat_boxplot).
```

```
## [1] "Batch1T0"
## [1] 7
## [1] "PRE7g7-RFC5-NRg-2"  "PRE4g9-RFC5-NRg-2"  "COG3g1-RFC5-NRg-2" 
## [4] "SED5g5-RFC5-NRg-2"  "DIP2g5-RFC5-NRg-2"  "TIF6g8-RFC5-NRg-2" 
## [7] "SAP30g7-RFC5-NRg-2"
```

```
## Warning: Removed 4 rows containing non-finite values (stat_boxplot).
```

```
## [1] "YPDR1T1"
## [1] 4
## [1] "PRE7g7-RFC5-NRg-2" "PRE4g9-RFC5-NRg-2" "COG3g1-RFC5-NRg-2"
## [4] "DIP2g5-RFC5-NRg-2"
```

```
## Warning: Removed 4 rows containing non-finite values (stat_boxplot).
```

```
## [1] "YPDR1T2"
## [1] 4
## [1] "PRE7g7-RFC5-NRg-2"  "SED5g5-RFC5-NRg-2"  "TIF6g8-RFC5-NRg-2" 
## [4] "SAP30g7-RFC5-NRg-2"
```

```
## Warning: Removed 3 rows containing non-finite values (stat_boxplot).
```

```
## [1] "YPDR1T3"
## [1] 3
## [1] "PRE7g7-RFC5-NRg-2" "PRE4g9-RFC5-NRg-2" "COG3g1-RFC5-NRg-2"
```

```
## Warning: Removed 1 rows containing non-finite values (stat_boxplot).
```

```
## [1] "YPDR2T1"
## [1] 1
## [1] "DIP2g5-RFC5-NRg-2"
```

```
## Warning: Removed 4 rows containing non-finite values (stat_boxplot).
```

```
## [1] "YPDR2T2"
## [1] 4
## [1] "PRE7g7-RFC5-NRg-2" "PRE4g9-RFC5-NRg-2" "COG3g1-RFC5-NRg-2"
## [4] "DIP2g5-RFC5-NRg-2"
```

```
## Warning: Removed 6 rows containing non-finite values (stat_boxplot).
```

```
## [1] "YPDR2T3"
## [1] 6
## [1] "PRE4g9-RFC5-NRg-2" "COG3g1-RFC5-NRg-2" "SED5g5-RFC5-NRg-2"
## [4] "GET2g2-CCT8-TRg-2" "DIP2g5-RFC5-NRg-2" "TIF6g8-RFC5-NRg-2"
```

```
## Warning: Removed 5 rows containing non-finite values (stat_boxplot).
```

```
## [1] "YPDR3T1"
## [1] 5
## [1] "PRE7g7-RFC5-NRg-2"  "PRE4g9-RFC5-NRg-2"  "DIP2g5-RFC5-NRg-2" 
## [4] "TIF6g8-RFC5-NRg-2"  "SAP30g7-RFC5-NRg-2"
```

```
## Warning: Removed 7 rows containing non-finite values (stat_boxplot).
```

```
## [1] "YPDR3T2"
## [1] 7
## [1] "PRE7g7-RFC5-NRg-2"  "PRE4g9-RFC5-NRg-2"  "SED5g5-RFC5-NRg-2" 
## [4] "GET2g2-CCT8-TRg-2"  "DIP2g5-RFC5-NRg-2"  "TIF6g8-RFC5-NRg-2" 
## [7] "SAP30g7-RFC5-NRg-2"
```

```
## Warning: Removed 3 rows containing non-finite values (stat_boxplot).
```

```
## [1] "YPDR3T3"
## [1] 3
## [1] "PRE7g7-RFC5-NRg-2" "GET2g2-CCT8-TRg-2" "DIP2g5-RFC5-NRg-2"
```

```
## Warning: Removed 1 rows containing non-finite values (stat_boxplot).
```

```
## [1] "YPDR4T1"
## [1] 1
## [1] "PRE4g9-RFC5-NRg-2"
```

```
## Warning: Removed 4 rows containing non-finite values (stat_boxplot).
```

```
## [1] "YPDR4T2"
## [1] 4
## [1] "PRE7g7-RFC5-NRg-2" "COG3g1-RFC5-NRg-2" "SED5g5-RFC5-NRg-2"
## [4] "TIF6g8-RFC5-NRg-2"
```

```
## Warning: Removed 5 rows containing non-finite values (stat_boxplot).
```

```
## [1] "YPDR4T3"
## [1] 5
## [1] "PRE7g7-RFC5-NRg-2"    "DIP2g5-CDC42-TRg-6"   "DIP2g5-RFC5-NRg-2"   
## [4] "TIF6g8-RFC5-NRg-2"    "YLR050Cg1-RFC5-NRg-2"
```

```
## Warning: Removed 7 rows containing non-finite values (stat_boxplot).
```

```
## [1] "Batch2T0"
## [1] 7
## [1] "PRE7g7-RFC5-NRg-2"    "PRE4g9-RFC5-NRg-2"    "COG3g1-RFC5-NRg-2"   
## [4] "DIP2g5-RFC5-NRg-2"    "TIF6g8-RFC5-NRg-2"    "YLR050Cg1-RFC5-NRg-2"
## [7] "SAP30g7-RFC5-NRg-2"
```

Calculate and plot chimera subtraction

```
#store what BC1, BC2, and DBC is represented on each line of dat5
all_BC1 = key$query
all_BC2 = key$array
all_DBC = rownames(dat5)

#make data frame to store frequencies for each of BC1s in each samples
BC1_freqs = data.frame(matrix(nrow=length(unique(all_BC1)),ncol=no_libs))
names(BC1_freqs) = names(dat5)[1:no_libs]
BC1_freqs$BC1_name = unique(all_BC1)

#loop through BC1s and find total frequencies in each of the samples
for (i in 1:dim(BC1_freqs)[1]){
  for (j in 1:(dim(BC1_freqs)[2]-1)){
    BC1_freqs[i,j] = sum(dat5[which(all_BC1==BC1_freqs$BC1_name[i]),j])
    }
  }

#Make data frame to store frequencies for each of the BC2s in samples
BC2_freqs = data.frame(matrix(nrow=length(unique(all_BC2)),ncol=no_libs))
names(BC2_freqs)=names(dat5)[1:no_libs]
BC2_freqs$BC2_names = unique(all_BC2)

#loop through BC2s and find total frequencies in each of the samples
for (i in 1:dim(BC2_freqs)[1]){
  for (j in 1:(dim(BC2_freqs)[2]-1)){
    BC2_freqs[i,j] = sum(dat5[which(all_BC2==BC2_freqs$BC2_names[i]),j])
    }
  }
```

```
#Function to calculate expected number of counts at the given time point for each DBC
get_expected = function(sample){
  
  print(names(dat5)[sample])  
  temp = rep(NA,dim(dat5)[1])
  
  #calculate expected counts for each DBC at each time point
  for (i in 1:dim(dat5)[1]){      
    
    #expected frequency of DBC based on two single mutants
    BC1_i = which(BC1_freqs$BC1_name==all_BC1[i])
    BC1_count = as.numeric(BC1_freqs[BC1_i,sample])
    
    BC2_i = which(BC2_freqs$BC2_name==all_BC2[i])
    BC2_count = as.numeric(BC2_freqs[BC2_i,sample])
    
    temp[i]=BC1_count * BC2_count
    
    }
    return(temp)  
}

#Calculate expectation for all counts in data matrix
dat5_expected = data.frame(matrix(nrow=dim(dat5)[1],ncol=no_libs))
names(dat5_expected)=names(dat5)[1:no_libs]
rownames(dat5_expected)=rownames(dat5)
for(i in 1:no_libs){
  dat5_expected[,i] = get_expected(i)
}
```

```
## [1] "Batch1T0"
## [1] "YPDR1T1"
## [1] "YPDR1T2"
## [1] "YPDR1T3"
## [1] "YPDR2T1"
## [1] "YPDR2T2"
## [1] "YPDR2T3"
## [1] "YPDR3T1"
## [1] "YPDR3T2"
## [1] "YPDR3T3"
## [1] "YPDR4T1"
## [1] "YPDR4T2"
## [1] "YPDR4T3"
## [1] "Batch2T0"
```

Plot data before and after subtracting chimeric reads for each time point in each pool and save new count tables

```
subtract_BG = function(temp,column){
  
  #fit line to non-existent DBCs
  fit_non = coef(lm(Observed~Expected+0,data=temp,presence=="no"))
  print("slope/total counts (before chimera removal)/total counts (after chimera removal)")
  print(unlist(fit_non[1]))
     
  #make data frame with just strains that exsit in the pool
  sub_temp = temp[which(temp$presence == "yes"),]
  sub_temp$norm = NA
  
  #plot before correction
  p = ggplot(temp)+geom_point(aes(x=Expected,y=Observed,shape=presence,color=category),alpha=0.2)+
    theme(legend.position="none")+ylim(0,6000)+
    geom_abline(slope=fit_non[1])#+xlim(0,4e10)
  print(p+ggtitle(paste(names(dat5)[column]," Before")))
  
  #loop through each existent strain and correct for chimeras by subtracting non-existent value for that x-value
  for (i in 1:dim(sub_temp)[1]){
    sub_temp$norm[i]=sub_temp$Observed[i]-(as.numeric(fit_non[1])*sub_temp$Expected[i])
  }
  
  #print how many counts were subtracted
  print(sum(sub_temp$Observed))
  print(sum(sub_temp$norm))
  
  #return normalized data for fitness calculation
  return(sub_temp) 
}

#make dataframes to store bc counts before and after chimera removal
#only include strains which exist in the pool
dat5_unnorm = dat5[-which(key$present=="no"),1:no_libs]
dat5_norm = data.frame(matrix(nrow=dim(dat5_unnorm)[1],ncol=no_libs))
names(dat5_norm)=names(dat5_unnorm)
rownames(dat5_norm) = rownames(dat5_unnorm)

#loop through each library and subtract chimeras, plot data after subtraction
for (column in 1:no_libs){
  print(names(dat5)[column])
  temp = data.frame(dat5[,column],dat5_expected[,column],key$present,key$category)
  names(temp)=c("Observed","Expected","presence","category")
  rownames(temp)=rownames(dat5)
  temp2=subtract_BG(temp,column)
  p = ggplot()+geom_point(aes(x=temp$Expected,y=temp$Observed,color=temp$presence),alpha=0.2)+
    geom_point(aes(x=temp2$Expected,y=temp2$norm),shape=1,alpha=0.1)+
    theme(legend.position="none")+ylim(0,4000)+xlim(0,max(temp2$Expected))+
    xlab("Expected")+ylab("Observed")
  print(p+ggtitle(paste(names(dat5)[column],"After")))
  
  dat5_norm[,column]=temp2$norm
  
  }
```

```
## [1] "Batch1T0"
## [1] "slope/total counts (before chimera removal)/total counts (after chimera removal)"
##     Expected 
## 1.470344e-09
```

```
## [1] 6764283
## [1] 6697975
```

```
## Warning: Removed 7 rows containing missing values (geom_point).
```

```
## [1] "YPDR1T1"
## [1] "slope/total counts (before chimera removal)/total counts (after chimera removal)"
##     Expected 
## 5.546826e-09
```

```
## [1] 9239402
## [1] 8772261
```

```
## Warning: Removed 4 rows containing missing values (geom_point).
```

```
## [1] "YPDR1T2"
## [1] "slope/total counts (before chimera removal)/total counts (after chimera removal)"
##     Expected 
## 1.339322e-08
```

```
## [1] 6706965
## [1] 6114818
```

```
## Warning: Removed 5 rows containing missing values (geom_point).
```

```
## [1] "YPDR1T3"
## [1] "slope/total counts (before chimera removal)/total counts (after chimera removal)"
##     Expected 
## 9.850094e-09
```

```
## [1] 6376256
## [1] 5990272
```

```
## Warning: Removed 3 rows containing missing values (geom_point).
```

```
## [1] "YPDR2T1"
## [1] "slope/total counts (before chimera removal)/total counts (after chimera removal)"
##     Expected 
## 8.874103e-09
```

```
## [1] 7027968
## [1] 6595381
```

```
## Warning: Removed 2 rows containing missing values (geom_point).
```

```
## [1] "YPDR2T2"
## [1] "slope/total counts (before chimera removal)/total counts (after chimera removal)"
##     Expected 
## 7.592738e-09
```

```
## [1] 8332955
## [1] 7815110
```

```
## Warning: Removed 4 rows containing missing values (geom_point).
```

```
## [1] "YPDR2T3"
## [1] "slope/total counts (before chimera removal)/total counts (after chimera removal)"
##     Expected 
## 3.467408e-09
```

```
## [1] 8552540
## [1] 8308616
```

```
## Warning: Removed 27 rows containing missing values (geom_point).
```

```
## Warning: Removed 32 rows containing missing values (geom_point).
```

```
## [1] "YPDR3T1"
## [1] "slope/total counts (before chimera removal)/total counts (after chimera removal)"
##     Expected 
## 1.249243e-08
```

```
## [1] 6912390
## [1] 6322898
```

```
## Warning: Removed 5 rows containing missing values (geom_point).
```

```
## [1] "YPDR3T2"
## [1] "slope/total counts (before chimera removal)/total counts (after chimera removal)"
##     Expected 
## 5.038186e-09
```

```
## [1] 6529006
## [1] 6318387
```

```
## Warning: Removed 7 rows containing missing values (geom_point).
```

```
## [1] "YPDR3T3"
## [1] "slope/total counts (before chimera removal)/total counts (after chimera removal)"
##     Expected 
## 7.204287e-09
```

```
## [1] 7708560
## [1] 7295702
```

```
## Warning: Removed 17 rows containing missing values (geom_point).
```

```
## Warning: Removed 14 rows containing missing values (geom_point).
```

```
## [1] "YPDR4T1"
## [1] "slope/total counts (before chimera removal)/total counts (after chimera removal)"
##     Expected 
## 2.623798e-08
```

```
## [1] 6893068
## [1] 5656734
```

```
## Warning: Removed 2 rows containing missing values (geom_point).
```

```
## [1] "YPDR4T2"
## [1] "slope/total counts (before chimera removal)/total counts (after chimera removal)"
##     Expected 
## 1.714573e-08
```

```
## [1] 6436305
## [1] 5735235
```

```
## Warning: Removed 4 rows containing missing values (geom_point).
```

```
## [1] "YPDR4T3"
## [1] "slope/total counts (before chimera removal)/total counts (after chimera removal)"
##    Expected 
## 1.67254e-08
```

```
## [1] 7056021
## [1] 6243978
```

```
## Warning: Removed 1 rows containing missing values (geom_point).
```

```
## Warning: Removed 6 rows containing missing values (geom_point).
```

```
## [1] "Batch2T0"
## [1] "slope/total counts (before chimera removal)/total counts (after chimera removal)"
##     Expected 
## 1.217239e-09
```

```
## [1] 7147676
## [1] 7086267
```

```
## Warning: Removed 7 rows containing missing values (geom_point).
```

Calculate fitness!!!
