# Supplementary material for: Improved discovery of genetic interactions using CRISPRiSeq across multiple environments
Source: Genome Res. 2019 Apr;29(4):668–81. doi: 10.1101/gr.246603.118 (PMC6442382; doi:10.1101/gr.246603.118)
Supplement: Supplemental Material [file supp_gr.246603.118_Supplemental_CRISPRiSeq-master.zip › CRISPRiSeq-master/processing_data/2_PCRchimera_removal_RMarkdown_files/seqlib13.html]

seqlib13


# seqlib13

```
require(ggplot2)
```

```
## Loading required package: ggplot2
```

```
require(reshape2)
```

```
## Loading required package: reshape2
```

```
theme_dbc <- theme_set(theme_gray())
theme_dbc <- theme_update(
  panel.background = element_rect(fill = "white"),
  panel.border = element_rect( colour = "black",fill=NA,size=2),
  panel.grid.major = element_line(colour = "gray93",size=1),
  panel.grid.minor = element_line(colour = "gray98",size=1),
  strip.text.x = element_text(size=12,face='bold'),
  axis.title = element_text(size=16),
  strip.background = element_rect(colour="black", fill="white",size = 1),
  axis.text = element_text(colour = "black",face="bold",size=16),
  axis.ticks=element_line(color="black",size=2))

dat = read.table(file="~/Desktop/SherlockLab2/System_test/screen2_hiseq/seqlib13_analysis/bc_counts/all_counts.txt", sep="\t",header=TRUE,row.names=1,stringsAsFactors = FALSE)
dat2 = read.table(file = "~/Desktop/SherlockLab2/System_test/screen2_hiseq/seqlib13_analysis/bc_counts/recovered_counts.txt", sep="\t",header=TRUE,row.names=1,stringsAsFactors = FALSE)
dat5 = dat+dat2

rm(dat)
rm(dat2)

key = read.table(file="~/Desktop/SherlockLab2/System_test/screen2_hiseq/data_table_row_key.txt",sep="\t",header=TRUE,stringsAsFactors = FALSE)

no_libs = 18
```

Look at counts for present barcodes vs not present barcodes

```
for(i in 1:25){
  p=ggplot()+geom_histogram(aes(x=dat5[which(key$present=="yes"),i]),alpha=0.5,binwidth=5,fill="green")+
    geom_histogram(aes(x=dat5[which(key$present=="no"),i]),alpha=0.5,binwidth=5,fill="blue")+ylim(0,500)+ggtitle(names(dat5)[i])
  print(p)
  }
```

Look at coverage across libraries

```
ggplot(melt(dat5))+geom_boxplot(aes(x=variable,y=value,color=variable))+
  theme(legend.position="none",axis.text.x=element_text(angle=60,hjust=1))
```

```
## No id variables; using all as measure variables
```

```
ggplot(melt(dat5))+geom_boxplot(aes(x=variable,y=value,color=variable))+
  theme(legend.position="none",axis.text.x=element_text(angle=60,hjust=1))+ylim(0,500)
```

```
## No id variables; using all as measure variables
```

```
## Warning: Removed 57847 rows containing non-finite values (stat_boxplot).
```

Look at representation of each query guide in starting pool

```
sub = dat5
sub$category = key$category
sub$query = key$query
sub = sub[which(key$present=="yes"),]
for(i in 1:no_libs){
  temp = sub[,c(i,26,27)]
  names(temp)[1]="count"
  p=ggplot(temp)+geom_boxplot(aes(x=query,y=count,color=query))+ggtitle(names(dat5)[i])+
    theme(legend.position="none",axis.text.x = element_text(angle = 60, hjust = 1))+scale_y_log10()
  print(p+facet_wrap(~category))
  print(names(dat5)[i])
  print(length(which(temp$count==0)))
  print(rownames(sub)[which(temp$count==0)])
}
```

```
## Warning: Removed 3 rows containing non-finite values (stat_boxplot).
```

```
## [1] "X37YPDR1T1"
## [1] 3
## [1] "PRE7g7-RFC5-NRg-2"  "DIP2g5-RFC5-NRg-2"  "SAP30g7-RFC5-NRg-2"
```

```
## Warning: Removed 7 rows containing non-finite values (stat_boxplot).
```

```
## [1] "X37YPDR1T2"
## [1] 7
## [1] "PRE4g9-RFC5-NRg-2"  "SEC22g1-CCT8-TRg-2" "GET2g2-CCT8-TRg-2" 
## [4] "DIP2g5-MSN5-TRg-7"  "DIP2g5-CCT8-TRg-2"  "DIP2g5-RFC5-NRg-2" 
## [7] "TIF6g8-RFC5-NRg-2"
```

```
## Warning: Removed 12 rows containing non-finite values (stat_boxplot).
```

```
## [1] "X37YPDR1T3"
## [1] 12
##  [1] "PRE7g7-MSN5-TRg-7"    "PRE4g9-MSN5-TRg-7"    "COG3g1-CCT8-TRg-2"   
##  [4] "GET2g2-MSN5-TRg-7"    "IMP4g6-CDC42-TRg-6"   "DIP2g5-MSN5-TRg-7"   
##  [7] "DIP2g5-CCT8-TRg-2"    "DIP2g5-GLC7-TRg-2"    "DIP2g5-RFC5-NRg-2"   
## [10] "TIF6g8-CCT8-TRg-2"    "TIF6g8-RFC5-NRg-2"    "YLR050Cg1-CCT8-TRg-2"
```

```
## Warning: Removed 4 rows containing non-finite values (stat_boxplot).
```

```
## [1] "X37YPDR2T1"
## [1] 4
## [1] "PRE7g7-RFC5-NRg-2" "COG3g1-RFC5-NRg-2" "DIP2g5-RFC5-NRg-2"
## [4] "TIF6g8-RFC5-NRg-2"
```

```
## Warning: Removed 2 rows containing non-finite values (stat_boxplot).
```

```
## [1] "X37YPDR2T2"
## [1] 2
## [1] "DIP2g5-RFC5-NRg-2" "TIF6g8-RFC5-NRg-2"
```

```
## Warning: Removed 12 rows containing non-finite values (stat_boxplot).
```

```
## [1] "X37YPDR2T3"
## [1] 12
##  [1] "PRE7g7-RFC5-NRg-2"     "PRE4g3-CCT8-TRg-2"    
##  [3] "COG3g1-RFC5-NRg-2"     "SEC22g1-CCT8-TRg-2"   
##  [5] "SEC22g1-RFC5-NRg-2"    "SEC22g2-CCT8-TRg-2"   
##  [7] "GET2g2-CCT8-TRg-2"     "GET2g2-RFC5-NRg-2"    
##  [9] "IMP4g6-CCT8-TRg-2"     "PWP2g2_BC1-MSN5-TRg-7"
## [11] "TIF6g8-RFC5-NRg-2"     "YLR050Cg1-MSN5-TRg-7"
```

```
## Warning: Removed 4 rows containing non-finite values (stat_boxplot).
```

```
## [1] "X37YPDR3T1"
## [1] 4
## [1] "PRE4g9-RFC5-NRg-2" "COG3g1-RFC5-NRg-2" "SED5g5-RFC5-NRg-2"
## [4] "DIP2g5-RFC5-NRg-2"
```

```
## Warning: Removed 6 rows containing non-finite values (stat_boxplot).
```

```
## [1] "X37YPDR3T2"
## [1] 6
## [1] "PRE7g7-RFC5-NRg-2"  "SED5g5-RFC5-NRg-2"  "IMP4g6-MSN5-TRg-7" 
## [4] "DIP2g5-RFC5-NRg-2"  "TIF6g8-RFC5-NRg-2"  "SAP30g7-RFC5-NRg-2"
```

```
## Warning: Removed 15 rows containing non-finite values (stat_boxplot).
```

```
## [1] "X37YPDR3T3"
## [1] 15
##  [1] "PRE7g7-RFC5-NRg-2"     "PRE4g9-RFC5-NRg-2"    
##  [3] "PRE4g3-CCT8-TRg-2"     "COG3g1-MSN5-TRg-7"    
##  [5] "COG3g1-RFC5-NRg-2"     "SED5g5-MSN5-TRg-7"    
##  [7] "SEC22g1-MSN5-TRg-7"    "SEC22g1-RFC5-NRg-2"   
##  [9] "GET2g2-MSN5-TRg-7"     "GET2g2-CCT8-TRg-2"    
## [11] "DIP2g5-RFC5-NRg-2"     "PWP2g2_BC2-CCT8-TRg-2"
## [13] "TIF6g8-MSN5-TRg-7"     "TIF6g8-RFC5-NRg-2"    
## [15] "YLR050Cg1-CCT8-TRg-2"
```

```
## Warning: Removed 4 rows containing non-finite values (stat_boxplot).
```

```
## [1] "X48hrR1T1"
## [1] 4
## [1] "PRE4g9-RFC5-NRg-2"  "DIP2g5-RFC5-NRg-2"  "TIF6g8-RFC5-NRg-2" 
## [4] "SAP30g7-RFC5-NRg-2"
```

```
## Warning: Removed 5 rows containing non-finite values (stat_boxplot).
```

```
## [1] "X48hrR1T2"
## [1] 5
## [1] "COG3g1-RFC5-NRg-2"  "SED5g5-RFC5-NRg-2"  "IMP4g6-RPP1-NRg-1" 
## [4] "IMP4g6-CDC42-TRg-6" "DIP2g5-RFC5-NRg-2"
```

```
## Warning: Removed 173 rows containing non-finite values (stat_boxplot).
```

```
## [1] "X48hrR1T3"
## [1] 173
##   [1] "PRE7g7-MNE1-TRg-5"      "PRE7g7-ARP2-NRg-3"     
##   [3] "PRE7g7-RPS2-NRg-11"     "PRE7g7-LST8-NRg-1"     
##   [5] "PRE7g7-GLC7-TRg-2"      "PRE7g7-SEC22-NRg-8"    
##   [7] "PRE7g7-SEC13-NRg-5"     "PRE7g7-CDC42-TRg-6"    
##   [9] "PRE7g7-ARC35-TRg-5"     "PRE7g7-RPS15-NRg-3"    
##  [11] "PRE7g7-RPL5-NRg-4"      "PRE7g7-SEC31-NRg-1"    
##  [13] "PRE7g7-ASK1-TRg-7"      "PRE7g7-NEO1-NRg-3"     
##  [15] "PRE7g7-PTI1-TRg-3"      "PRE7g7-PRP22-TRg-3"    
##  [17] "PRE7g7-VPS53-NRg-1"     "PRE7g7-CYR1-TRg-4"     
##  [19] "PRE7g7-MPS1-NRg-1"      "PRE7g7-RFC5-NRg-2"     
##  [21] "PRE7g7-SPC97-NRg-6"     "PRE7g7-COG1-TRg-3"     
##  [23] "PRE4g9-ISA1-TRg-15"     "PRE4g9-SEC31-NRg-1"    
##  [25] "PRE4g9-RFC5-NRg-2"      "PRE4g9-SPC97-NRg-6"    
##  [27] "RPN5g1-GLC7-TRg-2"      "RPN5g1-RPL30-NRg-5"    
##  [29] "RPN5g1-NEO1-NRg-3"      "RPN5g1-RFC5-NRg-2"     
##  [31] "RPN5g1-SEC16-TRg-7"     "COG3g1-MNE1-TRg-5"     
##  [33] "COG3g1-SCD5-TRg-5"      "COG3g1-CDC3-NRg-3"     
##  [35] "COG3g1-AFG2-TRg-1"      "COG3g1-RPS15-NRg-3"    
##  [37] "COG3g1-DAM1-TRg-2"      "COG3g1-ASK1-TRg-7"     
##  [39] "COG3g1-SPC97-NRg-5"     "COG3g1-DAD2-TRg-2"     
##  [41] "COG3g1-RFC5-NRg-2"      "COG3g1-SEC16-TRg-7"    
##  [43] "COG3g1-SPC97-NRg-6"     "COG3g1-REB1-NRg-4"     
##  [45] "SED5g5-GLC7-TRg-2"      "SED5g5-PCF11-TRg-4"    
##  [47] "SED5g5-SEC31-NRg-1"     "SED5g5-TAF12-TRg-2"    
##  [49] "SED5g5-RFC5-NRg-2"      "SEC22g1-SEC27-TRg-2"   
##  [51] "SEC22g1-CDC25-TRg-9"    "SEC22g1-CCT8-TRg-2"    
##  [53] "SEC22g1-GLC7-TRg-2"     "SEC22g1-SEC13-NRg-5"   
##  [55] "SEC22g1-CDC42-TRg-6"    "SEC22g1-DAM1-TRg-2"    
##  [57] "SEC22g1-GAL11-NRg-10"   "SEC22g1-COG1-NRg-1"    
##  [59] "SEC22g1-SEC16-TRg-7"    "SEC22g1-SEC27-TRg-4"   
##  [61] "SEC22g2-CCT8-TRg-2"     "SEC22g2-SEC18-NRg-2"   
##  [63] "SEC22g2-SEC27-TRg-4"    "GET2g2-PMI40-NRg-1"    
##  [65] "GET2g2-PAH1-TRg-7"      "GET2g2-MSN5-TRg-7"     
##  [67] "GET2g2-GPI11-TRg-3"     "GET2g2-MNE1-TRg-5"     
##  [69] "GET2g2-CCT8-TRg-2"      "GET2g2-NRD1-NRg-10"    
##  [71] "GET2g2-RET2-TRg-4"      "GET2g2-ERG25-TA-18"    
##  [73] "GET2g2-RAP1-NRg-5"      "GET2g2-TOA2-NRg-1"     
##  [75] "GET2g2-SEC18-TRg-5"     "GET2g2-BIG1-TRg-4"     
##  [77] "GET2g2-VPS45-NRg-9"     "GET2g2-SAR1-NRg-4"     
##  [79] "GET2g2-DOA4-NRg-2"      "GET2g2-GLC7-TRg-2"     
##  [81] "GET2g2-SEC22-NRg-8"     "GET2g2-SSS1-TRg-2"     
##  [83] "GET2g2-CDC3-TRg-4"      "GET2g2-BBP1-NRg-1"     
##  [85] "GET2g2-BBP1-TRg-1"      "GET2g2-RVB1-TRg-2"     
##  [87] "GET2g2-SEC24-TRg-2"     "GET2g2-ACT1-NRg-6"     
##  [89] "GET2g2-POB3-TRg-3"      "GET2g2-CDC42-TRg-6"    
##  [91] "GET2g2-ARC35-TRg-2"     "GET2g2-ARC35-TRg-5"    
##  [93] "GET2g2-BCP1-NRg-5"      "GET2g2-PFS2-TRg-3"     
##  [95] "GET2g2-SEC65-TRg-2"     "GET2g2-PCF11-TRg-6"    
##  [97] "GET2g2-SEC31-NRg-1"     "GET2g2-ASK1-TRg-7"     
##  [99] "GET2g2-NEO1-NRg-3"      "GET2g2-GAL11-TRg-5"    
## [101] "GET2g2-GAL11-NRg-10"    "GET2g2-GPI15-TRg-1"    
## [103] "GET2g2-ALG14-NRg-4"     "GET2g2-TFA2-TRg-4"     
## [105] "GET2g2-TAF12-TRg-2"     "GET2g2-TAF12-TRg-3"    
## [107] "GET2g2-TFB2-NRg-4"      "GET2g2-RFC5-NRg-2"     
## [109] "GET2g2-SCC2-TRg-1"      "GET2g2-SEC16-TRg-7"    
## [111] "GET2g2-TAO3-TRg-8"      "GET2g2-RPT1-NRg-6"     
## [113] "GET2g2-RPN6-TRg-3"      "IMP4g6-ARP2-NRg-3"     
## [115] "IMP4g6-CDC42-TRg-6"     "IMP4g6-ARC35-TRg-5"    
## [117] "IMP4g6-TIF5-NRg-9"      "IMP4g6-PCF11-TRg-5"    
## [119] "IMP4g6-ASK1-TRg-7"      "IMP4g6-SPC97-NRg-5"    
## [121] "IMP4g6-RFC5-NRg-2"      "IMP4g6-SCC2-TRg-1"     
## [123] "IMP4g6-SPC97-NRg-6"     "IMP4g6-SEC27-TRg-4"    
## [125] "DIP2g5-SEC27-TRg-2"     "DIP2g5-PAH1-TRg-6"     
## [127] "DIP2g5-CDC25-TRg-9"     "DIP2g5-ARP2-NRg-3"     
## [129] "DIP2g5-SEC18-NRg-1"     "DIP2g5-SAR1-NRg-4"     
## [131] "DIP2g5-TAF4-NRg-1"      "DIP2g5-NSL1-NRg-3"     
## [133] "DIP2g5-ISA1-TRg-15"     "DIP2g5-SEC13-NRg-5"    
## [135] "DIP2g5-CDC42-TRg-6"     "DIP2g5-PCF11-TRg-6"    
## [137] "DIP2g5-ASK1-TRg-7"      "DIP2g5-NEO1-NRg-3"     
## [139] "DIP2g5-REB1-TRg-6"      "DIP2g5-CYR1-TRg-7"     
## [141] "DIP2g5-RFC5-NRg-2"      "DIP2g5-DOP1-NRg-4"     
## [143] "DIP2g5-SPC97-NRg-6"     "DIP2g5-RPN3-TRg-6"     
## [145] "DIP2g5-RPT1-NRg-6"      "DIP2g5-SMX3-TRg-1"     
## [147] "PWP2g2_BC1-CDC25-TRg-9" "PWP2g2_BC1-ARP2-NRg-3" 
## [149] "PWP2g2_BC1-SEC18-NRg-1" "PWP2g2_BC1-GLC7-TRg-2" 
## [151] "PWP2g2_BC1-ISA1-TRg-15" "PWP2g2_BC1-SEC13-NRg-5"
## [153] "PWP2g2_BC1-ASK1-TRg-7"  "PWP2g2_BC1-NEO1-NRg-3" 
## [155] "PWP2g2_BC1-SPC97-NRg-5" "PWP2g2_BC1-TAF12-TRg-3"
## [157] "PWP2g2_BC1-SPC97-NRg-6" "PWP2g2_BC1-SEC27-TRg-4"
## [159] "TIF6g8-SEC27-TRg-2"     "TIF6g8-ARP2-NRg-3"     
## [161] "TIF6g8-DAM1-TRg-2"      "TIF6g8-SRP1-TRg-4"     
## [163] "TIF6g8-RFC5-NRg-2"      "TIF6g8-RPN6-TRg-3"     
## [165] "RPF1g3-GLC7-TRg-2"      "RPF1g3-CDC42-TRg-6"    
## [167] "RPF1g3-RFC5-NRg-2"      "RPF1g3-SEC27-TRg-4"    
## [169] "MAK16g1-ARP2-NRg-3"     "MAK16g1-DAM1-TRg-2"    
## [171] "YCR016Wg4-ARC35-TRg-5"  "YLR050Cg1-SPC97-NRg-5" 
## [173] "YLR050Cg1-SEC16-TRg-7"
```

```
## Warning: Removed 7 rows containing non-finite values (stat_boxplot).
```

```
## [1] "X48hrR2T1"
## [1] 7
## [1] "PRE7g7-RFC5-NRg-2"    "PRE4g9-RFC5-NRg-2"    "COG3g1-RFC5-NRg-2"   
## [4] "SED5g5-RFC5-NRg-2"    "DIP2g5-RFC5-NRg-2"    "TIF6g8-RFC5-NRg-2"   
## [7] "YLR050Cg1-RFC5-NRg-2"
```

```
## Warning: Removed 5 rows containing non-finite values (stat_boxplot).
```

```
## [1] "X48hrR2T2"
## [1] 5
## [1] "PRE7g7-RFC5-NRg-2" "COG3g1-RFC5-NRg-2" "GET2g2-CCT8-TRg-2"
## [4] "DIP2g5-RFC5-NRg-2" "TIF6g8-RFC5-NRg-2"
```

```
## Warning: Removed 161 rows containing non-finite values (stat_boxplot).
```

```
## Warning: Removed 2 rows containing non-finite values (stat_boxplot).
```

```
## [1] "X48hrR2T3"
## [1] 163
##   [1] "CC16-CCT8-TRg-2"        "CC16-ARC35-TRg-5"      
##   [3] "PRE7g7-LST8-NRg-1"      "PRE7g7-BBP1-TRg-1"     
##   [5] "PRE7g7-SEC10-NRg-1"     "PRE7g7-PRP9-TRg-3"     
##   [7] "PRE7g7-CDC42-TRg-6"     "PRE7g7-ARC35-TRg-5"    
##   [9] "PRE7g7-STU2-TRg-3"      "PRE7g7-PCF11-TRg-6"    
##  [11] "PRE7g7-SEC31-NRg-1"     "PRE7g7-ASK1-TRg-7"     
##  [13] "PRE7g7-NEO1-NRg-3"      "PRE7g7-SPC97-TRg-3"    
##  [15] "PRE7g7-KAP95-TRg-8"     "PRE7g7-ACO1-TRg-6"     
##  [17] "PRE7g7-PRP22-TRg-3"     "PRE7g7-CDC20-TRg-6"    
##  [19] "PRE7g7-RFC5-NRg-2"      "PRE7g7-SPC97-NRg-6"    
##  [21] "PRE7g7-SEC27-TRg-4"     "PRE4g9-SEC27-TRg-2"    
##  [23] "PRE4g9-CDC42-TRg-6"     "PRE4g9-ASK1-TRg-7"     
##  [25] "PRE4g9-RFC5-NRg-2"      "PRE4g3-CDC20-TRg-6"    
##  [27] "RPN5g1-MSN5-TRg-7"      "RPN5g1-GLC7-TRg-2"     
##  [29] "RPN5g1-SPC97-NRg-5"     "RPN5g1-RFC5-NRg-2"     
##  [31] "RPN5g1-SEC16-TRg-7"     "RPN5g1-SEC27-TRg-4"    
##  [33] "COG3g1-MSN5-TRg-7"      "COG3g1-MNE1-TRg-5"     
##  [35] "COG3g1-CCT8-TRg-2"      "COG3g1-YEF3-NRg-9"     
##  [37] "COG3g1-SEC24-TRg-2"     "COG3g1-SEC13-NRg-5"    
##  [39] "COG3g1-CDC42-TRg-6"     "COG3g1-ARC35-TRg-5"    
##  [41] "COG3g1-NAB3-TRg-3"      "COG3g1-NEO1-NRg-3"     
##  [43] "COG3g1-PAN1-TRg-4"      "COG3g1-SRP1-TRg-5"     
##  [45] "COG3g1-RFC5-NRg-2"      "COG3g1-SEC16-TRg-7"    
##  [47] "COG3g1-REB1-TRg-4"      "COG3g1-SEC27-TRg-4"    
##  [49] "COG3g1-EPL1-TRg-5"      "SED5g5-ARP2-NRg-3"     
##  [51] "SED5g5-SEC18-NRg-1"     "SED5g5-NSL1-NRg-3"     
##  [53] "SED5g5-SRP1-TRg-5"      "SED5g5-TAF12-TRg-2"    
##  [55] "SED5g5-RFC5-NRg-2"      "SED5g5-SPC97-NRg-6"    
##  [57] "SEC22g1-SEC27-TRg-2"    "SEC22g1-BET1-NRg-1"    
##  [59] "SEC22g1-CDC42-TRg-6"    "SEC22g1-SPC97-TRg-3"   
##  [61] "SEC22g1-GAL11-NRg-10"   "SEC22g1-TAF12-TRg-3"   
##  [63] "SEC22g1-RFC5-NRg-2"     "SEC22g1-SEC16-TRg-7"   
##  [65] "SEC22g2-CCT8-TRg-2"     "SEC22g2-CDC42-TRg-6"   
##  [67] "SEC22g2-ARC35-TRg-5"    "SEC22g2-MPS1-NRg-1"    
##  [69] "SEC22g2-RFC5-NRg-2"     "SEC22g2-COG1-TRg-3"    
##  [71] "GET2g2-PAH1-TRg-6"      "GET2g2-MSN5-TRg-7"     
##  [73] "GET2g2-ATP15-NRg-5"     "GET2g2-CCT8-TRg-2"     
##  [75] "GET2g2-ERG25-TA-18"     "GET2g2-SPN1-NRg-1"     
##  [77] "GET2g2-SEC18-NRg-1"     "GET2g2-CLF1-NRg-2"     
##  [79] "GET2g2-GLC7-TRg-2"      "GET2g2-SEC22-NRg-8"    
##  [81] "GET2g2-SNF6-TRg-6"      "GET2g2-SEC13-NRg-5"    
##  [83] "GET2g2-ARC35-TRg-5"     "GET2g2-SED5-TRg-6"     
##  [85] "GET2g2-SEC31-NRg-1"     "GET2g2-ASK1-TRg-7"     
##  [87] "GET2g2-NEO1-NRg-3"      "GET2g2-REB1-TRg-6"     
##  [89] "GET2g2-SPC97-TRg-3"     "GET2g2-RPL3-NRg-2"     
##  [91] "GET2g2-NUP145-NRg-4"    "GET2g2-COG4-TRg-1"     
##  [93] "GET2g2-RHO1-NRg-2"      "GET2g2-MPS1-NRg-1"     
##  [95] "GET2g2-TAF12-TRg-2"     "GET2g2-SCM3-TRg-4"     
##  [97] "GET2g2-RFC5-NRg-2"      "GET2g2-CDC48-NRg-9"    
##  [99] "GET2g2-RPT5-TRg-8"      "IMP4g6-ARP2-NRg-3"     
## [101] "IMP4g6-GLC7-TRg-2"      "IMP4g6-ARC35-TRg-5"    
## [103] "IMP4g6-TIF5-NRg-9"      "IMP4g6-TAF12-TRg-3"    
## [105] "IMP4g6-TFB2-NRg-4"      "IMP4g6-SPC97-NRg-6"    
## [107] "DIP2g5-SEC27-TRg-2"     "DIP2g5-PAH1-TRg-5"     
## [109] "DIP2g5-ARP2-NRg-3"      "DIP2g5-SCD5-TRg-5"     
## [111] "DIP2g5-CCT8-TRg-2"      "DIP2g5-YEF3-NRg-9"     
## [113] "DIP2g5-NSL1-NRg-3"      "DIP2g5-SEC24-TRg-2"    
## [115] "DIP2g5-CDC42-TRg-6"     "DIP2g5-YPT6-NRg-2"     
## [117] "DIP2g5-NAB3-TRg-3"      "DIP2g5-TOA2-TRg-3"     
## [119] "DIP2g5-DAM1-TRg-2"      "DIP2g5-RPL5-NRg-4"     
## [121] "DIP2g5-PCF11-TRg-6"     "DIP2g5-SEC31-NRg-1"    
## [123] "DIP2g5-ASK1-TRg-7"      "DIP2g5-PTI1-TRg-3"     
## [125] "DIP2g5-SPC97-NRg-5"     "DIP2g5-RPL3-NRg-2"     
## [127] "DIP2g5-RHO1-NRg-2"      "DIP2g5-PAN1-NRg-1"     
## [129] "DIP2g5-CYR1-TRg-4"      "DIP2g5-RFC5-NRg-2"     
## [131] "DIP2g5-RPN3-TRg-6"      "DIP2g5-SEC27-TRg-4"    
## [133] "PWP2g2_BC1-SEC27-TRg-2" "PWP2g2_BC1-YEF3-NRg-9" 
## [135] "PWP2g2_BC1-SEC13-NRg-5" "PWP2g2_BC1-ARC35-TRg-5"
## [137] "PWP2g2_BC1-CYR1-TRg-7"  "PWP2g2_BC1-SEC16-TRg-7"
## [139] "PWP2g2_BC1-TEN1-NRg-2"  "PWP2g2_BC1-COG1-TRg-3" 
## [141] "PWP2g2_BC2-GLC7-TRg-2"  "TIF6g8-SEC27-TRg-2"    
## [143] "TIF6g8-MSN5-TRg-7"      "TIF6g8-SEC24-TRg-2"    
## [145] "TIF6g8-ARC35-TRg-5"     "TIF6g8-DAM1-TRg-2"     
## [147] "TIF6g8-SEC31-NRg-1"     "TIF6g8-NEO1-NRg-3"     
## [149] "TIF6g8-DAD2-TRg-2"      "TIF6g8-CYR1-TRg-7"     
## [151] "TIF6g8-TAF12-TRg-2"     "TIF6g8-RFC5-NRg-2"     
## [153] "TIF6g8-SEC16-TRg-7"     "TIF6g8-SPC97-NRg-6"    
## [155] "RPF1g3-ASK1-TRg-7"      "MAK16g1-GLC7-TRg-2"    
## [157] "MAK16g1-SPC97-NRg-5"    "MAK16g1-RHO1-NRg-2"    
## [159] "YCR016Wg4-GLC7-TRg-2"   "YLR050Cg1-ASK1-TRg-7"  
## [161] "YLR050Cg1-DAD2-TRg-2"   "YLR050Cg1-RFC5-NRg-2"  
## [163] "YLR050Cg1-SPC97-NRg-6"
```

```
## Warning: Removed 5 rows containing non-finite values (stat_boxplot).
```

```
## [1] "X48hrR3T1"
## [1] 5
## [1] "PRE4g9-RFC5-NRg-2" "COG3g1-RFC5-NRg-2" "SED5g5-RFC5-NRg-2"
## [4] "DIP2g5-RFC5-NRg-2" "TIF6g8-RFC5-NRg-2"
```

```
## Warning: Removed 8 rows containing non-finite values (stat_boxplot).
```

```
## [1] "X48hrR3T2"
## [1] 8
## [1] "PRE7g7-RFC5-NRg-2"  "PRE4g9-RFC5-NRg-2"  "COG3g1-RFC5-NRg-2" 
## [4] "SED5g5-RFC5-NRg-2"  "GET2g2-CCT8-TRg-2"  "DIP2g5-RFC5-NRg-2" 
## [7] "TIF6g8-RFC5-NRg-2"  "SAP30g7-RFC5-NRg-2"
```

```
## Warning: Removed 151 rows containing non-finite values (stat_boxplot).
```

```
## Warning: Removed 2 rows containing non-finite values (stat_boxplot).
```

```
## [1] "X48hrR3T3"
## [1] 153
##   [1] "GET2g2-CC32"            "CC17-SEC13-NRg-5"      
##   [3] "PRE7g7-SEC27-TRg-2"     "PRE7g7-MSN5-TRg-7"     
##   [5] "PRE7g7-RPL25-NRg-1"     "PRE7g7-BBP1-TRg-1"     
##   [7] "PRE7g7-CDC42-TRg-6"     "PRE7g7-PRE3-TRg-4"     
##   [9] "PRE7g7-PCF11-TRg-4"     "PRE7g7-ASK1-TRg-7"     
##  [11] "PRE7g7-RHO1-NRg-2"      "PRE7g7-CDC20-TRg-6"    
##  [13] "PRE7g7-SRP1-TRg-4"      "PRE7g7-TAF12-TRg-2"    
##  [15] "PRE7g7-RFC5-NRg-2"      "PRE7g7-SPC97-NRg-6"    
##  [17] "PRE7g7-CDC48-TRg-5"     "PRE7g7-CKS1-TRg-5"     
##  [19] "PRE7g7-SEC27-TRg-4"     "PRE7g7-ERG9-NRg-7"     
##  [21] "PRE4g9-GLC7-TRg-2"      "PRE4g9-SEC10-NRg-1"    
##  [23] "PRE4g9-RFC5-NRg-2"      "PRE4g9-SEC16-TRg-7"    
##  [25] "PRE4g9-SEC27-TRg-4"     "RPN5g1-MSN5-TRg-7"     
##  [27] "RPN5g1-SSS1-TRg-2"      "RPN5g1-CDC42-TRg-6"    
##  [29] "RPN5g1-CDC20-TRg-6"     "RPN5g1-TAF12-TRg-3"    
##  [31] "RPN5g1-RFC5-NRg-2"      "RPN5g1-SCC2-TRg-1"     
##  [33] "COG3g1-MSN5-TRg-7"      "COG3g1-CCT8-TRg-2"     
##  [35] "COG3g1-ACT1-NRg-6"      "COG3g1-ARC35-TRg-5"    
##  [37] "COG3g1-PCF11-TRg-6"     "COG3g1-RRP45-TRg-4"    
##  [39] "COG3g1-RFC5-NRg-2"      "COG3g1-SEC16-TRg-7"    
##  [41] "COG3g1-SPC97-NRg-6"     "COG3g1-REB1-NRg-4"     
##  [43] "SED5g5-CDC25-TRg-9"     "SED5g5-BET1-NRg-1"     
##  [45] "SED5g5-SPN1-NRg-1"      "SED5g5-GLC7-TRg-2"     
##  [47] "SED5g5-DAM1-TRg-2"      "SED5g5-RFC5-NRg-2"     
##  [49] "SED5g5-SEC16-NRg-12"    "SEC22g1-SEC27-TRg-2"   
##  [51] "SEC22g1-CCT8-TRg-2"     "SEC22g1-SEC18-NRg-1"   
##  [53] "SEC22g1-SEC18-NRg-2"    "SEC22g1-CDC42-TRg-6"   
##  [55] "SEC22g1-PCF11-TRg-4"    "SEC22g1-ASK1-TRg-7"    
##  [57] "SEC22g1-SRP1-TRg-4"     "SEC22g1-TAF12-TRg-3"   
##  [59] "SEC22g1-SCC2-TRg-1"     "SEC22g1-SEC27-TRg-4"   
##  [61] "SEC22g2-MSN5-TRg-7"     "SEC22g2-CCT8-TRg-2"    
##  [63] "SEC22g2-ISA1-TRg-15"    "SEC22g2-ARC35-TRg-5"   
##  [65] "SEC22g2-SEC21-NRg-3"    "SEC22g2-COG1-NRg-1"    
##  [67] "COG8g2-GLC7-TRg-2"      "GET2g2-MSN5-TRg-7"     
##  [69] "GET2g2-MNE1-TRg-5"      "GET2g2-ARP2-NRg-3"     
##  [71] "GET2g2-TIF34-TRg-2"     "GET2g2-BET1-NRg-1"     
##  [73] "GET2g2-RPP1-NRg-1"      "GET2g2-RAP1-NRg-5"     
##  [75] "GET2g2-SEC10-NRg-1"     "GET2g2-NSL1-NRg-3"     
##  [77] "GET2g2-SEC24-TRg-2"     "GET2g2-SEC13-NRg-5"    
##  [79] "GET2g2-CDC42-TRg-6"     "GET2g2-DAM1-TRg-2"     
##  [81] "GET2g2-RPN12-NRg-11"    "GET2g2-RTP1-NRg-2"     
##  [83] "GET2g2-SEC31-NRg-1"     "GET2g2-SPC97-TRg-3"    
##  [85] "GET2g2-PTI1-TRg-5"      "GET2g2-UTP23-TRg-1"    
##  [87] "GET2g2-GAL11-NRg-10"    "GET2g2-RPB7-NRg-3"     
##  [89] "GET2g2-DIP2-TRg-1"      "GET2g2-TFA2-TRg-4"     
##  [91] "GET2g2-CYR1-TRg-7"      "GET2g2-MPS1-NRg-1"     
##  [93] "GET2g2-RFC5-NRg-2"      "GET2g2-SCC2-TRg-1"     
##  [95] "GET2g2-RPT1-TRg-5"      "GET2g2-SEC27-TRg-4"    
##  [97] "GET2g2-CDC48-NRg-6"     "IMP4g6-PCF11-TRg-5"    
##  [99] "IMP4g6-SPC97-NRg-5"     "IMP4g6-RPL3-NRg-2"     
## [101] "IMP4g6-SPC97-NRg-6"     "DIP2g5-SEC27-TRg-2"    
## [103] "DIP2g5-MSN5-TRg-7"      "DIP2g5-ARP2-NRg-3"     
## [105] "DIP2g5-SEC18-NRg-2"     "DIP2g5-GLC7-TRg-2"     
## [107] "DIP2g5-ISA1-TRg-15"     "DIP2g5-CDC42-TRg-6"    
## [109] "DIP2g5-ARC35-TRg-5"     "DIP2g5-RPL5-NRg-4"     
## [111] "DIP2g5-SEC31-NRg-1"     "DIP2g5-ASK1-TRg-7"     
## [113] "DIP2g5-CDC24-TRg-3"     "DIP2g5-CDC20-TRg-6"    
## [115] "DIP2g5-COG1-NRg-1"      "DIP2g5-CYR1-TRg-7"     
## [117] "DIP2g5-SRP1-TRg-5"      "DIP2g5-RFC5-NRg-2"     
## [119] "DIP2g5-SPC97-NRg-6"     "PWP2g2_BC1-CDC25-TRg-9"
## [121] "PWP2g2_BC1-CCT8-TRg-2"  "PWP2g2_BC1-SEC18-NRg-1"
## [123] "PWP2g2_BC1-SEC13-NRg-5" "PWP2g2_BC1-ARC35-TRg-5"
## [125] "PWP2g2_BC1-NAB3-TRg-3"  "PWP2g2_BC1-DAM1-TRg-2" 
## [127] "PWP2g2_BC1-PCF11-TRg-4" "PWP2g2_BC1-NEO1-NRg-3" 
## [129] "PWP2g2_BC1-SPC97-NRg-5" "PWP2g2_BC1-DAD2-TRg-2" 
## [131] "PWP2g2_BC1-SRP1-TRg-4"  "PWP2g2_BC1-TFB2-NRg-4" 
## [133] "PWP2g2_BC1-SPC97-NRg-6" "TIF6g8-CDC25-TRg-9"    
## [135] "TIF6g8-GLC7-TRg-2"      "TIF6g8-ARC35-TRg-5"    
## [137] "TIF6g8-SEC31-NRg-1"     "TIF6g8-RFC5-NRg-2"     
## [139] "TIF6g8-SEC16-TRg-7"     "RPF1g3-RHO1-NRg-2"     
## [141] "RPF1g3-TAF12-TRg-3"     "RPF1g3-RFC5-NRg-2"     
## [143] "MAK16g1-MSN5-TRg-7"     "MAK16g1-GLC7-TRg-2"    
## [145] "MAK16g1-DAM1-TRg-2"     "MAK16g1-SEC27-TRg-4"   
## [147] "YCR016Wg4-ARC35-TRg-5"  "YLR050Cg1-ARP2-NRg-3"  
## [149] "YLR050Cg1-DAD2-TRg-2"   "YLR050Cg1-SPC97-NRg-6" 
## [151] "SAP30g7-CCT8-TRg-2"     "SAP30g7-SEC31-NRg-1"   
## [153] "SAP30g7-RFC5-NRg-2"
```

Calculate and plot chimera subtraction

```
#store what BC1, BC2, and DBC is represented on each line of dat5
all_BC1 = key$query
all_BC2 = key$array
all_DBC = rownames(dat5)

#make data frame to store frequencies for each of BC1s in each samples
BC1_freqs = data.frame(matrix(nrow=length(unique(all_BC1)),ncol=no_libs))
names(BC1_freqs) = names(dat5)[1:no_libs]
BC1_freqs$BC1_name = unique(all_BC1)

#loop through BC1s and find total frequencies in each of the samples
for (i in 1:dim(BC1_freqs)[1]){
  for (j in 1:(dim(BC1_freqs)[2]-1)){
    BC1_freqs[i,j] = sum(dat5[which(all_BC1==BC1_freqs$BC1_name[i]),j])
    }
  }

#Make data frame to store frequencies for each of the BC2s in samples
BC2_freqs = data.frame(matrix(nrow=length(unique(all_BC2)),ncol=no_libs))
names(BC2_freqs)=names(dat5)[1:no_libs]
BC2_freqs$BC2_names = unique(all_BC2)

#loop through BC2s and find total frequencies in each of the samples
for (i in 1:dim(BC2_freqs)[1]){
  for (j in 1:(dim(BC2_freqs)[2]-1)){
    BC2_freqs[i,j] = sum(dat5[which(all_BC2==BC2_freqs$BC2_names[i]),j])
    }
  }
```

```
#Function to calculate expected number of counts at the given time point for each DBC
get_expected = function(sample){
  
  print(names(dat5)[sample])  
  temp = rep(NA,dim(dat5)[1])
  
  #calculate expected counts for each DBC at each time point
  for (i in 1:dim(dat5)[1]){      
    
    #expected frequency of DBC based on two single mutants
    BC1_i = which(BC1_freqs$BC1_name==all_BC1[i])
    BC1_count = as.numeric(BC1_freqs[BC1_i,sample])
    
    BC2_i = which(BC2_freqs$BC2_name==all_BC2[i])
    BC2_count = as.numeric(BC2_freqs[BC2_i,sample])
    
    temp[i]=BC1_count * BC2_count
    
    }
    return(temp)  
}

#Calculate expectation for all counts in data matrix
dat5_expected = data.frame(matrix(nrow=dim(dat5)[1],ncol=no_libs))
names(dat5_expected)=names(dat5)[1:no_libs]
rownames(dat5_expected)=rownames(dat5)
for(i in 1:no_libs){
  dat5_expected[,i] = get_expected(i)
}
```

```
## [1] "X37YPDR1T1"
## [1] "X37YPDR1T2"
## [1] "X37YPDR1T3"
## [1] "X37YPDR2T1"
## [1] "X37YPDR2T2"
## [1] "X37YPDR2T3"
## [1] "X37YPDR3T1"
## [1] "X37YPDR3T2"
## [1] "X37YPDR3T3"
## [1] "X48hrR1T1"
## [1] "X48hrR1T2"
## [1] "X48hrR1T3"
## [1] "X48hrR2T1"
## [1] "X48hrR2T2"
## [1] "X48hrR2T3"
## [1] "X48hrR3T1"
## [1] "X48hrR3T2"
## [1] "X48hrR3T3"
```

Plot data before and after subtracting chimeric reads for each time point in each pool and save new count tables

```
subtract_BG = function(temp,column){
  
  #fit line to non-existent DBCs
  fit_non = coef(lm(Observed~Expected+0,data=temp,presence=="no"))
  print("slope/total counts (before chimera removal)/total counts (after chimera removal)")
  print(unlist(fit_non[1]))
     
  #make data frame with just strains that exsit in the pool
  sub_temp = temp[which(temp$presence == "yes"),]
  sub_temp$norm = NA
  
  #plot before correction
  p = ggplot(temp)+geom_point(aes(x=Expected,y=Observed,shape=presence,color=category),alpha=0.2)+
    theme(legend.position="none")+ylim(0,14100)+
    geom_abline(slope=fit_non[1])#+xlim(0,4e10)
  print(p+ggtitle(paste(names(dat5)[column]," Before")))
  
  #loop through each existent strain and correct for chimeras by subtracting non-existent value for that x-value
  for (i in 1:dim(sub_temp)[1]){
    sub_temp$norm[i]=sub_temp$Observed[i]-(as.numeric(fit_non[1])*sub_temp$Expected[i])
  }
  
  #print how many counts were subtracted
  print(sum(sub_temp$Observed))
  print(sum(sub_temp$norm))
  
  #return normalized data for fitness calculation
  return(sub_temp) 
}

#make dataframes to store bc counts before and after chimera removal
#only include strains which exist in the pool
dat5_unnorm = dat5[-which(key$present=="no"),1:no_libs]
dat5_norm = data.frame(matrix(nrow=dim(dat5_unnorm)[1],ncol=no_libs))
names(dat5_norm)=names(dat5_unnorm)
rownames(dat5_norm) = rownames(dat5_unnorm)

#loop through each library and subtract chimeras, plot data after subtraction
for (column in 1:no_libs){
  print(names(dat5)[column])
  temp = data.frame(dat5[,column],dat5_expected[,column],key$present,key$category)
  names(temp)=c("Observed","Expected","presence","category")
  rownames(temp)=rownames(dat5)
  temp2=subtract_BG(temp,column)
  p = ggplot()+geom_point(aes(x=temp$Expected,y=temp$Observed,color=temp$presence),alpha=0.2)+
    geom_point(aes(x=temp2$Expected,y=temp2$norm),shape=1,alpha=0.1)+
    theme(legend.position="none")+ylim(0,14100)+xlim(0,max(temp2$Expected))+
    xlab("Expected")+ylab("Observed")
  print(p+ggtitle(paste(names(dat5)[column],"After")))
  
  dat5_norm[,column]=temp2$norm
  
  }
```

```
## [1] "X37YPDR1T1"
## [1] "slope/total counts (before chimera removal)/total counts (after chimera removal)"
##     Expected 
## 2.805312e-08
```

```
## [1] 5529954
## [1] 4679789
```

```
## Warning: Removed 5 rows containing missing values (geom_point).
```

```
## [1] "X37YPDR1T2"
## [1] "slope/total counts (before chimera removal)/total counts (after chimera removal)"
##     Expected 
## 1.769412e-08
```

```
## [1] 5166711
## [1] 4700014
```

```
## Warning: Removed 8 rows containing missing values (geom_point).
```

```
## [1] "X37YPDR1T3"
## [1] "slope/total counts (before chimera removal)/total counts (after chimera removal)"
##     Expected 
## 2.333898e-08
```

```
## [1] 5547797
## [1] 4840117
```

```
## Warning: Removed 14 rows containing missing values (geom_point).
```

```
## [1] "X37YPDR2T1"
## [1] "slope/total counts (before chimera removal)/total counts (after chimera removal)"
##     Expected 
## 1.737414e-08
```

```
## [1] 5815773
## [1] 5234051
```

```
## Warning: Removed 6 rows containing missing values (geom_point).
```

```
## [1] "X37YPDR2T2"
## [1] "slope/total counts (before chimera removal)/total counts (after chimera removal)"
##     Expected 
## 2.317307e-08
```

```
## [1] 5719825
## [1] 4969790
```

```
## Warning: Removed 3 rows containing missing values (geom_point).
```

```
## [1] "X37YPDR2T3"
## [1] "slope/total counts (before chimera removal)/total counts (after chimera removal)"
##     Expected 
## 2.133525e-08
```

```
## [1] 5732461
## [1] 5041758
```

```
## Warning: Removed 14 rows containing missing values (geom_point).
```

```
## [1] "X37YPDR3T1"
## [1] "slope/total counts (before chimera removal)/total counts (after chimera removal)"
##     Expected 
## 1.174484e-08
```

```
## [1] 5104882
## [1] 4802307
```

```
## Warning: Removed 4 rows containing missing values (geom_point).
```

```
## [1] "X37YPDR3T2"
## [1] "slope/total counts (before chimera removal)/total counts (after chimera removal)"
##     Expected 
## 1.126032e-08
```

```
## [1] 4676583
## [1] 4433653
```

```
## Warning: Removed 6 rows containing missing values (geom_point).
```

```
## [1] "X37YPDR3T3"
## [1] "slope/total counts (before chimera removal)/total counts (after chimera removal)"
##     Expected 
## 5.856923e-09
```

```
## [1] 4881775
## [1] 4744830
```

```
## Warning: Removed 15 rows containing missing values (geom_point).
```

```
## [1] "X48hrR1T1"
## [1] "slope/total counts (before chimera removal)/total counts (after chimera removal)"
##     Expected 
## 1.302035e-08
```

```
## [1] 5184212
## [1] 4838426
```

```
## Warning: Removed 4 rows containing missing values (geom_point).
```

```
## [1] "X48hrR1T2"
## [1] "slope/total counts (before chimera removal)/total counts (after chimera removal)"
##     Expected 
## 7.627967e-09
```

```
## [1] 5313812
## [1] 5104523
```

```
## Warning: Removed 6 rows containing missing values (geom_point).
```

```
## [1] "X48hrR1T3"
## [1] "slope/total counts (before chimera removal)/total counts (after chimera removal)"
##     Expected 
## 9.344047e-09
```

```
## [1] 5048994
## [1] 4830745
```

```
## Warning: Removed 178 rows containing missing values (geom_point).
```

```
## [1] "X48hrR2T1"
## [1] "slope/total counts (before chimera removal)/total counts (after chimera removal)"
##    Expected 
## 5.08851e-09
```

```
## [1] 5304844
## [1] 5163445
```

```
## Warning: Removed 7 rows containing missing values (geom_point).
```

```
## [1] "X48hrR2T2"
## [1] "slope/total counts (before chimera removal)/total counts (after chimera removal)"
##     Expected 
## 1.528185e-08
```

```
## [1] 5596422
## [1] 5128469
```

```
## Warning: Removed 7 rows containing missing values (geom_point).
```

```
## [1] "X48hrR2T3"
## [1] "slope/total counts (before chimera removal)/total counts (after chimera removal)"
##    Expected 
## 3.79131e-09
```

```
## [1] 6223656
## [1] 6089421
```

```
## Warning: Removed 167 rows containing missing values (geom_point).
```

```
## [1] "X48hrR3T1"
## [1] "slope/total counts (before chimera removal)/total counts (after chimera removal)"
##     Expected 
## 8.405392e-09
```

```
## [1] 5632071
## [1] 5368597
```

```
## Warning: Removed 6 rows containing missing values (geom_point).
```

```
## [1] "X48hrR3T2"
## [1] "slope/total counts (before chimera removal)/total counts (after chimera removal)"
##     Expected 
## 1.174376e-08
```

```
## [1] 4669597
## [1] 4420523
```

```
## Warning: Removed 8 rows containing missing values (geom_point).
```

```
## [1] "X48hrR3T3"
## [1] "slope/total counts (before chimera removal)/total counts (after chimera removal)"
##     Expected 
## 9.036795e-09
```

```
## [1] 4633894
## [1] 4456639
```

```
## Warning: Removed 157 rows containing missing values (geom_point).
```

Save matrixes of bc counts before and after chimera removal

```
#save(dat5_unnorm,file="~/Desktop/SherlockLab2/System_test/screen2_hiseq/seqlib13_analysis/seqlib13_raw_counts.RData")

#save(dat5_norm,file="~/Desktop/SherlockLab2/System_test/screen2_hiseq/seqlib13_analysis/seqlib13_chimera_normalized_counts.RData")
```

Also look at differences in slope for wt strain unlike last experiment some combos didnt exist, and the ones that did exist were at different expected values!

```
temp = dat5[1:100,1:18]
temp2 = dat5_expected[1:100,1:18]
for(i in 1:18){
  temp3 = data.frame(temp[,i],temp2[,i],key$present[1:100])
  names(temp3)=c("obs","exp","pres")
  print(ggplot(temp3,aes(x=exp,y=obs,color=pres))+geom_point()+ggtitle(names(dat5)[i]))
}
```

```
#non-existent strain with relatively high count is CC16-CC16
```

```
