# Supplementary material for: Improved discovery of genetic interactions using CRISPRiSeq across multiple environments
Source: Genome Res. 2019 Apr;29(4):668–81. doi: 10.1101/gr.246603.118 (PMC6442382; doi:10.1101/gr.246603.118)
Supplement: Supplemental Material [file supp_gr.246603.118_Supplemental_CRISPRiSeq-master.zip › CRISPRiSeq-master/processing_data/3_Compute_fit_GI_RMarkdown/fit_gi_v4.html]

v4


# v4

```
#load packages and set theme
require(ggplot2)
```

```
## Loading required package: ggplot2
```

```
require(reshape2)
```

```
## Loading required package: reshape2
```

```
theme_dbc <- theme_set(theme_gray())
theme_dbc <- theme_update(
  panel.background = element_rect(fill = "white"),
  panel.border = element_rect( colour = "black",fill=NA,size=2),
  panel.grid.major = element_line(colour = "gray93",size=1),
  panel.grid.minor = element_line(colour = "gray98",size=1),
  strip.text.x = element_text(size=12,face='bold'),
  axis.title = element_text(size=16),
  strip.background = element_rect(colour="black", fill="white",size = 1),
  axis.text = element_text(colour = "black",face="bold",size=16),
  axis.ticks=element_line(color="black",size=2))


#load in chimera normalized count data from 3 lanes of hiseq data (Seqlib11-13)
load("~/Desktop/SherlockLab2/manuscript_aug2017/code/processing_data/Data_R_counts/seqlib11_chimera_normalized_counts.RData")
ypd24 = dat5_norm[,2:10]
rm(dat5_norm)
load("~/Desktop/SherlockLab2/manuscript_aug2017/code/processing_data/Data_R_counts/seqlib12_chimera_normalized_counts.RData")
ypeg = dat5_norm[,1:9]
ura = dat5_norm[10:18]
rm(dat5_norm)
load("~/Desktop/SherlockLab2/manuscript_aug2017/code/processing_data/Data_R_counts/seqlib13_chimera_normalized_counts.RData")
ypd37 = dat5_norm[,1:9]
ypd48 = dat5_norm[,10:18]
rm(dat5_norm)

#load in row key for count data tables
key = read.table(file="~/Desktop/SherlockLab2/manuscript_aug2017/code/processing_data/row_key/data_table_row_key.txt",sep="\t",header=TRUE,stringsAsFactors = FALSE)
key = key[-which(key$present=="no"),]


#define constants
#thresholds chosen by looking at frequency trajectories
threshold1=5e-6 
threshold3=1e-6
#growth monitored by Coulter Counter (Beckman) during pooled fitness assay
#number of generations is estimated from cell density measurements at each transfer
ypd24_gen = c(0,1.5476,3.3255)
ypd48_gen = c(0,2.5839,5.5889)
ypeg_gen = c(0,1.4255,3.6308)
ypd37_gen = c(0,0.7439,3.0416)
ura_gen = c(0,1.1096,2.5880)
```

Clean up count data

```
#remove strains carrying 2 control guides with fitness defect (CC3, CC33)
arrays = gsub(".+-","",rownames(ypd24))
rem_i = which(arrays =="CC3"|arrays=="CC33")
rownames(ypd24)[rem_i]
```

```
##  [1] "PRE4g3-CC3"      "RPN5g1-CC3"      "SEC22g1-CC3"    
##  [4] "SEC22g2-CC3"     "COG8g2-CC3"      "GET2g2-CC3"     
##  [7] "PWP2g2_BC2-CC3"  "RPF1g3-CC3"      "MAK16g1-CC3"    
## [10] "YCR016Wg4-CC3"   "YLR050Cg1-CC3"   "SAP30g7-CC3"    
## [13] "CC8-CC3"         "CC16-CC3"        "CC17-CC3"       
## [16] "CC8_BC2-CC3"     "CC16_BC2-CC3"    "PRE4g3-CC33"    
## [19] "RPN5g1-CC33"     "SEC22g1-CC33"    "SEC22g2-CC33"   
## [22] "COG8g2-CC33"     "GET2g2-CC33"     "PWP2g2_BC2-CC33"
## [25] "RPF1g3-CC33"     "MAK16g1-CC33"    "YCR016Wg4-CC33" 
## [28] "YLR050Cg1-CC33"  "SAP30g7-CC33"    "CC8-CC33"       
## [31] "CC16-CC33"       "CC17-CC33"       "CC8_BC2-CC33"   
## [34] "CC16_BC2-CC33"
```

```
ypd24 = ypd24[-rem_i,]
ypd48 = ypd48[-rem_i,]
ypd37 = ypd37[-rem_i,]
ypeg = ypeg[-rem_i,]
ura = ura[-rem_i,]
key = key[-rem_i,]
```

Thresholds used: -T1 threshold of 5-e6 (~30-40 reads), and T3 threshold of 1e-6 (~5-8 reads) -Use these threshold for all categories of strains

Summary of workflow to estimate fitness for single mutants 1. Calculate frequency for each strain by dividing by total counts at for that replicate at that time point 2. Sum frequencies for all 100 WT strains 3. Set frequencies to 0 for all three time points if T1 or T3 thresholds not met for that replicate 4. Normalize to change in WT frequency 5. Normalize to frequency at T1 6. Calculate a fitness from slope of line in log space

```
#Calculate frequency data for each data frame (i.e. divide by total number of reads)
norm_return_freq = function(sub){
  #calculate total counts
  totals = apply(sub,2,sum)
  
  #divide each count by total count
  sub=sweep(sub, 2, unlist(totals), "/")
  names(sub)=c("r1t1_f","r1t2_f","r1t3_f","r2t1_f","r2t2_f","r2t3_f","r3t1_f","r3t2_f","r3t3_f")
  return(sub)
}

#Apply thresholds for T1 and T3 frequencies
apply_thresh = function(sub, t1_thresh, t3_thresh){
  #first combine frequencies of all 100 "WT" strains
  sub=rbind(apply(sub[1:100,],2,sum),sub[101:dim(sub)[1],])
  
  #For each replicate, check T1 threshold, set all three time points to 0 if not met
  for(i in c(1,4,7)){
    set_zero = which(sub[,i] < t1_thresh)
    if(length(set_zero>0)){sub[set_zero,i:(i+2)]=0}
  }
  #For each replicate, check T3 threshold, set all three time points to 0 if not met
  for(i in c(3,6,9)){
    set_zero = which(sub[,i] < t3_thresh)
    if(length(set_zero>0)){sub[set_zero,(i-2):i]=0}
  }
  names(sub)=c("r1t1_f","r1t2_f","r1t3_f","r2t1_f","r2t2_f","r2t3_f","r3t1_f","r3t2_f","r3t3_f")
  return(sub)
}

#Normalize frequency to wt frequency and frequency at T1
norm = function(sub){
  #calculate WT normalization factor
  wtsum = sub[1,]
  for (i in 3:1){
    wtsum[i]=wtsum[1]/wtsum[i]
    }
  for (i in 6:4){
    wtsum[i]=wtsum[4]/wtsum[i]
    }
  for (i in 9:7){
    wtsum[i]=wtsum[7]/wtsum[i]
    }
  
  #multiply each mutant strain's line by WT normalization
  sub = sweep(sub[2:dim(sub)[1],], 2, unlist(wtsum), "*")
  
  #divide each frequency by frequency at T1 for that replicate
  for (i in 3:1){
    sub[,i]=sub[,i]/sub[,1]
    }
  for (i in 6:4){
    sub[,i]=sub[,i]/sub[,4]
    }
  for (i in 9:7){
    sub[,i]=sub[,i]/sub[,7]
    }
  names(sub)=c("r1t1_nf","r1t2_nf","r1t3_nf","r2t1_nf","r2t2_nf","r2t3_nf","r3t1_nf","r3t2_nf","r3t3_nf")
  return(sub)
}  

#Calculate fitness
#require all three time points to make a fitness estimate
fit=function(sub,gens){
  estimates = NA
  for(i in 1:dim(sub)[1]){
    line = sub[i,]
    #check if strain is below detection limit
    if(is.na(line[1])){
      estimates[i]=NA
      next
      }
    #calculate fitness
    estimates[i]=(1+coefficients(lm(unlist(log(line))~0+gens))[1])
    }
  return(estimates)
}

#Wrapper function to call all of the above sub functions and plot QC results
analyze_condition = function(counts, gens,thresholdT1,thresholdT3,name){
  print(name)
  #normalize data
  dat_freq = norm_return_freq(counts)
  dat_freq_filt = apply_thresh(dat_freq,thresholdT1,thresholdT3)
  print(apply(dat_freq_filt,2,function(x)length(which(x==0))))
  dat_freq_norm = norm(dat_freq_filt)
  all_dat = cbind(counts[101:dim(counts)[1],],
                      dat_freq[101:dim(dat_freq)[1],],
                      dat_freq_norm)
  print("calculating fitness, may take a couple minutes")
  all_dat$r1 = fit(all_dat[,19:21],gens)
  all_dat$r2 = fit(all_dat[,22:24],gens)
  all_dat$r3 = fit(all_dat[,25:27],gens)
  print("fitness done")
  #record strain info and mean/sd
  all_dat$strain = rownames(all_dat)
  all_dat$query = key$query[-which(key$category=="wt")]
  all_dat$array = key$array[-which(key$category=="wt")]
  all_dat$category = key$category[-which(key$category=="wt")]
  all_dat$mean = apply(all_dat[,28:30],1,mean)
  all_dat$sd = apply(all_dat[,28:30],1,sd)
  #print(head(all_dat$sd))
  
  #plots to look at reproducibility of estimates
  print(ggplot(all_dat)+geom_point(aes(x=r1,y=r2),alpha=0.1)+geom_abline(color="red")+xlim(-0.75,1.5)+ylim(-0.75,1.5)+ggtitle(name)+annotate("text",x=-0.5,y=1.25,label = paste("rho = ",round(cor.test(all_dat$r1,all_dat$r2,method="spearman")$est,digits=3),sep="")))
  print(ggplot(all_dat)+geom_point(aes(x=r1,y=r3),alpha=0.1)+geom_abline(color="red")+xlim(-0.75,1.5)+ylim(-0.75,1.5)+ggtitle(name)+annotate("text",x=-0.5,y=1.25,label = paste("rho = ",round(cor.test(all_dat$r1,all_dat$r3,method="spearman")$est,digits=3),sep="")))
  print(ggplot(all_dat)+geom_point(aes(x=r2,y=r3),alpha=0.1)+geom_abline(color="red")+xlim(-0.75,1.5)+ylim(-0.75,1.5)+ggtitle(name)+annotate("text",x=-0.5,y=1.25,label = paste("rho = ",round(cor.test(all_dat$r2,all_dat$r3,method="spearman")$est,digits=3),sep="")))
  
  print(ggplot(all_dat)+geom_histogram(aes(x=sd))+ggtitle(name)+geom_vline(xintercept=0.05)+xlab("SD across rep cultures")+
          annotate("text",x=0.2,y=1000,label=paste("median sd = ",round(median(all_dat$sd,na.rm=TRUE),digits=3),sep="")))
  print(median(all_dat$sd,na.rm=TRUE))
  return(all_dat)
}

ypd24_all_dat = analyze_condition(ypd24,ypd24_gen,threshold1,threshold3,"YPD 24hr")
```

```
## [1] "YPD 24hr"
## r1t1_f r1t2_f r1t3_f r2t1_f r2t2_f r2t3_f r3t1_f r3t2_f r3t3_f 
##     60     60     60     62     62     62     58     58     58 
## [1] "calculating fitness, may take a couple minutes"
## [1] "fitness done"
```

```
## Warning: Removed 79 rows containing missing values (geom_point).
```

```
## Warning: Removed 78 rows containing missing values (geom_point).
```

```
## Warning: Removed 80 rows containing missing values (geom_point).
```

```
## stat_bin: binwidth defaulted to range/30. Use 'binwidth = x' to adjust this.
```

```
## [1] 0.04291565
```

```
ypd48_all_dat = analyze_condition(ypd48,ypd48_gen,threshold1,threshold3,"YPD 48hr")
```

```
## [1] "YPD 48hr"
## r1t1_f r1t2_f r1t3_f r2t1_f r2t2_f r2t3_f r3t1_f r3t2_f r3t3_f 
##   1192   1192   1192   1208   1208   1208   1138   1138   1138 
## [1] "calculating fitness, may take a couple minutes"
## [1] "fitness done"
```

```
## Warning: Removed 1710 rows containing missing values (geom_point).
```

```
## Warning: Removed 1644 rows containing missing values (geom_point).
```

```
## Warning: Removed 1673 rows containing missing values (geom_point).
```

```
## stat_bin: binwidth defaulted to range/30. Use 'binwidth = x' to adjust this.
```

```
## [1] 0.03638845
```

```
ypd37_all_dat = analyze_condition(ypd37,ypd37_gen,threshold1,threshold3,"YPD 37C")
```

```
## [1] "YPD 37C"
## r1t1_f r1t2_f r1t3_f r2t1_f r2t2_f r2t3_f r3t1_f r3t2_f r3t3_f 
##    171    171    171    156    156    156    179    179    179 
## [1] "calculating fitness, may take a couple minutes"
## [1] "fitness done"
```

```
## Warning: Removed 227 rows containing missing values (geom_point).
```

```
## Warning: Removed 269 rows containing missing values (geom_point).
```

```
## Warning: Removed 256 rows containing missing values (geom_point).
```

```
## stat_bin: binwidth defaulted to range/30. Use 'binwidth = x' to adjust this.
```

```
## [1] 0.07176701
```

```
ypeg_all_dat = analyze_condition(ypeg,ypeg_gen,threshold1,threshold3,"YPEG")
```

```
## [1] "YPEG"
## r1t1_f r1t2_f r1t3_f r2t1_f r2t2_f r2t3_f r3t1_f r3t2_f r3t3_f 
##    452    452    452    617    617    617    596    596    596 
## [1] "calculating fitness, may take a couple minutes"
## [1] "fitness done"
```

```
## Warning: Removed 817 rows containing missing values (geom_point).
```

```
## Warning: Removed 786 rows containing missing values (geom_point).
```

```
## Warning: Removed 905 rows containing missing values (geom_point).
```

```
## stat_bin: binwidth defaulted to range/30. Use 'binwidth = x' to adjust this.
```

```
## [1] 0.06288007
```

```
ura_all_dat = analyze_condition(ura,ura_gen,threshold1,threshold3,"SC-URA")
```

```
## [1] "SC-URA"
## r1t1_f r1t2_f r1t3_f r2t1_f r2t2_f r2t3_f r3t1_f r3t2_f r3t3_f 
##     82     82     82     96     96     96     68     68     68 
## [1] "calculating fitness, may take a couple minutes"
## [1] "fitness done"
```

```
## Warning: Removed 116 rows containing missing values (geom_point).
```

```
## Warning: Removed 95 rows containing missing values (geom_point).
```

```
## Warning: Removed 110 rows containing missing values (geom_point).
```

```
## stat_bin: binwidth defaulted to range/30. Use 'binwidth = x' to adjust this.
```

```
## [1] 0.0608557
```

```
#remove count data, as it's now stored in new data frames
rm(ypd24)
rm(ypd48)
rm(ypd37)
rm(ypeg)
rm(ura)
rm(key)
```

Process data for before genetic interaction score calculations

```
#calculate mean starting frequency and remove columns with count and frequency data
process_fit_table = function(all_dat){
  temp = apply(all_dat[,c(10,13,16)],1,mean)
  all_dat = all_dat[,-c(1:27)]
  all_dat$avg_start_freq = temp
  return(all_dat)
  }

ypd24_doubles=process_fit_table(ypd24_all_dat)
ypd48_doubles=process_fit_table(ypd48_all_dat)
ypd37_doubles=process_fit_table(ypd37_all_dat)
ypeg_doubles=process_fit_table(ypeg_all_dat)
ura_doubles=process_fit_table(ura_all_dat)
```

Calculate genetic interactions score using multiplicative model and look at QC plots

```
calc_iscore = function(all_dat,name){
  print(name)
  singles = subset(all_dat,category=="single") 
  doubles = subset(all_dat,category=="double")
  doubles$reason_iscore_skipped = NA
  doubles$num_expected = NA
  doubles$min_expected = NA
  doubles$max_expected = NA
  doubles$mean_expected = NA
  doubles$sd_expected = NA
  doubles$range_sig = NA
  doubles$t_pval = NA
  doubles$array_mean = NA
  doubles$query_mean = NA
  
  print("calculating i scores (~15,000 total), printing every 500 done")
  for(i in 1:dim(doubles)[1]){
    if(is.na(doubles$mean[i])|doubles$mean[i]<0){
      doubles$reason_iscore_skipped[i] = "double_missing"
      } 
    if(i%%500==0){print(i)}
    #get single query replicate data
    sub_query = subset(singles,query==doubles$query[i])
    if(length(which(is.na(sub_query$mean)))==length(sub_query$mean)){
      doubles$reason_iscore_skipped = "query_missing"
      next
    }
    
    #get single array data
    sub_array = subset(singles,array==doubles$array[i])
    if(length(which(is.na(sub_array$mean)))==length(sub_array$mean)){
      doubles$reason_iscore_skipped[i] = "query_missing"
      next
    }
    
    #calculate all expectation values
    count=0
    expected_values = NA
    for(m in 1:dim(sub_array)[1]){
      for(k in 1:dim(sub_query)[1]){
        count = count + 1
        expected_values[count]=sub_query$mean[k]*sub_array$mean[m]
        }
      }
    
    #record min, max, and mean expectation values
    doubles$num_expected[i] = length(which(!is.na(expected_values)))
    doubles$min_expected[i] = min(expected_values,na.rm=TRUE)
    doubles$max_expected[i] = max(expected_values,na.rm=TRUE)
    doubles$mean_expected[i] = mean(expected_values, na.rm=TRUE)
    doubles$sd_expected[i] = sd(expected_values, na.rm=TRUE)
    doubles$array_mean[i] = mean(sub_array$mean,na.rm=TRUE)
    doubles$query_mean[i] = mean(sub_query$mean,na.rm=TRUE)
    
    #see whether 3 replicate double mutant values fall in range
    above = which(doubles[i,1:3]>doubles$max_expected[i])
    below = which(doubles[i,1:3]<doubles$min_expected[i])
    
    
    #if double mutant has fitness estimate record results of range method and t.test
    if(is.na(doubles$reason_iscore_skipped[i])){
      doubles$range_sig[i] = FALSE
      if(length(above)==3|length(below)==3){
        doubles$range_sig[i] = TRUE
        }
      doubles$t_pval[i] = t.test(expected_values,doubles[i,1:3])$p.value
      }
    }
  print("done calculating scores, now plotting results")
  doubles$i_score = doubles$mean - doubles$mean_expected
  print(ggplot(doubles)+geom_histogram(aes(x=i_score),binwidth=0.02)+geom_vline(xintercept=0)+ggtitle(name)+
          annotate("text",x=0.4,y=1000,label=paste("median iscore=",round(median(doubles$i_score,na.rm=TRUE),digits=3),sep="")))
  print(median(doubles$i_score,na.rm=TRUE))
  
  print(ggplot(doubles)+geom_point(aes(x=mean_expected,y=mean),alpha=0.1)+geom_abline(color="red")+
    xlab("Expected")+ylab("Observed")+ggtitle(name))
  
  p=ggplot(doubles,aes(x=array_mean,y=mean))+geom_abline(color="red")+ggtitle(name)+
    geom_abline(color="blue")+geom_abline(aes(slope=query_mean),color="red")+
    geom_point(color="black",alpha=0.3)
  print(p+facet_wrap(~query))
  return(doubles)
}  

ypd24_doubles = calc_iscore(ypd24_doubles,"YPD 24hr")
```

```
## [1] "YPD 24hr"
## [1] "calculating i scores (~15,000 total), printing every 500 done"
## [1] 500
## [1] 1000
## [1] 1500
## [1] 2000
## [1] 2500
## [1] 3000
## [1] 3500
## [1] 4000
## [1] 4500
## [1] 5000
## [1] 5500
## [1] 6000
## [1] 6500
## [1] 7000
## [1] 7500
## [1] 8000
## [1] 8500
## [1] 9000
## [1] 9500
## [1] 10000
## [1] 10500
## [1] 11000
## [1] 11500
## [1] 12000
## [1] 12500
## [1] 13000
## [1] 13500
## [1] 14000
## [1] 14500
## [1] 15000
## [1] "done calculating scores, now plotting results"
```

```
## [1] 0.04123428
```

```
## Warning: Removed 91 rows containing missing values (geom_point).
```

```
## Warning: Removed 3 rows containing missing values (geom_point).
```

```
## Warning: Removed 33 rows containing missing values (geom_point).
```

```
## Warning: Removed 4 rows containing missing values (geom_point).
```

```
## Warning: Removed 6 rows containing missing values (geom_point).
```

```
## Warning: Removed 1 rows containing missing values (geom_point).
```

```
## Warning: Removed 2 rows containing missing values (geom_point).
```

```
## Warning: Removed 3 rows containing missing values (geom_point).
```

```
## Warning: Removed 8 rows containing missing values (geom_point).
```

```
## Warning: Removed 8 rows containing missing values (geom_point).
```

```
## Warning: Removed 2 rows containing missing values (geom_point).
```

```
## Warning: Removed 2 rows containing missing values (geom_point).
```

```
## Warning: Removed 1 rows containing missing values (geom_point).
```

```
## Warning: Removed 3 rows containing missing values (geom_point).
```

```
## Warning: Removed 3 rows containing missing values (geom_point).
```

```
## Warning: Removed 3 rows containing missing values (geom_point).
```

```
## Warning: Removed 5 rows containing missing values (geom_point).
```

```
## Warning: Removed 1 rows containing missing values (geom_point).
```

```
## Warning: Removed 3 rows containing missing values (geom_point).
```

```
ypd48_doubles = calc_iscore(ypd48_doubles,"YPD 48hr")
```

```
## [1] "YPD 48hr"
## [1] "calculating i scores (~15,000 total), printing every 500 done"
## [1] 500
## [1] 1000
## [1] 1500
## [1] 2000
## [1] 2500
## [1] 3000
## [1] 3500
## [1] 4000
## [1] 4500
## [1] 5000
## [1] 5500
## [1] 6000
## [1] 6500
## [1] 7000
## [1] 7500
## [1] 8000
## [1] 8500
## [1] 9000
## [1] 9500
## [1] 10000
## [1] 10500
## [1] 11000
## [1] 11500
## [1] 12000
## [1] 12500
## [1] 13000
## [1] 13500
## [1] 14000
## [1] 14500
## [1] 15000
## [1] "done calculating scores, now plotting results"
```

```
## [1] 0.02013564
```

```
## Warning: Removed 1960 rows containing missing values (geom_point).
```

```
## Warning: Removed 1 rows containing missing values (geom_segment).
```

```
## Warning: Removed 1 rows containing missing values (geom_segment).
```

```
## Warning: Removed 1 rows containing missing values (geom_segment).
```

```
## Warning: Removed 1 rows containing missing values (geom_segment).
```

```
## Warning: Removed 1 rows containing missing values (geom_segment).
```

```
## Warning: Removed 1 rows containing missing values (geom_segment).
```

```
## Warning: Removed 1 rows containing missing values (geom_segment).
```

```
## Warning: Removed 1 rows containing missing values (geom_segment).
```

```
## Warning: Removed 1 rows containing missing values (geom_segment).
```

```
## Warning: Removed 1 rows containing missing values (geom_segment).
```

```
## Warning: Removed 1 rows containing missing values (geom_segment).
```

```
## Warning: Removed 1 rows containing missing values (geom_segment).
```

```
## Warning: Removed 1 rows containing missing values (geom_segment).
```

```
## Warning: Removed 1 rows containing missing values (geom_segment).
```

```
## Warning: Removed 1 rows containing missing values (geom_segment).
```

```
## Warning: Removed 1 rows containing missing values (geom_segment).
```

```
## Warning: Removed 1 rows containing missing values (geom_segment).
```

```
## Warning: Removed 1 rows containing missing values (geom_segment).
```

```
## Warning: Removed 1 rows containing missing values (geom_segment).
```

```
## Warning: Removed 1 rows containing missing values (geom_segment).
```

```
## Warning: Removed 182 rows containing missing values (geom_point).
```

```
## Warning: Removed 16 rows containing missing values (geom_point).
```

```
## Warning: Removed 188 rows containing missing values (geom_point).
```

```
## Warning: Removed 489 rows containing missing values (geom_point).
```

```
## Warning: Removed 84 rows containing missing values (geom_point).
```

```
## Warning: Removed 50 rows containing missing values (geom_point).
```

```
## Warning: Removed 19 rows containing missing values (geom_point).
```

```
## Warning: Removed 57 rows containing missing values (geom_point).
```

```
## Warning: Removed 182 rows containing missing values (geom_point).
```

```
## Warning: Removed 94 rows containing missing values (geom_point).
```

```
## Warning: Removed 14 rows containing missing values (geom_point).
```

```
## Warning: Removed 36 rows containing missing values (geom_point).
```

```
## Warning: Removed 86 rows containing missing values (geom_point).
```

```
## Warning: Removed 24 rows containing missing values (geom_point).
```

```
## Warning: Removed 115 rows containing missing values (geom_point).
```

```
## Warning: Removed 68 rows containing missing values (geom_point).
```

```
## Warning: Removed 89 rows containing missing values (geom_point).
```

```
## Warning: Removed 91 rows containing missing values (geom_point).
```

```
## Warning: Removed 22 rows containing missing values (geom_point).
```

```
## Warning: Removed 54 rows containing missing values (geom_point).
```

```
ypd37_doubles = calc_iscore(ypd37_doubles,"YPD 37C")
```

```
## [1] "YPD 37C"
## [1] "calculating i scores (~15,000 total), printing every 500 done"
## [1] 500
## [1] 1000
## [1] 1500
## [1] 2000
## [1] 2500
## [1] 3000
## [1] 3500
## [1] 4000
## [1] 4500
## [1] 5000
## [1] 5500
## [1] 6000
## [1] 6500
## [1] 7000
## [1] 7500
## [1] 8000
## [1] 8500
## [1] 9000
## [1] 9500
## [1] 10000
## [1] 10500
## [1] 11000
## [1] 11500
## [1] 12000
## [1] 12500
## [1] 13000
## [1] 13500
## [1] 14000
## [1] 14500
## [1] 15000
## [1] "done calculating scores, now plotting results"
```

```
## [1] 0.0133926
```

```
## Warning: Removed 288 rows containing missing values (geom_point).
```

```
## Warning: Removed 1 rows containing missing values (geom_segment).
```

```
## Warning: Removed 1 rows containing missing values (geom_segment).
```

```
## Warning: Removed 1 rows containing missing values (geom_segment).
```

```
## Warning: Removed 1 rows containing missing values (geom_segment).
```

```
## Warning: Removed 1 rows containing missing values (geom_segment).
```

```
## Warning: Removed 1 rows containing missing values (geom_segment).
```

```
## Warning: Removed 1 rows containing missing values (geom_segment).
```

```
## Warning: Removed 1 rows containing missing values (geom_segment).
```

```
## Warning: Removed 1 rows containing missing values (geom_segment).
```

```
## Warning: Removed 1 rows containing missing values (geom_segment).
```

```
## Warning: Removed 1 rows containing missing values (geom_segment).
```

```
## Warning: Removed 1 rows containing missing values (geom_segment).
```

```
## Warning: Removed 1 rows containing missing values (geom_segment).
```

```
## Warning: Removed 1 rows containing missing values (geom_segment).
```

```
## Warning: Removed 1 rows containing missing values (geom_segment).
```

```
## Warning: Removed 1 rows containing missing values (geom_segment).
```

```
## Warning: Removed 1 rows containing missing values (geom_segment).
```

```
## Warning: Removed 1 rows containing missing values (geom_segment).
```

```
## Warning: Removed 1 rows containing missing values (geom_segment).
```

```
## Warning: Removed 1 rows containing missing values (geom_segment).
```

```
## Warning: Removed 8 rows containing missing values (geom_point).
```

```
## Warning: Removed 3 rows containing missing values (geom_point).
```

```
## Warning: Removed 93 rows containing missing values (geom_point).
```

```
## Warning: Removed 17 rows containing missing values (geom_point).
```

```
## Warning: Removed 23 rows containing missing values (geom_point).
```

```
## Warning: Removed 4 rows containing missing values (geom_point).
```

```
## Warning: Removed 2 rows containing missing values (geom_point).
```

```
## Warning: Removed 8 rows containing missing values (geom_point).
```

```
## Warning: Removed 56 rows containing missing values (geom_point).
```

```
## Warning: Removed 21 rows containing missing values (geom_point).
```

```
## Warning: Removed 2 rows containing missing values (geom_point).
```

```
## Warning: Removed 4 rows containing missing values (geom_point).
```

```
## Warning: Removed 4 rows containing missing values (geom_point).
```

```
## Warning: Removed 4 rows containing missing values (geom_point).
```

```
## Warning: Removed 8 rows containing missing values (geom_point).
```

```
## Warning: Removed 3 rows containing missing values (geom_point).
```

```
## Warning: Removed 5 rows containing missing values (geom_point).
```

```
## Warning: Removed 12 rows containing missing values (geom_point).
```

```
## Warning: Removed 3 rows containing missing values (geom_point).
```

```
## Warning: Removed 8 rows containing missing values (geom_point).
```

```
ypeg_doubles = calc_iscore(ypeg_doubles,"YPEG")
```

```
## [1] "YPEG"
## [1] "calculating i scores (~15,000 total), printing every 500 done"
## [1] 500
## [1] 1000
## [1] 1500
## [1] 2000
## [1] 2500
## [1] 3000
## [1] 3500
## [1] 4000
## [1] 4500
## [1] 5000
## [1] 5500
## [1] 6000
## [1] 6500
## [1] 7000
## [1] 7500
## [1] 8000
## [1] 8500
## [1] 9000
## [1] 9500
## [1] 10000
## [1] 10500
## [1] 11000
## [1] 11500
## [1] 12000
## [1] 12500
## [1] 13000
## [1] 13500
## [1] 14000
## [1] 14500
## [1] 15000
## [1] "done calculating scores, now plotting results"
```

```
## [1] 0.01390644
```

```
## Warning: Removed 1027 rows containing missing values (geom_point).
```

```
## Warning: Removed 1 rows containing missing values (geom_segment).
```

```
## Warning: Removed 1 rows containing missing values (geom_segment).
```

```
## Warning: Removed 1 rows containing missing values (geom_segment).
```

```
## Warning: Removed 1 rows containing missing values (geom_segment).
```

```
## Warning: Removed 1 rows containing missing values (geom_segment).
```

```
## Warning: Removed 1 rows containing missing values (geom_segment).
```

```
## Warning: Removed 1 rows containing missing values (geom_segment).
```

```
## Warning: Removed 1 rows containing missing values (geom_segment).
```

```
## Warning: Removed 1 rows containing missing values (geom_segment).
```

```
## Warning: Removed 1 rows containing missing values (geom_segment).
```

```
## Warning: Removed 1 rows containing missing values (geom_segment).
```

```
## Warning: Removed 1 rows containing missing values (geom_segment).
```

```
## Warning: Removed 1 rows containing missing values (geom_segment).
```

```
## Warning: Removed 1 rows containing missing values (geom_segment).
```

```
## Warning: Removed 1 rows containing missing values (geom_segment).
```

```
## Warning: Removed 1 rows containing missing values (geom_segment).
```

```
## Warning: Removed 1 rows containing missing values (geom_segment).
```

```
## Warning: Removed 1 rows containing missing values (geom_segment).
```

```
## Warning: Removed 1 rows containing missing values (geom_segment).
```

```
## Warning: Removed 1 rows containing missing values (geom_segment).
```

```
## Warning: Removed 32 rows containing missing values (geom_point).
```

```
## Warning: Removed 4 rows containing missing values (geom_point).
```

```
## Warning: Removed 163 rows containing missing values (geom_point).
```

```
## Warning: Removed 91 rows containing missing values (geom_point).
```

```
## Warning: Removed 88 rows containing missing values (geom_point).
```

```
## Warning: Removed 16 rows containing missing values (geom_point).
```

```
## Warning: Removed 6 rows containing missing values (geom_point).
```

```
## Warning: Removed 39 rows containing missing values (geom_point).
```

```
## Warning: Removed 307 rows containing missing values (geom_point).
```

```
## Warning: Removed 72 rows containing missing values (geom_point).
```

```
## Warning: Removed 4 rows containing missing values (geom_point).
```

```
## Warning: Removed 10 rows containing missing values (geom_point).
```

```
## Warning: Removed 13 rows containing missing values (geom_point).
```

```
## Warning: Removed 9 rows containing missing values (geom_point).
```

```
## Warning: Removed 34 rows containing missing values (geom_point).
```

```
## Warning: Removed 8 rows containing missing values (geom_point).
```

```
## Warning: Removed 33 rows containing missing values (geom_point).
```

```
## Warning: Removed 49 rows containing missing values (geom_point).
```

```
## Warning: Removed 7 rows containing missing values (geom_point).
```

```
## Warning: Removed 42 rows containing missing values (geom_point).
```

```
ura_doubles = calc_iscore(ura_doubles,"SC-URA")
```

```
## [1] "SC-URA"
## [1] "calculating i scores (~15,000 total), printing every 500 done"
## [1] 500
## [1] 1000
## [1] 1500
## [1] 2000
## [1] 2500
## [1] 3000
## [1] 3500
## [1] 4000
## [1] 4500
## [1] 5000
## [1] 5500
## [1] 6000
## [1] 6500
## [1] 7000
## [1] 7500
## [1] 8000
## [1] 8500
## [1] 9000
## [1] 9500
## [1] 10000
## [1] 10500
## [1] 11000
## [1] 11500
## [1] 12000
## [1] 12500
## [1] 13000
## [1] 13500
## [1] 14000
## [1] 14500
## [1] 15000
## [1] "done calculating scores, now plotting results"
```

```
## [1] 0.01942055
```

```
## Warning: Removed 122 rows containing missing values (geom_point).
```

```
## Warning: Removed 5 rows containing missing values (geom_point).
```

```
## Warning: Removed 33 rows containing missing values (geom_point).
```

```
## Warning: Removed 7 rows containing missing values (geom_point).
```

```
## Warning: Removed 12 rows containing missing values (geom_point).
```

```
## Warning: Removed 3 rows containing missing values (geom_point).
```

```
## Warning: Removed 2 rows containing missing values (geom_point).
```

```
## Warning: Removed 4 rows containing missing values (geom_point).
```

```
## Warning: Removed 15 rows containing missing values (geom_point).
```

```
## Warning: Removed 8 rows containing missing values (geom_point).
```

```
## Warning: Removed 2 rows containing missing values (geom_point).
```

```
## Warning: Removed 3 rows containing missing values (geom_point).
```

```
## Warning: Removed 2 rows containing missing values (geom_point).
```

```
## Warning: Removed 8 rows containing missing values (geom_point).
```

```
## Warning: Removed 3 rows containing missing values (geom_point).
```

```
## Warning: Removed 2 rows containing missing values (geom_point).
```

```
## Warning: Removed 7 rows containing missing values (geom_point).
```

```
## Warning: Removed 2 rows containing missing values (geom_point).
```

```
## Warning: Removed 4 rows containing missing values (geom_point).
```

Calculated genetic interaction scores empirically, using a linear model to estimate expectation for each group of double mutants generated from the same query guide measured in the same condition

```
queries = c("PRE7g7","PRE4g9","PRE4g3","RPN5g1","COG3g1","SED5g5","SEC22g1","SEC22g2","COG8g2","GET2g2","IMP4g6","DIP2g5","PWP2g2_BC1","PWP2g2_BC2","TIF6g8","RPF1g3","MAK16g1","YCR016Wg4","YLR050Cg1","SAP30g7")

get_corrected = function(dat,cond_name){
  
  dat$corrected_i_score = NA
  dat$corrected_expected = NA
  
  #loop through each query guide
  for(i in 1:20){
    
    rows = which(dat$query==queries[i])#find rows with query guide
    sub = subset(dat,query==queries[i])#subset data to these rows
    lin_fit = lm(sub$mean~sub$array_mean)#fit linear model from data for this query guide
    #for each strain carrying this query guide,
    #calculate and record expectation and deviation from expectation
    for(row in rows){
      xval = dat$array_mean[row]
      expected = lin_fit$coefficients[2]*xval+lin_fit$coefficients[1]
      observed = dat$mean[row]
      dat$corrected_expected[row]=expected
      dat$corrected_i_score[row]=observed-expected
      }
    }

  #plot histograms of gi scores by query guide
  p=ggplot(dat)+geom_histogram(aes(x=corrected_i_score))+geom_vline(xintercept=0)
  print(p+facet_wrap(~query))
  
  #print median and sd of gi scores
  print(median(dat$corrected_i_score,na.rm=TRUE))
  print(sd(dat$corrected_i_score,na.rm=TRUE))
  
  #plot points and model used for correction (red line is multiplicative mode, blue is empirical)
  p=ggplot(dat,aes(x=array_mean,y=mean))+geom_point(alpha=0.3,size=1)+
    geom_abline(aes(slope=query_mean),color="red",size=1)+
    ggtitle(cond_name)+geom_smooth(method="lm",size=1)
  p+facet_wrap(~query)
  return(dat)
}

ypd24_doubles=get_corrected(ypd24_doubles,"YPD24hr")
```

```
## stat_bin: binwidth defaulted to range/30. Use 'binwidth = x' to adjust this.
## stat_bin: binwidth defaulted to range/30. Use 'binwidth = x' to adjust this.
## stat_bin: binwidth defaulted to range/30. Use 'binwidth = x' to adjust this.
## stat_bin: binwidth defaulted to range/30. Use 'binwidth = x' to adjust this.
## stat_bin: binwidth defaulted to range/30. Use 'binwidth = x' to adjust this.
## stat_bin: binwidth defaulted to range/30. Use 'binwidth = x' to adjust this.
## stat_bin: binwidth defaulted to range/30. Use 'binwidth = x' to adjust this.
## stat_bin: binwidth defaulted to range/30. Use 'binwidth = x' to adjust this.
## stat_bin: binwidth defaulted to range/30. Use 'binwidth = x' to adjust this.
## stat_bin: binwidth defaulted to range/30. Use 'binwidth = x' to adjust this.
## stat_bin: binwidth defaulted to range/30. Use 'binwidth = x' to adjust this.
## stat_bin: binwidth defaulted to range/30. Use 'binwidth = x' to adjust this.
## stat_bin: binwidth defaulted to range/30. Use 'binwidth = x' to adjust this.
## stat_bin: binwidth defaulted to range/30. Use 'binwidth = x' to adjust this.
## stat_bin: binwidth defaulted to range/30. Use 'binwidth = x' to adjust this.
## stat_bin: binwidth defaulted to range/30. Use 'binwidth = x' to adjust this.
## stat_bin: binwidth defaulted to range/30. Use 'binwidth = x' to adjust this.
## stat_bin: binwidth defaulted to range/30. Use 'binwidth = x' to adjust this.
## stat_bin: binwidth defaulted to range/30. Use 'binwidth = x' to adjust this.
## stat_bin: binwidth defaulted to range/30. Use 'binwidth = x' to adjust this.
```

```
## [1] 0.001062496
## [1] 0.05894914
```

```
ypd48_doubles=get_corrected(ypd48_doubles,"YPD48hr")
```

```
## stat_bin: binwidth defaulted to range/30. Use 'binwidth = x' to adjust this.
## stat_bin: binwidth defaulted to range/30. Use 'binwidth = x' to adjust this.
## stat_bin: binwidth defaulted to range/30. Use 'binwidth = x' to adjust this.
## stat_bin: binwidth defaulted to range/30. Use 'binwidth = x' to adjust this.
## stat_bin: binwidth defaulted to range/30. Use 'binwidth = x' to adjust this.
## stat_bin: binwidth defaulted to range/30. Use 'binwidth = x' to adjust this.
## stat_bin: binwidth defaulted to range/30. Use 'binwidth = x' to adjust this.
## stat_bin: binwidth defaulted to range/30. Use 'binwidth = x' to adjust this.
## stat_bin: binwidth defaulted to range/30. Use 'binwidth = x' to adjust this.
## stat_bin: binwidth defaulted to range/30. Use 'binwidth = x' to adjust this.
## stat_bin: binwidth defaulted to range/30. Use 'binwidth = x' to adjust this.
## stat_bin: binwidth defaulted to range/30. Use 'binwidth = x' to adjust this.
## stat_bin: binwidth defaulted to range/30. Use 'binwidth = x' to adjust this.
## stat_bin: binwidth defaulted to range/30. Use 'binwidth = x' to adjust this.
## stat_bin: binwidth defaulted to range/30. Use 'binwidth = x' to adjust this.
## stat_bin: binwidth defaulted to range/30. Use 'binwidth = x' to adjust this.
## stat_bin: binwidth defaulted to range/30. Use 'binwidth = x' to adjust this.
## stat_bin: binwidth defaulted to range/30. Use 'binwidth = x' to adjust this.
## stat_bin: binwidth defaulted to range/30. Use 'binwidth = x' to adjust this.
## stat_bin: binwidth defaulted to range/30. Use 'binwidth = x' to adjust this.
```

```
## [1] 0.001813365
## [1] 0.07846925
```

```
ypeg_doubles=get_corrected(ypeg_doubles,"YPEG")
```

```
## stat_bin: binwidth defaulted to range/30. Use 'binwidth = x' to adjust this.
## stat_bin: binwidth defaulted to range/30. Use 'binwidth = x' to adjust this.
## stat_bin: binwidth defaulted to range/30. Use 'binwidth = x' to adjust this.
## stat_bin: binwidth defaulted to range/30. Use 'binwidth = x' to adjust this.
## stat_bin: binwidth defaulted to range/30. Use 'binwidth = x' to adjust this.
## stat_bin: binwidth defaulted to range/30. Use 'binwidth = x' to adjust this.
## stat_bin: binwidth defaulted to range/30. Use 'binwidth = x' to adjust this.
## stat_bin: binwidth defaulted to range/30. Use 'binwidth = x' to adjust this.
## stat_bin: binwidth defaulted to range/30. Use 'binwidth = x' to adjust this.
## stat_bin: binwidth defaulted to range/30. Use 'binwidth = x' to adjust this.
## stat_bin: binwidth defaulted to range/30. Use 'binwidth = x' to adjust this.
## stat_bin: binwidth defaulted to range/30. Use 'binwidth = x' to adjust this.
## stat_bin: binwidth defaulted to range/30. Use 'binwidth = x' to adjust this.
## stat_bin: binwidth defaulted to range/30. Use 'binwidth = x' to adjust this.
## stat_bin: binwidth defaulted to range/30. Use 'binwidth = x' to adjust this.
## stat_bin: binwidth defaulted to range/30. Use 'binwidth = x' to adjust this.
## stat_bin: binwidth defaulted to range/30. Use 'binwidth = x' to adjust this.
## stat_bin: binwidth defaulted to range/30. Use 'binwidth = x' to adjust this.
## stat_bin: binwidth defaulted to range/30. Use 'binwidth = x' to adjust this.
## stat_bin: binwidth defaulted to range/30. Use 'binwidth = x' to adjust this.
```

```
## [1] 0.003694326
## [1] 0.107496
```

```
ypd37_doubles=get_corrected(ypd37_doubles,"YPD37C")
```

```
## stat_bin: binwidth defaulted to range/30. Use 'binwidth = x' to adjust this.
## stat_bin: binwidth defaulted to range/30. Use 'binwidth = x' to adjust this.
## stat_bin: binwidth defaulted to range/30. Use 'binwidth = x' to adjust this.
## stat_bin: binwidth defaulted to range/30. Use 'binwidth = x' to adjust this.
## stat_bin: binwidth defaulted to range/30. Use 'binwidth = x' to adjust this.
## stat_bin: binwidth defaulted to range/30. Use 'binwidth = x' to adjust this.
## stat_bin: binwidth defaulted to range/30. Use 'binwidth = x' to adjust this.
## stat_bin: binwidth defaulted to range/30. Use 'binwidth = x' to adjust this.
## stat_bin: binwidth defaulted to range/30. Use 'binwidth = x' to adjust this.
## stat_bin: binwidth defaulted to range/30. Use 'binwidth = x' to adjust this.
## stat_bin: binwidth defaulted to range/30. Use 'binwidth = x' to adjust this.
## stat_bin: binwidth defaulted to range/30. Use 'binwidth = x' to adjust this.
## stat_bin: binwidth defaulted to range/30. Use 'binwidth = x' to adjust this.
## stat_bin: binwidth defaulted to range/30. Use 'binwidth = x' to adjust this.
## stat_bin: binwidth defaulted to range/30. Use 'binwidth = x' to adjust this.
## stat_bin: binwidth defaulted to range/30. Use 'binwidth = x' to adjust this.
## stat_bin: binwidth defaulted to range/30. Use 'binwidth = x' to adjust this.
## stat_bin: binwidth defaulted to range/30. Use 'binwidth = x' to adjust this.
## stat_bin: binwidth defaulted to range/30. Use 'binwidth = x' to adjust this.
## stat_bin: binwidth defaulted to range/30. Use 'binwidth = x' to adjust this.
```

```
## [1] 0.0008836626
## [1] 0.05536485
```

```
ura_doubles=get_corrected(ura_doubles,"SCURA")
```

```
## stat_bin: binwidth defaulted to range/30. Use 'binwidth = x' to adjust this.
## stat_bin: binwidth defaulted to range/30. Use 'binwidth = x' to adjust this.
## stat_bin: binwidth defaulted to range/30. Use 'binwidth = x' to adjust this.
## stat_bin: binwidth defaulted to range/30. Use 'binwidth = x' to adjust this.
## stat_bin: binwidth defaulted to range/30. Use 'binwidth = x' to adjust this.
## stat_bin: binwidth defaulted to range/30. Use 'binwidth = x' to adjust this.
## stat_bin: binwidth defaulted to range/30. Use 'binwidth = x' to adjust this.
## stat_bin: binwidth defaulted to range/30. Use 'binwidth = x' to adjust this.
## stat_bin: binwidth defaulted to range/30. Use 'binwidth = x' to adjust this.
## stat_bin: binwidth defaulted to range/30. Use 'binwidth = x' to adjust this.
## stat_bin: binwidth defaulted to range/30. Use 'binwidth = x' to adjust this.
## stat_bin: binwidth defaulted to range/30. Use 'binwidth = x' to adjust this.
## stat_bin: binwidth defaulted to range/30. Use 'binwidth = x' to adjust this.
## stat_bin: binwidth defaulted to range/30. Use 'binwidth = x' to adjust this.
## stat_bin: binwidth defaulted to range/30. Use 'binwidth = x' to adjust this.
## stat_bin: binwidth defaulted to range/30. Use 'binwidth = x' to adjust this.
## stat_bin: binwidth defaulted to range/30. Use 'binwidth = x' to adjust this.
## stat_bin: binwidth defaulted to range/30. Use 'binwidth = x' to adjust this.
## stat_bin: binwidth defaulted to range/30. Use 'binwidth = x' to adjust this.
## stat_bin: binwidth defaulted to range/30. Use 'binwidth = x' to adjust this.
```

```
## [1] 0.0006842162
## [1] 0.06418071
```

For each condition, save a long and short format data file: Long format should contain all data calculated in this file. Short format should have strain information, fitness in each replicate, mean and sd of fitness, and genetic interaction score (empirical for all guides except SAP30g7, for which use multiplicative model).

```
get_short=function(dat){
  dat_short = dat[,c(4:6,1:3,8,9,21,22)]
  dat_short$gi_score = dat_short$corrected_i_score
  dat_short$gi_score[which(dat_short$query=="SAP30g7")]=dat_short$i_score[which(dat_short$query=="SAP30g7")]
  return(dat_short[,-c(9:10)])
  }

write.table(get_short(ypd24_doubles),"~/Desktop/SherlockLab2/manuscript_aug2017/code/ypd24_short.txt",sep="\t")
write.table(get_short(ypd48_doubles),"~/Desktop/SherlockLab2/manuscript_aug2017/code/ypd48_short.txt",sep="\t")
write.table(get_short(ypeg_doubles),"~/Desktop/SherlockLab2/manuscript_aug2017/code/ypeg_short.txt",sep="\t")
write.table(get_short(ypd37_doubles),"~/Desktop/SherlockLab2/manuscript_aug2017/code/ypd37_short.txt",sep="\t")
write.table(get_short(ura_doubles),"~/Desktop/SherlockLab2/manuscript_aug2017/code/ura_short.txt",sep="\t")

get_long = function(dat1,dat2){
  dat1 = cbind(dat1,data.frame(matrix(nrow=dim(dat1)[1],ncol=14)))
  names(dat1)[37:50]=names(dat2)[10:23]
  dat1[1770:16969,37:50]=dat2[,10:23]
  return(dat1)
}

write.table(get_long(ypd24_all_dat,ypd24_doubles),"~/Desktop/SherlockLab2/manuscript_aug2017/code/ypd24_data.txt",sep="\t")
write.table(get_long(ypd48_all_dat,ypd48_doubles),"~/Desktop/SherlockLab2/manuscript_aug2017/code/ypd48_data.txt",sep="\t")
write.table(get_long(ypeg_all_dat,ypeg_doubles),"~/Desktop/SherlockLab2/manuscript_aug2017/code/ypeg_data.txt",sep="\t")
write.table(get_long(ypd37_all_dat,ypd37_doubles),"~/Desktop/SherlockLab2/manuscript_aug2017/code/ypd37_data.txt",sep="\t")
write.table(get_long(ura_all_dat,ura_doubles),"~/Desktop/SherlockLab2/manuscript_aug2017/code/ura_data.txt",sep="\t")
```
